# Parp7 generates an ADP-ribosyl degron that controls negative feedback of androgen signaling

**DOI:** 10.1038/s44318-025-00510-4

**Published:** 2025-07-18

**Authors:** Krzysztof Wierbiłowicz, Chun-Song Yang, Ahmed Almaghasilah, Patryk A Wesołowski, Philipp Pracht, Natalia M Dworak, Jack Masur, Sven Wijngaarden, Dmitri V Filippov, David J Wales, Joshua B Kelley, Aakrosh Ratan, Bryce M Paschal

**Affiliations:** 1 Department of Biochemistry and Molecular Genetics, University of Virginia School of Medicine, PO Box 800733, Charlottesville, VA 22908 USA; 2 Department of Genome Sciences, University of Virginia School of Medicine, Charlottesville, VA USA; 3 Department of Molecular and Biomedical Sciences, University of Maine, Orono, ME USA; 4 Yusuf Hamied Department of Chemistry, University of Cambridge, Lensfield Road, Cambridge, CB2 1EW UK; 5 Advanced Microscopy Facility, University of Virginia School of Medicine, Charlottesville, VA 22908 USA; 6 University of Virginia Comprehensive Cancer Center, Charlottesville, VA 22903 USA; 7 Leiden Institute of Chemistry, Leiden University, Einsteinweg 55, Leiden, 2333 CC The Netherlands

**Keywords:** AR, ADP-ribosylation, Ubiquitin, DTX2, RBN2397, Chromatin, Transcription & Genomics, Post-translational Modifications & Proteolysis

## Abstract

The androgen receptor (AR) transduces the effects of circulating and tumor-derived androgens to the nucleus through ligand-induced changes in protein conformation, localization, and chromatin engagement. Defining how these events are integrated with signal transduction is critical to understand how AR drives prostate cancer and unveil pathway features that are potentially amenable to therapeutic intervention. We describe a novel post-transcriptional mechanism that controls AR levels on chromatin and gene output based on highly selective, inducible degradation. We find that the mono-ADP-ribosyltransferase PARP7 generates an ADP-ribosyl degron in the DNA-binding domain of AR, which is recognized by the ADP-ribose reader domain in the ubiquitin E3 ligase DTX2 and degraded by the proteasome. Mathematical modeling of the pathway suggested that PARP7 ADP-ribosylates chromatin-bound AR, a prediction that was validated in cells using an AR DNA-binding mutant. Non-conventional ubiquitin conjugation to ADP-ribosyl-cysteine and degradation by the proteasome forms the basis of a negative feedback loop that regulates modules of AR target genes. Our data expand the repertoire of mono-ADP-ribosyltransferases to include gene regulation via highly selective protein degradation.

## Synopsis

PARP7 modifies chromatin-bound androgen receptor (AR) by generating an ADP-ribosyl-cysteine degron, which marks AR for degradation. The degron is recognized by the E3 ligase DTX2, which mediates ubiquitin conjugation of ADP-ribosylated AR en route to its proteasomal degradation.

- The AR transcriptome in prostate cancer cells includes gene modules with temporal expression patterns indicative of negative feedback.
- Inhibition of PARP7 with RBN2397 stabilizes AR protein levels and disrupts the negative feedback loop, suggesting a functional link between PARP7 mediated AR ADP-ribosylation, AR turnover, and transcriptional output.
- Molecular modeling accurately predicts that PARP7 mono-ADP-ribosylation of AR occurs on chromatin and regulates AR protein stability.
- Reconstitution experiments with model peptides show the E2/E3 reaction generates a Ub-ADP-ribosyl-cysteine, independent of lysine residues in the substrate.

## Abstract (web summary)

Mono-ADP-ribosylation of the agonist conformation of AR marks it for degradation via DTX2, an E3 ligase with ADP-ribose reader function.

## Graphical abstract

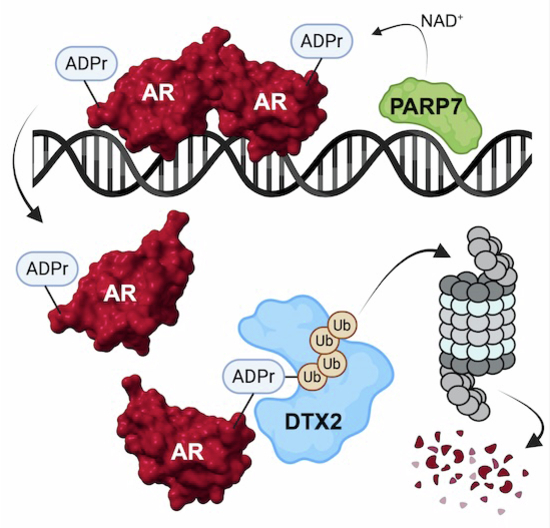

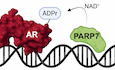

## Introduction

The poly-ADP-ribosyltransferase (PARP) family of enzymes use NAD^+^ as a co-substrate for conjugation of ADP-ribose to substrates. Most PARP family members mediate mono-ADP-ribosylation, though a subset including PARP1 and PARP2 generate poly-ADP-ribose chains (PAR) (Dasovich and Leung, 2023). As the founding member of the PARP family, PARP1 has been studied extensively, especially in the context of DNA repair and cancer. PARP1/2 inhibitors are FDA-approved for the treatment of several cancers, with the greatest activity against tumors with homologous recombination deficiency (Lord and Ashworth, 2017). For the PARP family members that mediate mono-ADP-ribosylation, major knowledge gaps limit our understanding of associated biology and therapeutic opportunities. What is clear is that the mono-ADP-ribosyltransferases participate in a variety of biological pathways that impact major cellular events, including gene expression, cell identity, and immune function, among others (Kraus, 2020; Luscher et al, 2022).

The exact mechanisms by which ADP-ribosylation regulates protein function and cellular outcomes remain an area of active investigation. PARP1 auto-modification promotes its release from chromatin, and PARP1-generated poly-ADP-ribose chains provide scaffolds at damage sites that concentrate DNA repair factors (Duma and Ahel, 2023; Pascal, 2023). This observation indicates that ADP-ribosylation, like several other post-translational modifications, including those occurring in the nucleus, can regulate protein dynamics. Protein modules with three-dimensional structures known to read ADP-ribosylation are the macrodomain, Deltex C-terminal (DTC) domain, Tryptophan-Tryptophan-Glutamate (WWE) domain, and the PAR-binding zinc-finger (PBZ) domain (Karras et al, 2005; Rack et al, 2016; Suskiewicz et al, 2023). As these structural modules occur in a variety of proteins, including some PARP enzymes, ADP-ribose readers can act as ADP-ribosylation effectors that direct PARP- and substrate-specific outcomes.

PARP activity in the nucleus has also been shown to intersect with processes associated with transcription. Multiple studies have linked PARP1 and PARP2 to transcription through effects on chromatin structure (Zong et al, 2022). Another example of PARP regulation of transcriptional output involves the mono-ADP-ribosyltransferase 2,3,7,8-Tetrachlorodibenzodioxin-inducible PARP (TIPARP), also known as PARP7 (Popova et al, 2025). Induction of PARP7 by the aryl hydrocarbon receptor is part of a mechanism that limits the AHR-mediated xenobiotic response, which otherwise results in hepatotoxicity (Ahmed et al, 2015; MacPherson et al, 2013). Our group identified PARP7 in the context of androgen signaling through AR (Yang et al, 2021). AR directly induces PARP7 expression, which mono-ADP-ribosylates AR on Cys residues; these ADP-ribosyl-Cys sites are read by PARP9 macrodomains in the DTX3L/PARP9 complex and result in the assembly of a DTX3L/PARP9-AR complex (Yang et al, 2021). Thus, PARP7 induction, substrate (AR) mono-ADP ribosylation, and reading by a complex that contains an E3 ligase for histone mono-ubiquitylation (Takeyama et al, 2003) represents a multi-step mechanism that contributes to AR regulation of gene expression. A notable feature of the mechanism is that AR ADP ribosylation by PARP7 is specific for the agonist conformation and occurs on eleven cysteines (Kamata et al, 2021b; Yang et al, 2021). While the exact stoichiometry of ADP ribosylation remains to be determined, the number of ADP-ribosyl-cysteines in AR generated by PARP7 exceeds the number of macrodomains in the oligomeric DTX3L/PARP9 complex (Vela-Rodriguez et al, 2024). This raised the question of whether ADP-ribosylation sites in AR might engage with other reader proteins that affect transcription.

Here, we use gene expression analysis to show that PARP7 activity has a profound effect on androgen signaling by regulating the expression of distinct modules of AR target genes through negative feedback. The mechanism is based on the androgen-induced degradation of AR, which is mediated by the ubiquitin (Ub) E3 ligase, DTX2. Our biochemical analysis shows that DTX2 uses an ADP-ribose reader domain to recognize and conjugate Ub to ADP-ribosyl-cysteine in AR. These data, together with other recent work (Ahmed et al, 2020; Zhu et al, 2022) indicate that PARP activity, reader function, and non-canonical ubiquitylation can be combined for highly selective protein degradation. In the case of androgen signaling, ADP ribosylation and turnover of chromatin-associated AR illustrate how PARP-mediated degradation of a transcription factor can shape the transcriptome of prostate cancer cells.

## Results

**Figure 1:**
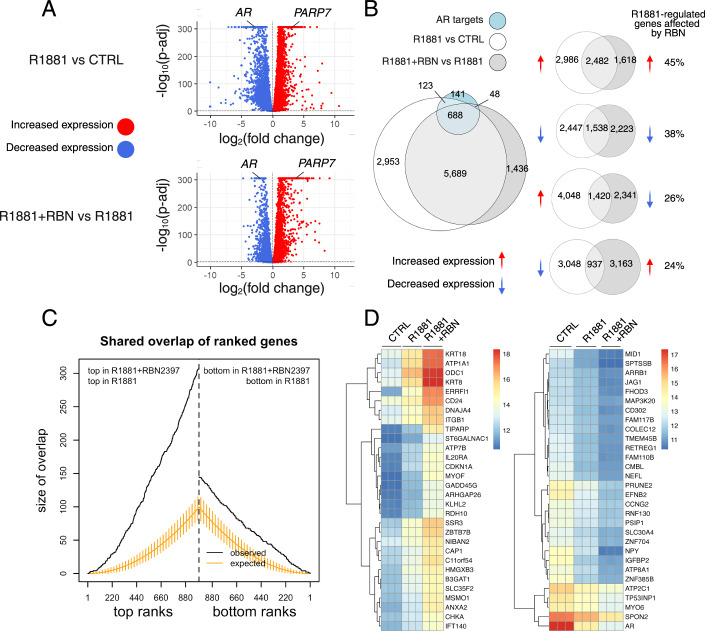
Androgen-regulated gene expression is sensitive to PARP7 inhibition by RBN2397. (**A**) Volcano plots showing differential gene expression analysis in the VCaP cell line across three experimental conditions: untreated (CTRL), treated with R1881 (R1881), and treated with R1881 + RBN2397 (R1881 + RBN). The top plot compares gene expression between R1881-treated and untreated samples (R1881 vs. CTRL), while the bottom plot compares gene expression between R1881 + RBN-treated samples to R1881-treated samples (R1881 + RBN vs. R1881), using R1881 as the control. Each dot represents a gene. The *x* axis shows the log_2_ fold change (Log_2_FC) and the *y* axis shows the negative log_10_ of the adjusted *P* value (*P*-adj). Genes that have significantly increased or decreased expression (*P*-adj <0.001) are highlighted in red and blue, respectively. The AR and PARP7 genes are indicated on the plots. The experiment was done with three biological replicates, and the *P* values were calculated using the Wald test with multiple testing correction—Benjamini–Hochberg method. (**B**) Venn diagrams visualizing the overlap between differentially expressed genes (*P*-adj <0.001) in two comparisons: R1881 vs CTRL (white) and R1881 + RBN vs R1881 (gray) differentially expressed genes (*P*-adj <0.001), alongside androgen receptor AR gene target genes (blue), identified through integration of AR ChIP-seq and RNA-seq from R1881-treated VCaP cells). The Venn diagrams on the right show PARP7 inhibition with RBN2397 affects the expression of R1881-sensitive genes, whether they are R1881-induced or R1881-repressed. The largest group of genes affected by RBN2397 treatment are those which are positively regulated by R1881 and undergo a further increase (45%). The second largest group are negatively regulated by R1881 and undergo a further decrease (38%). We also show genes that are upregulated by R1881 but downregulated by RBN2397, and vice versa. (**C**) Plot illustrating the overlap of differentially expressed genes between the R1881 vs. CTRL and R1881 + RBN vs. R1881 comparisons. On the *x* axis, “top ranks” represent genes with increased expression in both comparisons, while “bottom ranks” correspond to genes with decreased expression. The y axis shows the number of overlapping genes, plotted as a step function across ranked genes based on the Wald statistic. A dotted line separates the “top ranks” and “bottom ranks.” Only the top 1000 and bottom 1000 ranked genes are shown. For reference, the expected overlap and corresponding 95% confidence intervals, calculated from an empirical distribution obtained through subsampling, are also displayed. Notably, genes with increased expression in both comparisons exhibit a much larger overlap. The experiment was done with three biological replicates, and the lines represent the means. (**D**) Heatmaps displaying the top 30 genes contributing to the similarity score in (**C**) (left— increased expression with R1881, right—decreased expression with R1881). The left heatmap shows genes with increased expression upon R1881 treatment, while the right shows those with decreased expression. Columns represent experimental groups: CTRL, R1881, and R1881 + RBN. Expression values were normalized using variance-stabilizing transformation (VST). The color gradient used for visualization is indicated in the figure. The dendrograms to the left of each heatmap illustrate gene clustering based on expression patterns, grouping more similar genes together. Source data are available online for this figure.

PARP7 ADP ribosylation of AR results in selective recruitment of the DTX3L/PARP9 complex (Yang et al, 2021). PARP9 provides ADP-ribose reader function while DTX3L contributes E3 mono-ubiquitylation activity, potentially for substrates such as core histones (Yan et al, 2009; Yang et al, 2017). Because depletion of DTX3L/PARP9 affects the expression of only 6% of the AR transcriptome (Yang et al, 2021), we hypothesized that multi-site AR ADP ribosylation by PARP7 might contribute to AR regulation through additional mechanisms. To explore this hypothesis, we used a potent PARP7 inhibitor, RBN2397, that eliminates AR ADP ribosylation in prostate cancer cells (Gozgit et al, 2021; Yang et al, 2023). We queried the effects of PARP7 inhibition on androgen signaling in the vertebral metastasis-derived prostate cancer line VCaP using RNA-seq. VCaP cells were treated +R1881 (synthetic androgen) and +RBN2397 for 18 h (h), harvested, and processed. As expected, R1881 treatment affected the expression of a very large number of genes (9453; using adjusted *P* value (*P*-adj) <0.001), with similar numbers of genes showing increased and decreased expression (Fig. 1A, upper plot). A comparison of R1881 + RBN2397 vs. R1881 alone showed a substantial effect of PARP7 inhibition, with 7861 genes differentially expressed using the same significance threshold (Fig. 1A, lower plot). In contrast, RBN2397 treatment alone affected only 19 genes (Appendix Fig. S1A). When tested for overlap of differentially expressed genes (DEGs), 67% of the androgen-regulated genes were affected by PARP7 inhibition (Fig. 1B). The overlap included 688 genes that are among the top 1000 direct AR target genes identified by CistromeGO in VCaP cells (Li et al, 2019). These data show that PARP7 plays a major role in androgen signaling.

PARP7 inhibition exhibited a trend where genes upregulated by R1881 showed further increased expression with R1881 + RBN2397 cotreatment, while genes downregulated by R1881 were further suppressed by the addition of RBN2397 (Fig. 1B). The largest percentage of androgen-sensitive genes affected by PARP7 inhibition (45%) were those positively regulated by R1881 (Fig. 1B). To better understand the effects of PARP7 inhibition, we quantified the similarity between the ranked lists of genes from the two comparisons. Figure 1C shows the number of overlaps between the top- and bottom-ranked genes based on the Wald statistic in the two comparisons, along with the expected overlap and corresponding 95% confidence intervals calculated from an empirical distribution obtained through subsampling. The number of shared genes with increased expression in this analysis is striking, suggesting the genes whose expression was most elevated by R1881 are also among the genes that are most elevated by PARP7 inhibition. Genes that contribute most to this similarity clearly exhibit a stronger RBN2397 effect compared to the response to R1881 alone in the same direction (Fig. 1D). We also used our previously generated RNA-seq from DTX3L knockdown (KD), R1881-treated VCaP cells, to test for enrichment in R1881 + RBN2397 vs. R1881 DEGs (Appendix Fig. S1B). We used DTX3L KD-sensitive genes from R1881-treated samples, excluding genes whose basal expression levels were affected by DTX3L KD, to create a gene set. We then performed gene set enrichment analysis (GSEA) to test the enrichment of R1881 + RBN2397 vs. R1881 DEGs in that gene set. This analysis showed no enrichment. Taken together, these data suggest that PARP7 can act as a negative regulator of AR-dependent gene expression, and the effect is separable from the E3 function provided by DTX3L.

### Modules of AR-dependent gene expression

Our finding that genes whose expression was most affected by RBN2397 also showed a relatively small effect of R1881 alone in the same direction led us to hypothesize that PARP7 exerts negative feedback on AR, apparent at the 18-h time point. PARP7 is known to exert negative feedback on AHR-regulated genes such as CYP1A1 (MacPherson et al, 2013). We envisioned that negative feedback in prostate cancer cells, initiated by AR induction of PARP7, could temporally regulate the effects of circulating and tumor-derived androgens and potentially affect disease progression.

**Figure 2:**
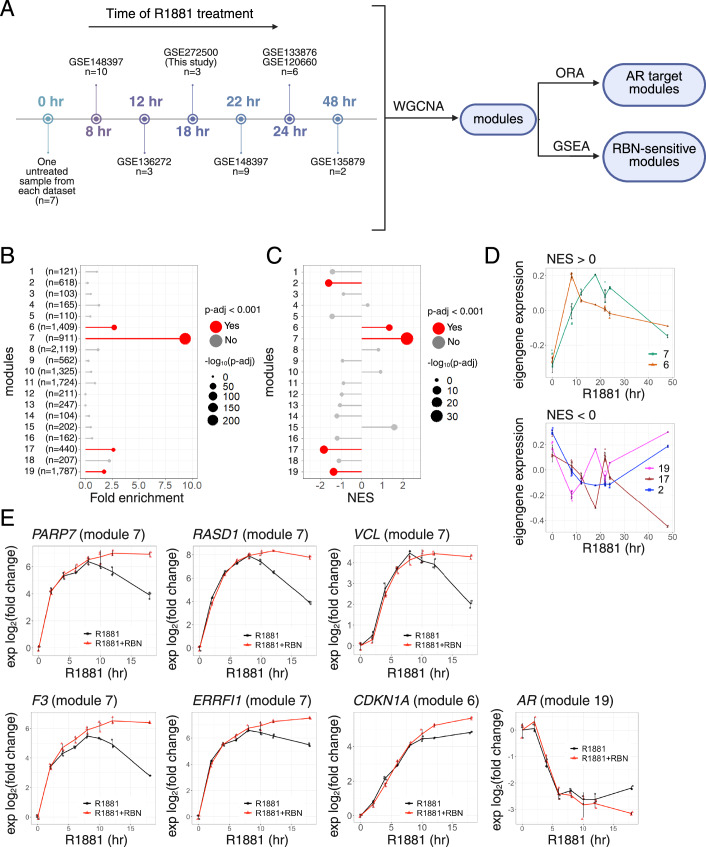
PARP7 regulates temporal patterns of AR-dependent gene expression through negative feedback. (**A**) Scheme visualizing the analysis workflow. The left side presents RNA-seq datasets used to create the compiled gene expression time course of androgen treatment in the VCaP cell line. The GEO accession number and the number of samples are shown for each time point. The right side presents the analysis workflow (WGCNA Weighted gene co-expression network analysis, ORA Overrepresentation analysis, GSEA Gene set enrichment analysis). (**B**) Dot plot showing the gene modules overrepresented in the set of 1000 AR target genes. The gene lists of modules only with more than 100 genes resulting from WGCNA were used as gene sets for ORA. The *y* axis represents the modules (the number (*n*) of genes is provided in parentheses for each module), and the x axis represents the fold enrichment. The dot size represents −log_10_(p-adj), and the dots colored in red represent modules with *P*-adj <0.001. The *P* values were calculated using the hypergeometric test with multiple testing correction—Benjamini–Hochberg method. (**C**) Dot plot showing the gene modules enriched in the R1881 + RBN vs R1881 differentially expressed genes. The gene lists of modules only with more than 100 genes resulting from WGCNA were used as gene sets for GSEA. The y axis represents the modules, and the *x* axis represents the Normalized enrichment score (NES). The dot size represents −log_10_(*P*-adj), and the dots colored in red represent modules with *P*-adj <0.001. The *P* values were calculated using the permutation-based approach. (**D**) Line plot showing the eigengene expression for significantly enriched modules from GSEA in (**C**). The plot on the top shows modules with positive NES (7 and 6), and the plot on the bottom shows modules with negative NES (19, 17, and 2). The *y* axis represents the eigengene expression, and the *x* axis represents the time of androgen treatment. The lines represent the means and the error bars represent standard deviation (0 h: *n* = 7; 8 h: *n* = 10; 12 h: *n* = 3; 18 h: *n* = 3; 22 h: *n* = 9; 24 h: *n* = 6; 48 h: *n* = 2; *n* represents number of biological replicates). (**E**) Line plots showing the results of a time course RT-qPCR experiment in VCaP cells treated with R1881 (black) and cotreated with R1881 and RBN3297 (red). The *y* axis represents the log_2_ of the expression fold change from 0 h time point normalized to the GUS housekeeping gene, and the *x* axis represents the time of treatment in hours. The module membership is shown in parentheses for each gene. The lines represent the means and the error bars represent standard deviation (*n* = 3; *n* represents number of biological replicates). Source data are available online for this figure.

To study the temporal effects of androgen signaling and AR-dependent gene expression in VCaP cells, we used publicly available RNA-seq data (R1881 treatment for 8, 12, 18, 22, 24, 48 h) to compile an androgen treatment time course (Fig. 2A). This analysis enabled the investigation of AR-dependent gene expression on a transcriptome scale. We employed weighted gene co-expression network analysis (WGCNA) to identify modules of genes that co-express with similar time-dependence (Langfelder and Horvath, 2008). Each module is summarized by a weighted average expression profile known as the module eigengene, which corresponds to the first principal component of the module. From the initial 32 modules identified by the analysis, we retained 19 modules, each containing >100 genes (Appendix Fig. S2).

We first identified the modules that were enriched for AR targets. Using modules as gene sets, we tested for enrichment of the 1,000 AR target genes identified by CistromeGO in VCaP cells using overrepresentation analysis (ORA). Using a 0.001 *P*-adj cut-off, four modules (6, 7, 17, 19) emerged as significant, with Module 7 showing the highest fold enrichment (Fig. 2B). Using GSEA, we also looked for modules that were enriched for R1881 + RBN2397 vs. R1881 DEGs. Strikingly, this analysis identified the same modules and, additionally, module 2 (Fig. 2C). Plotting the eigengene expression values of the five modules as a function of R1881 treatment time showed that modules with positive enrichment (NES > 0) in RBN2397-sensitive genes showed a rapid increase in expression with peaks at 8 h (module 7) and 18 h (module 6), followed by a steady decrease in expression (Fig. 2D). In contrast, modules with negative enrichment (NES < 0) in RBN2397-sensitive genes showed reduced expression, with troughs at 8 h (module 17) and 18 h (modules 2, 19), and subsequent increase in expression. Overall, the five modules illustrate the kinetic effects of androgen signaling in VCaP cells, and integrating these findings with our RNA-seq data suggests that PARP7 activity has a prominent role in shaping the AR transcriptome.

### PARP7 activity and module kinetics

To validate the results from the compiled dataset analysis, we performed a time course of R1881 and RBN2397 treatment in VCaP cells and used quantitative PCR (qPCR) to measure the expression of genes from select modules. We focused on a set of RBN2397-sensitive genes from module 7 since it had the highest enrichment score (Fig. 2B,C). We also included *CDKNA1* (module 6) and AR (module 19) for their biological and clinical relevance. All module 7 genes tested (*PARP7, RASD1, VCL, F3, ERRFI1*) displayed similar expression kinetics reflective of the module eigengene generated from compiled RNA-seq datasets (Fig. 2D,E). These kinetics feature a steep induction, a peak of transcription at approximately 8 hr, and a subsequent decrease, the effect of which was reduced by RBN2397 treatment (Fig. 2E). For comparison, the module 6 gene *CDKN1A* had peak expression at 18 h with a slight fold-increase in response to RBN2397. Lastly, the module 19 gene AR showed a steep R1881-dependent drop and was only affected by RBN2397 during a late recovery phase (12–18 h). These results confirm that PARP7 controls a negative feedback mechanism that acts preferentially on AR target genes.

### Androgen-induced degradation of AR

**Figure 3:**
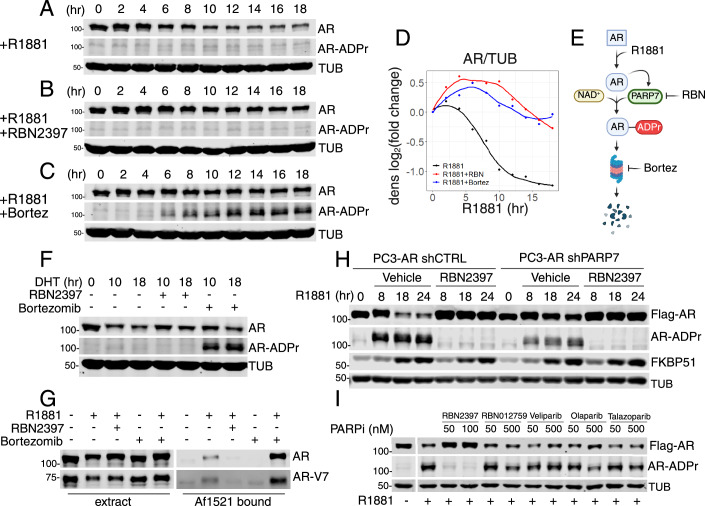
Androgen-induced ADP ribosylation by PARP7 targets AR for proteasomal degradation. (**A**–**C**) Immunoblot detection of AR and AR-ADPr (by FL-AF1521) in VCaP cells subjected to a time course of R1881 (**A**), R1881 and RBN2397 (**B**), or R1881 and Bortezomib (Bortez) (C) treatment. Cells were collected every 2 h for an 18-h period. (**D**) Line plot visualizing the AR protein density measurements for immunoblots from (**A**–**C**). The *y* axis represents the log_2_ of the AR/TUB density fold change from 0-h time point, and the *x* axis represents the time of treatment in hours. The curves were fitted using the loess method. Data points represent single measurements. (**E**) Schematic diagram of the androgen-induced AR degradation mechanism. (**F**) Immunoblot detection of AR and AR-ADPr (Fl-AF1521) in VCaP cells treated with DHT, DHT + RBN2397, and DHT+Bortezomib for times indicated on the panel. (**G**) Immunoblot detection of the AR and AR-V7 protein in VCaP cell extracts and AF1521 bound fractions. Cell extracts from VCaP cells treated with different combinations of R1881, RBN2397, and Bortezomib for 10 h and were combined with AF1521 beads for the enrichment of ADP-ribosylated proteins. (**H**) Immunoblot detection of Flag-AR, AR-ADPr, and FKBP51 in PC3-AR shCTRL (left) and shPARP7 (right) cells treated with R1881 and cotreated with R1881 and RBN2397 for times indicated on the panel. The shGFP was used as shCTRL. (**I**) Immunoblot detection of Flag-AR and AR-ADPr in PC3-AR cells treated for 18 h with R1881 and different PARP inhibitors (RBN2397—PARP7i; RBN012759—PARP14i; Veliparib, Olaparib, and Talazoparib—PARP1/2i). Source data are available online for this figure.

Given the temporal effects of androgen on certain modules of gene expression, we used immunoblotting to query if the transcriptional changes were associated with AR protein levels. In VCaP cells, we observed a decline in AR protein levels beyond 4 h of R1881 treatment (Fig. 3A), consistent with prior work showing that AR represses its own transcription (Cai et al, 2011). The reduction in AR protein level was blunted by PARP7 inhibition (Fig. 3B). Though this finding suggested a link between PARP7 activity and AR degradation, ADP-ribosylated AR was not readily detected by blotting using fluorescently labeled AF1521 (Yang et al, 2021) in extracts from VCaP cells exposed to R1881 in culture (Fig. 3A). This observation is despite the fact that AR undergoes androgen- and PARP7-dependent ADP ribosylation in response to androgen (Yang et al, 2021). ADP-ribosylated AR was, however, detected when VCaP cells were cotreated with R1881 and the proteasome inhibitor Bortezomib (Fig. 3C,D; +R1881+Bortez). The splice variant AR-V7 in VCaP cells also showed an androgen-induced degradation and Bortez-dependent accumulation, similar to full-length AR (Appendix Fig. S3A–D). In addition, AR levels were reduced in VCaP cells treated with nanomolar concentrations of dihydrotestosterone (DHT), a physiological ligand that is more metabolically labile than the synthetic compound R1881 (Appendix Fig. S3E).

### PARP7 marks AR for destruction by the proteasome

DHT- and Bortez-co-treatment resulted in the accumulation of ADP-ribosylated AR, consistent with a model where ADP ribosylation marks AR for degradation by the proteasome (Fig. 3E,F). The model is supported by pull-down assays using the recombinant macrodomain from *Archaeoglobus fulgidus* immobilized on beads (GST-AF1521) (Kamata et al, 2019). There was an increased recovery of ADP-ribosylated AR using extract from Bortez-treated cells (Fig. 3G). Partial depletion of PARP7 by shRNA (Yang et al, 2023) was sufficient to reduce the androgen enhancement of AR degradation; this effect was particularly evident at the 18 and 24 h time points (Fig. 3H). Although LSD1 recruitment mediates transcriptional repression of the AR gene (Cai et al, 2011), the results with Bortez (Fig. 3C,E–G), together with the fact that androgen-induced reduction in AR protein level occurs with expression from an ectopic promoter, establish an important contribution from a post-transcriptional, ADP-ribosylation-linked mechanism. The absence of AR transcriptional repression in PC3-AR cells may explain why longer periods of androgen exposure are necessary to reduce AR protein levels (Appendix Fig. S3F). Results with inhibitors to other PARP enzymes (PARP1, 2, 14) including several used clinically support the conclusion that AR ADP-ribosylation and androgen-induced degradation can be attributed to PARP7 (Fig. 3I).

### Mathematical model of PARP7 regulation of AR

PARP7-mediated AR degradation appears to explain the reduced AR output observed after 8 h of androgen treatment and is consistent with regulation by negative feedback. To explore whether our description of the PARP7-AR relationship accounts for the timing of the negative feedback mechanism, we developed an ordinary differential equation (ODE) based computational model of AR-PARP7 interactions and downstream transcriptional outputs. The model architecture includes AR and PARP7, each undergoing degradation at a basal and faster rate (Kamata et al, 2021a). It was assumed that the cell contains 6.6 AR molecules per promoter (based on 20,000 AR molecules—all ligand-bound—that can associate with 3050 responsive promoters (Bruchovsky et al, 1975)). AR transcript levels (Fig. 2E) were included in the model since AR represses its own transcription (Cai et al, 2011). Other key assumptions were that PARP7 drives AR ADP ribosylation and that AR-ADPr is degraded at a different rate from the total pool of AR. An androgen-induced agonist conformation and nuclear localization are required for AR ADP ribosylation by PARP7, which in prostate cancer cells is primarily a nuclear enzyme (Kamata et al, 2021b). We estimated the parameters using a Monte Carlo approach with 600,000 iterations (Model Parameters in Appendix Table S1).

**Figure 4:**
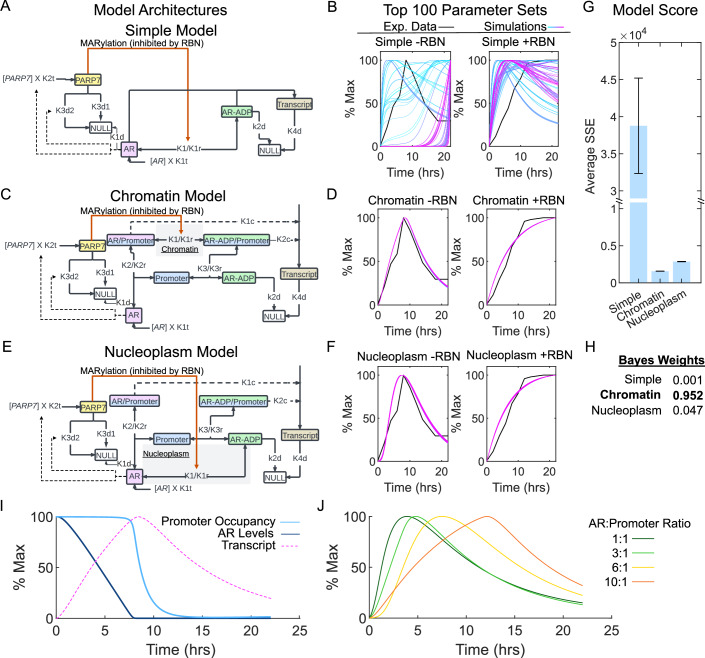
Mathematical modeling of androgen-induced AR degradation suggests that PARP7 acts upon chromatin-bound AR. (**A**) Schematic diagram of the Simple Model architecture that does not explicitly consider AR-chromatin binding to generate transcript. (**B**) Plots of experimental transcript and simulated transcript for the top 100 parameter sets during R1881 treatment, with and without RBN2397 using the Simple Model architecture. Data are normalized to have a maximal value of 100. (**C**) Schematic diagram of the Chromatin Model architecture where AR bound to chromatin generates transcript and PARP7 acts upon AR only when bound to chromatin. (**D**) Plots of experimental transcript and simulated transcript for the top 100 parameter sets during R1881 treatment, with and without RBN2397 using the Chromatin Model architecture. Data is normalized to have a maximal value of 100. (**E**) Schematic diagram of the Nucleoplasm Model architecture that where AR bound to chromatin generates transcript and PARP7 acts upon AR only in the nucleoplasm. (**F**) Plots of experimental transcript and simulated transcript for the top 100 parameter sets during R1881 treatment, with and without RBN2397 using the Nucleoplasm Model architecture. Data is normalized to have a maximal value of 100. (**G**) Graph of model performance for each model architecture, measured by the sum of squared error (SSE) after 600,000 Monte Carlo simulations to find the best parameters for each. The lower the SSE score, the better the simulated data matches the experimental data. For each model, top 100 scores were used to calculate *P* values (pairwise *t* test, *n* = 100 different parameter sets), all data significantly different for *P* < 0.0001 (simple vs chromatin *P* = 1.7 × 10^−78^, simple vs nuclear *P* = 9.8 × 10^−77^, chromatin vs nuclear *P* = 1.32 × 10^−214^). The error bars represent the standard deviation of the data. (**H**) Bayes weights calculated from the Bayes Information Criterion (BIC) of each model. (**I**) Simulated AR levels, promoter occupancy, and transcriptional output from the top parameter set using the Chromatin Model. (**J**) Simulations using the Chromatin Model with differing starting levels of AR to have the indicated ratio to promoters in the model. Increasing AR:Promoter increases the duration of the delay in negative feedback. Source data are available online for this figure.

We began with a simple model in which a transcript is generated directly by active AR (Fig. 4A). Examination of the top 100 performing parameter sets (Appendix Table S2) for this model architecture shows a poor fit to the experimental data, and no convergence of the model (Fig. 4B). We then included an explicit AR-promoter complex, which must form to generate transcript. When this compartment was added to the model, it raised the question, should PARP7 act upon AR that is bound to chromatin, or should it act upon AR in the nucleoplasm? We generated models with both architectures to test which better captured the experimental data. In both models, we allow modified and unmodified forms of AR to interact with promoters.

The Chromatin Model (Fig. 4C) best fit the shape and behavior of the experimental data and the top 100 parameters converge on a very similar output (Fig. 4D). The Nucleoplasm model also captured the shape of the experimental data well when compared to the Simple Model, and the top 100 parameters converge on a similar output (Fig. 4E). However, close examination reveals a slight delay in the beginning of transcript production and an earlier peak than in the data (Fig. 4F), errors that were not observed in the Chromatin Model. When we compare the average of the Sum of Square Errors (SSE) for the top 100 simulations, both the Chromatin Model and the Nucleoplasm Model perform far better (lower SSE) than the Simple Model, with the Chromatin Model showing the lowest average SSE (Fig. 4G). We compared the models by calculating their Bayesian Information Criterion (Appendix Table S3), an approach to evaluate models that considers SSE and number of parameters. We then calculated the Bayes Weights as a statistical test of the best model (Appendix Table S3). Based on these tests, the Chromatin Model with a Bayesian Weight of 0.952 (Fig. 4H) is most likely to be the correct model (*P* < 0.05).

In both models that feature AR-promoter interaction, the parameters that best fit the data result in a scenario where ADP-ribosylated AR has a lower affinity for the promoter than unmodified AR. In the Chromatin Model, the excess AR relative to promoter binding sites provides a mechanism where only a subset of AR molecules are available for PARP7 modification at a given time. Together with the low starting concentration of PARP7, this mechanism could explain the multi-hour delay of negative feedback, which is clear in module 7 genes (Fig. 2). The Chromatin Model predicts a continual decrease in AR until the protein levels become low enough to reduce promoter occupancy (Fig. 4I). This implies that in our model, the level of excess AR could dictate the time of delay preceding the decrease in transcriptional output. We tested the Chromatin Model with varying AR levels to adjust the initial AR:Promoter ratio and found that this ratio is responsible for the delayed decrease in transcriptional output in our modeling (Fig. 4J). Our modeling overestimates the amount of AR that has to be degraded (Fig. 4I compared to Fig. 3D), but our model considers no other forms of negative feedback to AR; this leaves degradation as the only mechanism in this model architecture to account for AR inhibition. This difference accounts for the inactive AR level and does not affect our ability to interpret the model. Our mathematical modeling indicates that PARP7-induced degradation of AR is sufficient to capture the transcriptional effects of PARP7 inhibition (given the assumptions about AR regulation) and suggests that ADP ribosylation of AR likely occurs on chromatin.

### Validation that AR ADP ribosylation requires DNA binding

**Figure 5:**
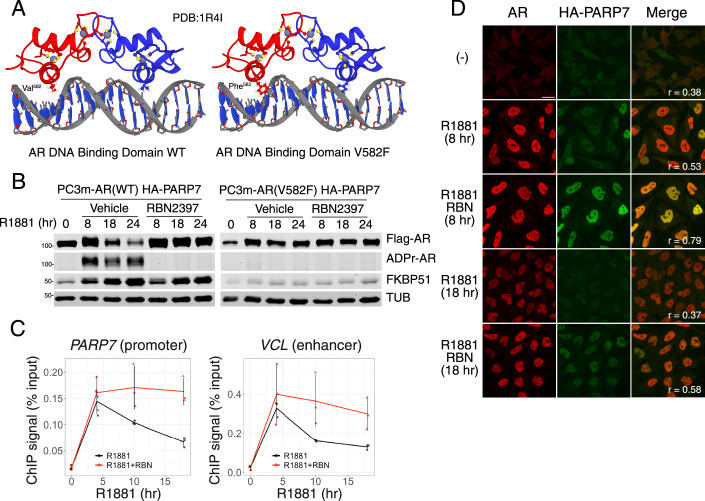
Androgen-induced AR degradation depends on DNA binding and affects chromatin occupancy. (**A**) AR DNA-binding domain structure (1R4I) in its wild-type form (left) and with V582F amino acid substitution (right). (**B**) Immunoblot detection of Flag-AR, AR-ADPr (by FL-AF1521), and FKBP51 in PC3m(HA-PARP7/Flag-AR WT) cells (left) and PC3m(HA-PARP7/Flag-AR V582F mutant) cells (right) treated with R1881 or cotreated with R1881 and RBN2397 for times indicated on the panel. (**C**) Line plots showing the results of a time course ChIP-qPCR experiment in VCaP cells treated with R1881 (black) or cotreated with R1881 and RBN3297 (red). The *y* axis represents the ChIP signal characterized as the percentage of input DNA, and the *x* axis represents the time of treatment in hours. The lines represent the means, and the error bars represent standard deviation (*n* = 3; *n* represents number of biological replicates). (**D**) Confocal microscopy staining of HA-PARP7 and AR in PC3-AR(HA-PARP7) cells treated with R1881 or cotreated with R1881 and RBN2397 for times (0–18 h) indicated on the panel. Images were also taken after 42 h drug treatment (Appendix Fig. S4 along with the same re-displayed 0 h time point to allow comparison). The third column shows merged channels, and the Pearson correlation coefficient for pixel co-localization is indicated in the bottom left corner for every condition. A scale bar of 10 µm is provided on the bottom right corner of the upper left panel and applies to all panels. Source data are available online for this figure.

We next tested the prediction from the Chromatin Model that PARP7 ADP ribosylation of AR occurs upon DNA binding, using an AR exon2 loss-of-function mutation (V582F) from a French family with complete androgen insensitivity syndrome that maps to the DNA recognition helix (Fig. 5A; described originally as V581F) (Lumbroso et al, 1993). The V582F substitution prevents DNA binding, but it does not detectably alter AR affinity for androgen, or the androgen-induced conformation necessary for AR phosphorylation (Black et al, 2004; Lobaccaro et al, 1996). In PC3m-AR(V582F) cells, the mutant AR showed virtually no androgen-induced ADP ribosylation nor androgen-induced AR degradation (Fig. 5B). As expected, the DNA-binding mutation V582F abrogated androgen induction of the AR target gene *FKBP51* (Fig. 5B).

Our data place the ADP-ribosylation and AR degradation mechanism in the context of chromatin. Consistent with this view, ADP-ribosylated AR can be crosslinked and detected at the androgen-responsive promoter and enhancer sites (Yang et al, 2021). PARP7-dependent degradation that reduces AR occupancy on the promoters of affected genes could provide an explanation for negative feedback. We tested this by chromatin immunoprecipitation followed by qPCR (ChIP-qPCR) for AR as a function of androgen treatment time using two androgen-regulated genes that are RBN2397-sensitive (PARP7 and VCL). We observed that AR occupancy on these genes was maximal after ~4 h of androgen treatment, at which point the PARP7-mediated negative feedback became apparent (Fig. 5C). This effect was largely prevented by RBN2397 treatment (Fig. 5C). Thus, negative feedback via PARP7 manifests as a reduction in AR-chromatin occupancy. Using confocal microscopy in a prostate cancer cell line expressing epitope-tagged AR and PARP7, we performed pixel-wise co-localization analysis. Based on Pearson’s correlation coefficients, AR and PARP7 distributions were correlated in cells treated with androgen (*r* = 0.53) and increased when cotreated with RBN2397 (*r* = 0.79) (Fig. 5D; Appendix Fig. S4). Taken together, these data suggest a mechanism for how androgen binding to AR in the cytoplasm culminates in AR ADP ribosylation and degradation on chromatin.

### Cysteine ADP ribosylation drives AR degradation

**Figure 6:**
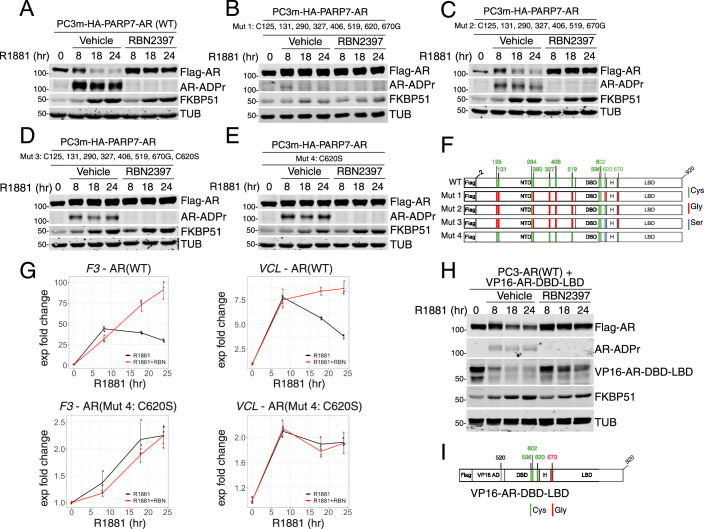
The DBD mediates androgen-induced degradation of AR. (**A**–**E**) Immunoblot detection of Flag-AR, AR-ADPr (by FL-AF1521), and FKBP51 in PC3m(HA-PARP7/Flag-AR wild type or mutant) cells treated with R1881 or cotreated with R1881 and RBN2397 for times indicated on the panel. (**A**) AR WT; (**B**) AR Mut1 (C125,131,290,327,406,519,620,670G); (**C**) AR Mut2 (C125,131,290,327,406,519,670G); (**D**) AR Mut3 (C125,131,290,327,406,519,670G & C620S); (**E**) AR Mut4 (C620S). (**F**) Diagrams of Flag-AR mutants employed in this figure (Mut1–4). All of the ADP-ribosylation cysteine sites on AR are marked in green, and the particular substitutions are marked in red for glycine and blue for serine. (**G**) Line plots showing the results of a time course RT-qPCR experiment in PC3m(HA-PARP7/Flag-AR WT) cells (upper panels) and PC3m(HA-PARP7/Flag-AR C620S mutant: Mut4) cells (lower panels) treated with R1881 (black) and cotreated with R1881 and RBN2397 (red). The *y* axis represents the log_2_ of the expression fold change from 0 h time point normalized to the GUS housekeeping gene, and the x axis represents the time of treatment in hours. The lines represent the means, and the error bars represent standard deviation (*n* = 3; *n* represents the number of biological replicates). (**H**) Immunoblot detection of VP16-AR-DBD-LBD stably co-expressed with WT AR in PC3 cells. The cells were treated with R1881 or cotreated with R1881 and the PARP7 inhibitor RBN2397 for the times indicated on the panel. The VP16-AR-DBD-LBD expresses at about 10% of the WT AR. (**I**) Diagram of the VP16-AR-DBD-LBD fusion. Previously described ADP-ribosylation cysteine sites in the AR DBD are marked in green,VP16-AR-DBD-LBD fusion contains a glycine substitution in the Cys670 ADP-ribosylation site. Source data are available online for this figure.

Previously we demonstrated that ADP-ribosyl-Cys modifications in the unstructured AR N-terminal domain (NTD) are recognized by macrodomains in the oligomeric DTX3L/PARP9 complex as part of a protein assembly mechanism (Yang et al, 2021). To determine which ADP-ribosyl-Cys sites are responsible for PARP7-mediated AR degradation, we used a panel of ten AR mutants with amino acid substitutions (Gly, Ser) for Cys sites shown by mass spectrometry (Yang et al, 2021) to undergo androgen-induced ADP ribosylation in prostate cancer cells (Fig. 6A–F; Appendix Fig. S5A–G; Appendix Table S4). Glycine is inactive for ADP ribosylation, while serine (similarity to cysteine) can serve as an ADP-ribose acceptor for some PARPs (Suskiewicz et al, 2021). Mutating eight ADP-ribosylation sites (Fig. 6B,F; Mut1) virtually eliminated androgen-induced AR ADP ribosylation and degradation, but this set of mutations also reduced the transcription function of AR based on impaired induction of the direct target gene, *FKBP51* (Fig. 6A,B). Restoring amino acid 620 in Mut1 to Cys to create Mut2 rescued both androgen-induced degradation and the appearance of ADP-ribose on AR (Fig. 6C,F). ADP-ribose was detected by blotting using fluorescently labeled recombinant AF1521^tandem^ (FL-AF1521), and by pull-down using AF1521^tandem^ immobilized on beads (Appendix Fig. S5H). Substituting a Ser at position 620 in the context of a multi-site mutant (Mut3; Fig. 6D,F) or as a single-site mutant Cys620Ser (Mut4; Fig. 6E,F) failed to restore androgen-induced degradation, possibly because PARP7 shows a preference for Cys over Ser for ADP ribosylation (Rodriguez et al, 2021; Yang et al, 2021). Notably, mutation of Cys620 was sufficient to eliminate androgen-induced degradation of AR Mut10 (Appendix Fig. S5F,G). This result suggests that Cys620 in the DBD is linked to androgen-induced AR degradation.

### DNA binding increases the solvent accessibility of AR Cys620

In co-crystal structures of the AR DNA-binding domain (DBD) bound to cognate DNA (Lee et al, 2024; Shaffer et al, 2004) Cys620 localizes to an alpha-helix within the DBD core, where it hydrogen bonds to several atoms within the same alpha-helix. Since DNA binding is required for AR ADP ribosylation, we used energy minimization to evaluate the effect of DNA and structural flexibility on the total energy and solvent-accessible surface area (SASA) of three androgen receptor DNA-binding domain (AR DBD) structures: two using the human DBD (C3 DNA, MTV DNA) and another with the rat DBD (DR3 DNA). We added hydrogen atoms to the PDB entries and optimized the structures using GFN-FF with the ALPB implicit solvent Model (Ehlert et al, 2021; Pracht et al, 2024; Spicher and Grimme, 2020; Wesolowski et al, 2024). Charges were initialized using the charge-extended Hückel (CEH) method, originally developed in the context of the charge-adaptive q-vSZP basis set (Muller et al, 2023), and the structures were then re-optimized under the same conditions. During this step, we computed the total SASA and per-residue SASA for each chain in each structure. Total energies (Appendix Table S5) and SASA values (Appendix Table S6) were computed before and after geometry optimization, including values for three cysteines in the DBD that can be ADP-ribosylated by PARP7 (Cys596, Cys602, and Cys620) (Yang et al, 2021). The SASA analysis showed that prior to DNA binding, Cys620 has much less solvent exposure than Cys596 and Cys602 (zinc coordinating), but Cys620 SASA increases in response to DNA binding (Appendix Table S6). The Cys620 SASA values were increased from 0.0227 to 0.0452 by C3 DNA, from 0.0387 to 0.1920 by MMTV DNA, and from 0.1308 to 0.542 by DR3 DNA. The SASA change at Cys620 was more pronounced in the A chain of the DNA-bound dimer, which is interesting given the Claessens group showed the DNA-bound A and B chains are not structurally identical (Lee et al, 2024). DNA-induced accessibility of AR Cys620 could help restrict PARP7 modification of this site to chromatin-bound AR.

While the gene module analysis was performed in VCaP cells, we determined that module 7 genes (*F3* and *VCL*) were similarly affected by PARP7 inhibition in the PC3 cell background (Fig. 6G, upper panels). In this setting, the Cys620 to Ser substitution stabilized AR (Fig. 6E) and rendered the androgen-dependent output of *F3* and *VCL* resistant to the effect of PARP7 inhibition (Fig. 6G). We observed that R1881-induced activation of the F3 and VCL genes with the AR(Mut4) was less than observed with AR(WT), raising the possibility that amino acid substitutions in the DBD could affect AR degradation by reducing DNA binding (Fig. 6G). On this note, AR has two additional ADP-ribosylation sites, that by mass spectrometry map to the second zinc finger in the DBD (Cys596 and Cys602) (Yang et al, 2021). Given the challenge of interpreting whether amino acid substitutions in the AR DBD affect the degradation mechanism independent of ADP ribosylation, we tested whether an artificial transcription factor that contains the WT AR DBD can undergo androgen- and PARP7-dependent degradation. We substituted the N-terminal AF1 domain (which contains most of the AR ADP-ribosylation sites) with the VP16 activation domain (AD). The AR LBD was retained to impart androgen regulation to the construct (VP16-AR-DBD-LBD; Fig. 6H,I). We also mutated Cys670 in the LBD to eliminate a contribution from this ADP-ribosylation site. We observed that VP16-AR-DBD-LBD undergoes androgen-induced degradation, and that degradation is prevented by PARP7 inhibition (Fig. 6H). These data lend support to the conclusion that the DBD in AR and PARP7 catalytic activity are critical features of the androgen-induced AR degradation and gene output mechanism.

### E3 ligase DTX2 controls the cellular level of ADP-ribosylated AR

**Figure 7:**
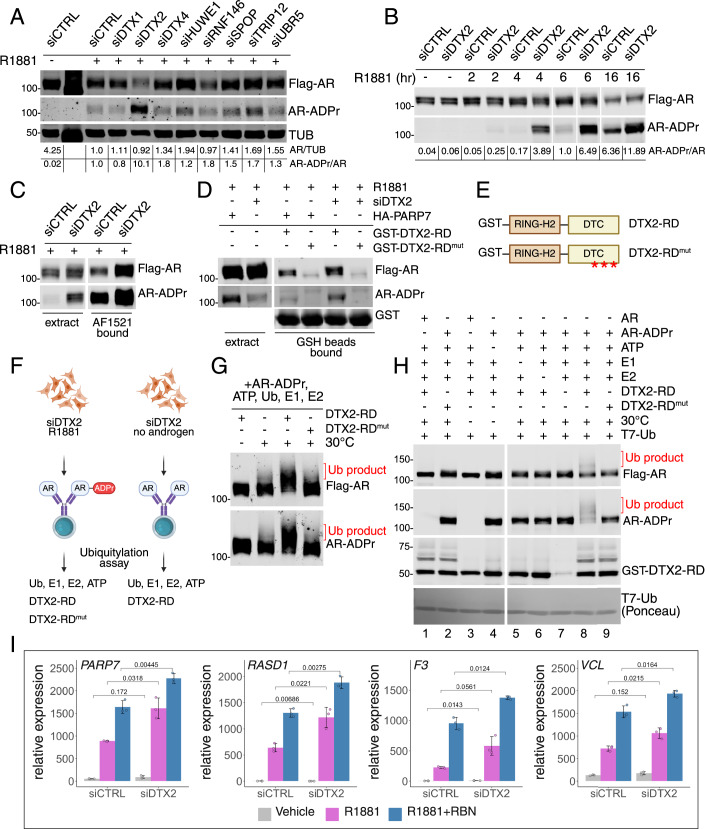
DTX2 is the E3 ligase for ADP-ribosylated AR. (**A**) Immunoblot detection of Flag-AR and AR-ADPr (by FL-AF1521) in PC3-AR cells with siRNA knockdowns (total 4-day knockdown) of the selected relevant E3 ligases (DTX1, DTX2, DTX4, HUWE1, RNF146, SPOP, TRIP12 and UBR5), treated with R1881 for 21 h before cell harvest. The AR/TUB and AR-ADPr/AR ratios for each lane are presented below the blot. (**B**) Immunoblot detection of Flag-AR and AR-ADPr in PC3-AR cells with siDTX2 knockdown, treated with R1881 for the times indicated on the panel. The AR-ADPr/AR ratio for each lane is presented below the blot. (**C**) Immunoblot detection of Flag-AR and AR-ADPr in PC3-AR siCTRL and siDTX2 cell extracts and AF1521 bound fraction. Cell extracts from PC3-AR siCTRL and siDTX2 cells treated with R1881 for 6 h were combined with AF1521 beads for the enrichment of ADP-ribosylated proteins. (**D**) Immunoblot detection of Flag-AR and AR-ADPr in PC3-AR siCTRL/siDTX2 and PC3-AR HA-PARP7 cell extracts and GSH beads bound fraction. Cell extracts from PC3-AR siCTRL/siDTX2 and PC3-AR HA-PARP7 cells treated with R1881 for 6 h were combined with GSH beads loaded with GST-DTX2-RD or GST-DTX2-RD^mut^ for the enrichment of proteins recognized by DTX2 DTC domain. (**E**) Diagrams of DTX2-RD and DTX2-RD^mut^. Three loss-of-function mutations in the DTC domain of DTX2-RD^mut^ (S568A, H582A, and H594A) are indicated with a red asterisk. (**F**) Schematic diagram of AR protein preparation as a substrate for biochemical reactions. Cell extracts from PC3-AR siDTX2 cells treated (left) or untreated (right) with R1881 for 6 h were combined with M2 beads for immunoprecipitation. The purified protein from the preparation with R1881 treatment was used for experiments in (**G**, **H**), and the purified protein from the preparation without R1881 treatment was used only in (**H**). (**G**) Immunoblot detection of Flag-AR and AR-ADPr from the ubiquitylation assay on AR protein prepared with siDTX2 and R1881 treatment (**F**, left). The ubiquitylated products (Ub product, red bracket) are labeled for Flag-AR and AR-ADPr detection. All reactions contained AR-ADPr (R1881-treated samples), ATP, Ub, E1, and E2. For DTX2-RD status (dropout or DTX2-RD^mut^), refer to labels. (**H**) Immunoblot detection of Flag-AR, AR-ADPr, and GST-DTX2-RD from the ubiquitylation assay on AR protein prepared with siDTX2 transfection, and with or without R1881 treatment (**F**). The ubiquitylated products (Ub product) are labeled in red for Flag-AR and AR-ADPr detection. The dropouts of the ubiquitylation assay components (Ub, E1, E2, and 30 °C incubation) are indicated on the labels. The T7-Ubiquitin (T7-Ub) was detected by Ponceau staining. Lane numbers are indicated below the blot. (**I**) Bar plots showing the results of an RT-qPCR experiment in PC3-AR siCTRL/siDTX2 cells, untreated (gray), treated with R1881 (purple), and cotreated with R1881 and RBN2397 (blue). The *y* axis represents the relative expression normalized to the GUS housekeeping gene, and the *x* axis represents the siRNA used. The *P* values from the Welch’s *t* test for comparisons between corresponding conditions in siCTRL and siDTX2 are indicated on the plots. The top of the columns represent the means and the error bars represent standard deviation (*n* = 3; *n* represents number of biological replicates). Source data are available online for this figure.

Multiple Ub E3 ligases can promote AR degradation (An et al, 2014; Li et al, 2014; Lin et al, 2002; Qi et al, 2013; Sarkar et al, 2014), though none require an androgen-induced conformation of AR for ubiquitylation. The dependence of AR degradation on AR ADP ribosylation suggested that cells express an E3 which recognizes an ADP-ribosyl-based degron. To identify an E3 that selectively degrades ADP-ribosylated AR, we focused on E3’s that encode ADP-ribose-binding domains. The Deltex C-terminal domain (DTC) is an ADP-ribose binding module conserved in DELTEX family members (Obiero et al, 2012), which is part of the RING-DTC that recruits ADP-ribosylated substrates for ubiquitylation (Ahmed et al, 2020; Zhu et al, 2022). We included three E3’s that encode the Trp-Trp-Glu (WWE) domain, which can bind poly-ADP-ribose (Aravind, 2001). Also tested were two E3’s that lack ADP-ribose sensing domains but mediate degradation of AR (An et al, 2014) and other steroid hormone receptors (Tsai et al, 2023). We predicted that the depletion of an E3 that selectively degrades ADP-ribosyl-AR would increase the AR-ADPr/AR ratio in cells. To this end, PC3-AR HA-PARP7 cells were transfected with siRNA to the E3’s (Fig. 7A; Appendix Fig. S6A), treated overnight with androgen, and subsequently blotted for ADP-ribosylated and total AR. Depletion of several E3’s resulted in AR-ADPr/AR ratios >1; however, DTX2 depletion generated an AR-ADPr/AR ratio >10 (Fig. 7A). A time course of R1881 treatment in siCTRL and siDTX2 cells displayed a clear accumulation of ADP-ribosylated AR (Fig. 7B). We verified the finding by showing that DTX2 depletion increases the level of ADP-ribosylated AR recovered on AF1521 beads (Fig. 7C). To assess whether DTX2 uses its DTC domain to bind ADP-ribosylated AR, we made use of a DTC mutant with three amino acid substitutions (Zhu et al, 2022) that eliminate ADP-ribose binding (Fig. 7E; Appendix Fig. S6B). With a synthetic ADP-ribosylated AR peptide (Wijngaarden et al, 2023), we used fluorescence polarization to confirm the DTC domain can bind ADP-ribosyl-Cys, that the interaction requires the ADP-ribose moiety, and that binding is lost in the DTC mutant (Appendix Fig. S6C). WT and mutant GST-DTX2 RING-DTC proteins immobilized on GSH beads were used for pull-down assays with extracts from cells depleted of DTX2 and treated with androgen. In the pull-down assay, ADP-ribosylated AR binding to DTX2 was dependent on a functional DTC domain, and the level of binding was increased about twofold by DTX2 depletion (Fig. 7D). Because the ADP-ribose conjugated to AR by PARP7 is also recognized by the MAR antibody (Appendix Fig. S6D), our data suggests the DTC domain of DTX2 recognizes mono-ADP-ribose conjugated to AR. It is also formally possible that the ADP-ribose detected by AF1521 is in the form of a short chain that is too small to cause a significant gel shift.

### DTX2 uses its DTC and RING domains to ubiquitylate AR

To determine if DTX2 can conjugate Ub to ADP-ribosylated AR in vitro, we performed ubiquitylation assays with recombinant enzymes and AR immunoprecipitated (IP’d) from DTX2-depleted cells as the substrate (+ R1881; Fig. 7F). When combined with Ub components, IP’d ADP-ribosylated AR underwent an upward gel shift indicative of ubiquitylation (Ub product) that was dependent on a functional DTC domain in DTX2 (Fig. 7G). Dropout of individual Ub reaction components showed that AR undergoes androgen-, ADP-ribosyl-, and DTX2-dependent Ub modification which is dependent on a functional DTC domain (Fig. 7H; lanes 1, 2, 8, 9). These data suggest that DTX2 regulates AR levels and may contribute to transcriptional output by mediating the turnover of androgen-bound, ADP-ribosylated AR. We tested this prediction in PC3-AR cells by depleting DTX2 and assaying the effect on androgen induction of Module 7 genes. By qPCR, we found that DTX2 depletion increased the androgen- and AR-dependent output of the *PARP7*, *RASD1*, *F3*, and *VCL* genes (Fig. 7I). The magnitude of the effects was less than observed with RBN2397 treatment, reflecting a partial depletion of DTX2 in these cells.

### ADP-ribosyl Cys is the acceptor site for ubiquitylation

**Figure 8:**
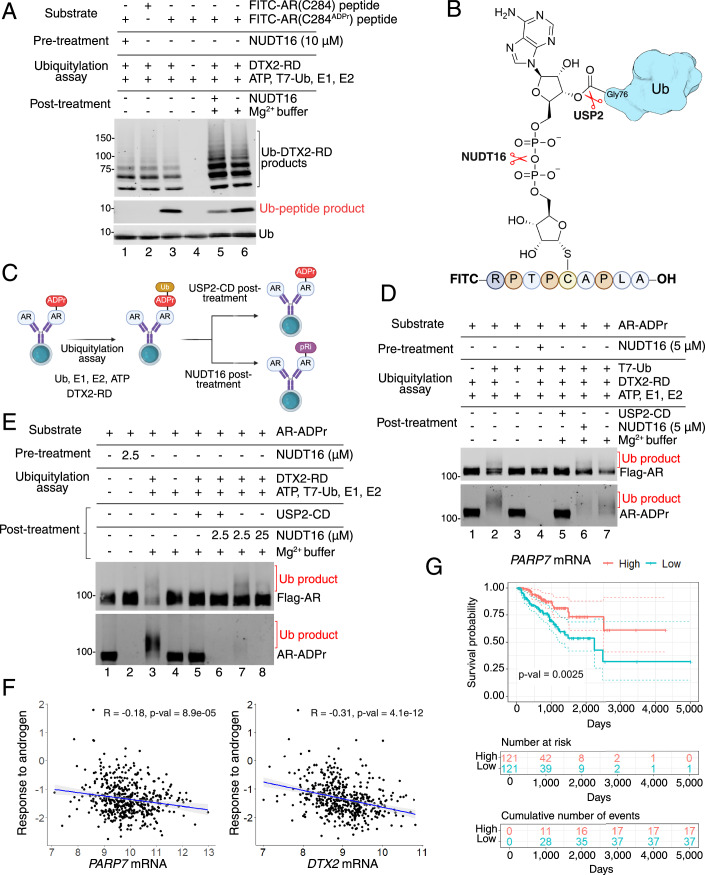
DTX2 conjugates ubiquitin to AR through ADP-ribose. (**A**) Immunoblot detection of Fluorescein (FITC) and T7-Ubiquitin (T7-Ub) from the ubiquitylation assay on FITC-AR(C284) or FITC-AR(C284^ADPr^) peptides. The labels indicate from the top: the substrate used (FITC-AR(C284) or FITC-AR(C284^ADPr^) peptides), pre-ubiquitylation assay treatments (NUDT16), the ubiquitylation assay (all reactions contained ATP, T7-Ub, E1 and E2, for DTX2-RD dropout, refer to labels), and the post- ubiquitylation assay treatments (NUDT16 or Mg^2+^ buffer). Lane numbers are indicated below the blot. (**B**) Schematic diagram representing FITC-AR(C284^ADPr^) peptide conjugated to ubiquitin (Ub). Indicated with red scissors are bonds within the ADP-ribose structure cleaved by NUDT16 and USP2. (**C**) Schematic of the ubiquitylation assay workflow for (**D**, **E**). (**D**) Immunoblot detection of Flag-AR and AR-ADPr (by FL-AF1521) from the ubiquitylation assay on AR protein prepared with siDTX2 transfection and R1881 treatment (refer to Fig. 6F for sample preparation workflow). The ubiquitylated products (Ub product) are labeled in red for Flag-AR and AR-ADPr detection. The labels separated by black lines indicate sequential steps from top to bottom. From the top, the labels indicate the substrate used (AR-ADPr), pre-ubiquitylation assay treatments (NUDT16), the ubiquitylation assay, and the post-ubiquitylation assay treatments (USP2-CD, NUDT16, or Mg^2+^ buffer). Lane numbers are indicated below the blot. (**E**) Immunoblot detection of Flag-AR and AR-ADPr from the ubiquitylation assay on AR protein prepared with siDTX2 and R1881 treatment (refer to Fig. 6F for sample preparation workflow). The ubiquitylated products (Ub product) are labeled in red for Flag-AR and AR-ADPr detection. The labels separated by black lines indicate sequential steps from top to bottom. From the top, the labels indicate the substrate used (AR-ADPr), pre-ubiquitylation assay treatments (NUDT16), the ubiquitylation assay, and the post-ubiquitylation assay treatments (USP2-CD, NUDT16, or Mg^2+^ buffer). Lane numbers are indicated below the blot. (**F**) Scatter plots depicting the correlation between PARP7 (left) or DTX2 (right) mRNA expression and the response to androgen pathway activity calculated by PARADIGM in primary prostate cancer patients from TCGA-PRAD cohort. Each dot represents one patient (*n* = 478, *n* represents the number of patients). Pearson correlation coefficients and corresponding *P* values are indicated on the plots. (**G**) Kaplan–Meier plot depicting progression-free interval (PFI) in primary prostate cancer patients from the TCGA-PRAD cohort, stratified by PARP7 expression levels. The red line represents patients with high PARP7 expression (top 25%), and the green line represents patients with low PARP7 expression (bottom 25%). The *x* axis represents time (days), and the *y* axis represents the progression-free interval probability. The interval distributions were compared using the log-rank test, with the *P* value indicating statistical significance. Dotted lines represent the 95% confidence interval. The *P* values were calculated using the log-rank test. Source data are available online for this figure.

Biochemical approaches have been used by other groups to show that DTX family E3s can ubiquitylate substrates modified with ADP-ribose on serine (Ahmed et al, 2020) and acidic amino acids (Zhu et al, 2022). To test if DTX2 can transfer Ub to ADP-ribosyl-Cys, we used a synthetic ADP-ribosylated AR peptide as a model substrate, which lacks lysine and a free amino terminus and cannot undergo canonical ubiquitylation (Fig. 8A,B). Ub conjugation to the AR peptide was found to be strictly dependent on the ADP-ribosyl-Cys moiety since no product was generated with an unmodified peptide or if the ADP-ribosyl peptide was pretreated with the phosphodiesterase NUDT16 which cleaves ADP-ribose (lanes 1–4; Fig. 8A,B; Appendix Fig. S7A). Post-treatment with NUDT16 reduced the amount of Ub-peptide conjugate, consistent with Ub attachment via ADP-ribose (lanes 3, 5, 6; Fig. 8A). These data show that DTX2 conjugates Ub to ADP-ribosyl-Cys, and the substrate requires neither a lysine nor a free N-terminus.

To further characterize the Ub linkage to ADP-ribosylated AR, we performed ubiquitylation assays with cell-derived ADP-ribosylated AR prepared by IP (see Fig. 7F). We included pre- and post-treatment with NUDT16, and post-treatment with the deubiquitylating enzyme USP2, which cleaves the Ub carboxyl terminus (Fig. 8B,C). A high molecular weight smear (Ub product) is detected when probing for AR and for ADPr; these products correspond to ubiquitylated AR since neither is detected with Ub dropout (lanes 1, 2; Fig. 8D). The Ub product was eliminated by pretreatment with NUDT16 and by post-treatment with USP2 (lanes 4, 5; Fig. 8D). The Ub product displayed a slight resistance to post-treatment with NUDT16, as a smear was still detectable upon probing for AR and ADPr (lane 6; Fig. 8D). NUDT16 resistance could be indicative of Ub transfer to a proximal lysine (Appendix Fig. S7B). The cleavage resistance of the Ub product was, however, overcome by increasing the NUDT16 concentration, temperature, and incubation time (lanes 3, 7, 8; Fig. 8E). These data are consistent with Ub conjugation to AR through an ADP-ribose linkage. Given how ADP-ribose sits in the binding pocket of NUDT16 (Thirawatananond et al, 2019), we speculate that Ub conjugated to the adenine-proximal ribose might disfavor NUDT16 binding and partially protect the ADPr linkage from hydrolysis prior to engagement with DTX2. The proximal ribose ring in ADP-ribose is predicted to be the site of Ub attachment since, by modeling, binding to the DTC domain would place it in close proximity to the carboxyl terminus of Ub in the Ub-E2 complex (Zhu et al, 2022). While our data shows that Ub is conjugated through an ADP-ribose linkage, it remains possible that lysines proximal to an ADP-ribosylation site undergo DTX2-dependent Ub conjugation, and are not detected by the assay.

### PARP7 and DTX2 are negatively correlated with androgen signaling in prostate cancer

Our functional analyses in multiple preclinical models show that PARP7 and DTX2 are components of a negative feedback mechanism that helps limit androgen signaling through AR. To determine whether the expression of PARP7 and DTX2 affect androgen signaling in human prostate tumors, we focused on the data generated from prostate primary tumors in The Cancer Genome Atlas (TCGA-PRAD). We downloaded the batch effect-normalized mRNA data and the pathway activity scores generated using PARADIGM from UCSC Xena (Goldman et al, 2020; Vaske et al, 2010) and queried the correlation between PARP7 and DTX2 mRNA expression and the “response to androgen” pathway activity. We found a weak negative correlation for TIPARP (*R* = −0.18) and a moderate negative correlation for DTX2 (*R* = −0.31) (Fig. 8F), showing that higher levels of these components are associated with a reduction in AR output. Using the log-rank test, we found significant differences in progression-free survival between patients stratified based on PARP7 gene expression in the TCGA-PRAD cohort (Fig. 8G). The effect of PARP7 expression on progression-free survival remains significant (HR = 0.56) even when we account for age of diagnosis in a multivariate Cox proportional hazard model. Our analysis shows that PARP7 expression is prognostic, and high TIPARP expression is associated with a better outcome (Fig. 8G). These conclusions fit our biochemical data and suggest PARP7 levels are correlated with better clinical outcomes through modulation of androgen signaling.

## Discussion

We identified a post-transcriptional mechanism that regulates AR protein levels and gene expression in prostate cancer cells. The mechanism is based on androgen induction of PARP7, ADP-ribose writing by PARP7, ADP-ribose reading and ubiquitylation by DTX2, and degradation by the proteasome (Appendix Fig. S7B). The mechanism results in post-transcriptional reduction of AR protein levels and is the basis of a PARP7-dependent negative feedback loop that controls the expression of specific modules of AR target genes. The data supporting these conclusions were derived from molecular, biochemical, and in silico approaches, which, taken together, provide new insight into how androgen signaling through AR can be regulated and generate temporal effects on gene expression. Prostate cancer cells therefore use three independent mechanisms to control AR levels: LSD1-mediated transcriptional repression of AR (Cai et al, 2011), E3-mediated degradation of ligand-free AR (An et al, 2014; Li et al, 2014; Lin et al, 2002; Qi et al, 2013; Sarkar et al, 2014), and ADP-ribosylation-dependent degradation of androgen-bound AR in association with chromatin. Importantly, the mechanism driven by androgen signaling limits degradation to AR that has fulfilled its transcription function.

One of the challenges for understanding PARP-dependent pathways is determining how family members locate and mediate substrate-specific ADP ribosylation. Through mathematical modeling of the AR-PARP7 pathway and associated transcriptional outputs, we were able to infer that ADP ribosylation occurs when AR is bound to DNA. We tested this hypothesis using a mutant form of AR that encodes an amino acid substitution in the DNA recognition helix (V582F) that results in loss-of-function and complete androgen insensitivity syndrome (Lumbroso et al, 1993). Notably, the disease mutant AR binds androgen with nanomolar affinity, undergoes nuclear translocation, and conformation-dependent AF1 domain phosphorylation (Black et al, 2004; Lobaccaro et al, 1996) but it fails to undergo ADP ribosylation by PARP7. As a consequence, the AR V582F mutant is not degraded in response to androgen treatment. The DNA-binding requirement for AR ADP ribosylation is also consistent with our observation that a subset of PARP7 is chromatin-associated and undergoes trapping in the nuclear fraction of cells treated with the catalytic inhibitor RBN2397 (Gozgit et al, 2021; Yang et al, 2023).

Although serine is a common ADP-ribose acceptor site for PARPs, our mass spectrometry data and PARP7 inhibitor experiments indicate that AR is ADP-ribosylated on cysteines. Indeed, PARP7 shows a bias for ADP ribosylation of Cys residues (Rodriguez et al, 2021; Yang et al, 2021). Our analysis of the sites responsible for ADP-ribosylation-dependent AR degradation led us to Cys620, one of three ADP-ribosylation sites in the AR DBD (Yang et al, 2021). Using previously solved crystal structures of the AR DBD bound to DNA (Lee et al, 2024; Shaffer et al, 2004) and energy minimization approaches, we found that the solvent accessibility of Cys620 increases upon DNA binding. While these data are consistent with Cys620 modification controlling AR degradation, other Cys sites in the AR DBD could also perform this function. Restricting the formation of an ADP-ribosyl-Cys degron to chromatin-bound AR could couple the degradation mechanism to AR that has homo-dimerized on DNA and fulfilled its transcription function.

The usefulness of inactivating ADP-ribose acceptor sites in the DBD by mutation is limited by the fact that AR ADP ribosylation requires DNA binding. Using an alternative strategy, we found that the WT DBD was capable of mediating androgen-induced degradation of a VP16-DBD-LBD reporter protein. These data lend support for our model that AR DBD modification by PARP7 in the context of chromatin is a key step for the degradation mechanism. PARP7 ADP-ribosylates cysteines in the RNA-binding zinc fingers of PARP13 (Rodriguez et al, 2021), raising the possibility that substrate recognition by PARP7 might include features associated with zinc binding loops.

Although PARP1 has been studied as an enzyme that can undergo auto-modification on multiple sites, to our knowledge AR is the first substrate shown to undergo extensive, multi-site mono-ADP ribosylation. Following the induction of the PARP7 gene, AR is ADP-ribosylated on a total of 11 sites, all of which are cysteines. Most of the ADP-ribosylation sites map to the unstructured NTD, which raises the question of how androgen binding to AR controls PARP7 engagement with sites that span a ~400 amino acid unstructured region. Given the striking dependence of AR ADP ribosylation on DNA binding, it appears that ADP ribosylation of the NTD, like the DBD, occurs when AR is chromatin-bound. The fact that point mutations in the PARP7 zinc finger eliminate AR ADP ribosylation (Kamata et al, 2021b) would be consistent with a chromatin targeting function for this domain. Chromatin could provide the context for PARP7 encounters with other transcription factors as well (Bindesboll et al, 2016; Rasmussen et al, 2021), though this remains to be tested.

Our previous work, together with the current study, is beginning to shed light on the functional significance of multi-site ADP ribosylation of AR and particularly the critical roles of reader proteins. ADP-ribosylated AR can be recognized by two different ADP-ribose readers. ADP-ribose on AR is read by the tandem macrodomains in the PARP9/DTX3L complex (Yang et al, 2021). Oligomeric assembly of PARP9/DTX3L, which would place at least six macrodomains in the complex, is required for efficient binding to ADP-ribosylated AR (Vela-Rodriguez et al, 2024). In this study, we show the DTC domain of DTX2 also can read ADP-ribose conjugated to AR and that the reader function is obligatory for DTX2-dependent Ub conjugation to AR since these interactions are lost upon mutation of the ADP-ribose binding pocket of the DTC domain.

Our mathematical modeling and biochemical data support the conclusion that PARP7 modifies AR on chromatin, which links AR transcriptional activity to AR protein degradation. This mechanism offers an efficient form of negative feedback in which agonist-bound nuclear AR can be degraded after engaging in transcriptional regulation. In doing so, it ensures that androgen signaling has productive effects on transcription prior to initiation of degradation. Although the relationship between signal duration and transcriptional programs has been characterized in detail for steroid receptors, the duration of signaling cascades can shape differential transcriptional outputs in multiple systems (Voss and Hager, 2014). Our findings link PARP7 and androgen-induced transcription to AR degradation. If this mechanism operates through degradation of transcriptionally engaged AR, then the initial abundance of AR can influence the timing of signal termination. This correlation offers the cell a PARP7-dependent mechanism for tuning AR levels to control the duration of androgen signaling and, as a result, elicit distinct transcriptional programs in different contexts.

It is interesting to note that although DTX3L contains a DTC domain, prostate cancer cells lacking DTX3L still undergo androgen-induced AR degradation. This suggests that DTC domains, ADP-ribosylated substrates, or both, have features that impart selectivity for ADP-ribose reading that leads to degradation. This consideration seems relevant to other reader-substrate interactions as well, given the large number of ADP-ribosylated proteins in the cell. PARP9/DTX3L binds to ADP-ribosylated AR through tandem macrodomains in PARP9, and in this setting, the DTC domain of DTX3L could be available to read and modify ADP-ribosyl-substrates in chromatin proximal to AR. Since DTX3L is required for p53 K48 Ub conjugation at DNA damage sites (Yan et al, 2023), it may have the capacity for both lysine-dependent and lysine-independent Ub conjugation, the latter utilizing a DTC domain.

Castrate-resistant prostate cancer (CRPC) remains a major clinical challenge. At the molecular level, CRPC generally relies on AR activity supported by intra-tumoral androgen synthesis, AR gene alterations to affect its sensitivity to ligands, and expression of therapy-resistant spliced forms such as AR-V7 that lack a ligand-binding domain. Our work shows that in cells, PARP7, in effect, restrains AR function by initiating its degradation, and analysis of patient data suggests the mechanism may be relevant to clinical outcomes. In a previous study, we showed that PARP7 expression is lower in metastatic tumors compared to primary tumors, and the difference is greater for AR+ metastatic tumors (Yang et al, 2021). Here, we show that within a cohort of patients, high PARP7 and high DTX2 expression in primary tumors are each negatively correlated with androgen signaling. Thus, high PARP7 could provide a survival benefit by limiting androgen signaling through a reduction of AR protein level. Interestingly, this benefit is conceptually analogous to androgen deprivation therapy used clinically to reduce AR activity.

There is an emerging view that ADP-ribose writing, reading, and lysine-independent ubiquitylation are integrated as a protein degradation mechanism. Substrate engagement with a particular PARP enzyme and site-specific ADP ribosylation act as substrate priming events that create an ADP-ribose degron. Ub conjugation then relies on selective recognition of ADP-ribose by an E3 ligase that contains a reader domain. In terms of the detailed mechanism, it was shown that DELTEX family E3 ligases conjugate the Gly76 carboxyl terminus of Ub to an oxygen atom in the adenine-proximal ribose (Zhu et al, 2022). In the case of androgen-induced degradation of AR, mutation of a single ADP-ribosylation site (Cys620) protects AR protein from the unconventional degradation, though as discussed other ADP-ribosylation sites in the DBD could mediate the same outcome. DTX2, the E3 ligase responsible for the degradation of ADP-ribosyl-AR, also senses and degrades DNA repair and chromatin-associated factors that, based on drug sensitivity, are ADP-ribosylated by PARP1 (Ahmed et al, 2020). ADP-ribosyl-dependent degradation is expanding the conceptual framework for cellular functions mediated by the PARP family, and it raises the question of whether PARP inhibitor effects on protein homeostasis might evolve as a therapeutic consideration. Understanding ADP-ribosyl-dependent degradation mechanisms could complement the current rationale for using PARP1/2 inhibitors that are based on synthetic lethality associated with defective DNA repair.

## Methods

**Table:** Reagents and tools table

Reagents/resources	Reference or source	Identifier or catalog number
FITC-AR(C290-ADPr) peptide	Wijngaarden et al (2023)	N/A
FITC-AR(C290) peptide	Wijngaarden et al (2023)	N/A
R1881	Perkin-Elmer	Cat#NLP005005MG
Dihydrotestosterone (DHT)	Sigma-Aldrich	Cat#D-073
RBN2397	DC Chemicals	Cat#DC31069
RBN012759	MedChemExpress	Cat#HY-136979
Velaparib	Selleck Chemical LLC	Cat#S100410MG
Olaparib	MedChemExpress	Cat#HY-10162
Talazoparib	MedChemExpress	Cat#HY-16106
Bortezomib	MedChemExpress	Cat#HY-10227
ViaFect transfection reagent	Promega	Cat#E498A
Lenti-X Concentrator	Takara	Cat#631232
Lipofectamin RNAiMAX transfection reagent	Invitrogen	Cat#13778-075
DNase	Roche	Cat#04716728001
HotStart™ 2X Green qPCR Master Mix	APExBIO	Cat#K1070
QIAshredder kit	Qiagen	Cat#79656
RNeasy kit	Qiagen	Cat#74104
iScript cDNA synthesis kit	Bio-Rad	Cat#1708890
QIAquick Gel Extraction kit	Qiagen	Cat#28704
ProLong Gold antifade reagent	Invitrogen	Cat#P36934
Anti-Flag M2 magnetic beads	Sigma	M8823
Glutathione MagBeads	GenScript	L00895-10
**Oligonucleotides, siRNAs, and recombinant plasmid DNAs**		
PARP7 exon 3 gRNA (5’-TGGATGGAGAGAGTATCCCG-3’)	This study	N/A
siCTRL	Ambion	AM-4635
siDTX1	ThermoFisherScientific	s4356
siDTX2	ThermoFisherScientific	s223231
siDTX4	ThermoFisherScientific	s23321
siHUWE1	ThermoFisherScientific	s19596
siRNF146	ThermoFisherScientific	s37822
siSPOP	ThermoFisherScientific	s15955
siTRIP12	ThermoFisherScientific	s17810
siUBR5	ThermoFisherScientific	s28025
GST-eAF1521	M. Hottiger lab (Nowak et al, 2020)	N/A
pGEX-4T-2: DTX2-RD and mutant	This study	N/A
pLhyg-Flag-AR and mutants	This study and Yang et al (2021)	N/A
pLhyg-Flag-VP16-AR-DBD-LBD(C670G)	This study	N/A
pDEST- Flag-HA-USP2	Addgene	cat #22577
pET28C-His-USP2-CD	This study	N/A
**Antibodies**		
HA tag	Covance	Cat#A488-101L; RRID:AB_10063991
Ub	ProteinTech	Cat#80992-1-RR; RRID:AB_2923694
T7 tag	EMD Millipore	Cat#69522; RRID:AB_11211744
DTX2	ProteinTech	Cat#67209-1-Ig; RRID:AB_2882502
Mono-ADP-ribose	BIO-RAD	Cat#TZA020
TUBULIN	Sigma-Aldrich	Cat#T9028; RRID:AB_261811
AR (1–21 amino acids)	Yang et al (2021)	N/A
AR (659–667 amino acids)	Yang et al (2021)	N/A
M2	Sigma-Aldrich	F3165
PARP7 (119–132 amino acids)	Yang et al (2021)	N/A
GST	T. Parsons’ Lab	mAb 9D9-F3-F7 hybridoma
FL-AF1521	Kamata et al (2019)	N/A
Donkey anti-human IgG(H + L) cross-absorbed secondary antibody DyLight 800	Invitrogen	Cat#SA5-10132;
		RRID:AB_2556712
Alexa Fluor 680 donkey anti-rabbit IgG(H + L)	Invitrogen	Cat#A10043;
		RRID:AB_2534018
Alexa Fluor 680 donkey anti-mouse IgG(H + L)	Invitrogen	Cat#A10038;
		RRID:AB_11180593
Anti-mouse IgG(H&L) (Goat) antibody DyLight 800 conjugated	Rockland	Cat#610-145-002;
		RRID:AB_10703265
Fluorescein (FITC) Polyclonal Antibody, DyLight™ 680	Rockland	Cat# 600-144-096; RRID:AB_1661059
**Bacterial strain and recombinant proteins**		
BL21(DE3)pLysS	EMD Millipore (Novagen)	Cat#69451
GST-AF1521^tandem^	Kamata et al (2019)	N/A
GST-eAF1521	This study	N/A
GST-DTX2-RD	This study	N/A
GST-DTX2-RD^mut^	This study	N/A
T7-Ub	Yang et al (2017)	N/A
Bovine Ub	Sigma	Cat#U6253
E1 Ube1	R&D Systems	Cat#E-304
His-UbcH5C	R&D Systems	Cat#E2-627
NUDT16	In-Kwon Kim Lab	N/A
USP2-CD	This study	N/A
**Experimental models: cell lines**		
293T	ATCC	Cat#CRL-3216;
		RRID:CVCL_0063
PC3-AR	Yang et al (2021)	RRID:CVCL_C3HH
PC3-AR(shCTRL or shPARP7)	Yang et al (2021)	N/A
PC3-AR(HA-PARP7)	Yang et al (2021)	N/A
PC3-AR(VP16-AR-DBD-LBD/C670G)	This study	N/A
VCaP	ATCC	Cat#CRL-2876;
		RRID:CVCL_2235
PC3m	ATCC	RRID:CVCL_9555
PC3m-HA-PARP7	Yang et al (2021)	N/A
PC3m-HA-PARP7-Flag-AR(WT and mutants)	This study and Yang et al (2021)	N/A
**Critical commercial assays**		
Deposited Data		
RNA-seq 8 h R1881	GEO database	GSE148397
RNA-seq 12 h R1881	GEO database	GSE136272
RNA-seq 18 h R1881/RBN2397	GEO database	GSE272500
RNA-seq 22 h R1881	GEO database	GSE148397
RNA-seq 24 h R1881 #1	GEO database	GSE133876
RNA-seq 24 h R1881 #2	GEO database	GSE120660
RNA-seq 48 h R1881	GEO database	GSE135879
ChIP-seq	GEO database	GSE84432
**Software and algorithms**		
R-4.2.2	http://www.r-project.org/	RRID:SCR_001905
RStudio	https://posit.co/	RRID:SCR_000432
Fiji	http://fiji.sc/	RRID:SCR_002285
kallisto	https://pachterlab.github.io/kallisto/about	RRID:SCR_016582
tximport	https://github.com/mikelove/tximport	RRID:SCR_016752
DESeq2	https://bioconductor.org/packages/release/bioc/html/DESeq2.html	RRID:SCR_015687
EnhancedVolcano	https://bioconductor.org/packages/EnhancedVolcano/	RRID:SCR_018931
eulerr	https://cran.r-project.org/package=eulerr	RRID:SCR_022753
pheatmap	https://www.rdocumentation.org/packages/pheatmap/versions/0.2/topics/pheatmap	RRID:SCR_016418
Cistrome	http://cistrome.org/	RRID:SCR_000242
fgsea	https://bioconductor.org/packages/fgsea/	RRID:SCR_020938
ComBat	http://www.bu.edu/jlab/wp-assets/ComBat/Abstract.html	RRID:SCR_010974
WGCNA	http://www.genetics.ucla.edu/labs/horvath/CoexpressionNetwork/	RRID:SCR_003302
tidyverse	https://cran.r-project.org/package=tidyverse	RRID:SCR_019186
survival	https://cran.r-project.org/package=survival	RRID:SCR_021137
survminer	https://rdocumentation.org/packages/survminer/versions/0.4.9	RRID:SCR_021094
CREST	https://github.com/grimme-lab/xtb/releases	N/A

### Cell culture

HEK293T (ATCC CRL-3216) was maintained in DMEM, 5% fetal bovine serum (FBS), and 1% Pen/Strep (P/S). PC3 (ATCC CRL-1435), PC3M (CVCL_9555), and derivative cell lines were grown in RPMI1640, 5% FBS, and 1% P/S. VCaP (ATCC CRL-2876) was grown in DMEM/F12, 10% FBS, and 1% P/S. All cells were grown in the presence of 5% CO_2_ and at 37 °C. For RNA-seq and qPCR experiments, cell growth media were switched to phenol red-free medium and FBS depleted of androgens 24 h before the drug treatments. HEK293T are female and all other cell lines used in this study are male.

### Stable cell line generation

A 60 mm-plate of HEK293T cells (60-70% confluency) were transfected with 1.5 μg of a lentiviral plasmid DNA carrying target gene and 0.75 μg each of two accessory plasmids pMD2g and psPAX2 using ViaFect transfection reagent (Promega E498A, total DNA: ViaFect ratio = 1 μg: 3 μl) and fresh medium for 16 h, replenished with a high FBS growth medium (DMEM, 35% fetal bovine serum and 1% P/S) and grow for another 24 h. The lentivirus-containing medium was moved to a 10 ml-conical tube which was centrifuged (700 × *g*) to remove the cell debris. The supernatant was passed through a 0.45 μm-EZFlow Syringe Filter, concentrated using Lenti-X Concentrator (Takara, 631232) and then transfected into a target cell line with 8 μg/ml of polybrene in the growth medium for 24 h. After cells grow in the growth medium for 2–3 duplication time, cells were started selection with 1–2 μg/ml of puromycin or 0.2 mg/ml of hygromycin in the growth medium according to its respective antibiotic selection marker on the plasmid DNA. For shRNA knockdown cell lines following shRNAs were used: shGFP (shCTRL): CCGGTACAACAGCCACAACGTCTATCTCGAGATAGACGTTGTGGCTGTTGTATTTTT, and shPARP7:CCGGGAAGGCAAGCTACTCTCATAACTCGAGTTATGAGAGTAGCTTGCCTTCTTTTT. The PARP7 knockdown efficiency with this shRNA is 50–70%.

### siRNA transfection

Cells were transfected with 20 nM of siRNA using Lipofectamin RNAiMAX transfection reagent (Invitrogen 13778-075) and passaged when they reach confluency. Cells were grown for 4 days in total, and drug treatments were done before cell harvest.

### RNA-seq

For RNA-sequencing, VCaP cells were plated in the phenol-free DMEM media supplemented with 10% charcoal-stripped (depleted of androgens) FBS and treated with R1881 (2 nM), RBN2397 (100 nM), and R1881 + RBN2397 and corresponding vehicles (ethanol for R1881 and DMSO for RBN2397) for 18 h in biological triplicate (distinct samples). RNA was extracted using a Qiagen RNeasy kit. Library preparation and sequencing were performed by Hudson Alpha. In brief, a fluorometric assay was used to assess the RNA concentration and integrity; the standard polyA method was used to make indexed libraries; size and concentration were determined using quality control, and samples were sequenced using Illumina HiSeq 2500 at a depth of 250 million × 50-bp paired-end reads. Reads were pseudoaligned to the hg38 genome (ENSEMBL GRCh38.89) using kallisto (Bray et al, 2016). The transcript-level abundance estimates were summarized to gene-level counts using tximport R package (Soneson et al, 2015). The DESeq2 R package (Love et al, 2014) was used to apply Variance-stabilized transformation (VST) to counts data and to determine differentially expressed genes between conditions. Genes with less than 20 counts in all conditions were eliminated (pre-filtering). For all downstream analyses, differentially expressed genes (DEGs) were additionally filtered (baseMean >100). Volcano plots were generated using the EnhancedVolcano R package (Blighe et al, 2023). Venn diagrams were generated using the eulerr R package (Larsson, 2024) on filtered genes (*P*-adj <0.001). All heatmaps were generated using the pheatmap R package (Kolde, 2019) with VST counts.

### AR targets

CistromeGO (http://go.cistrome.org/) online tool was used to create a set of 1000 AR target genes, allowing the integration of ChIP-seq and RNA-seq data. A VCaP ChIP-seq peak bed file was taken from a publicly available dataset (GSE84432, 4 h of R1881 treatment) (Data ref: Toropainen et al, 2016) and the differential expression file (DESeq2) was also generated from a publicly available dataset (GSE148397, 8 h of R1881 treatment) (Data ref: Nevedomskaya et al, 2020). Analysis was conducted with a half-decay distance of 10 kb. The top 1000 genes from the resulting analysis were used to create a list of AR targets.

### Pathway enrichment

All gene set enrichment analyses (GSEA) were performed using the fgsea package (preprint: Korotkevich et al, 2021) with nPermSimple = 500,000 and log_2_FoldChange as a gene-level statistic. Gene sets for GSEA in Fig. 1C were created by filtering the R1881 vs. CTRL DEGs (*P*-adj <0.001, log_2_FoldChange > 0 for increased gene expression, log_2_FoldChange < 0 for decreased gene expression). To create a gene set for Appendix Fig. S1B, our previously published RNA-seq dataset from VCaP cells +/− R1881 and +/− DTX3L knockdown (KD) (GSE133876) (Data Ref: Nyquist et al, 2019) was used. DTX3L KD vs. CTRL DEGs were eliminated from R1881 + DTX3L KD vs. R1881 DEGs (baseMean >100, *P*-adj <0.001) to enrich specifically for DTX3L-regulated genes that are induced by R1881. The gene set was created from the gene names of the remaining DEGs. Overrepresentation analysis (ORA) was performed using the fgsea R package. The expected frequency was calculated by dividing the gene set (module) size by the background set size (24,824 genes) and multiplied by the study set (AR targets) size ((module size/24,824) × 1000). To calculate the fold enrichment, the overlap between each module and AR targets was divided by the expected frequency.

### Compiling RNA-seq datasets

To create the compiled RNA-seq androgen treatment time course, the following publicly available RNA-seq datasets from VCaP cells were used: 8 h—GSE148397 (*n* = 10, Data ref: Nevedomskaya et al, 2020), 12 h—GSE136272 (*n* = 3, Data ref: Brzezinka et al, 2020), 18 h—GSE272500 (*n* = 3, Data ref: Wierbilowicz et al, 2025), 22 h—GSE148397 (*n* = 9, Data ref: Nevedomskaya et al, 2020), 24 h—GSE133876 (*n* = 3, Data ref: Nyquist et al, 2019) and GSE120660 (*n* = 3, Data ref: Jividen et al, 2018) and 48 h—GSE135879 (*n* = 2, Data ref: Nyquist et al, 2019). One untreated control from each study (*n* = 7) was used as 0-h. All datasets were uniformly reprocessed. FASTQ reads were pseudoaligned to the hg38 genome (ENSEMBL GRCh38.89) using kallisto. The transcript-level abundance estimates were summarized to gene-level counts using tximport R package. The resulting counts matrix was next batch-corrected using the ComBat R package (Johnson et al, 2007).

### Weighted gene co-expression network analyses

For weighted gene co-expression network analyses (WGCNA), the WGCNA R package (Langfelder and Horvath, 2008) was used. The compiled counts matrix was pre-filtered to keep genes with 15 or more counts in at least 75% of all samples (30) or 75 or more counts in at least one sample. Next, the VST was applied to normalize the data. The soft power (β value) of 14 was selected based on the appearance of diagnostic plots (scale independence and mean connectivity). The blockwiseModules function was used to create the signed network, with Pearson correlation as a measure of similarity between genes, and to identify modules using hierarchical clustering on a signed topological overlap matrix. The max block size was set to 14,000 to process all the genes (13,811) in one block. Similar modules were merged based on their module eigengene, using a dendrogram cut height of 0.25, resulting in 32 modules. Modules with less than 100 genes were excluded, leaving 19 modules, from which a GMT file for GSEA and ORA was created.

### Tumor analysis

The TCGA-PRAD (The Cancer Genome Atlas—Prostate Adenocarcinoma) cohort was used for the patient data analysis. Log_2_ transformed expression data, PARADIGM pathway activity, and survival data were downloaded from Xena (Goldman et al, 2020). Scatter plots and Pearson correlation were generated with ggpubr R package (Kassambara, 2023). The multivariate Cox model was built with progression-free interval (PFI) data using the survival R package (Therneau, 2024). The Kaplan–Meier (KM) plot was generated with the survminer R package (Kassambara et al, 2024).

### Mathematical modeling

The complete equations for the models are provided (Appendix Document 1).

Assumptions made: (1) All AR is considered to be ligand-bound. (2) AR degradation in the absence of ADP ribosylation is so slow as to be disregarded. (3) PARP7 has two degradation rates, a fast and a slow rate. AR promotes the slow rate. (4) For Model 1, AR can only be ADP-ribosylated when bound to a promoter. (5) For Model 2, AR is ADP-ribosylated when not bound to a promoter. Pre-processing experimental datasets: Simulations were scored on their ability to predict the dynamics of the AR-responsive transcripts PARP7, F3, and ERRFI1. Since PARP7, F3, and ERRFI transcripts have similar expression levels throughout the experiments, we averaged them and used that as a composite transcriptional profile for the model (referred to as PA7F3ER1 in the model). Additional time points in the transcriptional response were interpolated to score the simulations. The experimental datasets were normalized to have a maximum value of 100. Simulations: Modeling was performed in MATLAB (version R2023b) using the ODE solver ode23s to solve the ODE system of mass-action equations (see Appendix Table S1). The ODE solver runs 2200 times with 0 to 0.01 integration, and each 100 runs represents an hour worth of transcript expression. A second model was built to capture the PARP7 inhibition by RBN2397. The two models run simultaneously sharing the same parameters and initial concentrations, but with the rate constant for ADP ribosylation set to 0 for the inhibitor model. The two models output separate SSE scores which are then summed to assess the ability of the model to fit both + and − drug conditions. Initial Concentrations: All transcripts/proteins were initiated with zeros except AR and promoters. The number of AR-responsive promoters in a cell has been found to be 3050 and the number of AR molecules in the cells has been found to be 20,000, which leads to a ratio of 6.6 AR per promoter (Bruchovsky et al, 1975; Massie et al, 2011). We maintained this ratio and found that a condition of 660 AR and 100 promoters was effective for performing the simulations. Parameter Estimation: We divided the parameter estimation into two steps; initial parameters were determined by Monte Carlo selecting from a uniformly distributed parameters, followed by refinement using a Monte Carlo Markov Chain selecting from normally distributed parameters (Valderrama-Bahamondez and Fröhlich, 2019). We first ran 100,000 Monte Carlo simulations. The parameter sets were ranked from the lowest to highest SSE scoring, and we then chose the top 100 parameter sets. The last step is Monte Carlo Markov Chain method, more specifically Metropolis-Hastings algorithm (Valderrama-Bahamóndez and Fröhlich, 2019). Each parameter set runs 5000 times to explore its close surroundings and find the optimum parameter set. To ensure each worker does not generate overlapping random numbers, we initialized rng(), a random number generator, with a unique seed. The seed was calculated by multiplying the current second, using clock built-in function, by 1000 and rounding down to an integer. We add the number of iterations to the seed to ensure different seeds if workers are initiated simultaneously. The top-scoring parameter set for each model are shown in Appendix Table S2. To select the best model, we calculated the Bayesian Information Criterion (BIC) (Wagenmakers and Farrell, 2004). BIC is used as a method to assess the models using a maximum likelihood function (Leung, 2022). The model with the lowest BIC is more likely correct. Bayes weights were calculated to provide a probabilistic measure (a normalized score between 0 and 1) of the competing models. The highest Bayes weight indicates the best model among the considered models. BIC and Bayes weights for each model are shown in Appendix Table S3.

### Protein modeling

We calculated the solvent-accessible surface area (SASA) for three sets of cysteine residues (Cys596, Cys602, and Cys620), as well as the total SASA for three different PDB structures: two human entries (8RM6 and 8RM7) and a rat entry (1R4I). As the PDB structure originates from Rattus norvegicus, while our experimental system pertains to Homo sapiens, there is a consistent shift of 18 amino acids in residue numbering. To ensure consistency, the reported values have been aligned to the human amino acid numbering across all structures. We added hydrogen atoms to all PDB entries and optimized the structures using GFN-FF with the ALPB implicit solvent model (Ehlert et al, 2021; Pracht et al, 2024; Spicher and Grimme, 2020; Wesolowski et al, 2024). Charges were initialized using the charge-extended Hückel method, originally developed in the context of the charge-adaptive q-vSZP basis set (Muller et al, 2023), and the structures were then re-optimized under the same conditions. During this step, we computed the total SASA and per-residue SASA for each chain in each structure. As the DNA structures attached to the proteins proved highly labile during optimization, we “froze” the DNA atoms. This means the DNA backbone was constrained and could not change conformation during minimization. Nonetheless, interactions between the fixed DNA atoms and the protein were still included and directly affected the optimized protein geometry. Without freezing, the DNA underwent substantial flipping and introduced high-energy artefacts. This behavior is biologically plausible: in vivo, DNA is part of a much longer, stabilized structure, whereas the fragment studied here consists only of a short segment bound to the DNA-binding domain, making it more prone to distortion during geometry optimization. Constraining the DNA in this context results in a model that is more biologically relevant.

### RT-qPCR

Cells were harvested, the lysates were homogenized using a QIAshredder kit (Qiagen, 79656) and the genomic DNA was digested with DNase (Roche, 04716728001) according to the manufacturer's protocol. The RNA was purified using an RNeasy kit (Qiagen, 74104). The cDNA synthesis was performed using an iScript cDNA synthesis kit (Bio-Rad, 1708890). The amplification of cDNA was performed using pairs of primers for *PARP7* (Forward; 5’-TCTCAGGAGCACTTGGAAAGA-3’, Reverse; 5’-TCAGCCTTCGTAGTTGGTCA-3’), *RASD1* (Forward; 5’-CCGCAAGTTCTACTCCATCC-3’, Reverse; 5’-TGAACACCAGGATGAAAACG-3’), *VCL* (Forward; 5’-TGACATTCTACGTTCCCTTGG-3’, Reverse; 5’-TTGGTTTTGGTCTGCAGGTT-3’), *F3* (Forward; 5’-GAACCCAAACCCGTCAATC-3’, Reverse; 5’-CGTCTGCTTCACATCCTTCA-3’), *ERRFI1* (Forward; 5’-GGAGCGCCTAATACCACTTG-3’, Reverse; 5’-CCATTCATCGGAGCAGATTT-3’), *CDKN1A* (Forward; 5’-CTGCCGAAGTCAGTTCCTTG-3’, Reverse; 5’-CATGGGTTCTGACGGACAT-3’), and *AR* (Forward; 5’-GCCTTGCTCTCTAGCCTCAA-3’, Reverse; 5’-TGAATGACAGCCATCTGGTC-3’) in the presence of the HotStart™ 2X Green qPCR Master Mix (APExBIO, K1070), all according to the manufacturer instructions. Technical replicates were averaged and normalized to housekeeping genes GUS (Forward; 5’-CCGACTTCTCTGACAACCGACG-3’, Reverse; 5’-AGCCGACAAAATGCCGCAGACG-3’). Calculations were done using the comparative CT method. Reported are either fold change or relative expression values, depending which were more appropriate for the experimental question. Where needed, fold changes were log_2_ transformed to ensure a continuous scale for genes with increased and decreased expression. In each experiment, three biological replicates were used for each condition.

### Chromatin immunoprecipitation (ChIP)

VCaP cells were fixed using 1% formaldehyde in a growth medium at 37 °C for 10 min and neutralized with 125 mM glycine at room temperature for 5 min. Cells were harvested in cold PBS  +  1 mM PMSF at 4 °C by centrifugation (2200 × *g*, 4 °C, 5 min), resuspended in the extraction buffer (20 mM Tris pH 7.5, 1 mM EDTA, 100 mM NaCl, 2 mM DTT, 0.5% Triton X-100, and protease inhibitors) with end-over-end rotation at 4 °C for 20 min. Pellets were collected by centrifugation (2200 × *g*, 4 °C, 5 min), washed with EDTA-free extraction buffer to remove the EDTA, and then sonicated (0.4-ml volume, Scale 4, 50% cycle, 10 pulse; repeat two more times). Samples were supplemented with CaCl_2_ (4 mM final) and micrococcal nuclease (NEB 500 gel units) to cleave the DNA at 37 °C for 5 min. EGTA (50 mM final) and SDS (0.1%) were added to quench the nuclease reaction. The samples were incubated for 10 min at 4 °C, followed by centrifugation (16,800 × *g*, 4 °C, 20 min). The supernatant was pre-absorbed with unconjugated Protein G beads in the presence of BSA (1 mg/ml final) and salmon sperm DNA (0.2 mg/ml final), then subjected to antibody bead binding at 4 °C for overnight. Beads were collected with centrifugation, washed five times with the extraction buffer supplemented with 0.1% SDS, then resuspended in 100 µl of reverse cross-linking buffer (125 mM Tris pH 6.8, 5% BME, and 1% SDS), and incubated at 65 °C for 6 hr. DNA was purified using the QIAquick Gel Extraction kit (Qiagen, 28704). The amplification of DNA was performed using pairs of primers for the *PARP7* promoter region (forward; 5’-ACAAGGCCCACGAAATAGTC-3’, reverse; 5’-CACCCTGTGAGGAAGCAAAC-3’), and *VCL* enhancer region (forward; 5’-TGTGAGTTGGTGCTGCATAC-3’, reverse; 5’-GGGAGTCAGGAACACAGAGT-3’) in the presence of the HotStart™ 2× Green qPCR Master Mix (APExBIO, K1070), all according to manufacturer instructions. Primers were designed based on AR ChIP-seq binding profiles in VCaP cells from a publicly available dataset (GSE84432, 4 h of R1881 treatment) (Data ref: Toropainen et al, 2016). In each experiment, for every condition, there were three biological replicates (distinct samples).

### Immunoblotting and AR-ADPr detection

After drug treatments, the media was removed, and the cells were lysed using a 1× SDS loading buffer (200 mmol/L Tris-HCl pH 6.8, 2% SDS, 10% glycerol, and 3% β-mercaptoethanol). The lysates were then heated at 95 °C for 5 min, sonicated, centrifuged, separated by SDS-PAGE, and transferred to a nitrocellulose membrane. The membrane was blocked in PBS with 0.15% Tween-20 (PBST) and 5% nonfat milk at room temperature for 45 min. For standard western blotting, it was then incubated with 1° Ab/1% BSA/PBST for either 2 h at room temperature or overnight at 4 °C, washed at room temperature with PBST (five washes of 5 min each), then incubated with fluorescently labeled 2° Ab/1% nonfat milk/PBST for one hour at room temperature, and washed at room temperature with PBST (three washes of 5 min each). For AR-ADPr detection, it was incubated overnight at 4 °C with 1 μg/mL of FL-AF1521^tandem^, or 0.03 μg/mL of FL-eAF1521, and then washed at room temperature with PBST (five washes of 5 min each). The detection was done using the ODYSSEY CLx (LI-COR). The sensitivity of FL-AF1521^tandem^ and FL-eAF1521 at given concentrations appeared to be similar for AR-ADPr detection, and FL-AF1521 refers to either probe.

### Antibodies and siRNAs

Commercial antibodies used were anti-Flag M2 (0.75 μg/ml, 4 °C incubation overnight, Sigma-Aldrich F1804), anti-HA tag (1:1000, Covance A488-101L, mouse mAb 16b12), anti-Ub (1:20,000, ProteinTech 80992-1-RR), anti-T7 (1:10,000, EMD Millipore 69522), anti-DTX2 (1:3000, ProteinTech 67209-1-Ig), anti-HUWE1 (0.6 μg/ml, ProteinTech 19430-1-AP), anti-TRIP12/ULF (50 ng/ml, abcam AB86220), anti-UBR5/EDD (1 mg/ml, Santa Cruz Biotechnology, sc-515494), anti-SPOP (1:10,000, ProteinTech 167501AP), anti-RNF146 (1 μg/ml, abcam ab201212), anti-DTX4 (1 μg/ml, Aviva Systems Biology ARP68284_P050), anti-Tubulin (1:10,000, Sigma-Aldrich T9028), Fluorescein Antibody DyLight 680-conjugated (1:20,000, Rockland 600-144-096), anti-mono-ADP-ribose (1:1000, BIO-RAD TZA020), Donkey anti-Human IgG(H + L) Cross Absorbed Secondary Antibody DyLight 800 (1:10,000, Invitrogen SA5-10132), Donkey anti-human IgG(H + L) cross-absorbed secondary antibody DyLight 800 (1:10,000, Invitrogen SA5-10132), Alexa Fluor 680 donkey anti-rabbit IgG(H + L) (1:20,000, Invitrogen A10043), Alexa Fluor 680 donkey anti-mouse IgG(H + L) (1:20,000, Invitrogen A10038), Anti-mouse IgG(H&L) (Goat) antibody DyLight 800 conjugated (1:10,000, Rockland 610-145-002). Lab-made antibodies are anti-AR (1–21 amino acid, 1 μg/ml for immunofluorescence), anti-AR(659–667 amino acid, 1 μg/ml for western blot), anti-GST (1 μg/ml, T. Parsons’ lab mAb 9D9-F3-F7) and anti-PARP7 (this lab, 1 μg/ml). Flag-AR detection was performed with either anti-AR or M2 antibodies. All of the siRNAs, siCtrl (Am4635), siDTX2 (s223231 & s41519), siDTX1 (s4356), siDTX4 (s23321), siHUWE1 (s19596), siRNF146 (s37822), siSPOP (s15955), siTRIP12 (s17810), siUBR5 (s28025) are from Ambion. Cells were transfected with 20 nM siRNA and Lipofectamine RNAiMAX Reagent (Invitrogen 13778-075) for 24 h, followed by a total of three more days with cell growth and expansion, and drug treatments.

### Drug treatments

Synthetic androgen R1881 (Perkin-Elmer, NLP005005MG; used at 2 nM final concentration in most experiments), natural androgen agonist dihydrotestosterone (DHT, Sigma-Aldrich, D-073), proteasome inhibitor Bortezomib (MedChemExpress, HY-10227; used at 1 μM final concentration), PARP7 inhibitor RBN2397 (DC Chemicals, DC31069), PARP14 inhibitor RBN012759 (MedChemExpress, HY-136979), and PARP1/2 inhibitors Veliparib (Selleck Chemical LLC, S100410MG), Olaparib (MedChemExpress, HY-10162), and Talazoparib (MedChemExpress, HY-16106) were used at concentrations and treatment times specified in the figures or figure legends.

### Recombinant protein production

DNA constructs for the GST-DTX2-RD and GST-DTX2-RD^mut^ (S568A, H582A & H594A) with optimized codons were custom synthesized and transformed into *E. coli* strain *BL21*. Recombinant proteins were induced with 0.4 mM of Isopropyl β-d-1-thiogalactopyranoside at 18 °C overnight; cells were harvested and lysed; proteins were purified on a Glutathione-agarose beads (Sigma 4510). Preparation of recombinant T7-Ub and GST-AF1521^tandem^, and DyLight 800-labeling of the GST-AF1521^tandem^ have been described (Yang et al, 2021).

### In vitro protein binding

The procedures were carried out at 4 °C. Cells were extracted in the Buffer A (20 mM Tris-HCl pH 7.5, 100 mM NaCl, 0.5% Triton X-100 (Tx-100), 1 mM PMSF, 2 mM DTT, 5 mM EDTA, 5 µg/ml each of aprotinin/leupeptin/pepstatin (A/L/P) and 1 µM Veliparib with end-over-end rotation for 20 min (0.5 mM ADP-ribose would also be included in the buffer when making immobilized AR that was used for subsequent Ub assays). The extracts were clarified with centrifugation (16,800 × *g*) for 20 min. The supernatants were subjected to 2.5–4 h bindings with anti-Flag M2 magnetic beads (M8823, for immobilizing Flag-AR), or premade Glutathione MagBeads (GenScript L00895-10)/recombinant GST-fusion proteins (3 μl packed beads/10 μg of protein). The beads were collected under magnetic fields and washed 5–10 times in the Buffer B (20 mM Tris-HCl pH 7.5, 100 mM NaCl, 0.1% Tx-100, 2 mM DTT, 0.1 mM EDTA, 1 µg/ml each of A/L/P and 1 µM Veliparib). The beads were resuspended in the SDS loading buffer, heated at 95 °C for 5 min followed with SDS-PAGE separation, Western blot analysis and Odyssey CLx (LI-COR) detection.

### Ubiquitylation assays

Ubiquitylation assays were performed at 30 °C for 30 min with 1 mM ATP, 100 μg/ml Ub (T7-Ub or bovine Ub (Sigma U6253)), 5 μg/ml each of UB E1 (R&D Systems E-304) and UB E2 (His-UbcH5C, R&D Systems E2-627), and 20 μg/ml GST-DTX2-RD in the buffer E (20 mM Tris-HCl pH 7.5, 50 mM NaCl, 2 mM MgCl_2_, 1 mM DTT, 1 µg/ml each of A/L/P and 0.1 mg/ml BSA). Preparation of AR as a ubiquitylation substrate was performed by IP as described (Yang et al, 2021), with the addition of an N-Ethylmaleimide treatment step to inhibit trace levels of cell-derived E3 activity. Magnetic M2-Beads, carrying immobilized Flag-AR from PC3-AR(siDTX2) with 6 h of 2 nM R1881 treatment and washed thoroughly, were further washed twice with Buffer C (PBS, 1 µg/ml each of A/L/P and 0.1% Tx-100) followed with 5 mM N-Ethylmaleimide/Buffer C treatments at 37 °C for 60 min. These beads were washed twice with the assay buffer plus 0.1% Tx-100, once with the assay buffer lacking TX-100, and subsequently used in ubiquitylation assays. Ubiquitylation assays using a chemically synthesized AR peptide (1 μM) with ADP ribosylation on Cys284 (Wijngaarden et al, 2023) and FITC-labeled on the N-terminus were performed using the same reaction conditions as cell-derived AR. Conjugation products were detected by immunoblotting with anti-Fluorescein (FITC) antibody. NUDT16 reactions were conducted at 30 °C for 30 min or at 37 °C for 60 min with 2.5, 5, 10, or 25 μM NUDT16 in Buffer D (20 mM Tris-HCl pH 7.5, 50 mM NaCl, 5 mM MgCl_2_, 2 mM DTT, 1 µg/ml each of A/L/P and 0.1 mg/ml BSA). USP2 treatments were carried out at 37 °C for 15 min with 1 μM His-USP2-CD (catalytic domain, 258-606AA) in the Buffer F (50 mM Tris-HCl pH 7.5, 0.5 mM EDTA, 1 mM DTT, 1 μg/ml A/L/P and 0.1 mg/ml BSA).

### Immunofluorescence microscopy

Procedures were done at 23 °C (room temperature). Cells on coverslips were rinsed with PBS, fixed in 10% Neutral Buffered Formalin for 20 min, rinsed with PBS, permeabilized in PBS/0.2% Tx-100 (PBST) for 20 min, rinsed three times with PBST, blocked in 5% BSA/PBST for 30 min, incubated with 1° Ab/1% BSA/PBST for 2 h, washed three times with PBST, incubated with 2° Ab/1% BSA/PBST for 2 h, washed three times with PBST, incubated with 2 μg/ml 4’,6-diamidino-2-phenylindole/PBS for 15 s or more, rinsed with milli-Q water, mounted on slides with ProLong Gold antifade reagent (Invitrogen P36934). Images shown in the figures were acquired by confocal microscopy (Stellaris 8; Leica) equipped with a 100×/1.40 Oil STED White oil immersion objective and Leica software (LAS X).

### Statistical information

All statistical analysis and data visualization were done in R/RStudio (R Core Team, 2024; Posit Team, 2025). Mathematical modeling was performed in MATLAB (version R2023b) using the ODE solver ode23s to solve the ODE system of mass-action equations. Pixel Pearson correlation for images was done in Fiji (Schindelin et al, 2012) with the JaCoP plugin (Bolte and Cordelieres, 2006). For the differential expression analysis, *P* values were calculated using the two-tailed Wald test. For GSEA, the *P* values were calculated using the two-tailed permutation-based testing with 500,000 permutations. For ORA, the p values were calculated using the one-tailed hypergeometric test. All adjusted p values in this study were calculated using the Benjamini–Hochberg (BH) method. For SSE score comparison between models, *P* values were calculated using the two-tailed Wilcoxon test. For RT-qPCR relative expression comparisons, *P* values were calculated using the two-tailed Welch’s *t* test. If the exact *P* values are not stated, the following symbols were used to indicate significance: ns: *P* > 0.05; *P ≦ 0.05; **P ≦ 0.01; ***P ≦ 0.001; ****P ≦ 0.0001. For qPCR, a bar or a data point is an average of three biological replicates (distinct samples), and error bars represent the standard deviation. For correlation analysis, Pearson correlation was used to calculate a coefficient and *P* values (two-tailed). For multivariate Cox regression, *P* values were calculated using the Wald test. For the Kaplan–Meier (KM) analysis, *P* values were calculated using the two-tailed log-rank test. For RNA-seq and GSEA, we defined *P*-adj <0.001 as significant.

## Supplementary information

Appendix

Peer Review File

Source data Fig. 1

Source data Fig. 2

Source data Fig. 3

Source data Fig. 4

Source data Fig. 5

Source data Fig. 6

Source data Fig. 7

Source data Fig. 8

Appendix Fig 1-6 Source Data

## Data Availability

RNA-seq data that support the findings of this study have been deposited in the National Center for Biotechnology Information Gene Expression Omnibus and are accessible through the GEO Series accession number GSE272500. The source data of this paper are collected in the following database record: biostudies:S-SCDT-10_1038-S44318-025-00510-4.
