# Supplementary material for: Parp7 generates an ADP-ribosyl degron that controls negative feedback of androgen signaling
Source: EMBO J. 2025 Jul 18;44(17):4720–44. doi: 10.1038/s44318-025-00510-4 (PMC12402299; doi:10.1038/s44318-025-00510-4)
Supplement: Supplementary file 1 — Appendix [file 44318_2025_510_MOESM1_ESM.pdf]

Title: Parp7 generates an ADP-ribosyl degron that controls negative feedback of androgen signaling

Authors: Krzysztof Wierbiłowicz1,2,**, Chun-Song Yang1,**, Ahmed Almaghasilah3, Patryk A. Wesołowski4, Philipp Pracht4, Natalia M. Dworak5, Jack Masur6, Sven Wijngaarden7, Dmitri V. Filippov7, David J. Wales4, Joshua B. Kelley3, Aakrosh Ratan1,2, Bryce M. Paschal1,*

APPENDIX FIGURES, TABLES AND Documents

Appendix Contents

Appendix	Appendix Legend	Page
Appendix Fig. S1	Gene expression changes in response to androgen and RBN2397 treatment, related to Fig. 1.	2
Appendix Fig. S2	Heatmap showing the module eigengene expression for the 19 modules included in the analysis, related to Fig. 2.	3
Appendix Fig. S3	AR protein levels in cells treated with androgen, RBN2397, and bortezomib, related to Fig. 3.	4
Appendix Fig. S4	AR-Parp7 interactions analyzed by confocal microscopy, related to Fig. 4.	5
Appendix Fig. S5	Analysis of AR ADP-ribosylation mutants, related to Fig. 5.	6
Appendix Fig. S6	Mono-ADP-ribose recognition by the DTC domain in DTX2, related to Fig. 6.	7
Appendix Fig. S7	Predictions regarding the effect of DTX2 on Ub conjugation and AR, related to Fig. 7.	8
Appendix Table S1	Model Parameters and Scores	9
Appendix Table S2	Standard Deviation of the Top 100 Simulations	10
Appendix Table S3	Bayesian Information Criterion (BIC), Bayes Weights Scores and minimum Sum of Square Errors (SSE).	11
Appendix Table S4	AR ADP-ribosylation site mutants used in this study	12
Appendix Table S5	ΔE calculations for different configurations. All with GFN-FF/ALPB (water)	13
Appendix Table S6	Solvent Accessible Surface Area (SASA) calculation in Bohr² for different AR DBD structures and chosen Cys residues	14
Appendix Method 1	Model equations	15
Appendix Method 2	Equations for Sum of squared errors (SSE), Bayesian Information Criterion (BIC) and Bayes Weights related to Appendix Table S3.	17

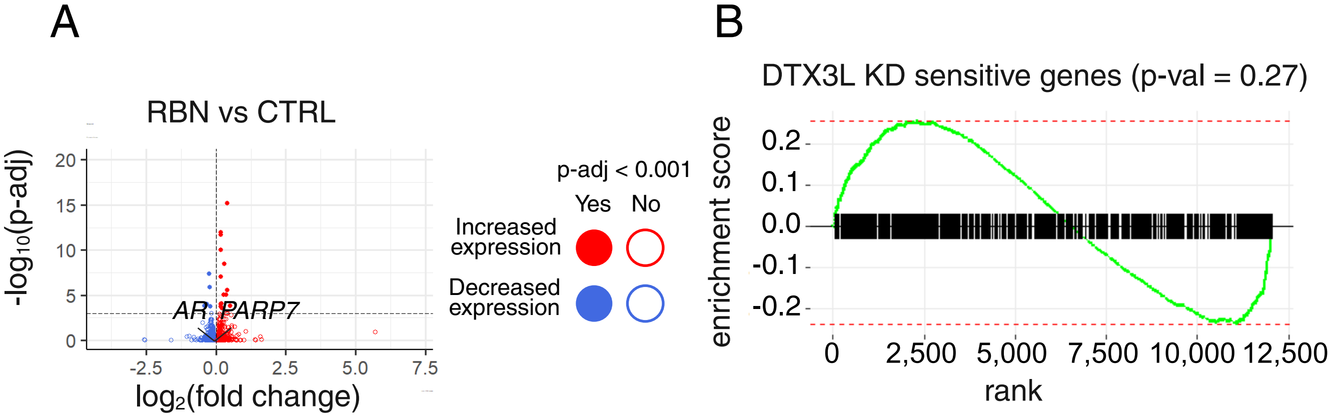

Appendix Fig. S1: Gene expression changes in response to androgen and RBN2397 treatment, related to Fig. 1.

A, Volcano plot depicting the differential gene expression analysis in VCaP cell line comparing gene expression between RBN2397-treated and untreated samples (RBN vs CTRL). Each dot represents a gene, with the x-axis showing the $\log_2$ fold change (Log_2FC) and the y-axis representing the negative $\log_{10}$ of the adjusted p-value (p-adj). Genes that are significantly upregulated or downregulated are highlighted in red and blue, respectively, based on the preset p-adj (0.001) threshold.

B, Enrichment plot depicting the enrichment of genes affected by shDTX3L knockdown in R1881+RBN vs R1881 differentially expressed genes. For the GSEA gene set, we utilized DTX3L KD-sensitive genes from samples treated with R1881, excluding any genes influenced by DTX3L KD under basal conditions. The x-axis in both plots represents a ranked list of R1881+RBN vs. R1881 differentially expressed genes based on log2 fold change, while the y-axis displays the enrichment score values. The green line in the plot shows the trend of enrichment scores, with positive values indicating an increased presence of gene sets toward the top of the ranked list, and negative values indicating an increased presence of gene sets toward the bottom of the ranked list. The analysis rendered insignificant with p-value = 0.27.

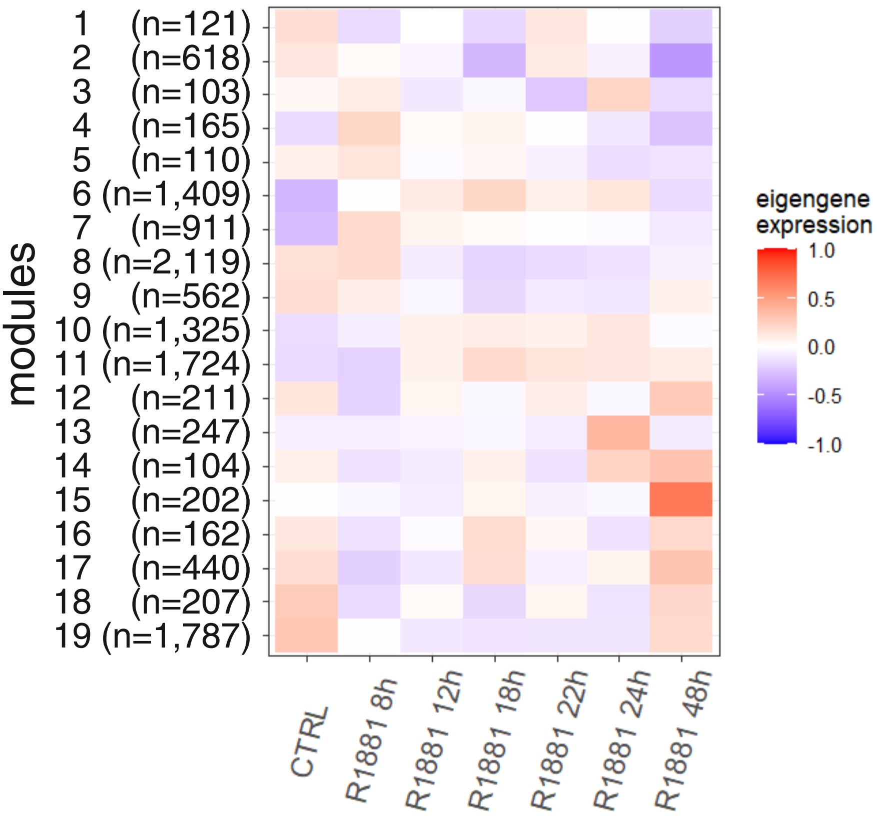

Appendix Fig. S2: Heatmap showing the module eigengene expression for the 19 modules included in the analysis, related to Fig. 2.
Module names, together with the number of genes, are presented on the y-axis. The x-axis represents the time points of the R1881 treatment. The representative color gradient used for the heatmap is presented in the figure.

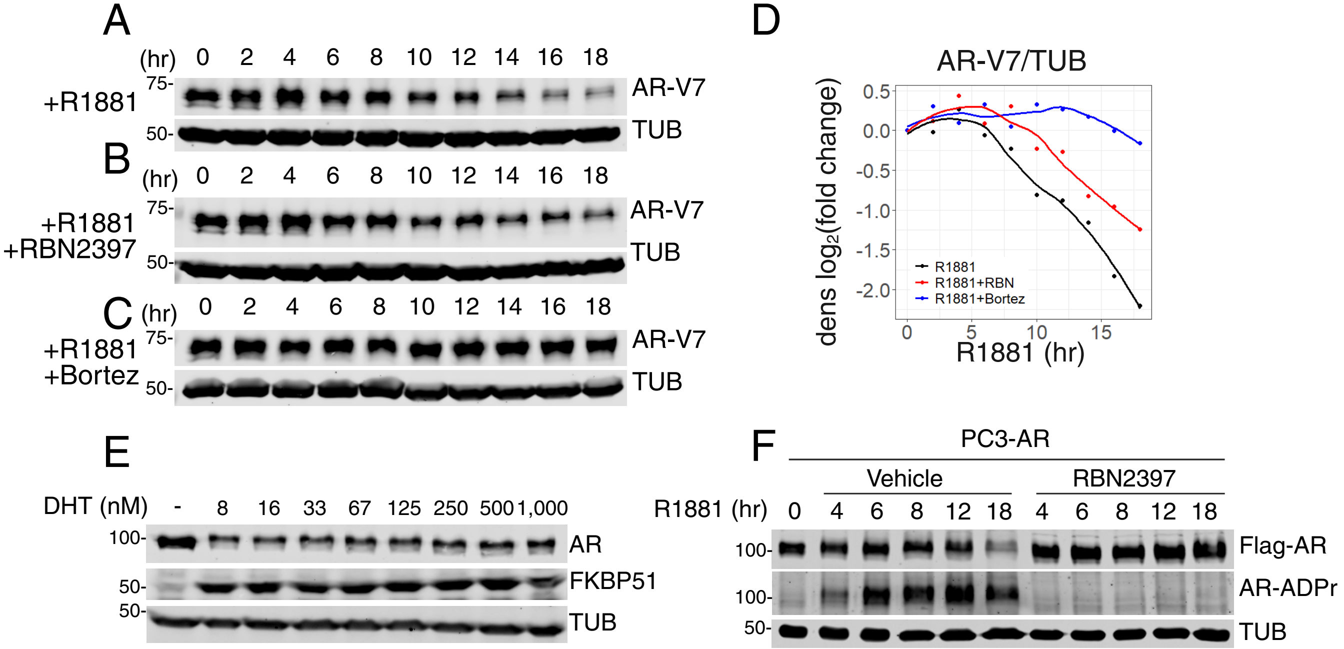

Appendix Fig. S3: AR protein levels in cells treated with androgen, RBN2397, and bortezomib, related to Fig. 3.

A, Immunoblot detection of AR-V7 in VCaP cells subjected to a time course of R1881 treatment. Cells were collected every 2 hours for an 18-hour period (the same experiment as Figure 3A). The Tubulin loading control used for this panel is the same as in Figure 3A as it is part of the same blot.

B, Immunoblot detection of AR-V7 in VCaP cells subjected to a time course of R1881 and RBN2397 cotreatment. Cells were collected every 2 hours for an 18-hour period (the same experiment as Figure 3B).

C, Immunoblot detection of AR-V7 in VCaP cells subjected to a time course of R1881 and Bortezomib (Bortez) cotreatment. Cells were collected every 2 hours for an 18-hour period (the same experiment as Figure 3C).

D, Line plot visualizing the AR-V7 protein density measurements for immunoblots from panels A-C. The y-axis represents the $\log_2$ of the AR-V7/TUB density fold change from 0-hr time point, and the x-axis represents the time of treatment in hours. The curves were fitted using the loess method.

E, Immunoblot detection of AR in VCaP cells treated with a concentration range of DHT for 10 hr.

F, Immunoblot detection of Flag-AR and AR-ADPr (FL-AF1521) in PC3-AR cells treated with R1881 and co-treated with R1881 and RBN2397 for times indicated on the panel.

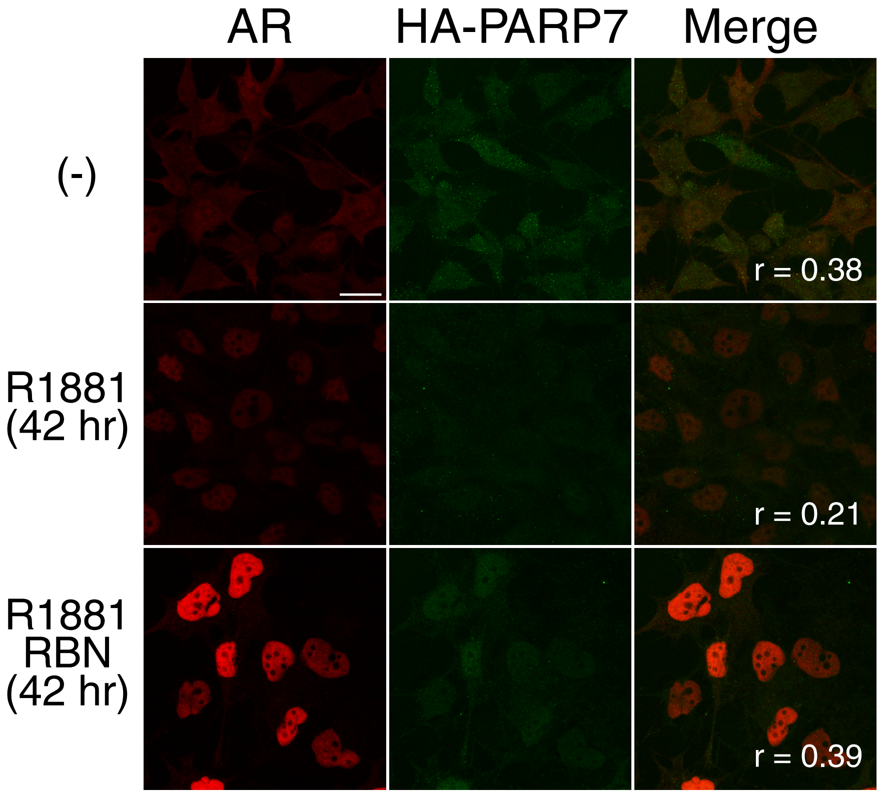

Appendix Fig. S4: AR-Parp7 interactions analyzed by confocal microscopy, related to Fig. 5. Confocal microscopy staining of HA-PARP7 and AR in PC3-AR HA-PARP7 cells treated with R1881 and co-treated with R1881 and RBN2397 for times indicated on the panel. The third column shows merged channels, and the Pearson correlation coefficient for pixel colocalization is indicated in the bottom left corner for every condition. A scale bar of 10μm is provided on the bottom right corner of the upper left panel and applies to all panels. These images including the untreated control sample (-) are from the same experiment shown in Fig. 5D. The comparison demonstrates the RBN2397 effect on AR stabilization is maintained for at least 42 hr.

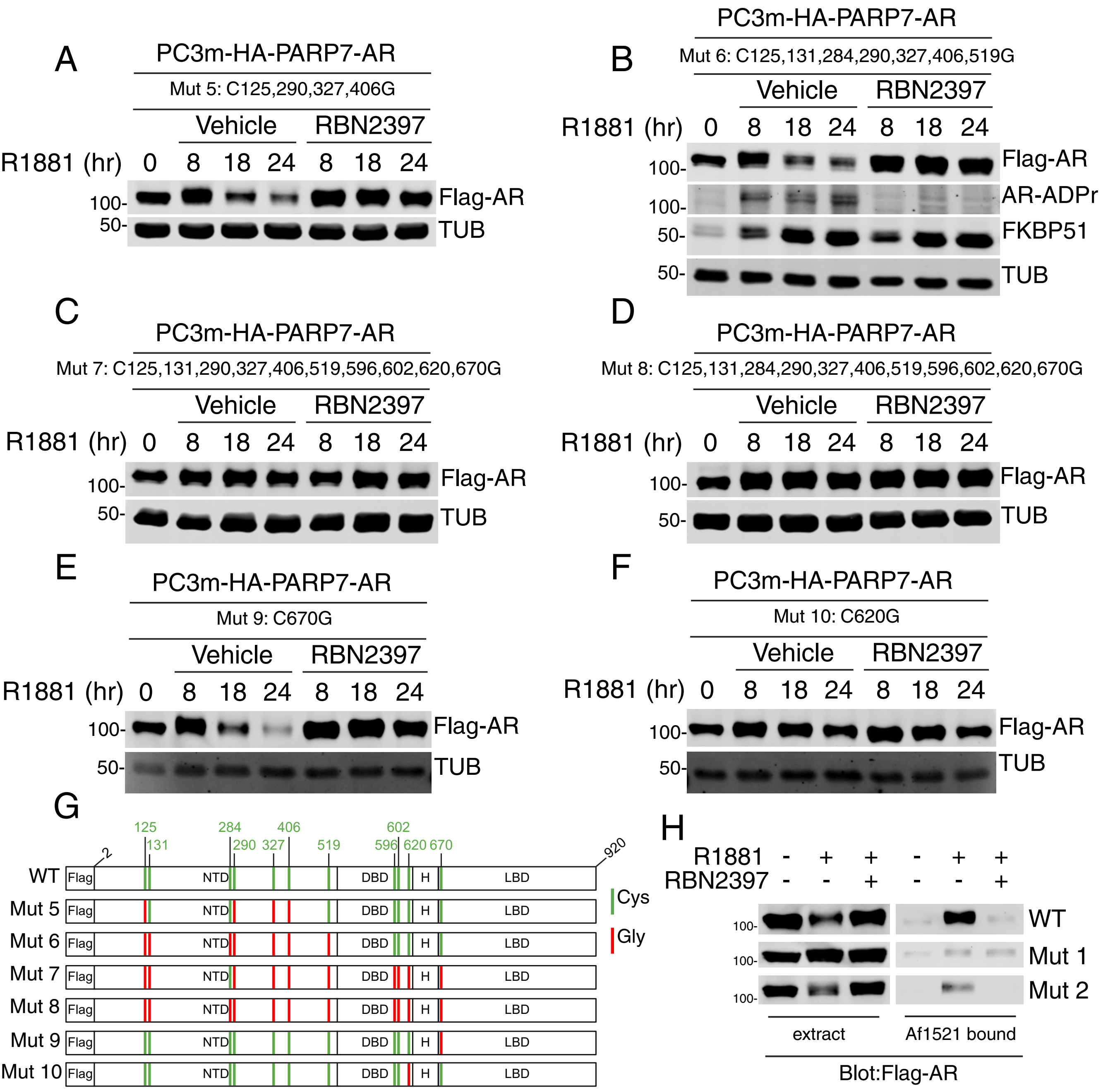

Appendix Fig. S5: Analysis of AR ADP-ribosylation mutants, related to Fig. 6.
A-F, Immunoblot detection of Flag-AR, TUB, AR-ADPr (FL-AF1521), and FKBP51 in PC3m(HA-PARP7/Flag-AR Mutants) cells treated with R1881 or co-treated with R1881 and RBN2397 for times indicated on the panels. A: AR Mut 5 (C125,290,327,406G); B: AR Mut 6 (C125,131,284,290,327,406,519G); C: AR Mut 7 (C125,131,290,327,406,519,596,602,620,670G); D: AR Mut 8 (C125,131,284,290,327,406,519,596,602,620,670G); E: AR Mut 9 (C670G); F: AR Mut 10 (C620G).
G, Diagrams of Flag-AR mutants employed in this figure (Mut 5-10). All of the ADP-ribosyl cysteine sites on AR are marked in green, and the glycine substitutions are marked in red.
H, Immunoblot detection of the Flag-AR protein in PC3m(HA-PARP7/Flag-AR WT, Mut 1 and Mut 2) cell extracts and GST-AF1521tandem bound fractions. Cell extracts from cells treated with different combinations of R1881 and RBN2397 for 18 hr and were combined with magnetic Glutathione/GST-AF1521tandem beads for the enrichment of ADP-ribosylated proteins.

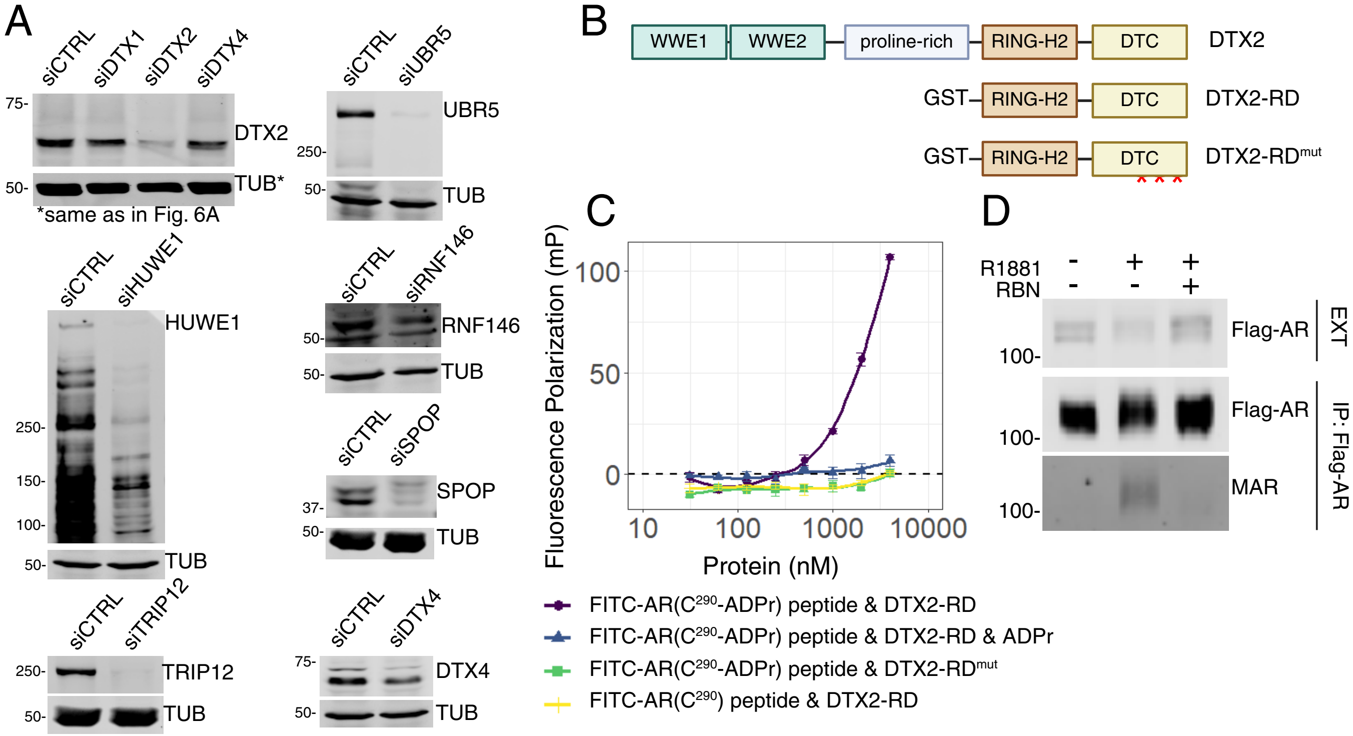

Appendix Fig. S6: Mono-ADP-ribose recognition by the DTC domain in DTX2, related to Fig. 7.
A, Knockdown (siRNA) and immunoblotting of E3 Ub ligases with reader domains, or known roles in nuclear receptor degradation.
B, Diagrams of DTX2 full length, DTX2-RD, and DTX2-RD^{mut}. Three loss of function substitutions in the DTC domain of DTX2-RD^{mut} (S568A, H582A, and H594A) are indicated with red asterisk.
C, Line plot presenting the binding measurements of DTX2-RD or DTX2-RD^{mut} to FITC-AR(C290-ADPr) or FITC-AR(C290) peptides by fluorescence polarization. The y-axis represents Fluorescence Polarization (mP) and the x-axis represents the concentration of DTX2-RD or DTX2-RD^{mut}. AR peptide sequence: FITC-PLAEC(-/+ADPr)KGSL-OH.
D, Immunoblot detection of the Flag-AR and mono-ADP-ribosylation (MAR) protein in the PC3-AR cell extracts and Flag-AR immunoprecipitation. Cell extracts from cells treated with different combinations of R1881 and RBN2397 for 18 hr and were combined with magnetic anti-Flag M2 beads for the IP.

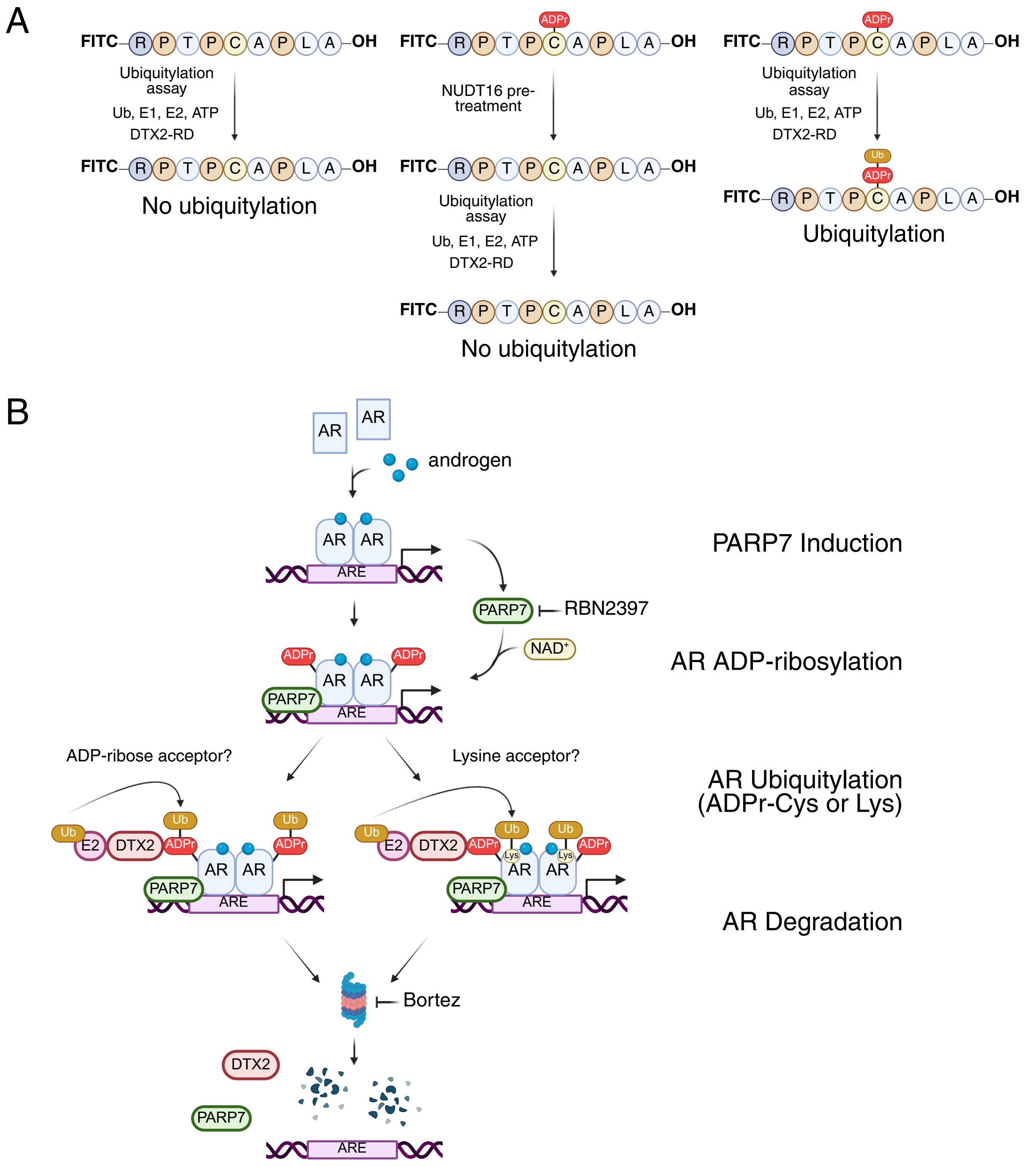

Appendix Fig. S7: Predictions regarding the effect of DTX2 on Ub conjugation and AR, related to Fig. 8.
A, Schematic presenting different molecular outcomes of FITC-AR(C290-ADPr) or FITC-AR(C290) peptides in-vitro ubiquitylation assays from Figure 7A.
B, Schematic presenting the molecular mechanism of negative feedback mediated by DTX2-dependent degradation of ADP-ribosylated AR.

Appendix Table S1: Model Parameters

Parameter	Simple	Chromatin	Nucleoplasm	Description of Parameter
k_1	0.95992	0.70809	0.52759	ADP-ribosylation of AR driven by PARP7
k_{1c}	-	0.16789	0.00011	AR/Promoter complex transcription
k_{1d}	0.32369	0.50713	0.05186	Degradation of unmodified AR
k_{1r}	0.02442	0.92896	0.83305	Removal of ADP-Ribosylation from AR
k_{1t}	0.44196	0.01295	0.00794	Production of AR
k_2	0.83291	0.85090	0.06761	AR association with the promoter
k_{2c}	-	0.85968	0.56508	ADP-AR/Promoter transcription
k_{2d}	0.67309	0.98985	0.80955	Degradation of ADP-AR
k_{2r}	-	0.78152	0.77838	AR dissociation from the promoter
k_{2t}	0.67067	0.31458	0.79561	Production of PARP7 influenced by AR
k_3	-	0.00010	0.00013	ADP-AR association with the promoter
k_{3d1}	0.17950	0.47086	0.85877	Degradation of PARP7
k_{3d2}	0.94157	0.67953	0.91768	Degradation of PARP7 influenced by AR
k_{3r}	-	0.91374	0.74739	ADP-AR dissociation from the promoter
k_{4d}	0.00094	0.13421	0.13743	Degradation of Transcript

Appendix Table S2: Standard Deviation of the Top 100 Simulations

Parameter	Simple	Nucleoplasm	Chromatin
k_1	0.31	0.0042	0.0055
k_{1c}	-	0.0002	0.0033
k_{1d}	0.29	0.0038	0.0041
k_{1r}	0.29	0.0063	0.0070
k_{1t}	0.29	0.0023	0.0050
k_2	0.36	0.0054	0.0086
k_{2c}	-	0.0079	0.0049
k_{2d}	0.30	0.0046	0.0062
k_{2r}	-	0.002	0.0071
k_{2t}	0.29	0.0062	0.0061
k_3	-	0.0002	0.00009
k_{3d1}	0.31	0.0048	0.0057
k_{3d2}	0.33	0.0052	0.0034
k_{3r}	-	0.005	0.0030
k_{4d}	0.06	0.0029	0.0018

Appendix Table S3: Bayesian Information Criterion (BIC), Bayes Weights Scores and minimum Sum of Square Errors (SSE).

	Simple Model	*Chromatin Model*	*Nucleoplasm Model*
BIC	127.2	113.3	119.3
Bayes Weights	0.001	0.952	0.047
Minimum SSE	19520.5	1545	2820.6

Related equations were shown in Appendix Document 2.

Appendix Table S4. AR ADP-ribosylation site mutants used in this study

	Amino acid substitutions in AR	Cys620 status	Androgen-induced AR degradation
WT	-	WT	YES
Mut 1	C125G, C131G, C290G, C327G, C406G, C519G, C620G, C670G	Gly	NO
Mut 2	C125G, C131G, C290G, C327G, C406G, C519G, C670G	WT	YES
Mut 3	C125G, C131G, C290G, C327G, C406G, C519G, C620S, C670G	Ser	NO
Mut 4	C620S	Ser	NO
Mut 5	C125G, C290G, C327G, C406G	WT	YES
Mut 6	C125G, C131G, C284G, C290G, C327G, C406G, C519G	WT	YES
Mut 7	C125G, C131G, C290G, C327G, C406G, C519G, C596G, C602G C620G, C670G	Gly	NO
Mut 8	C125G, C131G, C284G, C290G, C327G, C406G, C519G, C596G, C602G C620G, C670G	Gly	NO
Mut 9	C670G	WT	YES
Mut 10	C620G	Gly	NO

Appendix Table S5. ΔE calculations for different configurations. All with GFN-FF/ALPB (water).

Total energy in Hartree	C3 human	MTV human	DR3 rat
with DNA prior to opt	-603.3935989	-616.239546	-607.4714762
with DNA after opt	-606.3053626	-618.9651857	-610.6616056
with DNA (frozen) prior to opt	-603.3935989	-616.239546	-607.4714762
with DNA (frozen) after opt	-605.0617947	-617.7630953	-609.6269508
w/o DNA prior to opt	-351.2266241	-362.5897872	-396.7006269
w/o DNA after opt	-353.830337	-364.1898206	-398.7289144
DNA only prior opt	-249.609532	-249.9681148	-249.9466248
DNA only after opt	-250.8981618	-250.9830409	-250.9078873
ΔE / kcal/mol	-989.25	-1124.85	-611.66
ΔE (frozen) / kcal/mol	-1018.13	-1007.33	-597.31

Appendix Table S6. Solvent Accessible Surface Area (SASA) calculation in Bohr² for different AR DBD structures and chosen Cys residues.

SASA in Bohr² AR DBD	Total	Cys596 A/B	Cys602 A/B	Cys620 A/B
C3 human SASA w/o DNA prior to opt	2557.8784	6.3838/5.4469	5.5905/3.7328	0.1704/0.3667
C3 human SASA w/o DNA after opt	2359.7045	7.5163/10.7813	1.3695/0.3962	0.0227/0.0497
C3 human SASA with DNA prior to opt	2279.0549	6.7446/5.4892	5.3382/3.4600	0.1887/0.3593
C3 human SASA with DNA after opt	1869.4311	13.4328/5.1732	0.6342/2.5431	0.0016/0.0035
C3 human SASA with DNA (frozen) after opt	1999.2805	13.5294/5.4508	1.0354/1.7809	0.0452/0.3845
MTV human SASA w/o DNA prior to opt	2671.1243	7.3760/7.0292	7.1639/7.6712	0.1398/0.1952
MTV human SASA w/o DNA after opt	2344.6967	11.7253/4.3741	0.2952/2.8770	0.0387/0.0393
MTV human SASA with DNA prior to opt	2387.4262	7.3760/7.0293	7.1639/7.6712	0.1397/0.1952
MTV human SASA with DNA after opt	2387.4262	15.7439/4.8532	0.4056/3.7273	0.0103/0.0049
MTV human SASA with DNA (frozen) after opt	2111.7004	12.6129/6.1987	0.3746/2.7349	0.1920/0.0172
DR3 rat SASA w/o DNA prior to opt	2583.2319	5.0872/7.3593	6.2711/4.1015	1.4755/1.0337
DR3 rat SASA w/o DNA after opt	2226.0186	11.0264/14.4600	1.8883/3.8246	0.1308/0.0370
DR3 rat SASA with DNA prior to opt	2329.4252	5.0872/7.3260	6.2711/3.8429	1.4755/1.0337
DR3 rat SASA with DNA after opt	1816.4176	9.9828/9.8957	1.8361/0.7941	0.2682/0.2996
DR3 rat SASA with DNA (frozen) after opt	1949.5837	12.1481/7.6244	2.5796/4.7822	0.5425/0.0775

Appendix Document 1

-RBN

Simple model

$$\frac{dAR}{dt} = k_{1t} \times AR_{Transcript} + k_{1r} \times AR_{ADP} - k_1 \times AR \times PARP7 - k_{1d} \times AR - k_2 \times AR$$

$$\frac{dAR_{ADP}}{dt} = k_1 \times AR \times PARP7 - k_{1r} \times AR_{ADP} - k_{2d} \times AR_{ADP}$$

$$\frac{dPARP7}{dt} = k_{2t} \times AR \times PARP7_{transcript} - k_{3d1} \times PARP7 - k_{3d2} \times PARP7 \times AR$$

$$\frac{dTranscript}{dt} = k_2 \times AR - k_{4d} \times Transcript$$

Nucleoplasm model

$$\frac{dAR}{dt} = k_{1t} \times AR_{Transcript} + k_{1r} \times AR_{ADP} + k_{2r} \times AR\_P - k_1 \times AR \times PARP7 - k_{1d} \times AR - k_2 \times AR \times P$$

$$\frac{dAR_{ADP}}{dt} = k_1 \times AR \times PARP7 + k_{3r} \times AR_{ADP}\_P - k_{1r} \times AR_{ADP} - k_{2d} \times AR_{ADP} - k_3 \times P \times AR_{ADP}$$

$$\frac{dPARP7}{dt} = k_{2t} \times AR \times PARP7_{transcript} - k_{3d1} \times PARP7 - k_{3d2} \times PARP7 \times AR$$

$$\frac{dP}{dt} = k_{2r} \times AR\_P + k_{3r} \times AR_{ADP}\_P - k_2 \times P \times AR - k_3 \times P \times AR_{ADP}$$

$$\frac{dAR\_P}{dt} = k_2 \times P \times AR - k_{2r} \times AR\_P$$

$$\frac{dTranscript}{dt} = k_{1c} \times AR\_P + k_{2c} \times AR_{ADP}\_P - k_{4d} \times Transcript$$

$$\frac{dAR_{ADP}\_P}{dt} = k_3 \times P \times AR_{ADP} - k_{3r} \times AR_{ADP}\_P$$

Chromatin model

$$\frac{dAR}{dt} = k_{1t} \times AR_{Transcript} + k_{2r} \times AR\_P - k_2 \times AR \times P$$

$$\frac{dAR_{ADP}}{dt} = k_{3r} \times AR_{ADP}\_P - k_{2d} \times AR_{ADP} - k_3 \times P \times AR_{ADP}$$

$$\frac{dPARP7}{dt} = k_{2t} \times AR \times PARP7_{transcript} - k_{3d1} \times PARP7 - k_{3d2} \times PARP7 \times AR$$

$$\frac{dP}{dt} = k_{2r} \times AR\_P + k_{3r} \times AR_{ADP}\_P - k_2 \times P \times AR - k_3 \times P \times AR_{ADP}$$

$$\frac{dAR\_P}{dt} = k_2 \times P \times AR + k_{1r} \times AR_{ADP}\_P - k_{2r} \times AR\_P - k_1 \times AR\_P \times PARP7$$

$$\frac{dTranscript}{dt} = k_{1c} \times AR\_P + k_{2c} \times AR_{ADP}\_P - k_{4d} \times Transcript$$

$$\frac{dAR_{ADP}\_P}{dt} = k_3 \times P \times AR_{ADP} + k_1 \times AR\_P \times PARP7 - k_{3r} \times AR_{ADP}\_P - k_{1r} \times AR_{ADP}\_P$$

+RBN

Simple model

$$\frac{dAR}{dt} = k_{1t} \times AR_{Transcript} + k_{1r} \times AR\_P - k_{1d} \times AR - k_2 \times AR$$

$$\frac{dAR_{ADP}}{dt} = -k_{1r} \times AR_{ADP} - k_{2d} \times AR_{ADP}$$

$$\frac{dPARP7}{dt} = k_{2t} \times AR \times PARP7_{transcript} - k_{3d1} \times PARP7 - k_{3d2} \times PARP7 \times AR$$

$$\frac{dTranscript}{dt} = k_2 \times AR - k_{4d} \times Transcript$$

Nucleoplasm model

$$\frac{dAR}{dt} = k_{1t} \times AR_{Transcript} + k_{1r} \times AR_{ADP} + k_{2r} \times AR\_P - k_{1d} \times AR - k_2 \times AR \times P$$

$$\frac{dAR_{ADP}}{dt} = k_{3r} \times AR_{ADP}\_P - k_{1r} \times AR_{ADP} - k_{2d} \times AR_{ADP} - k_3 \times P \times AR_{ADP}$$

$$\frac{dPARP7}{dt} = k_{2t} \times AR \times PARP7_{transcript} - k_{3d1} \times PARP7 - k_{3d2} \times PARP7 \times AR$$

$$\frac{dP}{dt} = k_{2r} \times AR\_P + k_{3r} \times AR_{ADP}\_P - k_2 \times P \times AR - k_3 \times P \times AR_{ADP}$$

$$\frac{dAR\_P}{dt} = k_2 \times P \times AR - k_{2r} \times AR\_P$$

$$\frac{dTranscript}{dt} = k_{1c} \times AR\_P + k_{2c} \times AR_{ADP}\_P - k_{4d} \times Transcript$$

$$\frac{dAR_{ADP}\_P}{dt} = k_3 \times P \times AR_{ADP} - k_{3r} \times AR_{ADP}\_P$$

Chromatin model

$$\frac{dAR}{dt} = k_{1t} \times AR_{Transcript} + k_{2r} \times AR\_P - k_2 \times AR \times P$$

$$\frac{dAR_{ADP}}{dt} = k_{3r} \times AR_{ADP}\_P - k_{2d} \times AR_{ADP} - k_3 \times P \times AR_{ADP}$$

$$\frac{dPARP7}{dt} = k_{2t} \times AR \times PARP7_{transcript} - k_{3d1} \times PARP7 - k_{3d2} \times PARP7 \times AR$$

$$\frac{dP}{dt} = k_{2r} \times AR\_P + k_{3r} \times AR_{ADP}\_P - k_2 \times P \times AR - k_3 \times P \times AR_{ADP}$$

$$\frac{dAR\_P}{dt} = k_2 \times P \times AR + k_{1r} \times AR_{ADP}\_P - k_{2r} \times AR\_P$$

$$\frac{dTranscript}{dt} = k_{1c} \times AR\_P + k_{2c} \times AR_{ADP}\_P - k_{4d} \times Transcript$$

$$\frac{dAR_{ADP}\_P}{dt} = k_3 \times P \times AR_{ADP} - k_{3r} \times AR_{ADP}\_P - k_{1r} \times AR_{ADP}\_P$$

Appendix Document 2

Sum of squared errors (SSE)

$$SSE = \sum_{m=1}^{n} (y_m - \widehat{y_m})^2 + (y_{RBN-m} - \widehat{y_{RBN-m}})^2$$

y_m: The predicted (estimated) value for the *m*-th data point.

$\widehat{y_m}$: The actual (observed) value for the *m*-th data point.

y_{RBN-m}: The predicted (estimated) value for the *m*-th data point for the RBN treatment

$\widehat{y_{RBN-m}}$: The actual (observed) value for the *m*-th data point for the RBN treatment

n: The total number of data points.

Bayesian Information Criterion (BIC)

The Bayesian Information Criterion (BIC) is a criterion for selecting a model among a certain set of models. BIC is used as a method to asset the models using the likelihood function. It also includes a penalty term, preventing overfitting, for the number of parameters in the model. Model with the lowest BIC is generally preferred. The formula for BIC is [https://link.springer.com/article/10.3758/BF03206482, https://www.sciencedirect.com/science/article/pii/B9780128146231000071, https://www.sciencedirect.com/science/article/pii/B9780323901727000257

$$BIC = k\,ln(n) - 2\,ln(L)$$

Where;

- k is the number of parameters in the model
- n is the number of data points
- L is the likelihood of the model given the data
- $ln(L)$ log likelihood

$$L(\theta) = \prod_{i=1}^{n} \frac{1}{\sqrt{2\pi\sigma^2}} exp(\frac{(y_i - f(x_i,\theta))^2}{2\sigma^2})$$

$$ln(L) = -\frac{n}{2} ln(2\pi\sigma^2) - \frac{1}{2\sigma^2} \sum_{i=1}^{n} (y_i - f(x_i,\theta))^2$$

Where;

- $f(x_i,\theta)$ is the model's predicted value
- y_i is the observed data
- θ are the model parameters

σ^2 is the variance of the errors

You can simplify the equation since we calculate $\sum_{i=1}^{n} \quad (y_i - f(x_i, \theta))^2$ as SSE, we can basically substitute it with SSE in the equation

$$ln(L) = -\frac{n}{2} ln(2\pi\sigma^2) - \frac{1}{2\sigma^2} SSE$$

$$\sigma^2 = \frac{SSE}{n}$$

Therefore,

$$\Rightarrow ln(L) = -\frac{n}{2} ln(2\pi \frac{SSE}{n}) - \frac{1}{2\frac{SSE}{n}} SSE \Rightarrow -\frac{n}{2} ln(2\pi \frac{SSE}{n}) - \frac{n}{2}$$

Bayes Weights

Beside calculating BIC, Bayes weights are used to provide a probabilistic measure, a normalized score between 0 and 1. Given the data and the set of models, The highest Bayes weight refers to the best model among the considered models. The weight for model *m* is calculated as [https://link.springer.com/article/10.3758/BF03206482]:

$$w_m = \frac{exp(-1/2 * \Delta_m(BIC) \ )}{\sum_{k=1}^{M} \quad exp(-1/2 * \Delta_k(BIC))}$$

$$\Delta_i = (BIC_i - min(BIC))$$
