# Supplementary figures and images for: Parp7 generates an ADP-ribosyl degron that controls negative feedback of androgen signaling
Source: EMBO J. 2025 Jul 18;44(17):4720–44. doi: 10.1038/s44318-025-00510-4 (PMC12402299; doi:10.1038/s44318-025-00510-4)

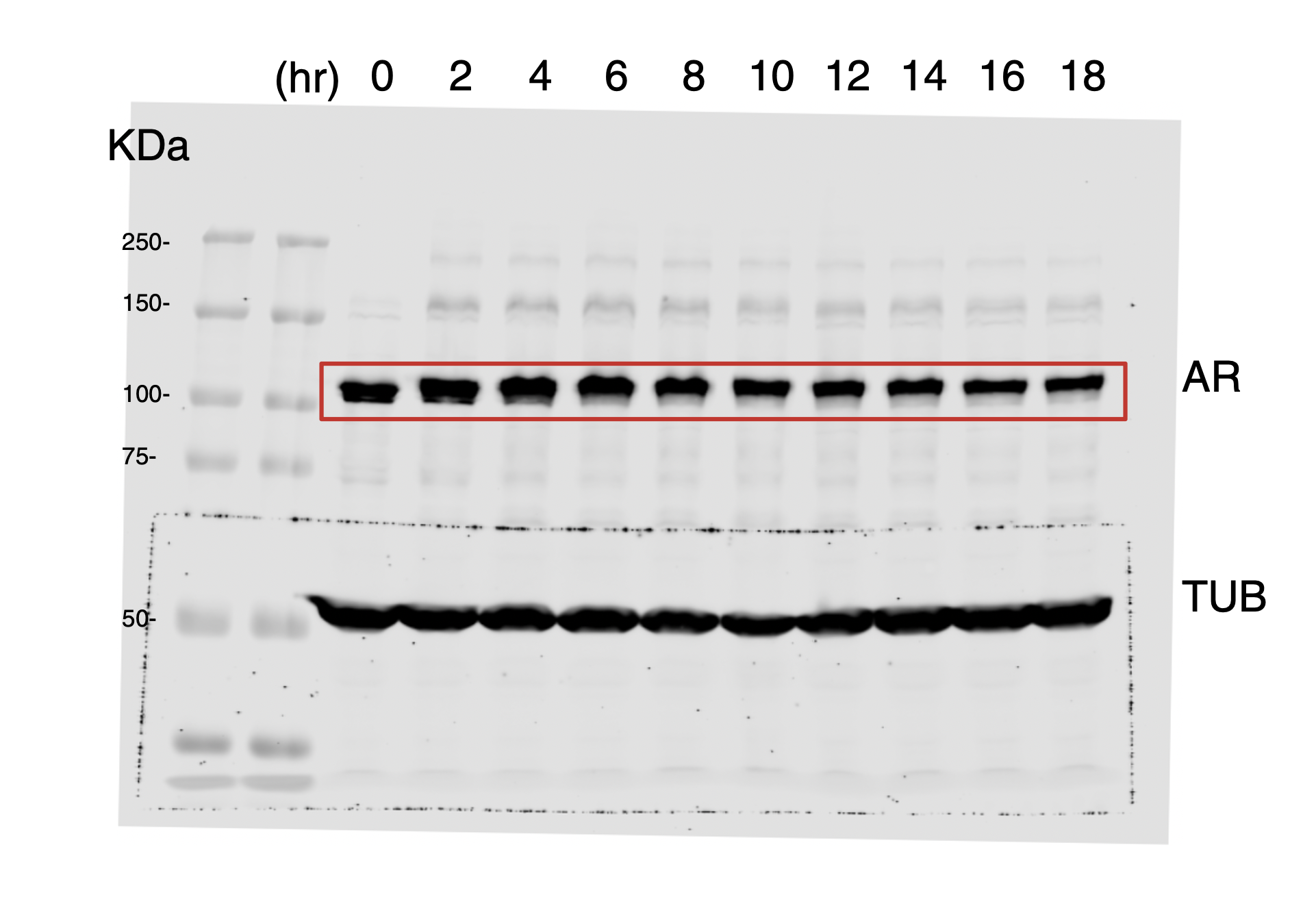

Supplement: Supplementary file 5 — Source data Fig. 3 [file 44318_2025_510_MOESM5_ESM.zip › Figure 3/3B/AR.png]

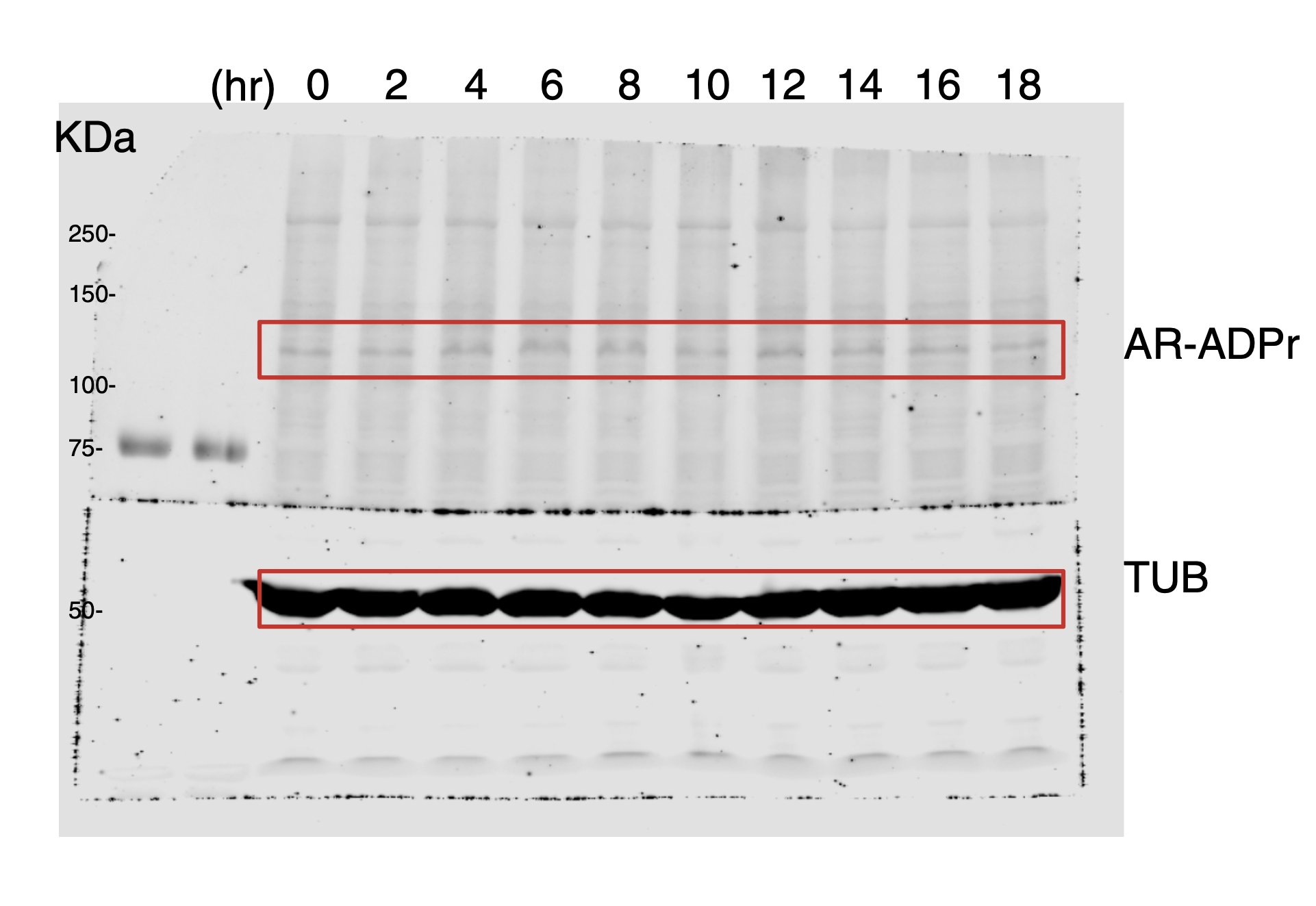

Supplement: Supplementary file 5 — Source data Fig. 3 [file 44318_2025_510_MOESM5_ESM.zip › Figure 3/3B/AR-ADPr and TUB.png]

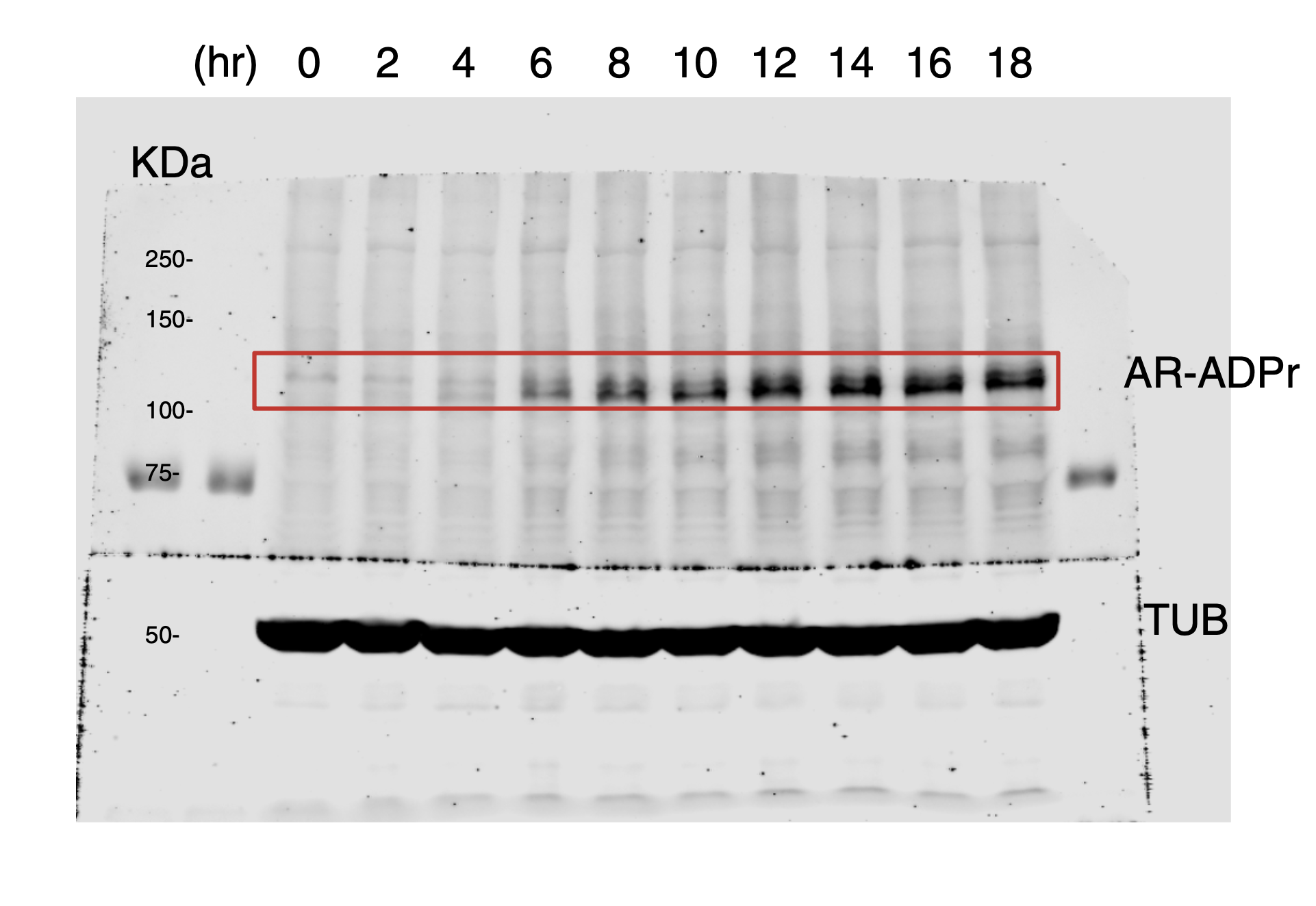

Supplement: Supplementary file 5 — Source data Fig. 3 [file 44318_2025_510_MOESM5_ESM.zip › Figure 3/3C/AR-ADPr.png]

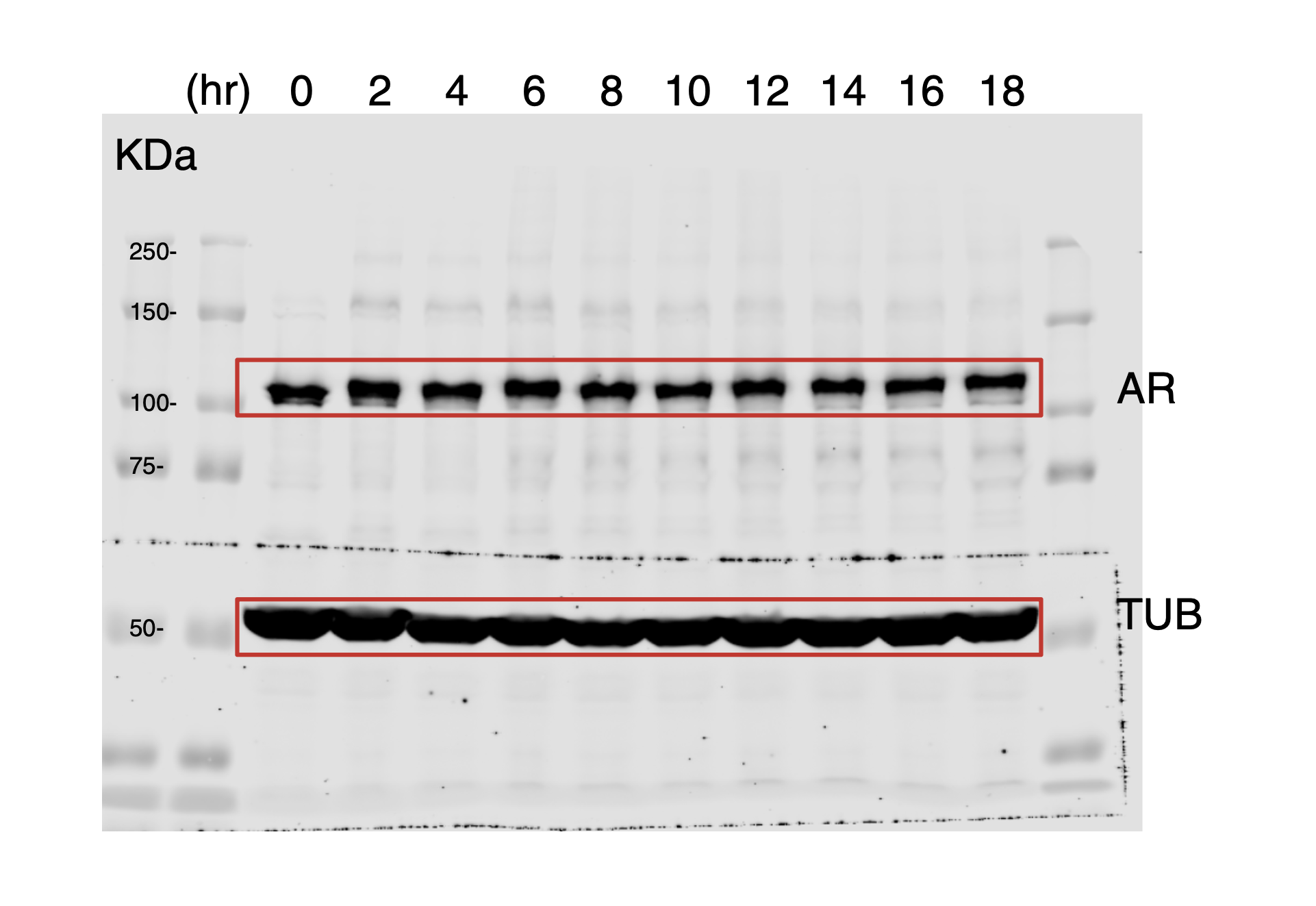

Supplement: Supplementary file 5 — Source data Fig. 3 [file 44318_2025_510_MOESM5_ESM.zip › Figure 3/3C/AR and TUB.png]

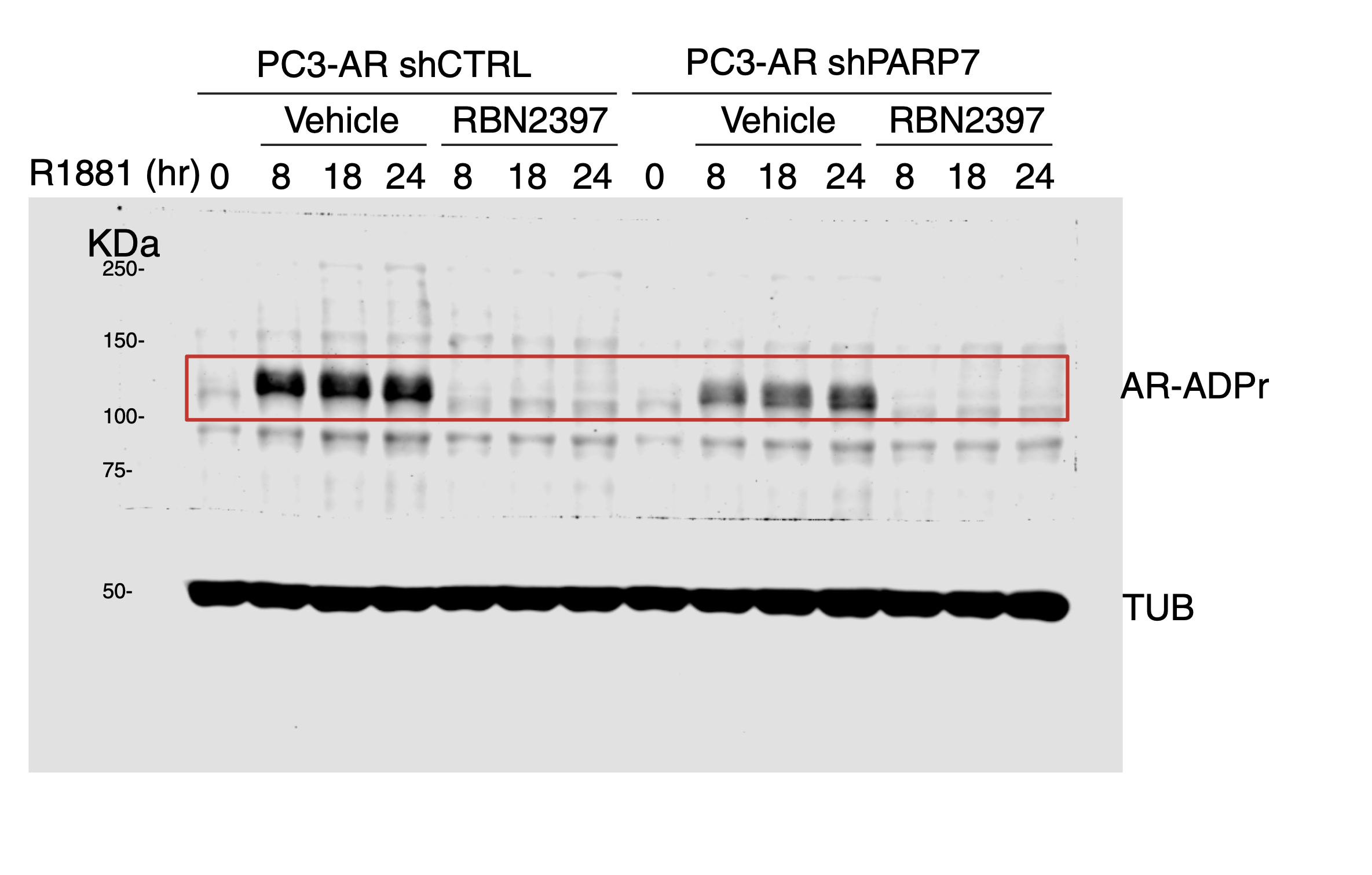

Supplement: Supplementary file 5 — Source data Fig. 3 [file 44318_2025_510_MOESM5_ESM.zip › Figure 3/3H/AR-ADPr.png]

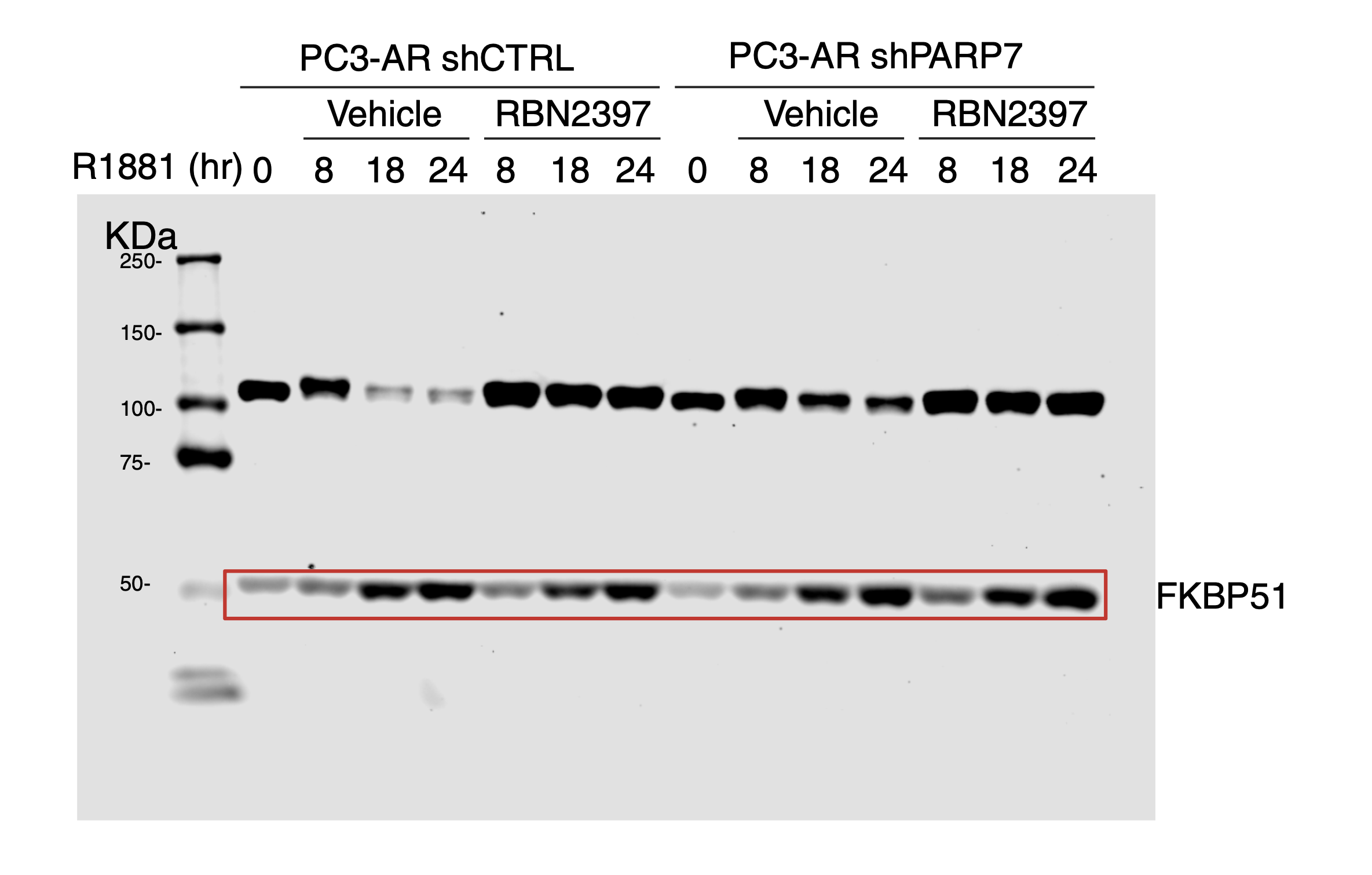

Supplement: Supplementary file 5 — Source data Fig. 3 [file 44318_2025_510_MOESM5_ESM.zip › Figure 3/3H/FKBP51.png]

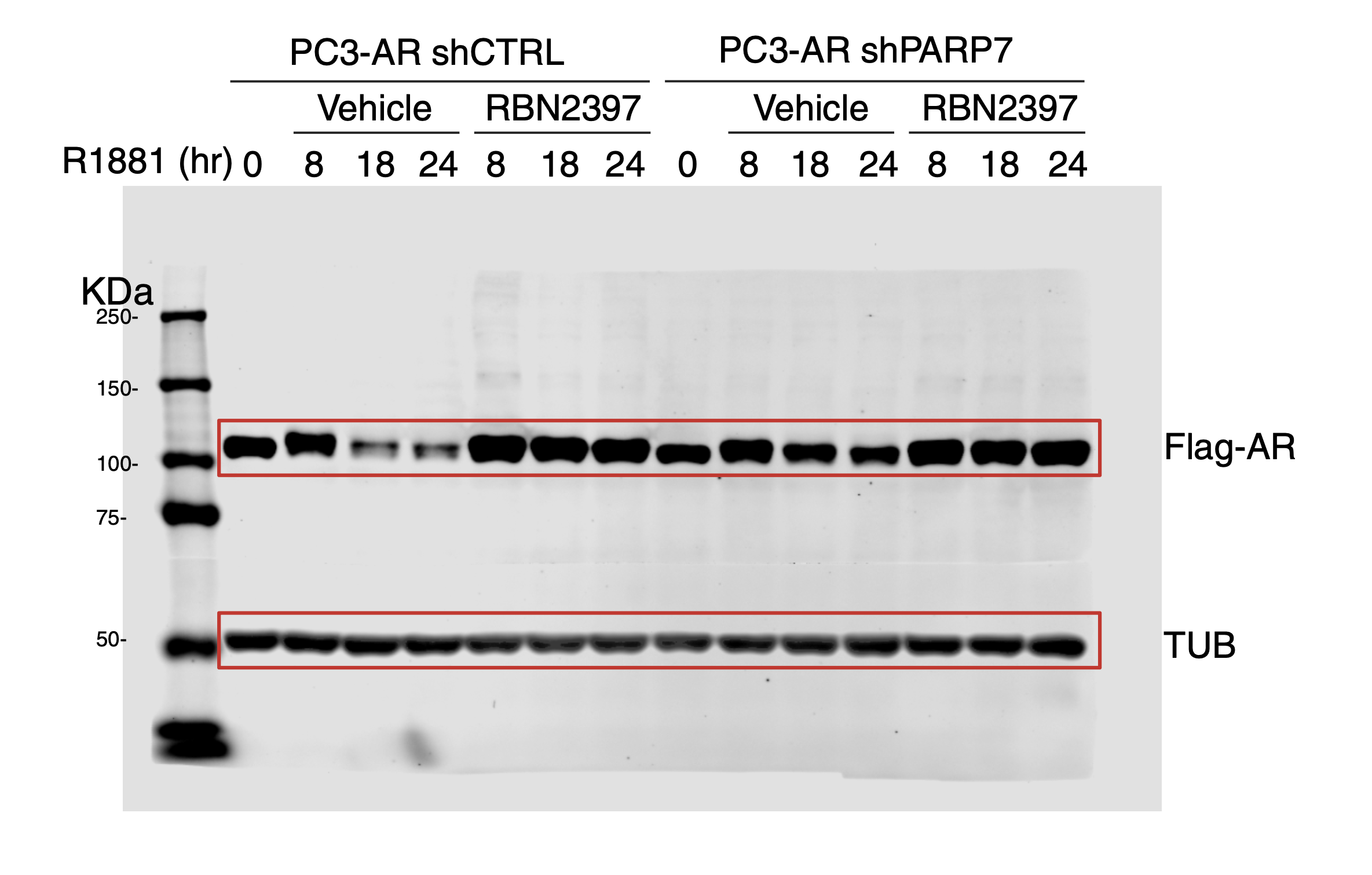

Supplement: Supplementary file 5 — Source data Fig. 3 [file 44318_2025_510_MOESM5_ESM.zip › Figure 3/3H/Flag-AR and TUB.png]

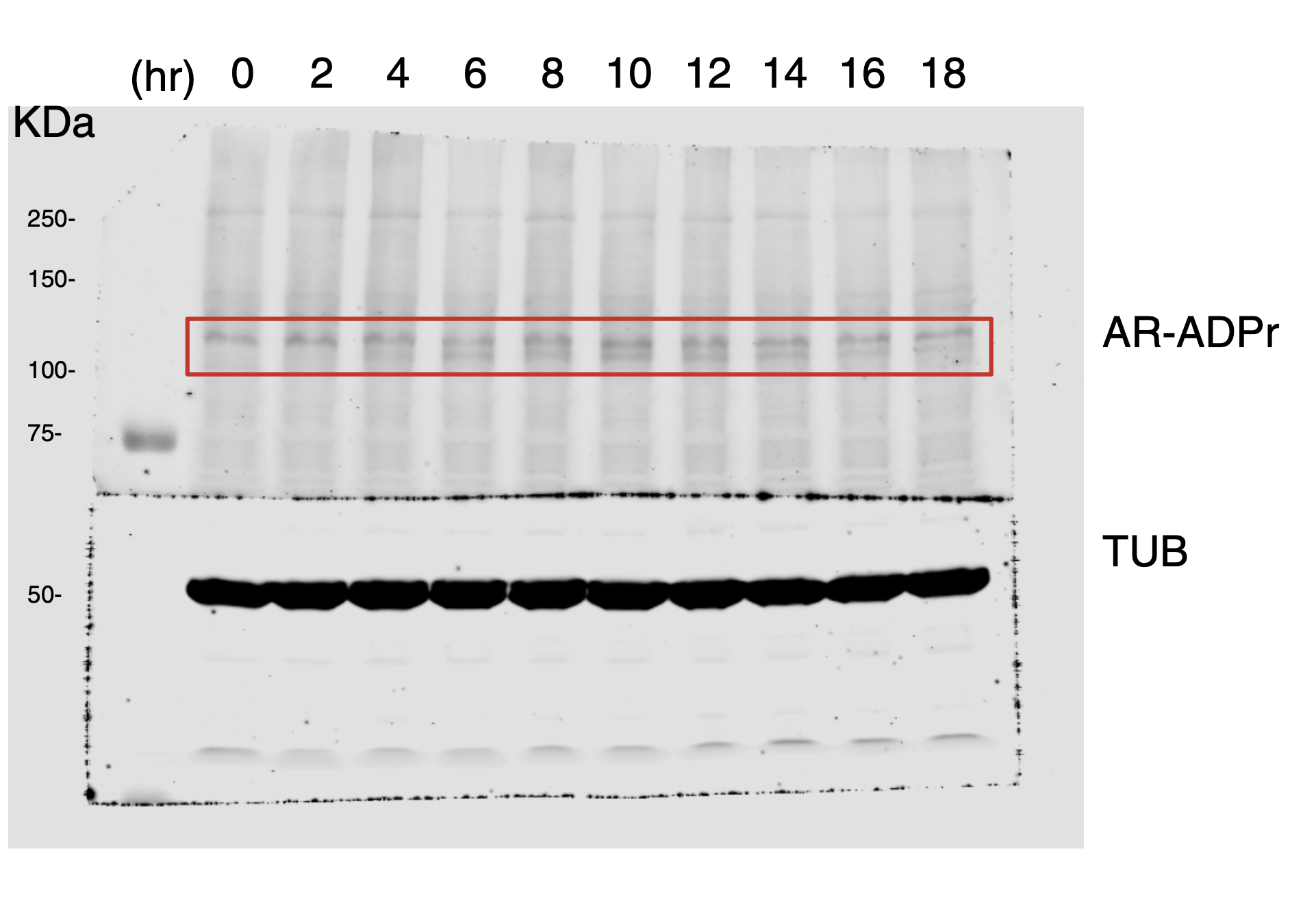

Supplement: Supplementary file 5 — Source data Fig. 3 [file 44318_2025_510_MOESM5_ESM.zip › Figure 3/3A/AR-ADPr.png]

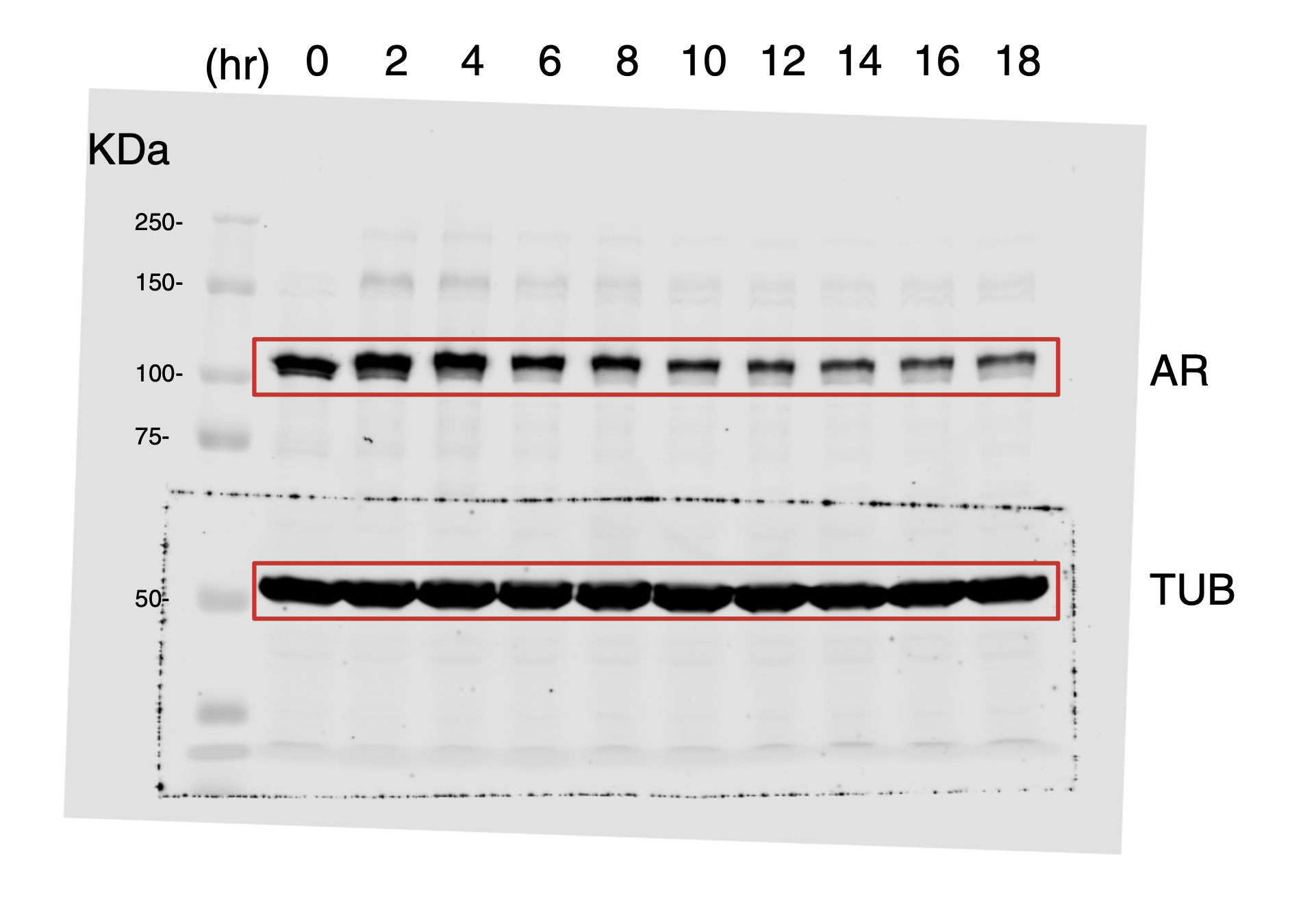

Supplement: Supplementary file 5 — Source data Fig. 3 [file 44318_2025_510_MOESM5_ESM.zip › Figure 3/3A/AR and TUB.png]

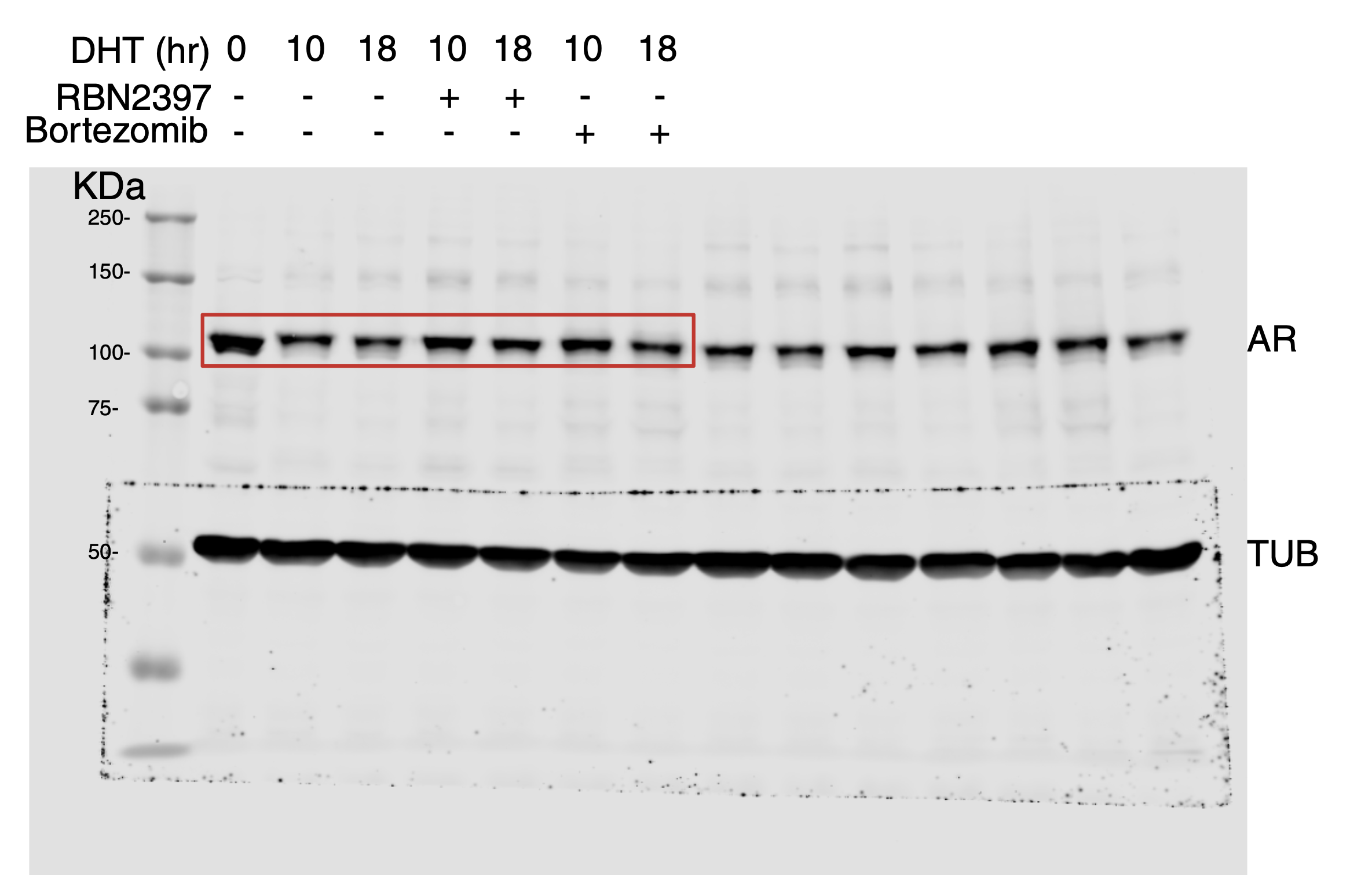

Supplement: Supplementary file 5 — Source data Fig. 3 [file 44318_2025_510_MOESM5_ESM.zip › Figure 3/3F/AR.png]

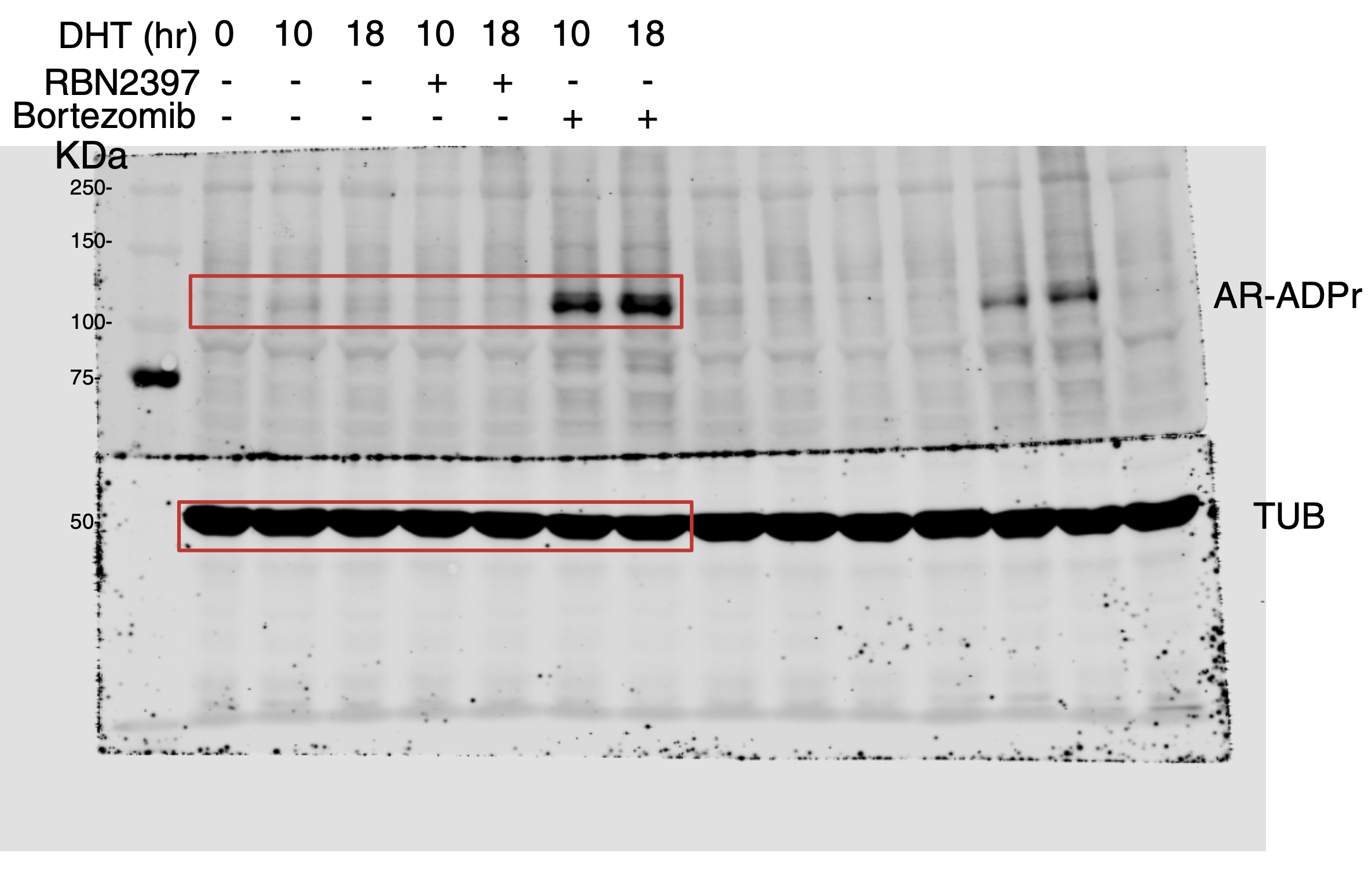

Supplement: Supplementary file 5 — Source data Fig. 3 [file 44318_2025_510_MOESM5_ESM.zip › Figure 3/3F/AR-ADPr_TUB.png]

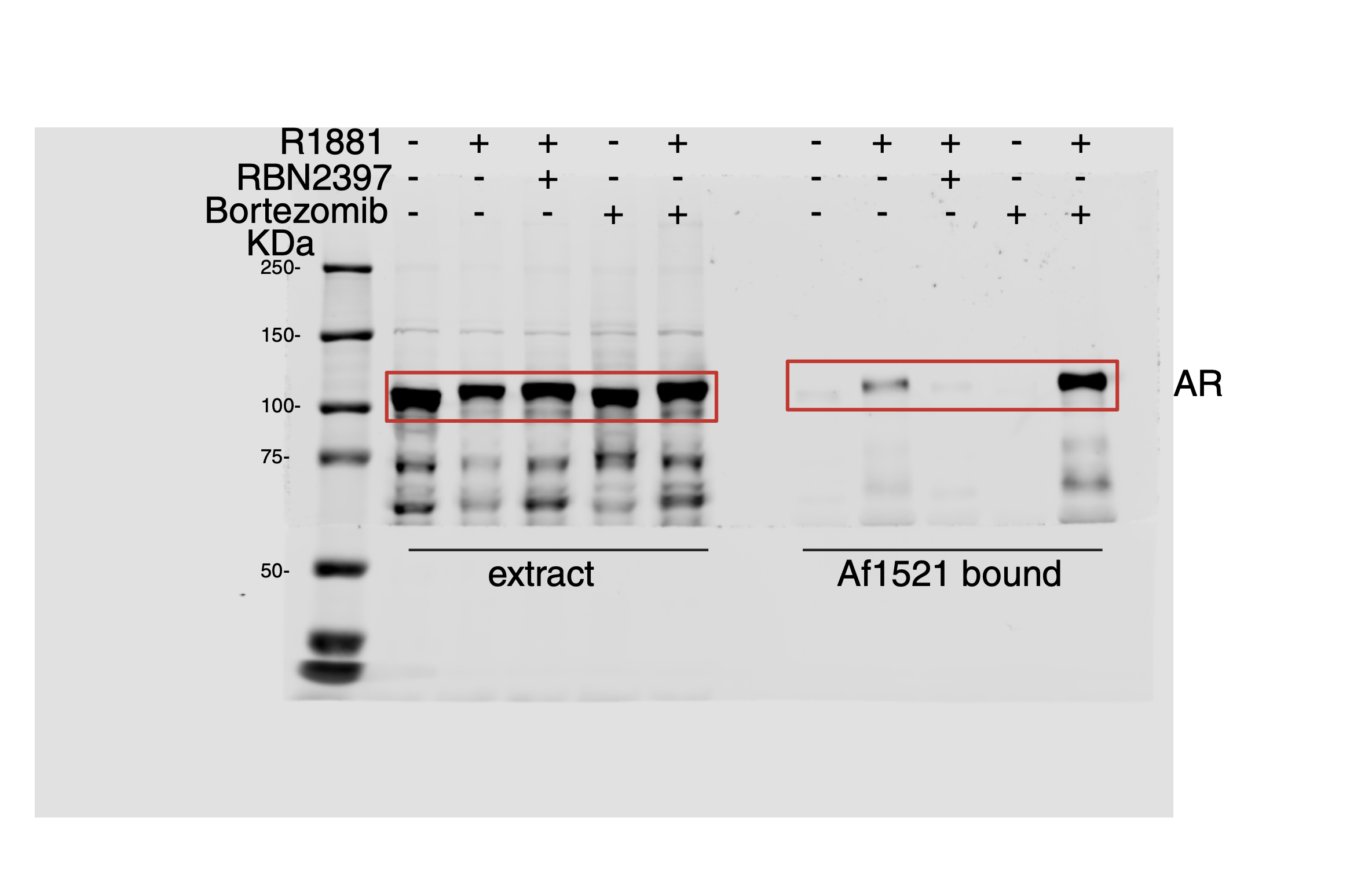

Supplement: Supplementary file 5 — Source data Fig. 3 [file 44318_2025_510_MOESM5_ESM.zip › Figure 3/3G/AR.png]

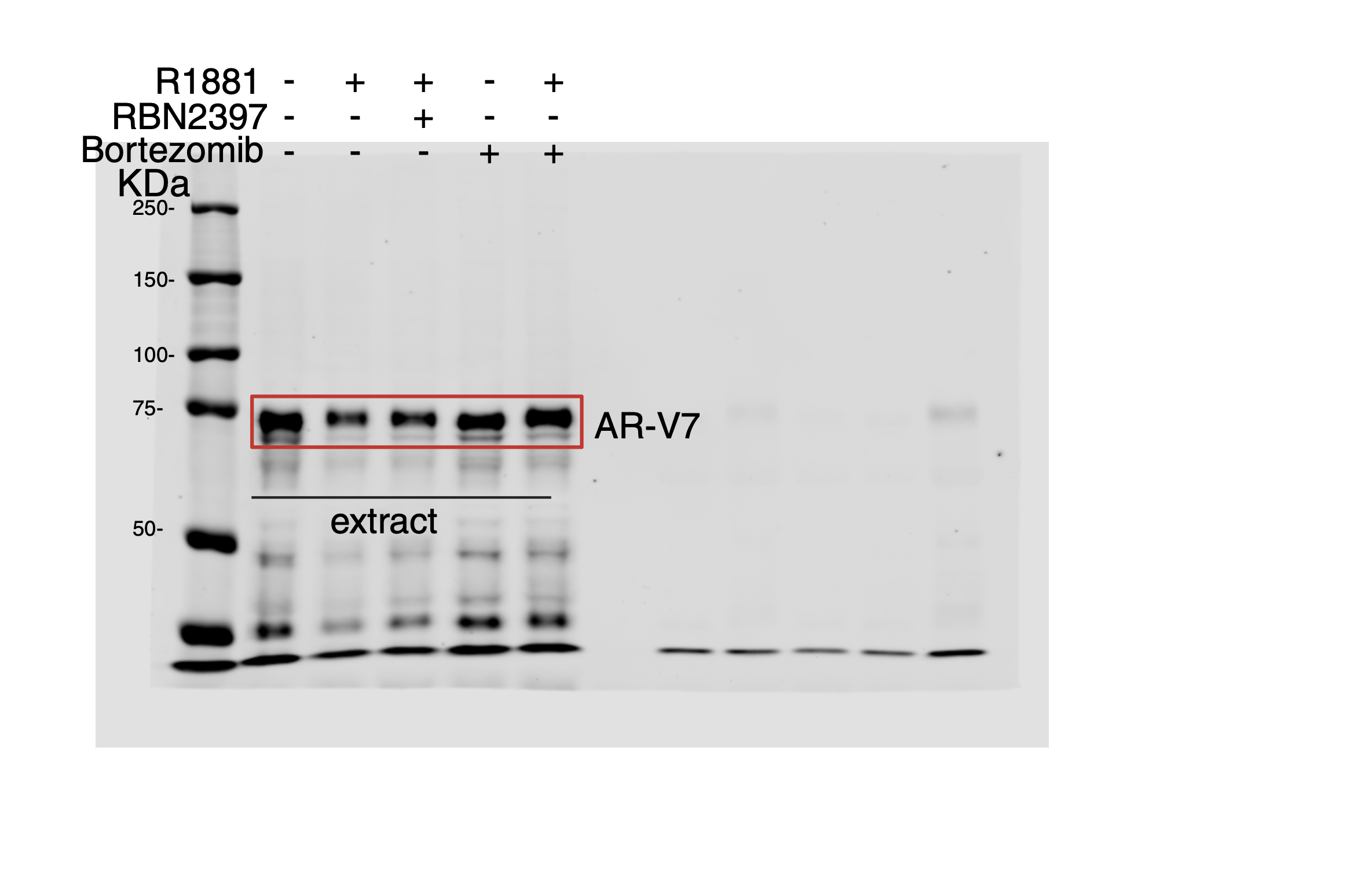

Supplement: Supplementary file 5 — Source data Fig. 3 [file 44318_2025_510_MOESM5_ESM.zip › Figure 3/3G/AR-V7 extract.png]

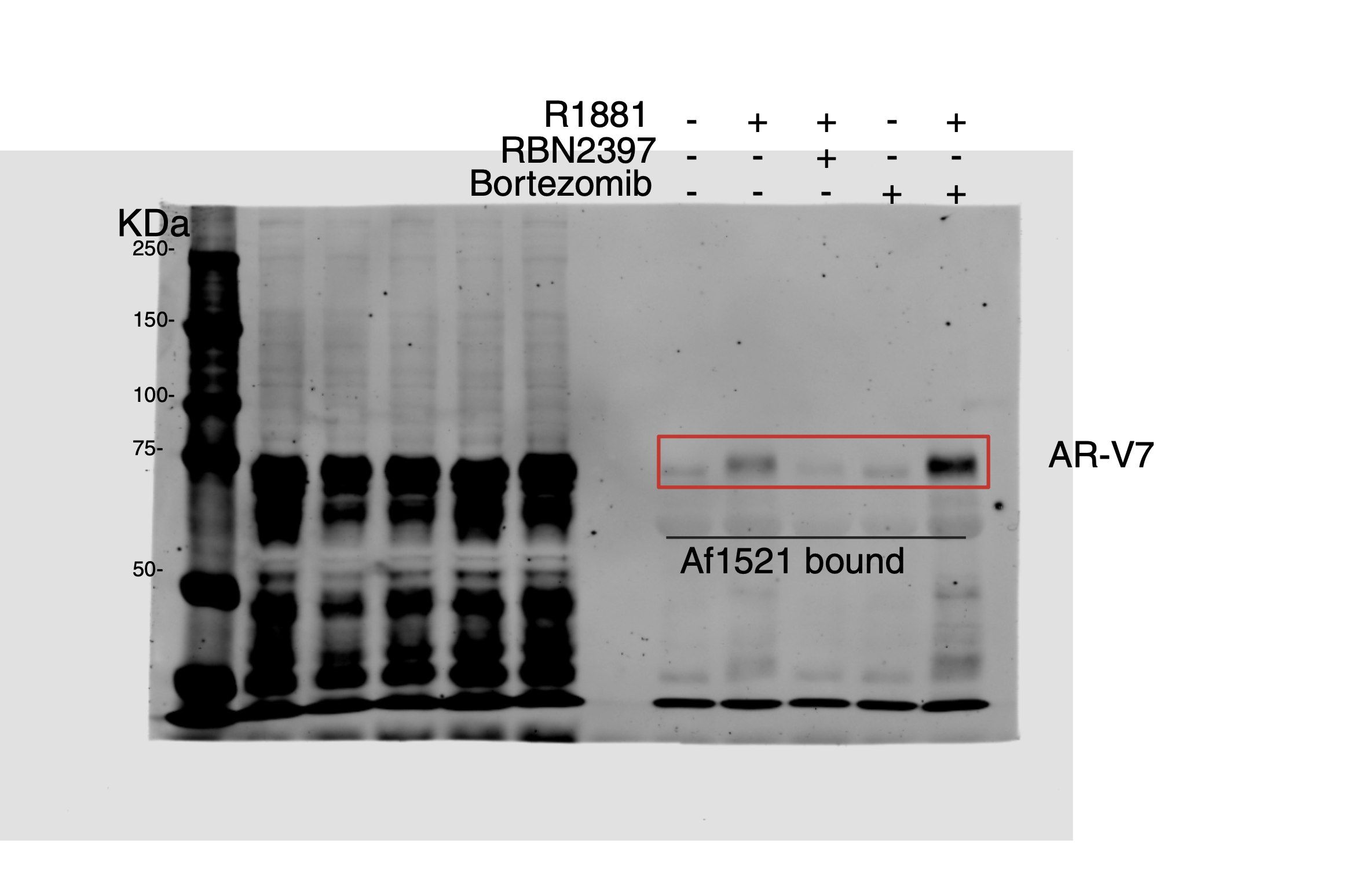

Supplement: Supplementary file 5 — Source data Fig. 3 [file 44318_2025_510_MOESM5_ESM.zip › Figure 3/3G/AR-V7 af1521 bound.png]

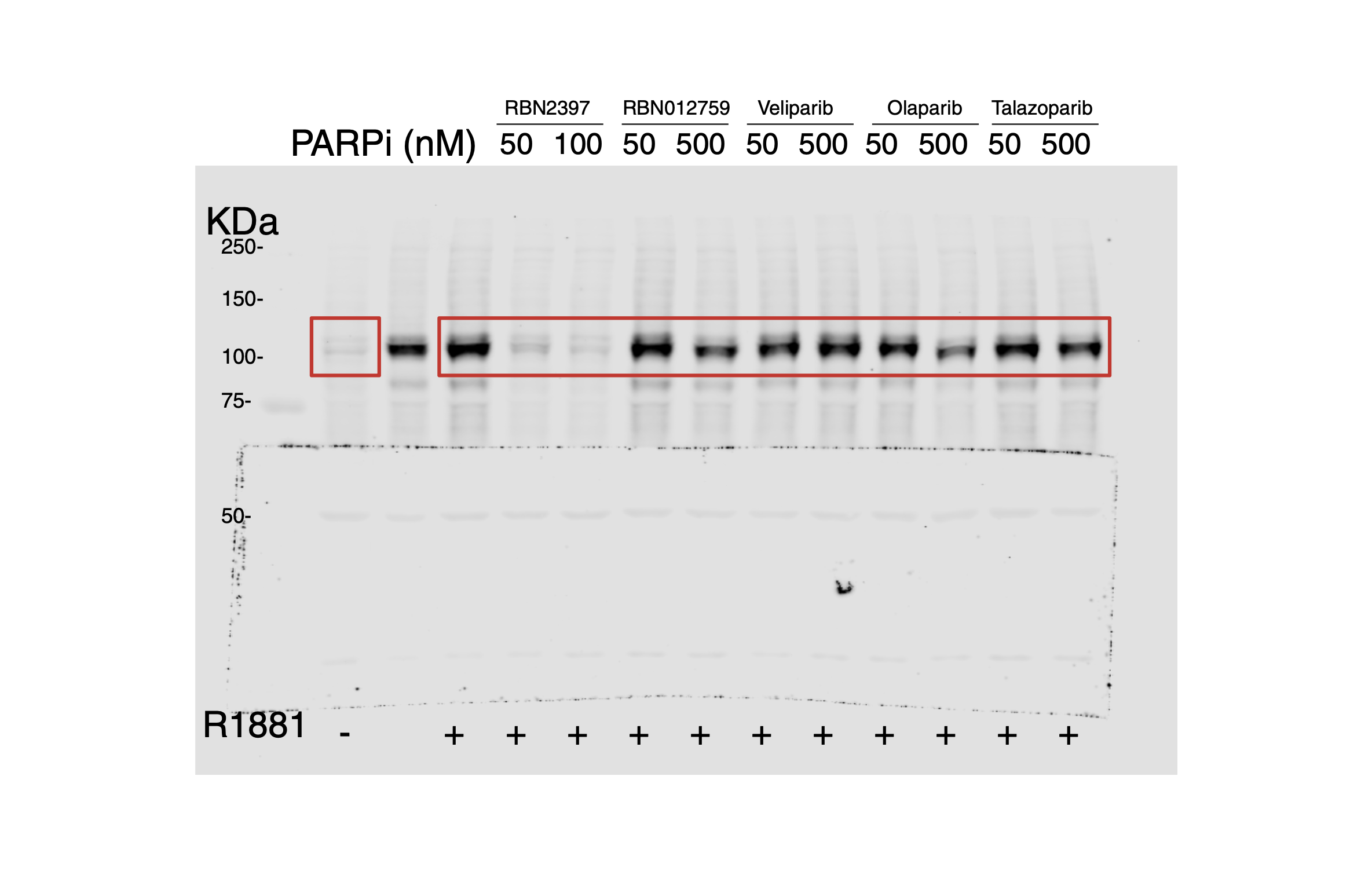

Supplement: Supplementary file 5 — Source data Fig. 3 [file 44318_2025_510_MOESM5_ESM.zip › Figure 3/3I/AR-ADPr.png]

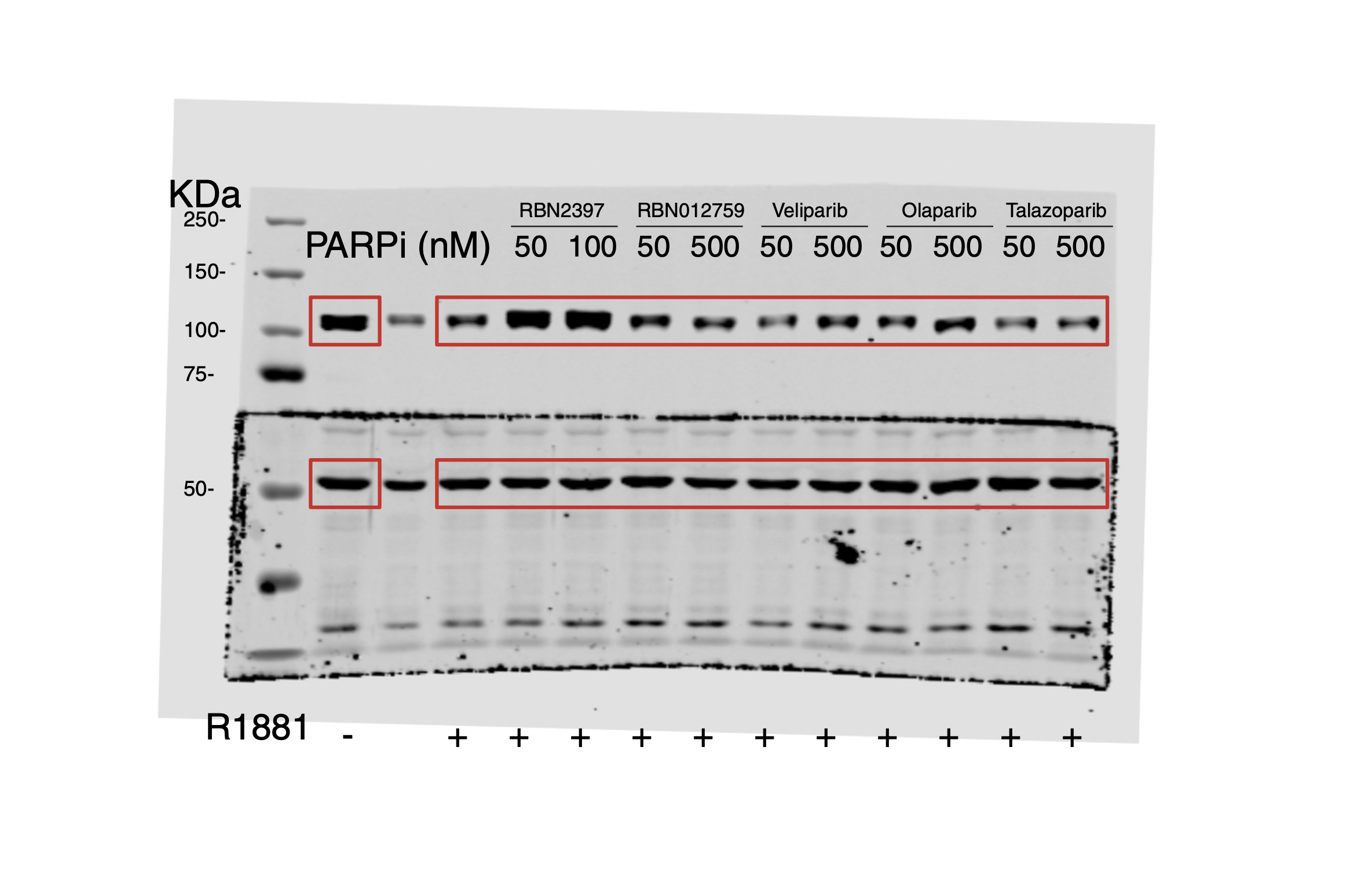

Supplement: Supplementary file 5 — Source data Fig. 3 [file 44318_2025_510_MOESM5_ESM.zip › Figure 3/3I/Flag-AR and TUB.png]

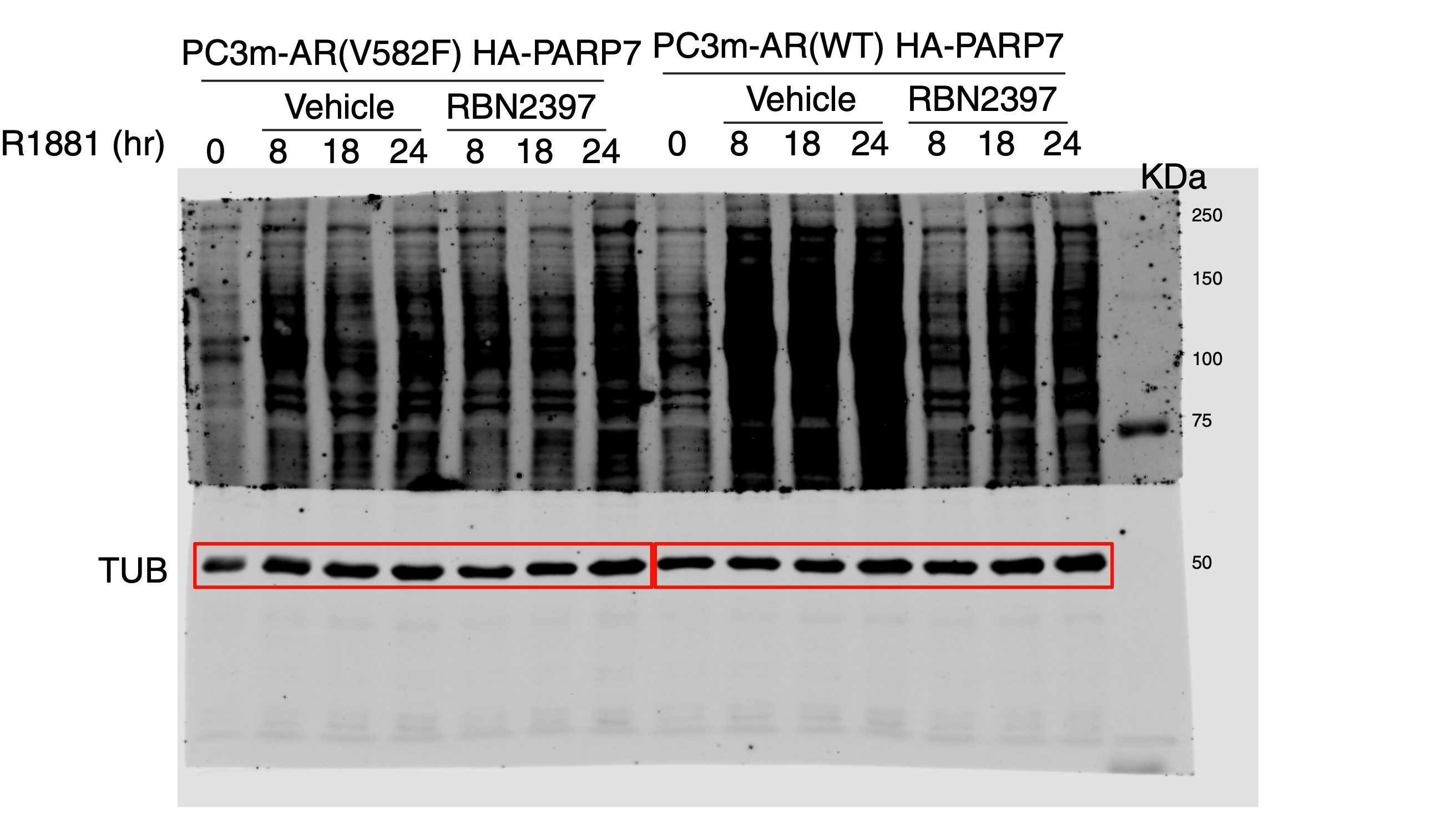

Supplement: Supplementary file 7 — Source data Fig. 5 [file 44318_2025_510_MOESM7_ESM.zip › Figure 5/5B/TUB.png]

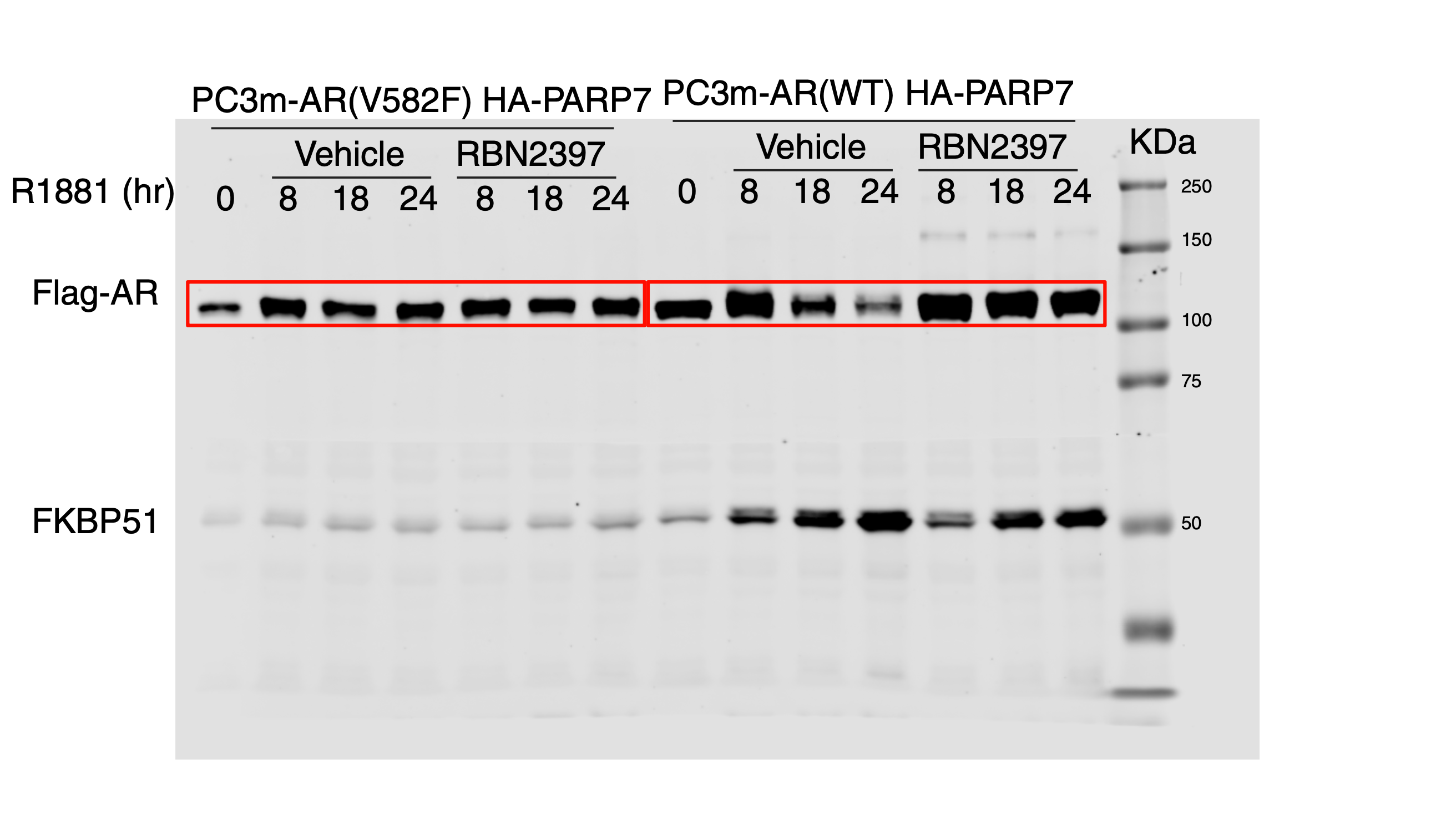

Supplement: Supplementary file 7 — Source data Fig. 5 [file 44318_2025_510_MOESM7_ESM.zip › Figure 5/5B/Flag-AR.png]

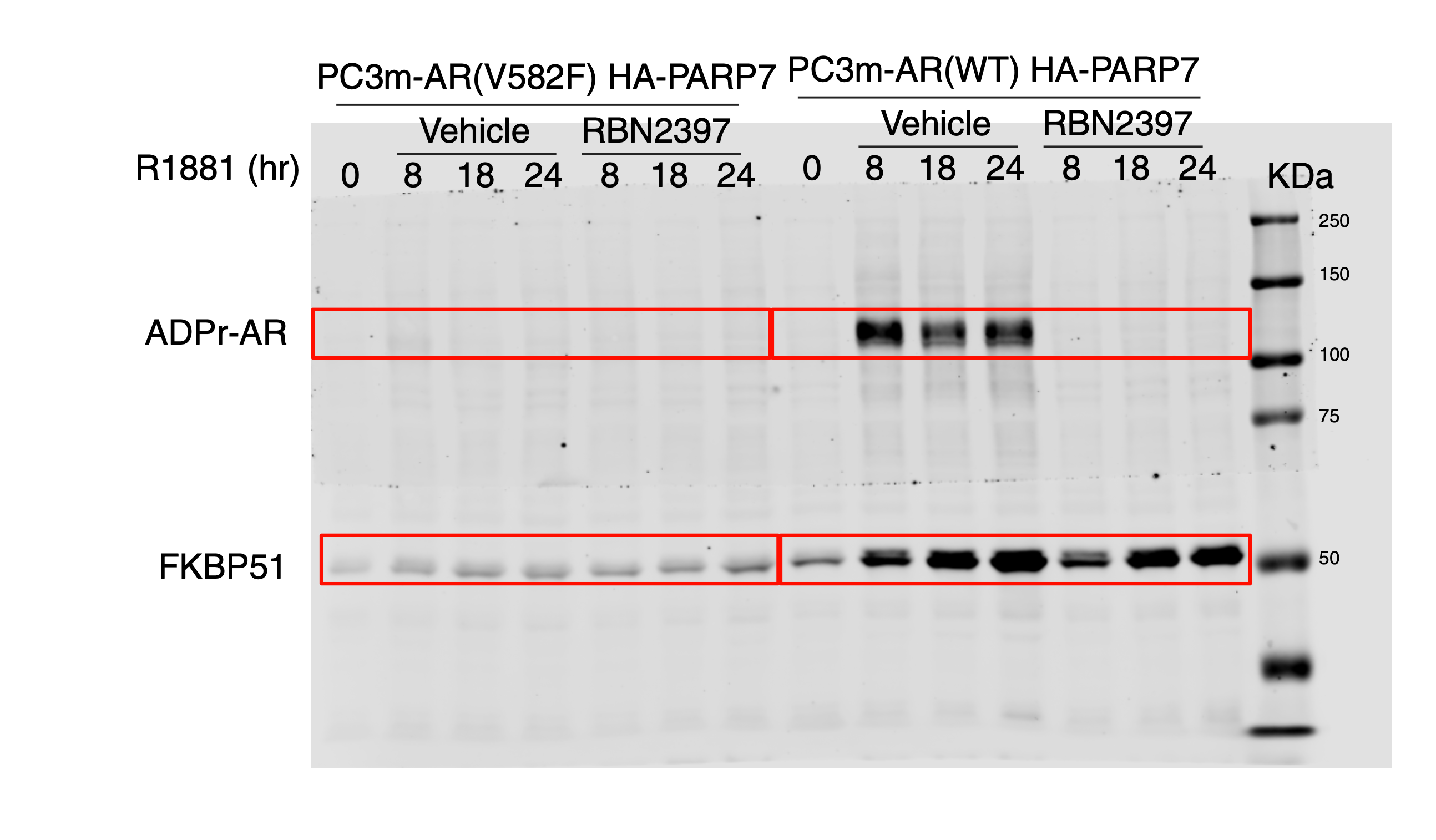

Supplement: Supplementary file 7 — Source data Fig. 5 [file 44318_2025_510_MOESM7_ESM.zip › Figure 5/5B/AR-ADPr and FKBP51.png]

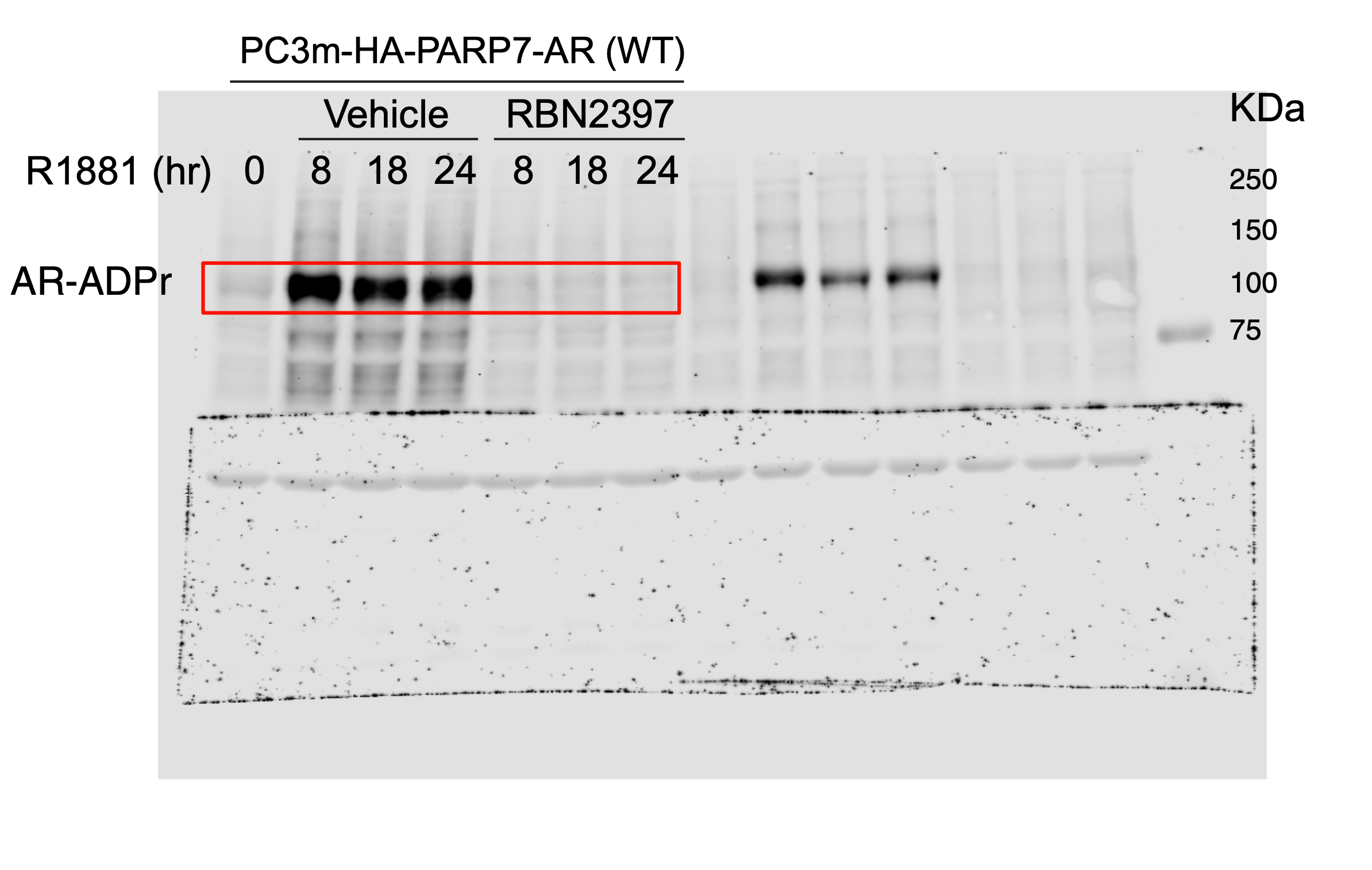

Supplement: Supplementary file 8 — Source data Fig. 6 [file 44318_2025_510_MOESM8_ESM.zip › Figure 6/6A/AR-ADPr.png]

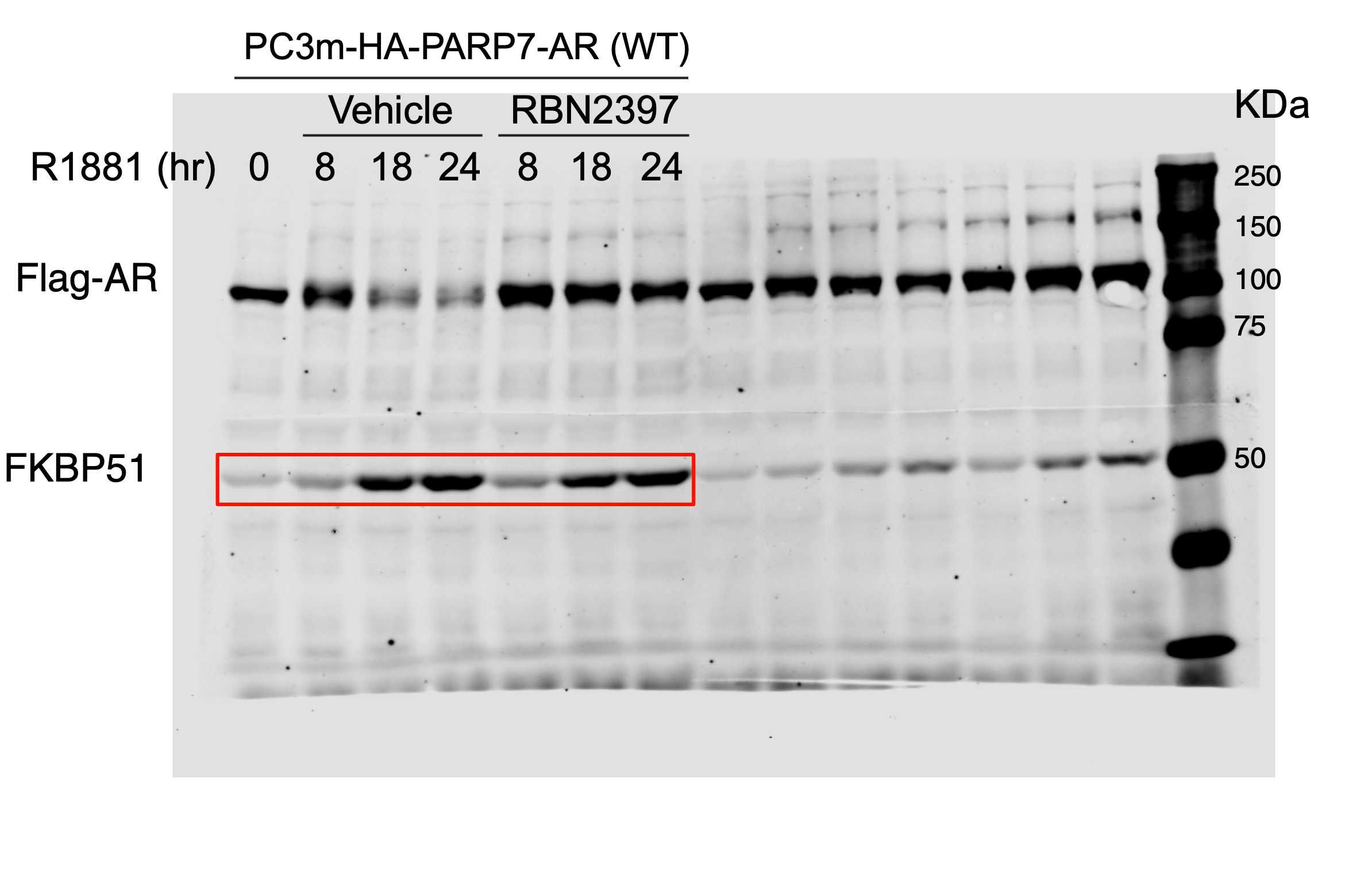

Supplement: Supplementary file 8 — Source data Fig. 6 [file 44318_2025_510_MOESM8_ESM.zip › Figure 6/6A/FKBP51.png]

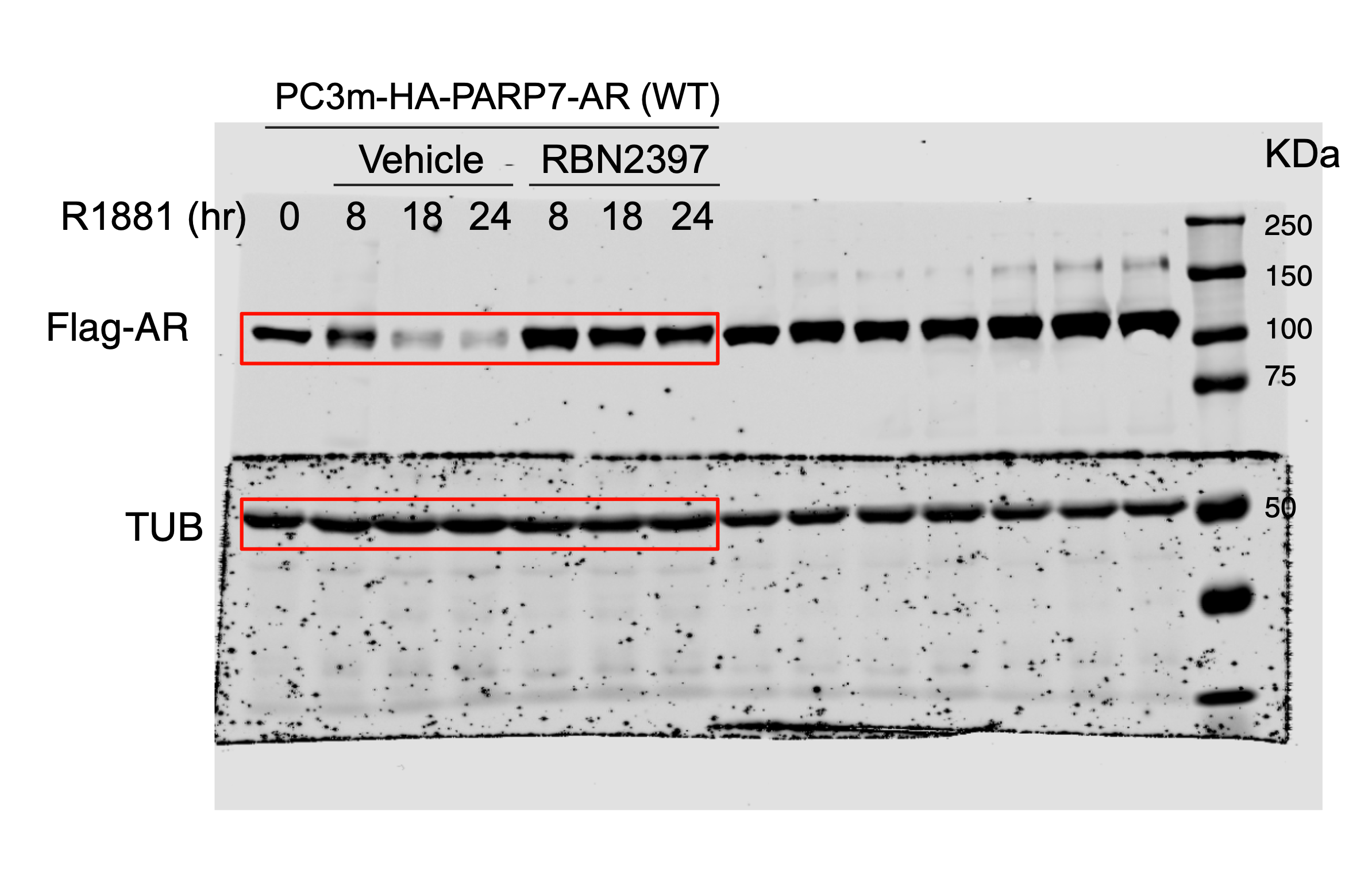

Supplement: Supplementary file 8 — Source data Fig. 6 [file 44318_2025_510_MOESM8_ESM.zip › Figure 6/6A/Flag-AR and TUB.png]

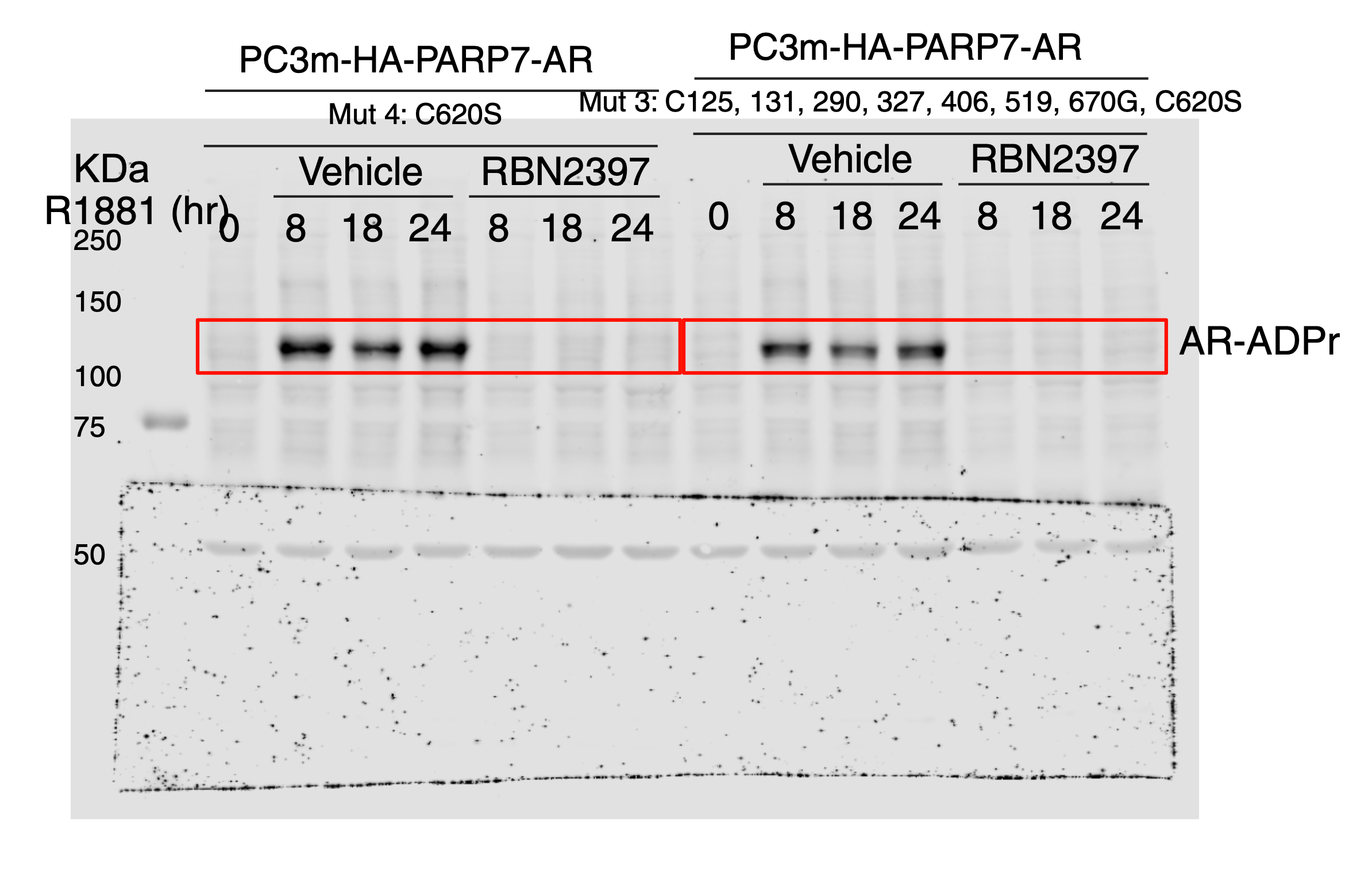

Supplement: Supplementary file 8 — Source data Fig. 6 [file 44318_2025_510_MOESM8_ESM.zip › Figure 6/6D, 6E/AR-ADPr.png]

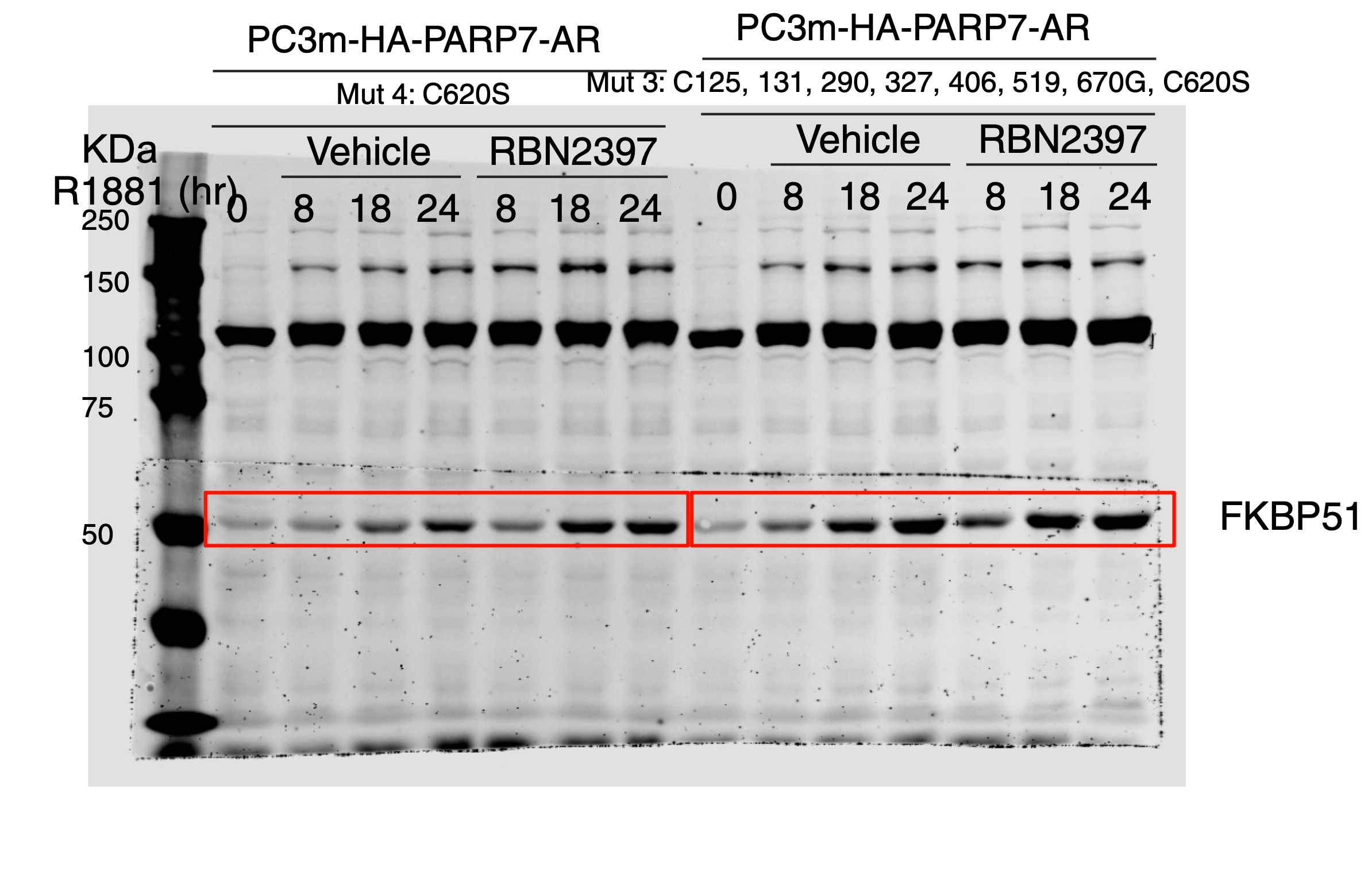

Supplement: Supplementary file 8 — Source data Fig. 6 [file 44318_2025_510_MOESM8_ESM.zip › Figure 6/6D, 6E/FKBP51.png]

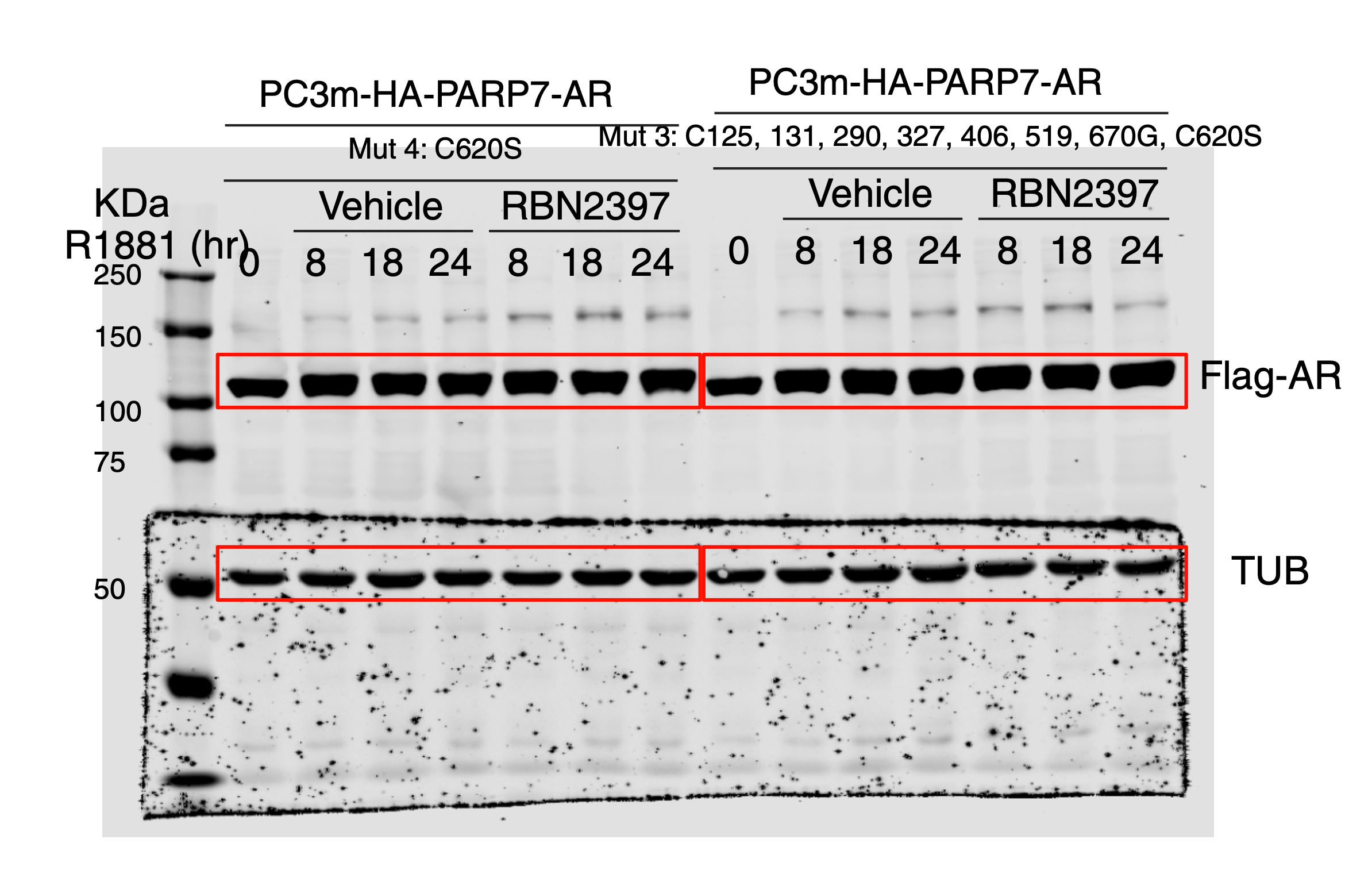

Supplement: Supplementary file 8 — Source data Fig. 6 [file 44318_2025_510_MOESM8_ESM.zip › Figure 6/6D, 6E/Flag-AR and TUB.png]

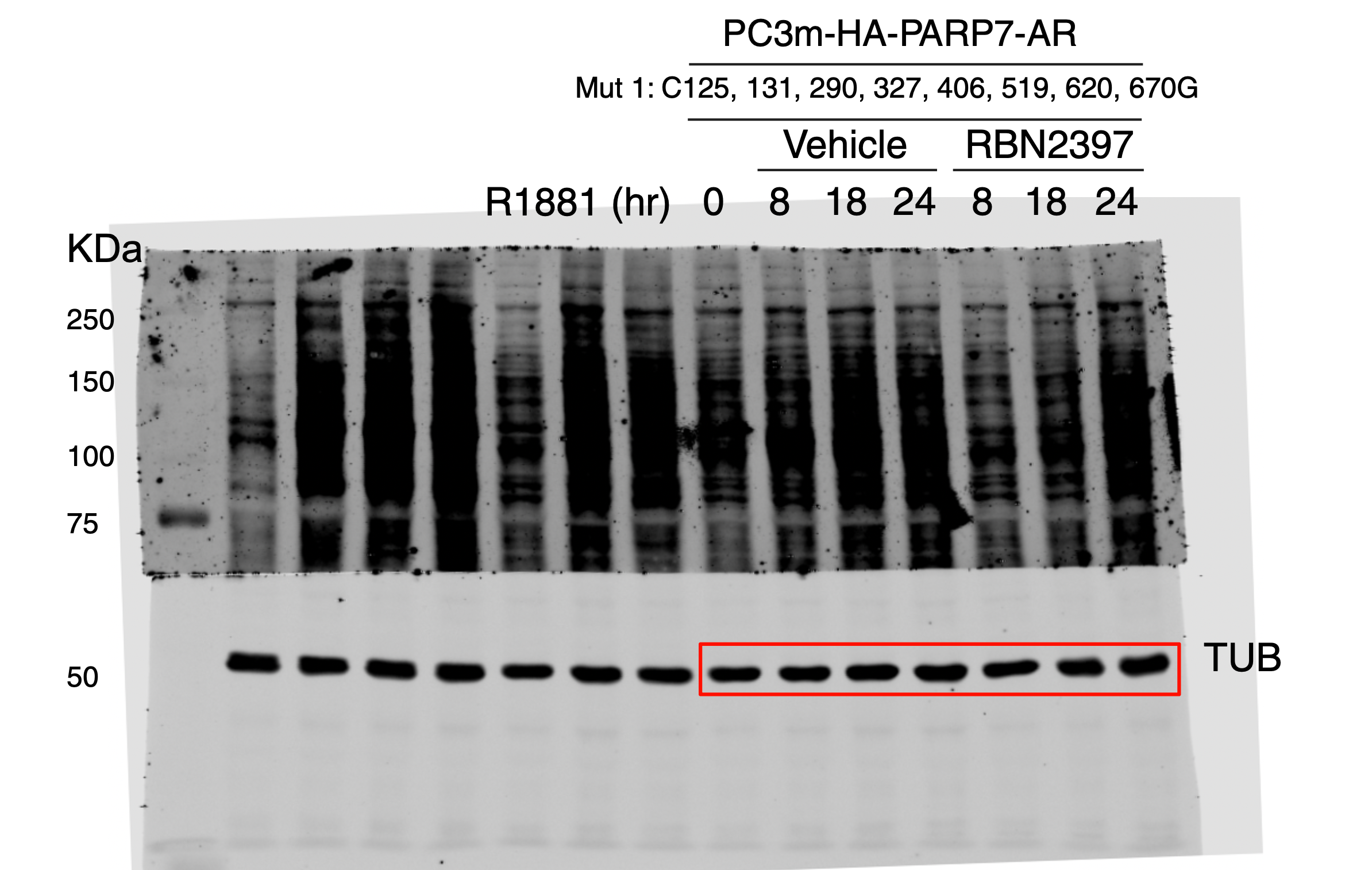

Supplement: Supplementary file 8 — Source data Fig. 6 [file 44318_2025_510_MOESM8_ESM.zip › Figure 6/6B/TUB.png]

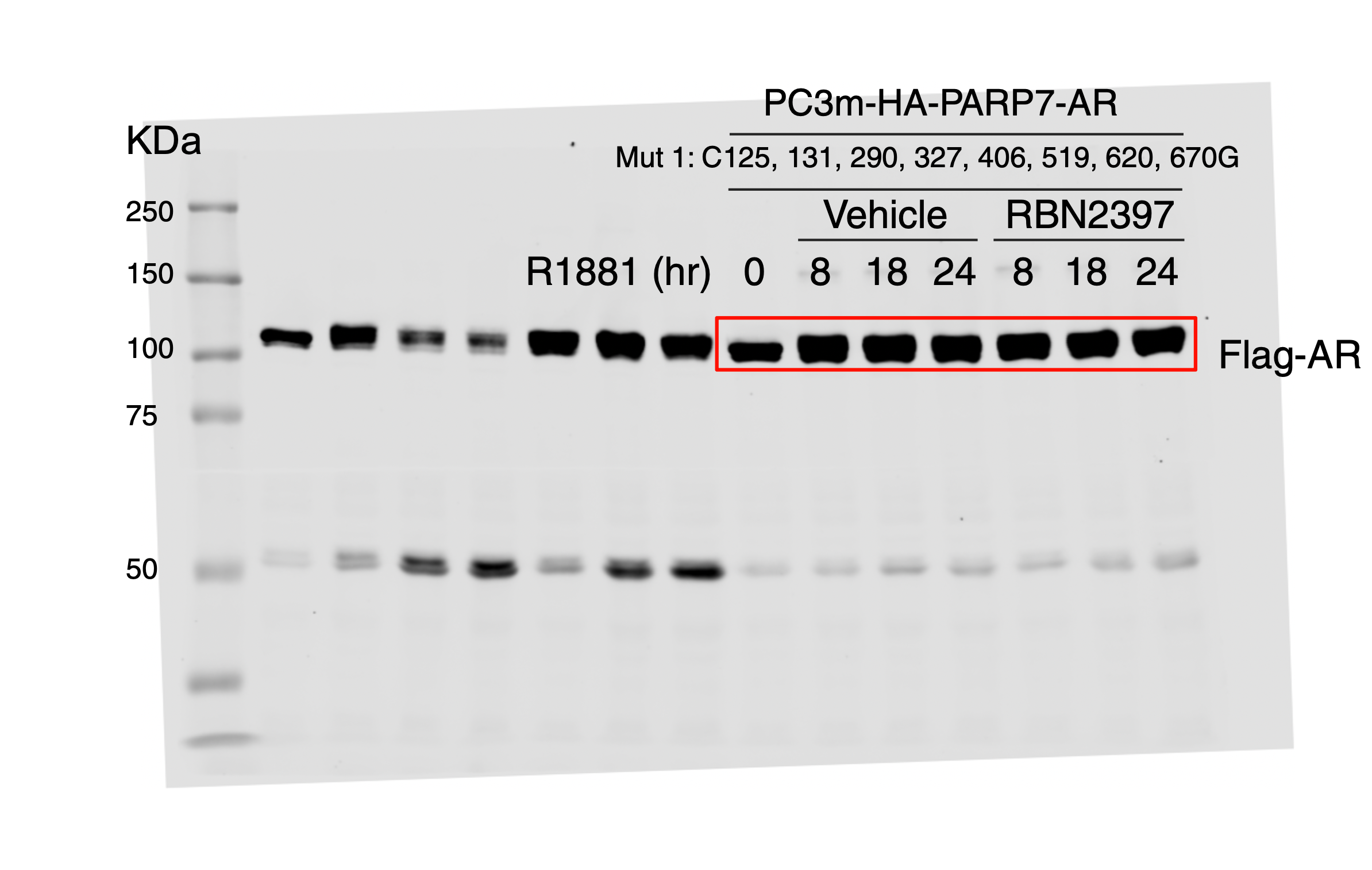

Supplement: Supplementary file 8 — Source data Fig. 6 [file 44318_2025_510_MOESM8_ESM.zip › Figure 6/6B/Flag-AR.png]

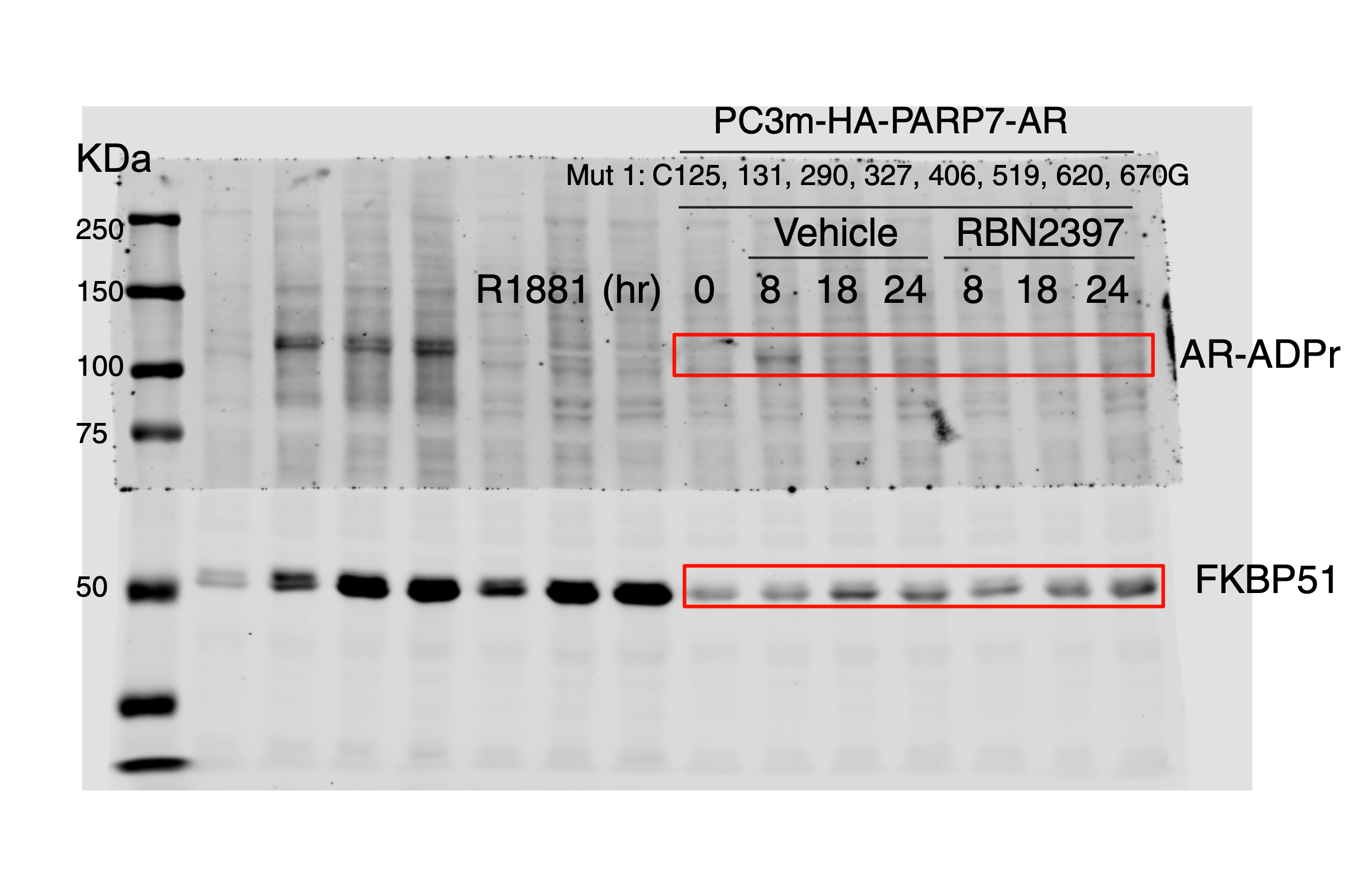

Supplement: Supplementary file 8 — Source data Fig. 6 [file 44318_2025_510_MOESM8_ESM.zip › Figure 6/6B/AR-ADPr and FKBP51.png]

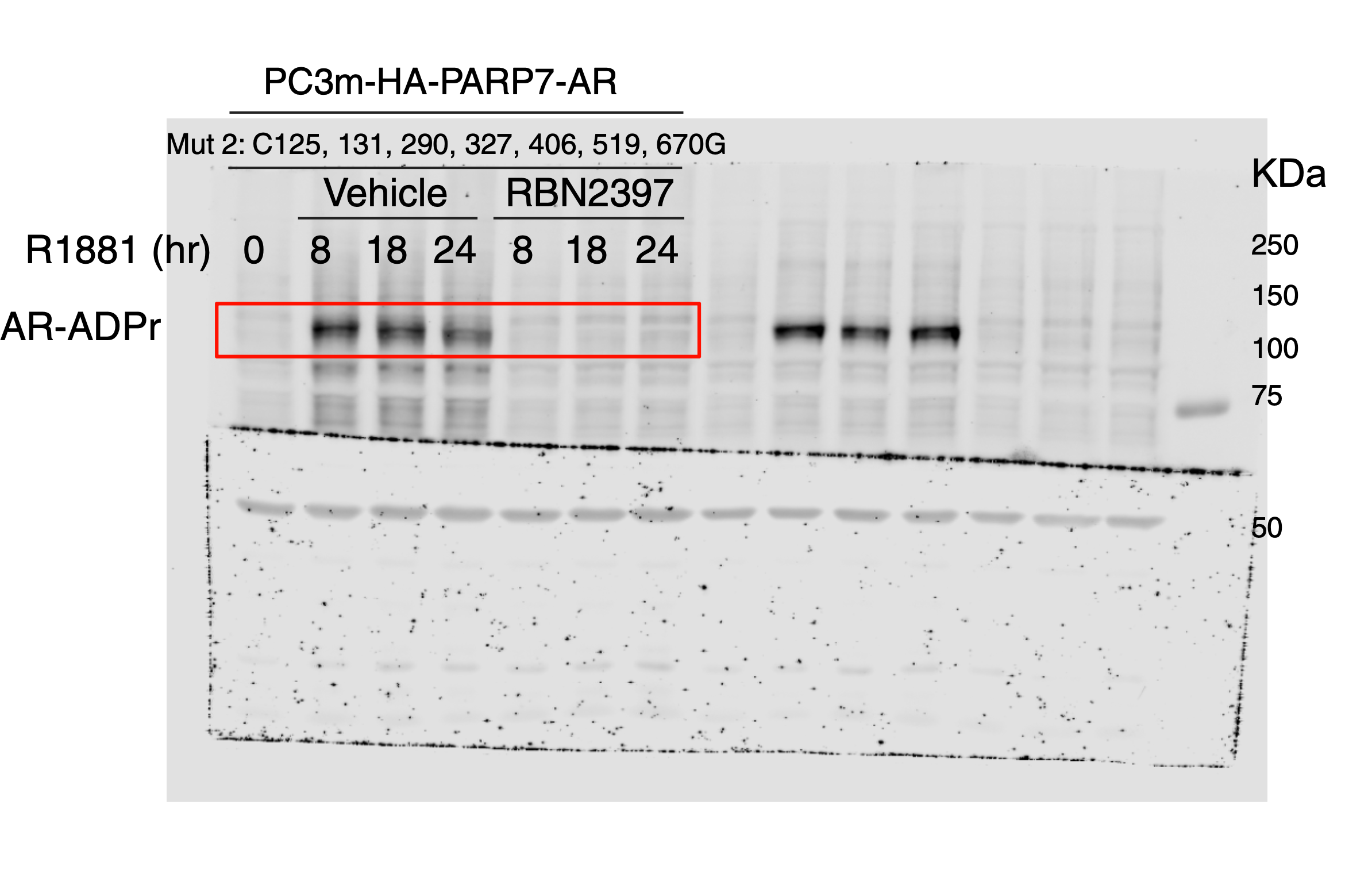

Supplement: Supplementary file 8 — Source data Fig. 6 [file 44318_2025_510_MOESM8_ESM.zip › Figure 6/6C/AR-ADPr.png]

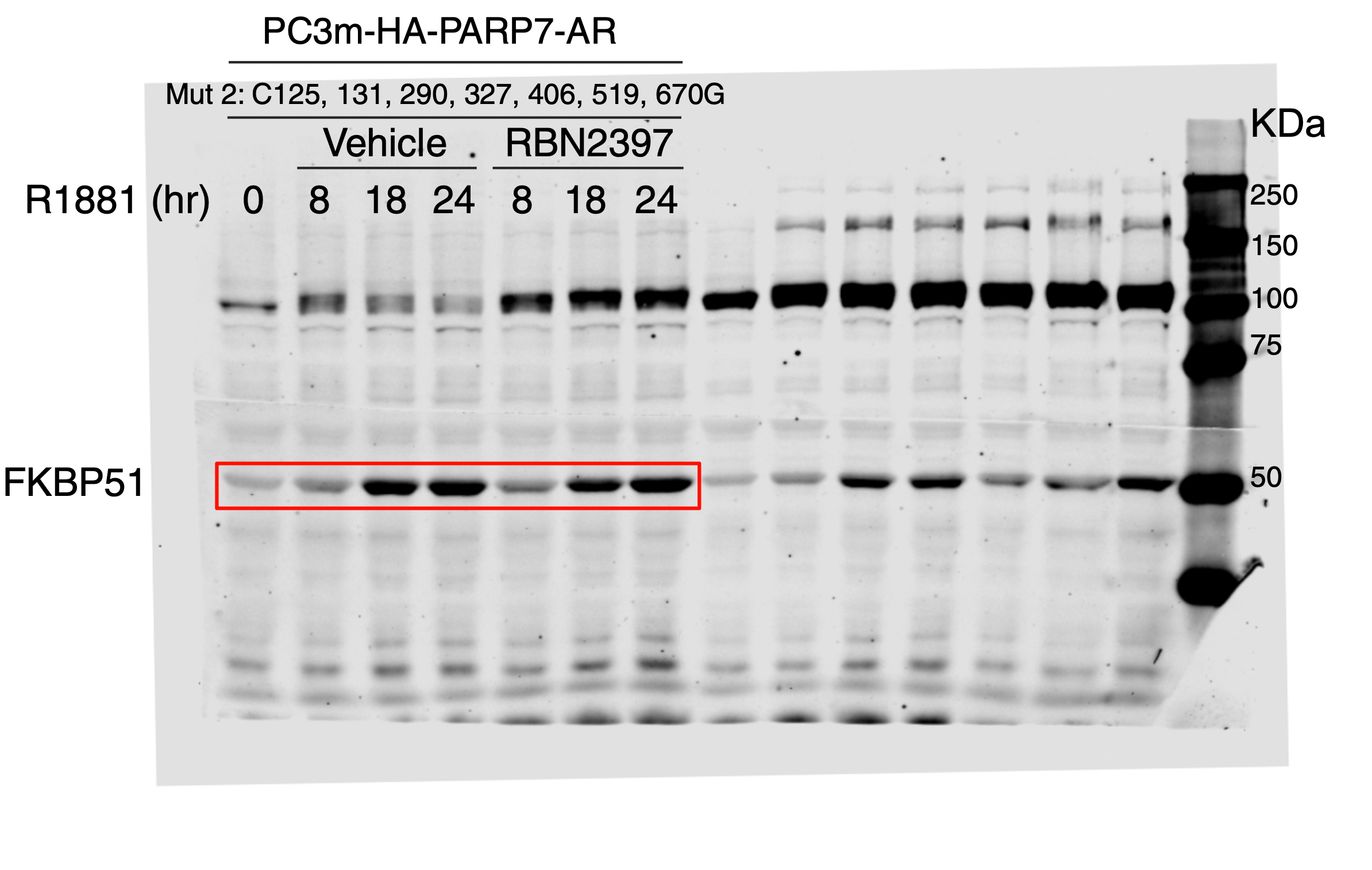

Supplement: Supplementary file 8 — Source data Fig. 6 [file 44318_2025_510_MOESM8_ESM.zip › Figure 6/6C/FKBP51.png]

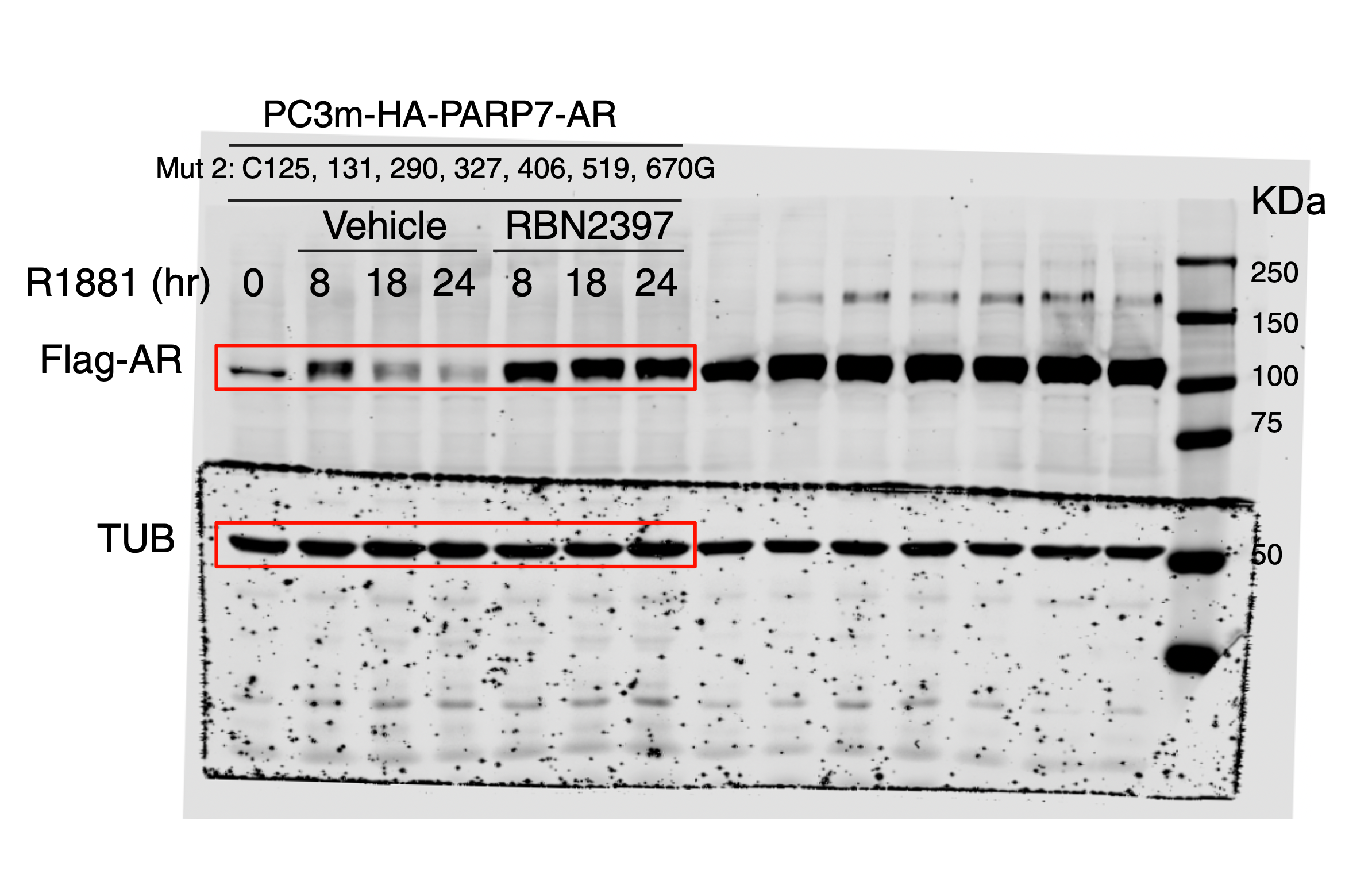

Supplement: Supplementary file 8 — Source data Fig. 6 [file 44318_2025_510_MOESM8_ESM.zip › Figure 6/6C/Flag-AR and TUB.png]

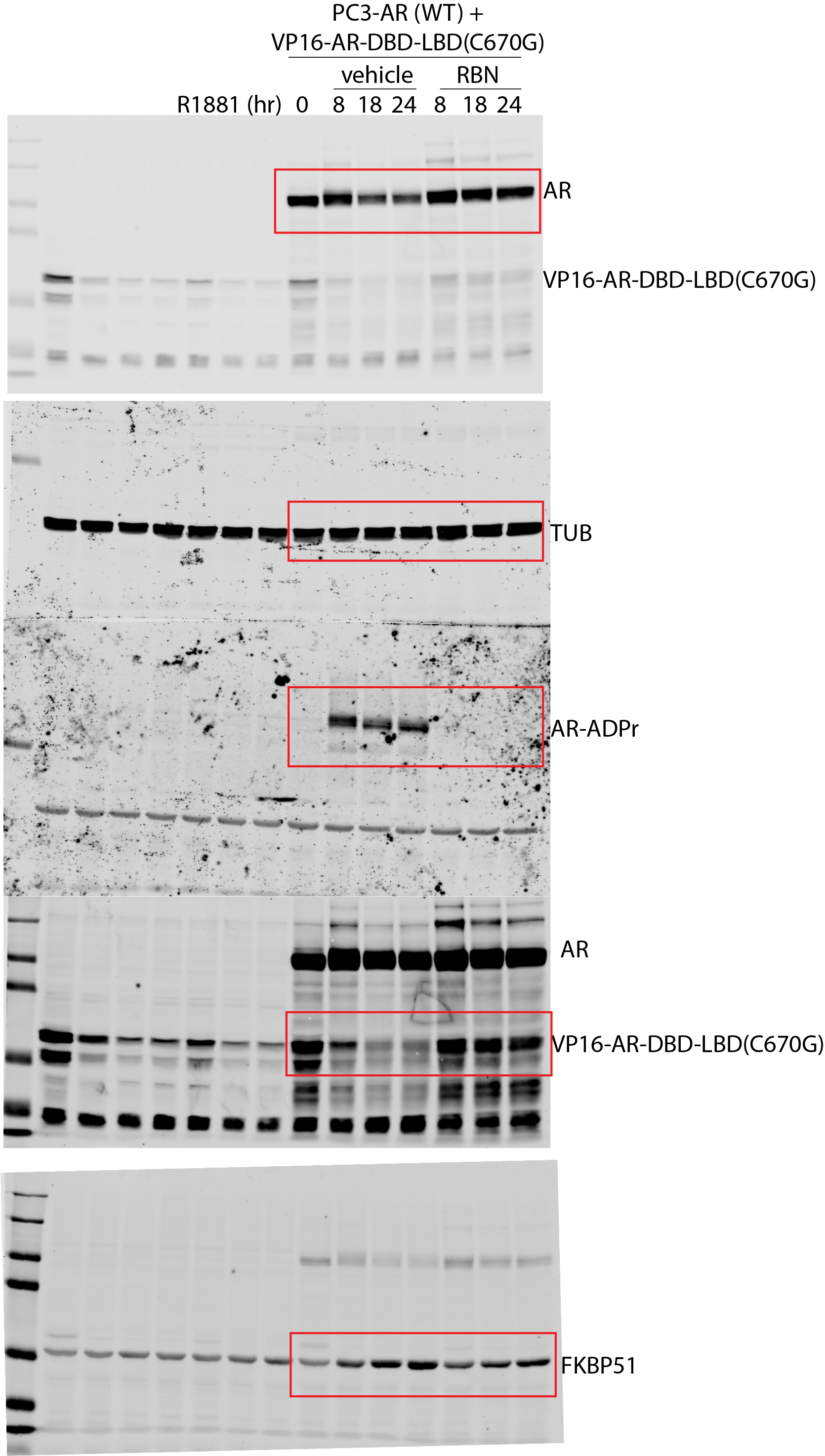

Supplement: Supplementary file 8 — Source data Fig. 6 [file 44318_2025_510_MOESM8_ESM.zip › Figure 6/6H/AR,TUB,AR-ADPr,VP16-AR-DBD-LBD, FKBP51.png]

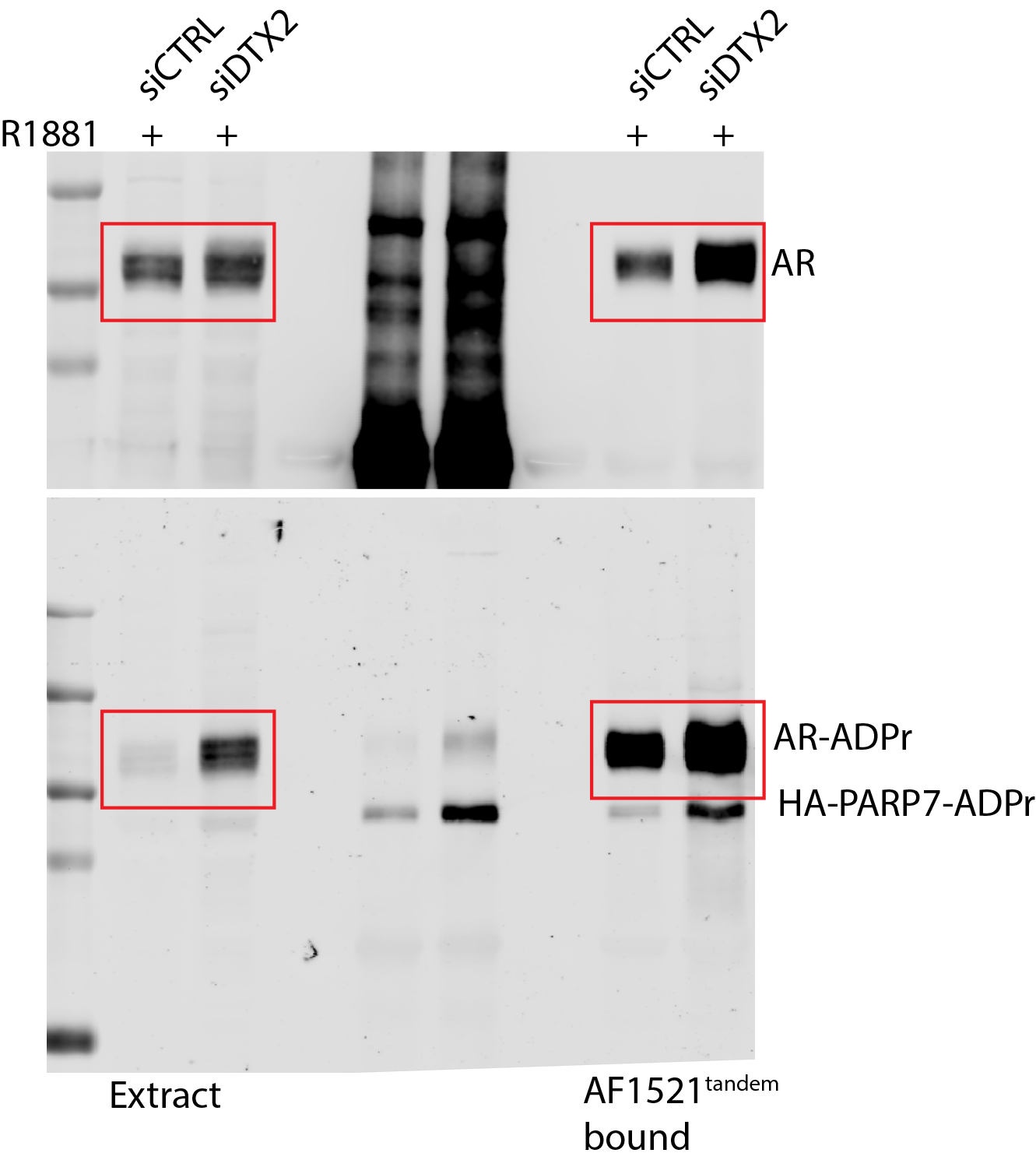

Supplement: Supplementary file 9 — Source data Fig. 7 [file 44318_2025_510_MOESM9_ESM.zip › Figure 7/7C/AR, AR-ADPr.png]

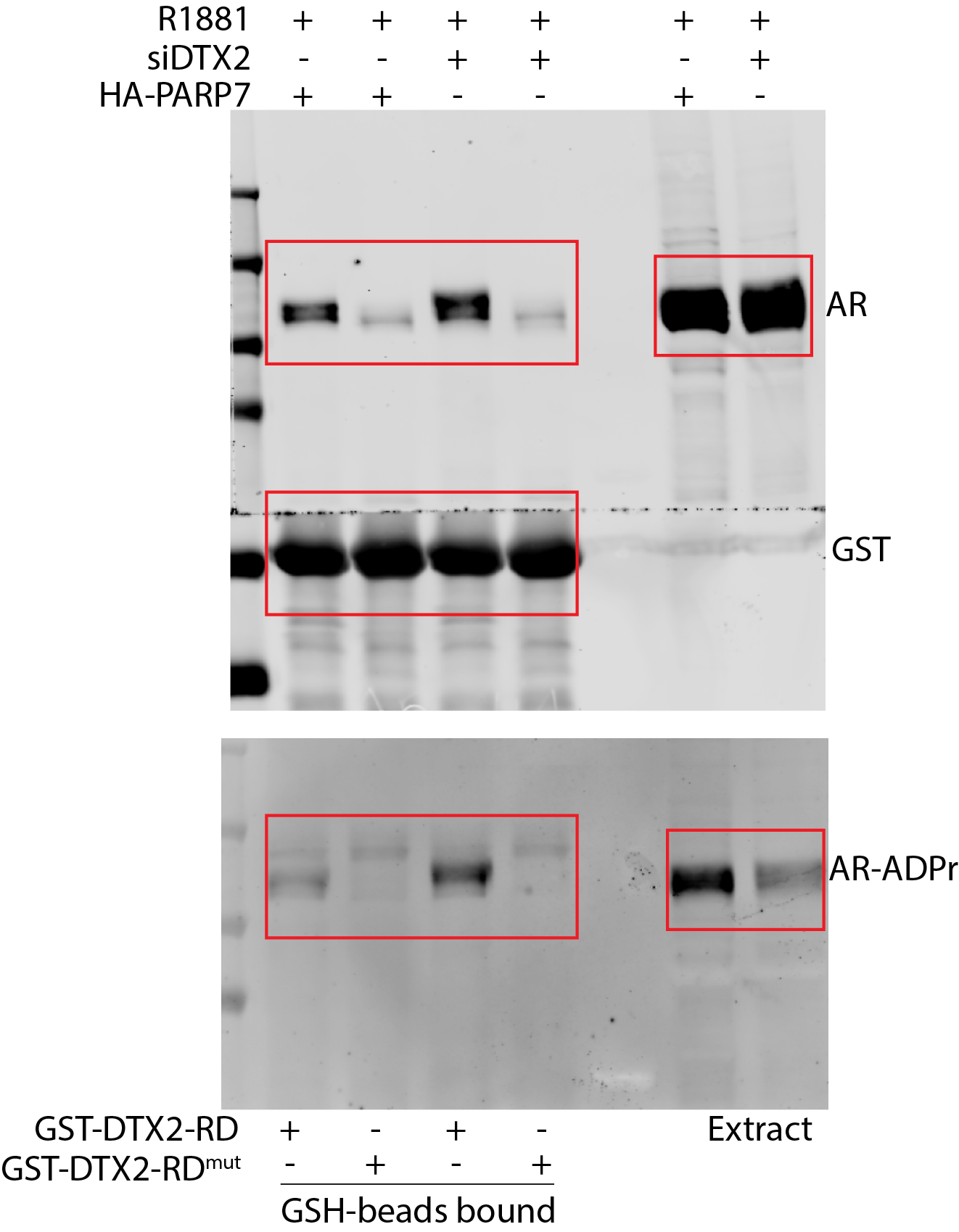

Supplement: Supplementary file 9 — Source data Fig. 7 [file 44318_2025_510_MOESM9_ESM.zip › Figure 7/7D/AR, AR-ADPr, GST.png]

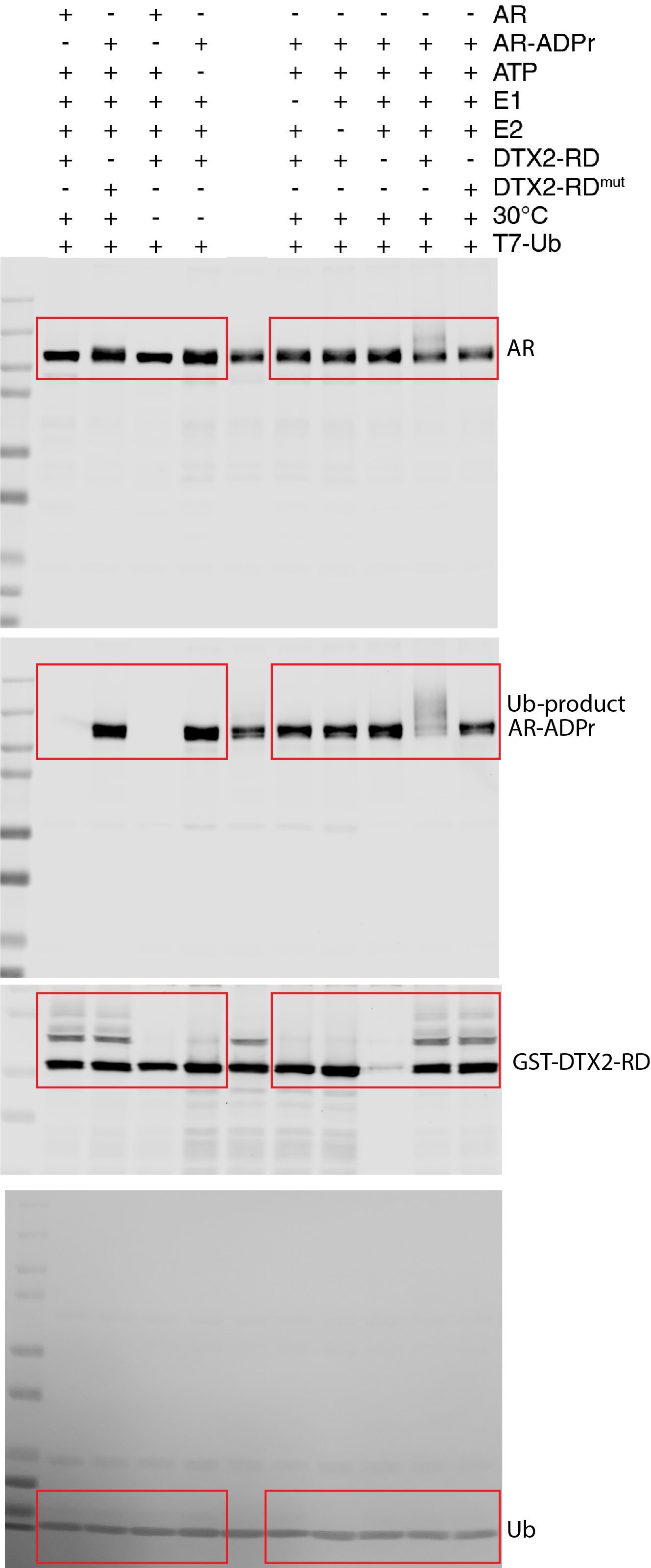

Supplement: Supplementary file 9 — Source data Fig. 7 [file 44318_2025_510_MOESM9_ESM.zip › Figure 7/7H/AR, ADPr-AR&Ub-product, GST-DTX2-RD, Ub.png]

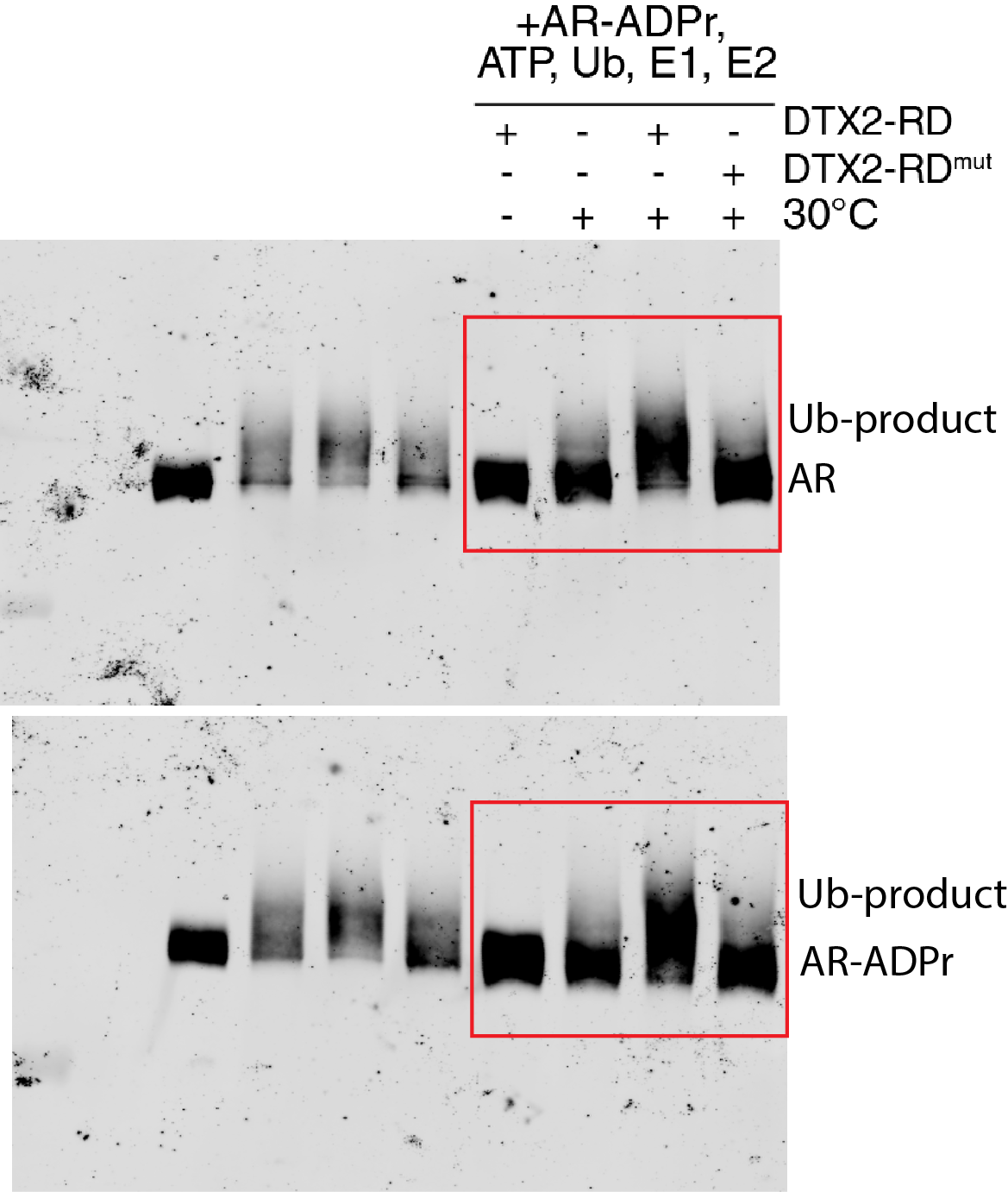

Supplement: Supplementary file 9 — Source data Fig. 7 [file 44318_2025_510_MOESM9_ESM.zip › Figure 7/7G/AR&Ub-product, AR-ADPr&Ub-product.png]

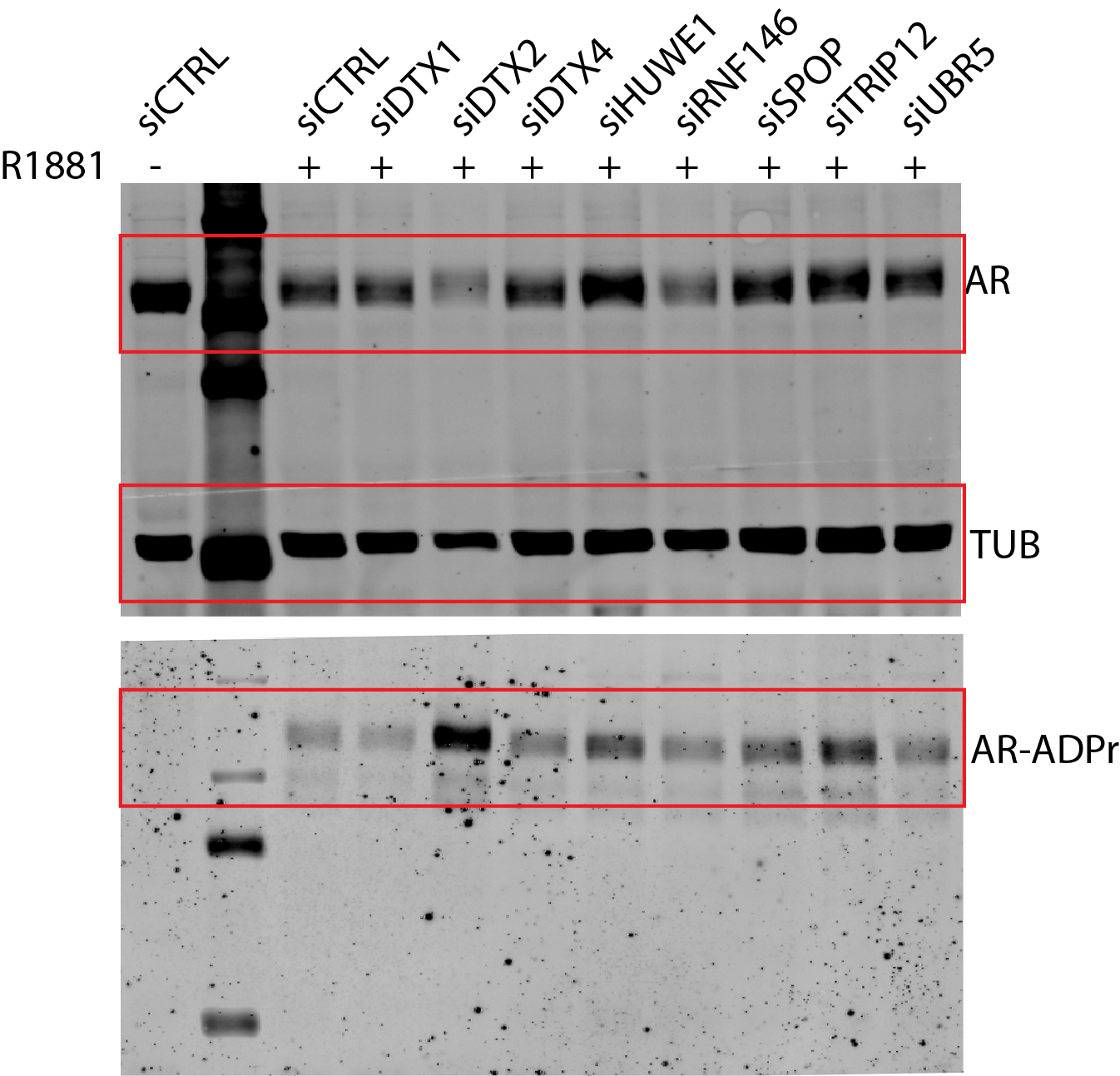

Supplement: Supplementary file 9 — Source data Fig. 7 [file 44318_2025_510_MOESM9_ESM.zip › Figure 7/7A/AR, AR-ADPr, TUB.png]

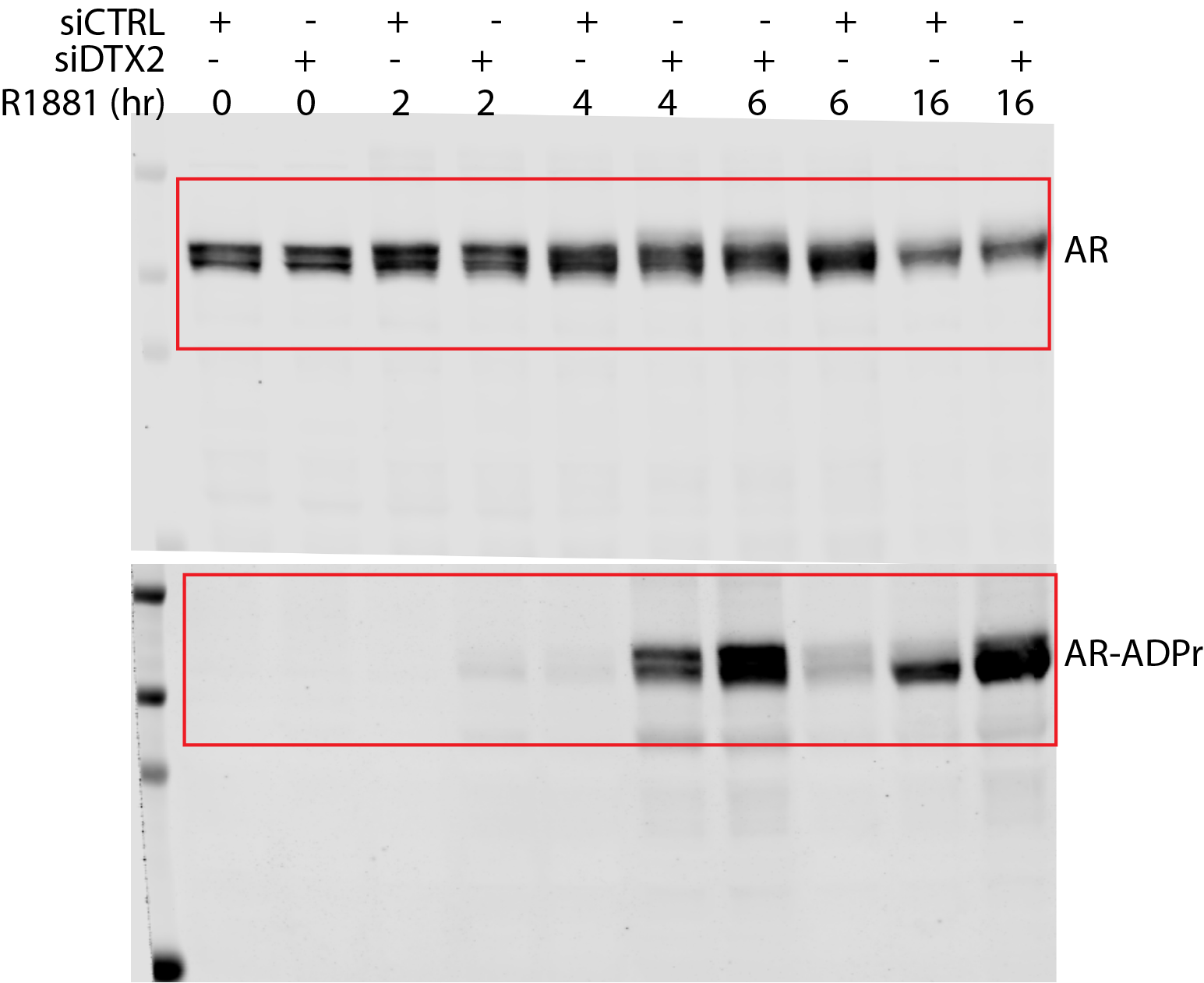

Supplement: Supplementary file 9 — Source data Fig. 7 [file 44318_2025_510_MOESM9_ESM.zip › Figure 7/7B/AR, AR-ADPr.png]

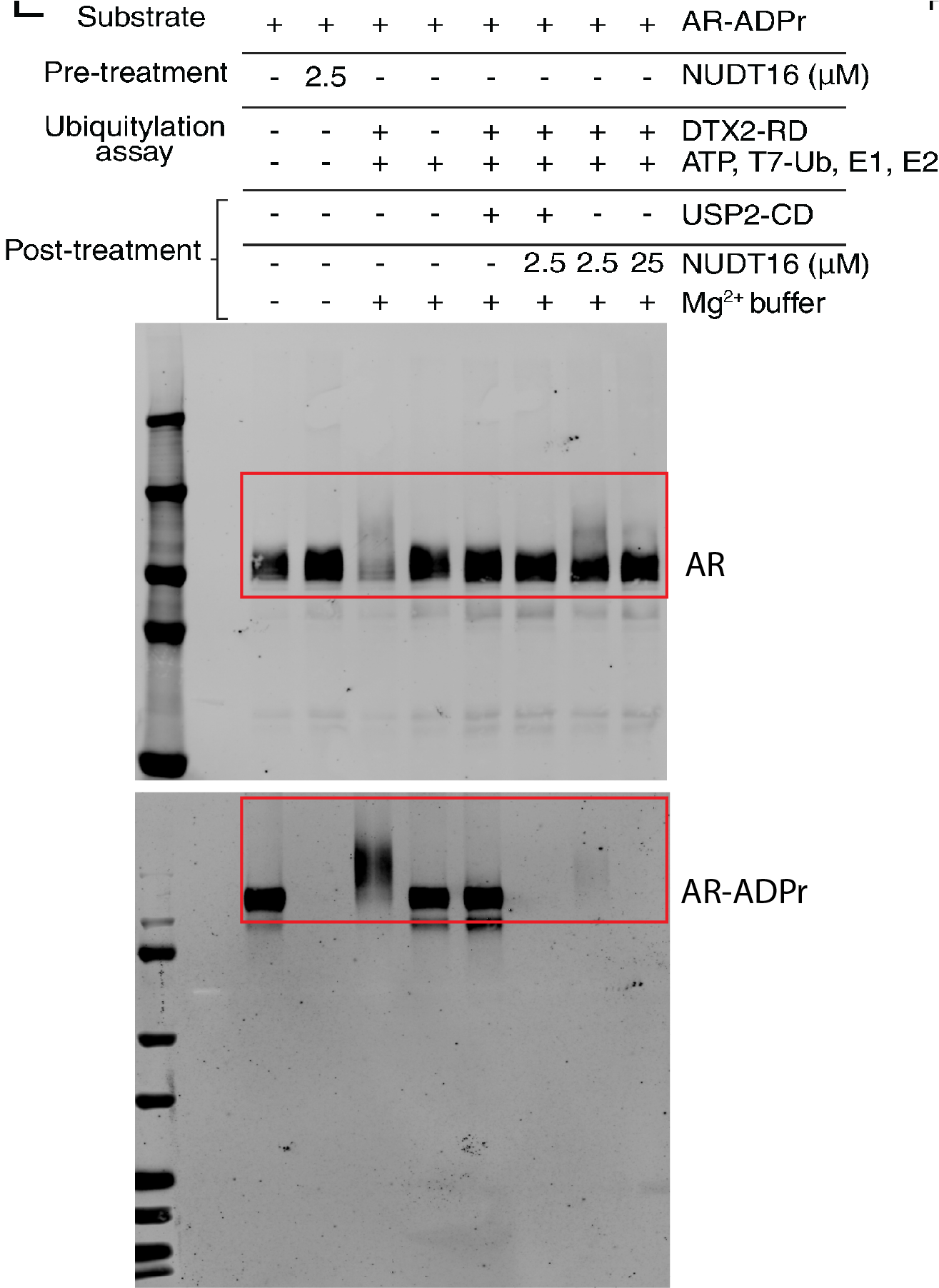

Supplement: Supplementary file 10 — Source data Fig. 8 [file 44318_2025_510_MOESM10_ESM.zip › Figure 8/8E/AR&Ub-product, AR-ADPr&Ub-product.png]

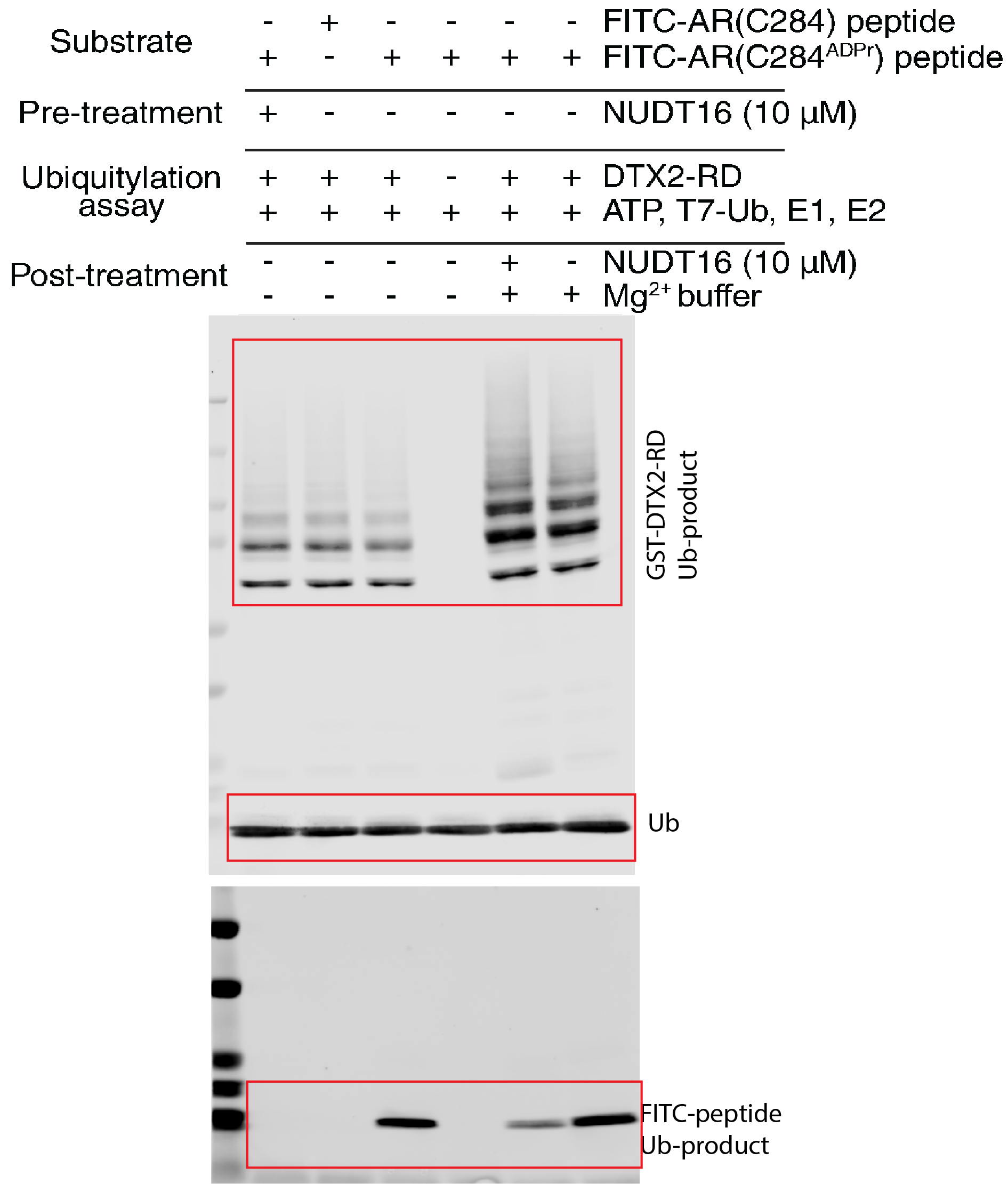

Supplement: Supplementary file 10 — Source data Fig. 8 [file 44318_2025_510_MOESM10_ESM.zip › Figure 8/8A/GST-DTX2-RD&Ub-product, Ub, FITC-peptide Ub-product.png]

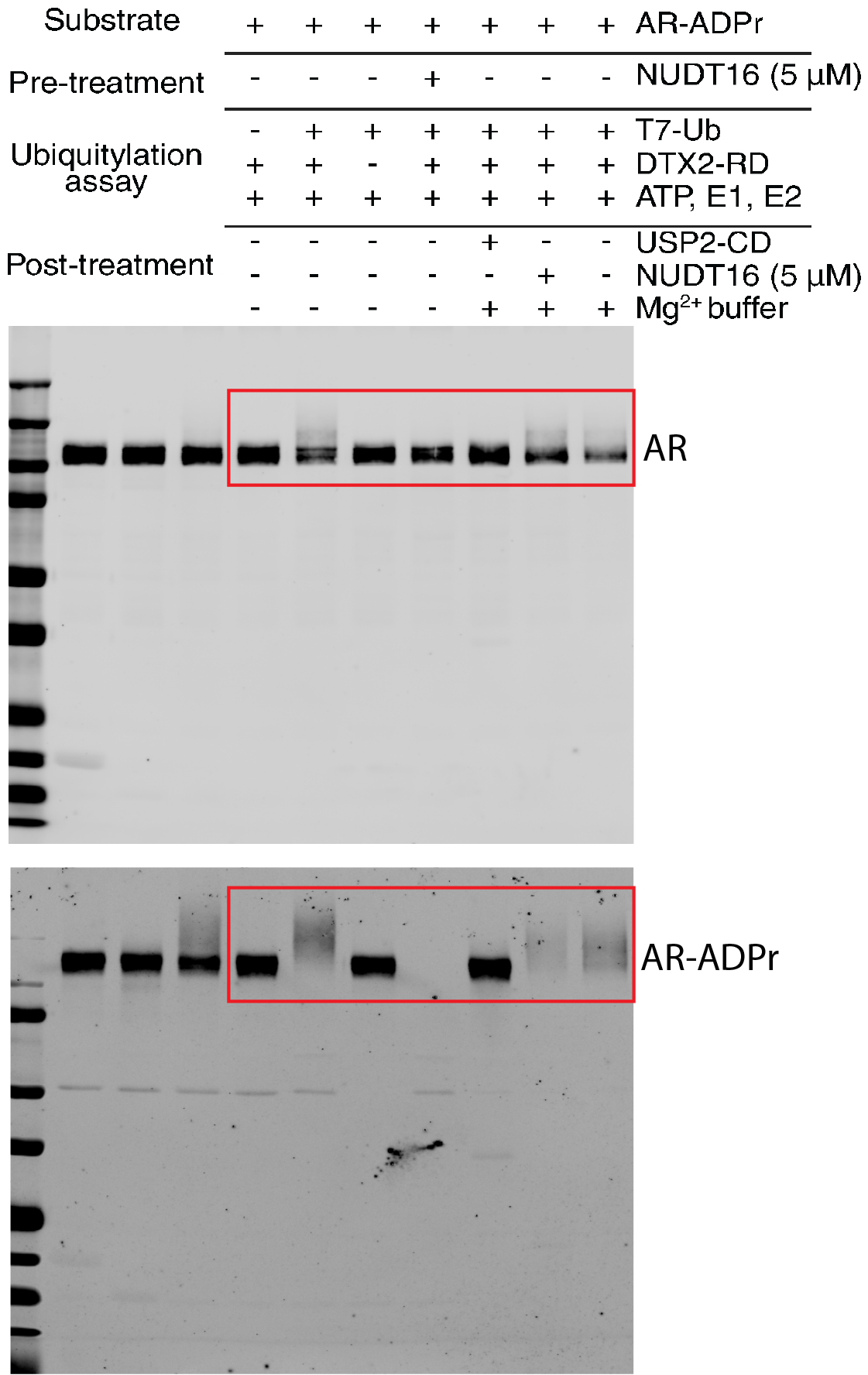

Supplement: Supplementary file 10 — Source data Fig. 8 [file 44318_2025_510_MOESM10_ESM.zip › Figure 8/8D/AR&Ub-product, AR-ADPr&Ub-product.png]

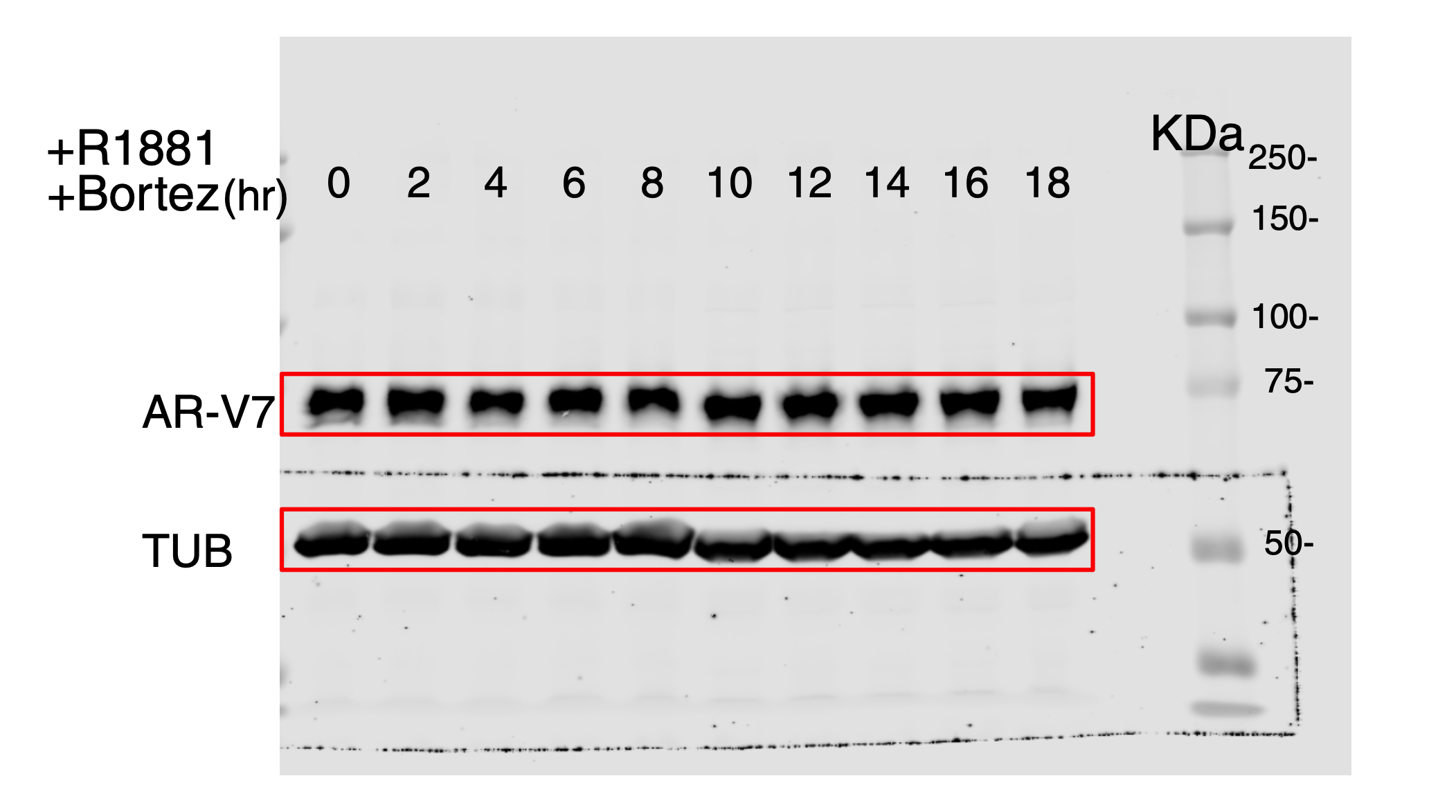

Supplement: Supplementary file 11 — Appendix Fig 1-6 Source Data [file 44318_2025_510_MOESM11_ESM.zip › Appendix Fig S3/S3C/AR-V7 and TUB.png]

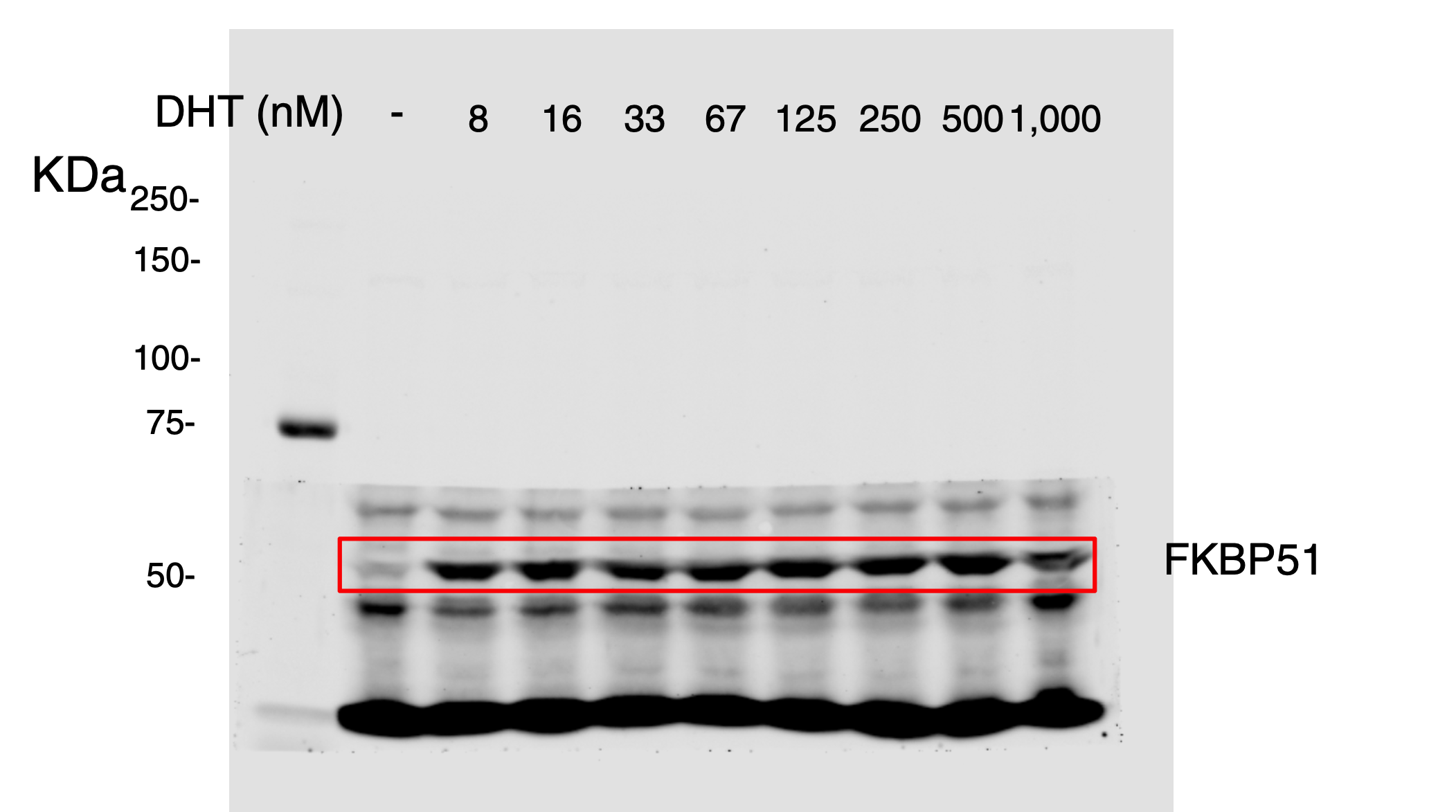

Supplement: Supplementary file 11 — Appendix Fig 1-6 Source Data [file 44318_2025_510_MOESM11_ESM.zip › Appendix Fig S3/S3E/FKBP51.png]

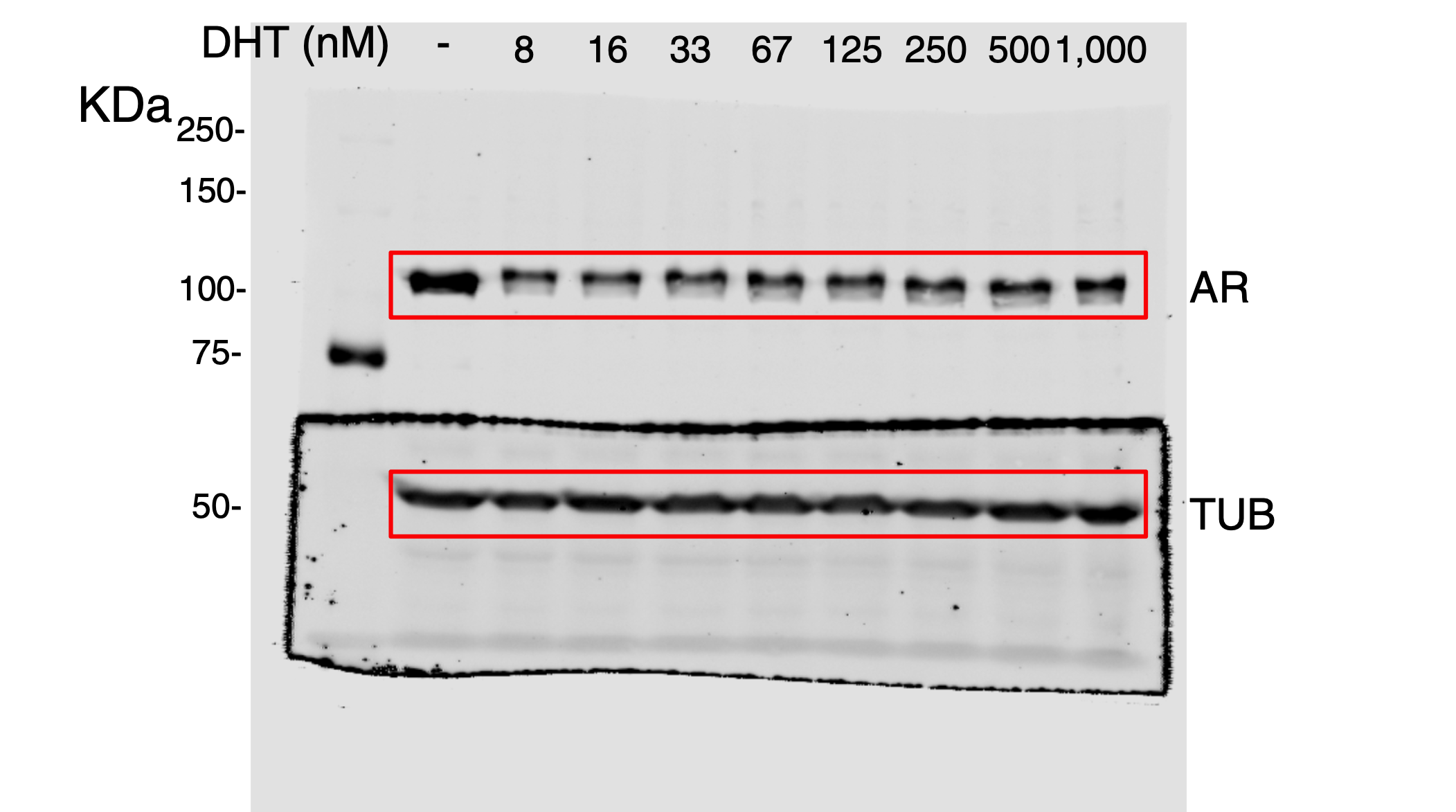

Supplement: Supplementary file 11 — Appendix Fig 1-6 Source Data [file 44318_2025_510_MOESM11_ESM.zip › Appendix Fig S3/S3E/AR and TUB.png]

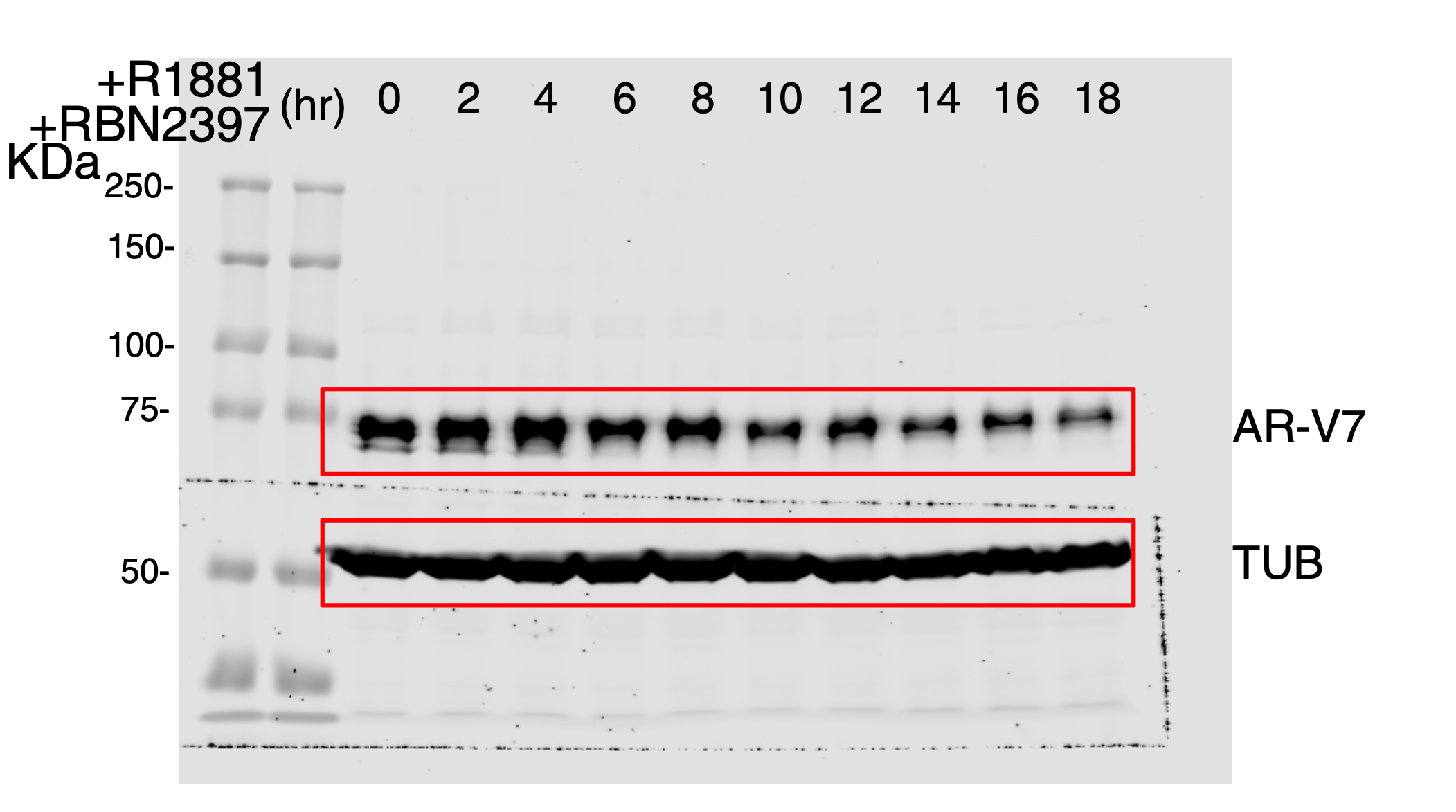

Supplement: Supplementary file 11 — Appendix Fig 1-6 Source Data [file 44318_2025_510_MOESM11_ESM.zip › Appendix Fig S3/S3B/AR-V7 and TUB.png]

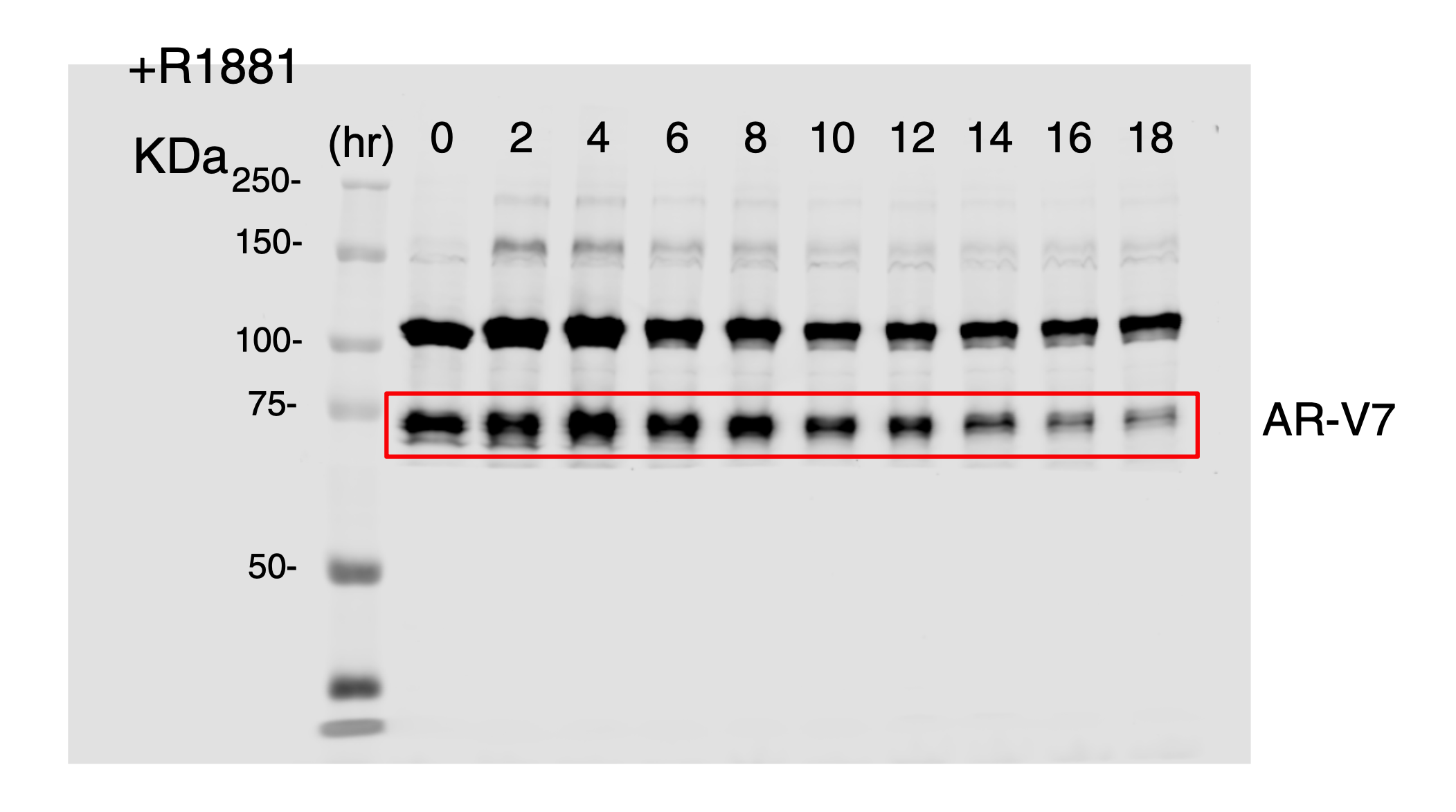

Supplement: Supplementary file 11 — Appendix Fig 1-6 Source Data [file 44318_2025_510_MOESM11_ESM.zip › Appendix Fig S3/S3A/AR-V7.png]

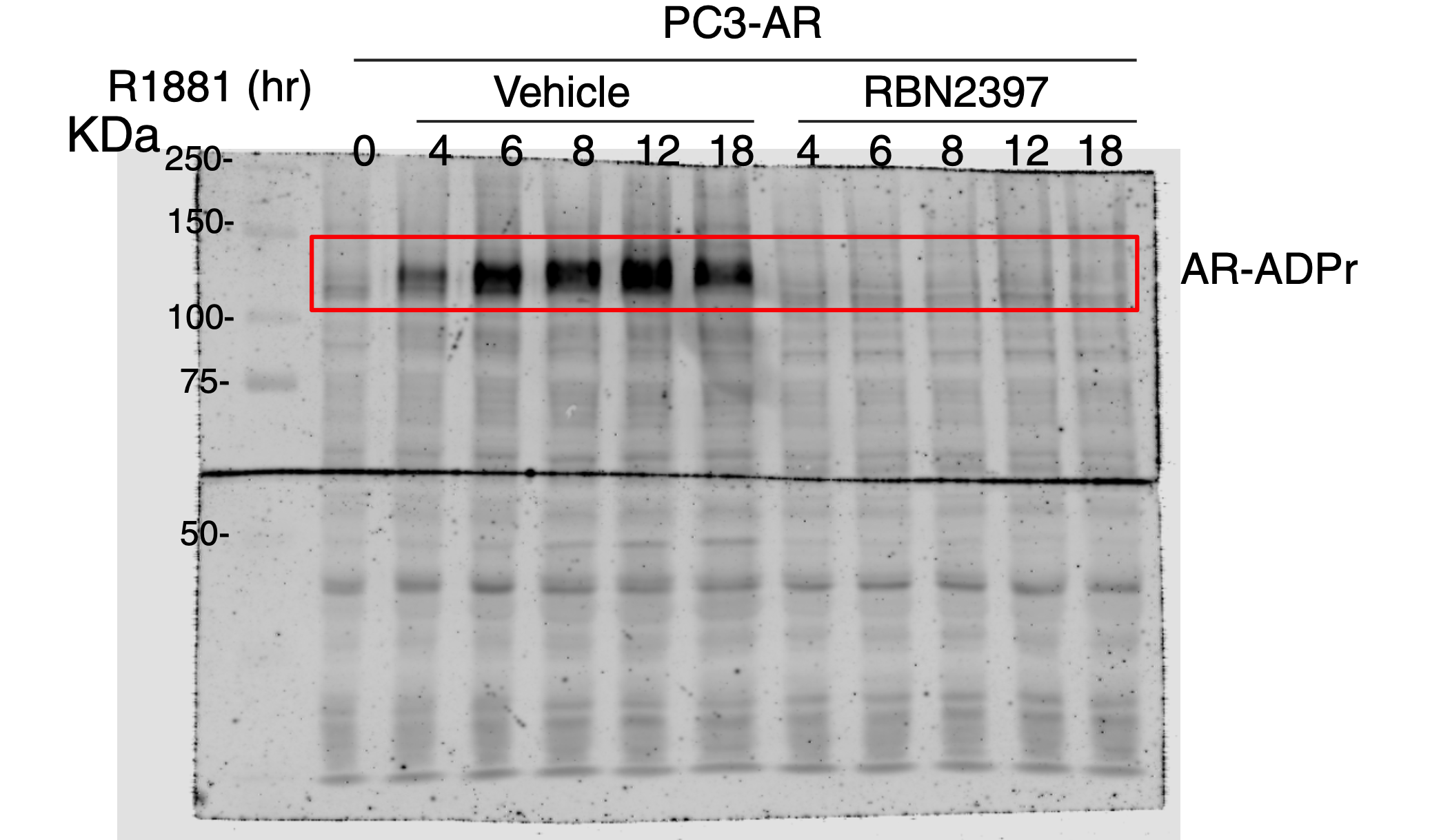

Supplement: Supplementary file 11 — Appendix Fig 1-6 Source Data [file 44318_2025_510_MOESM11_ESM.zip › Appendix Fig S3/S3F/AR-ADPr.png]

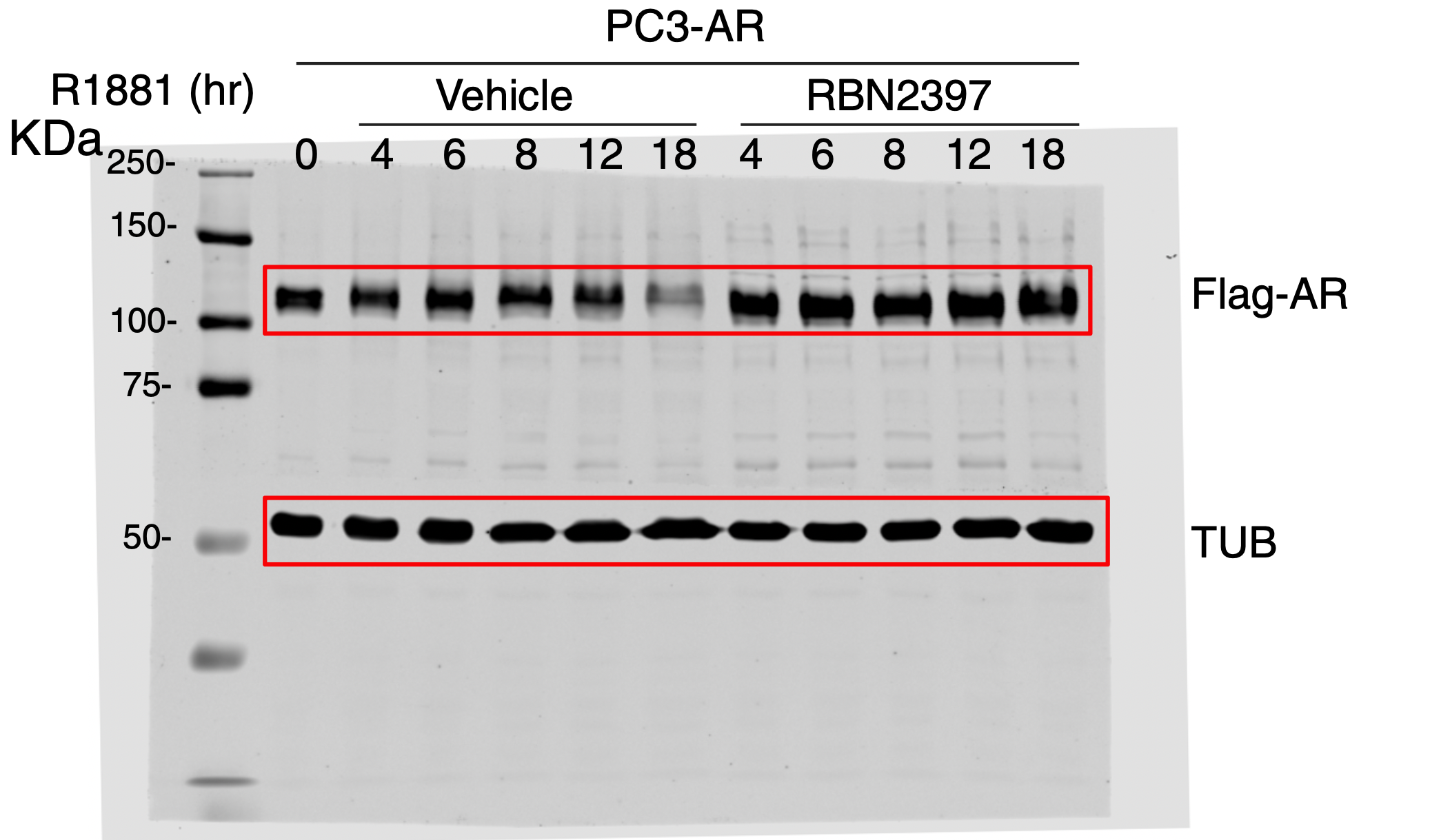

Supplement: Supplementary file 11 — Appendix Fig 1-6 Source Data [file 44318_2025_510_MOESM11_ESM.zip › Appendix Fig S3/S3F/Flag-AR and TUB.png]

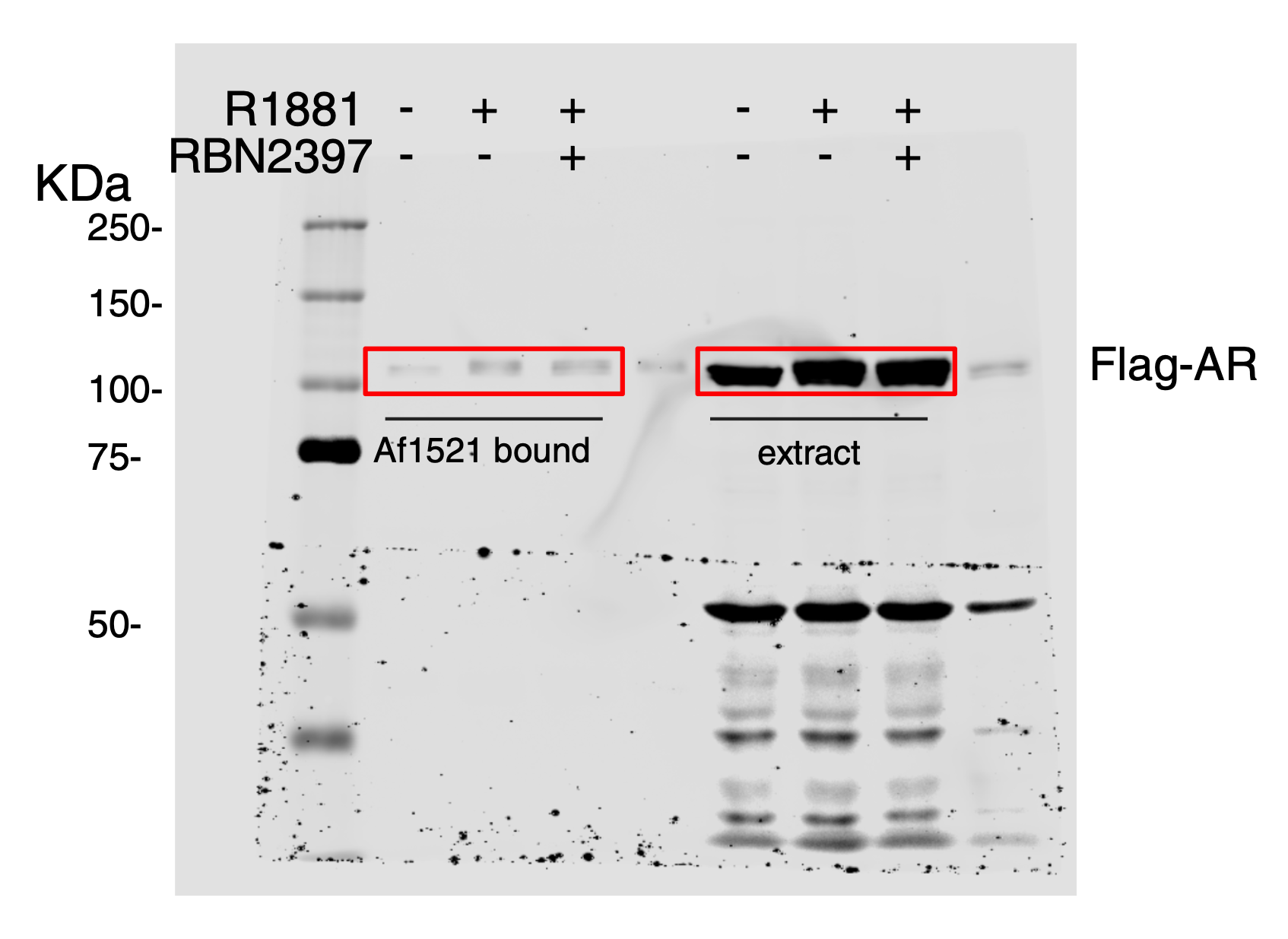

Supplement: Supplementary file 11 — Appendix Fig 1-6 Source Data [file 44318_2025_510_MOESM11_ESM.zip › Appendix Fig S5/S5H/Mut 1 Flag-AR.png]

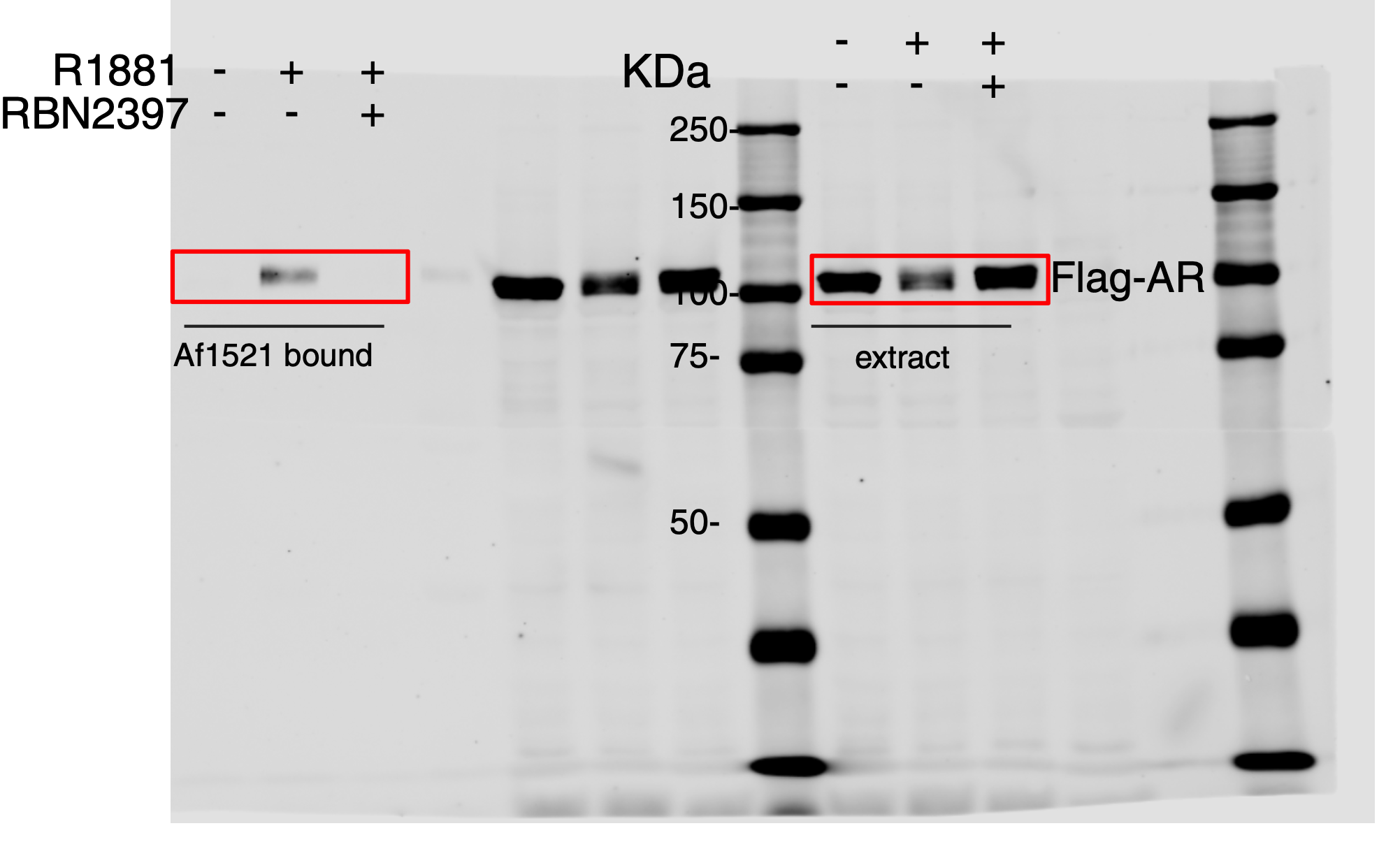

Supplement: Supplementary file 11 — Appendix Fig 1-6 Source Data [file 44318_2025_510_MOESM11_ESM.zip › Appendix Fig S5/S5H/Mut 2 Flag-AR.png]

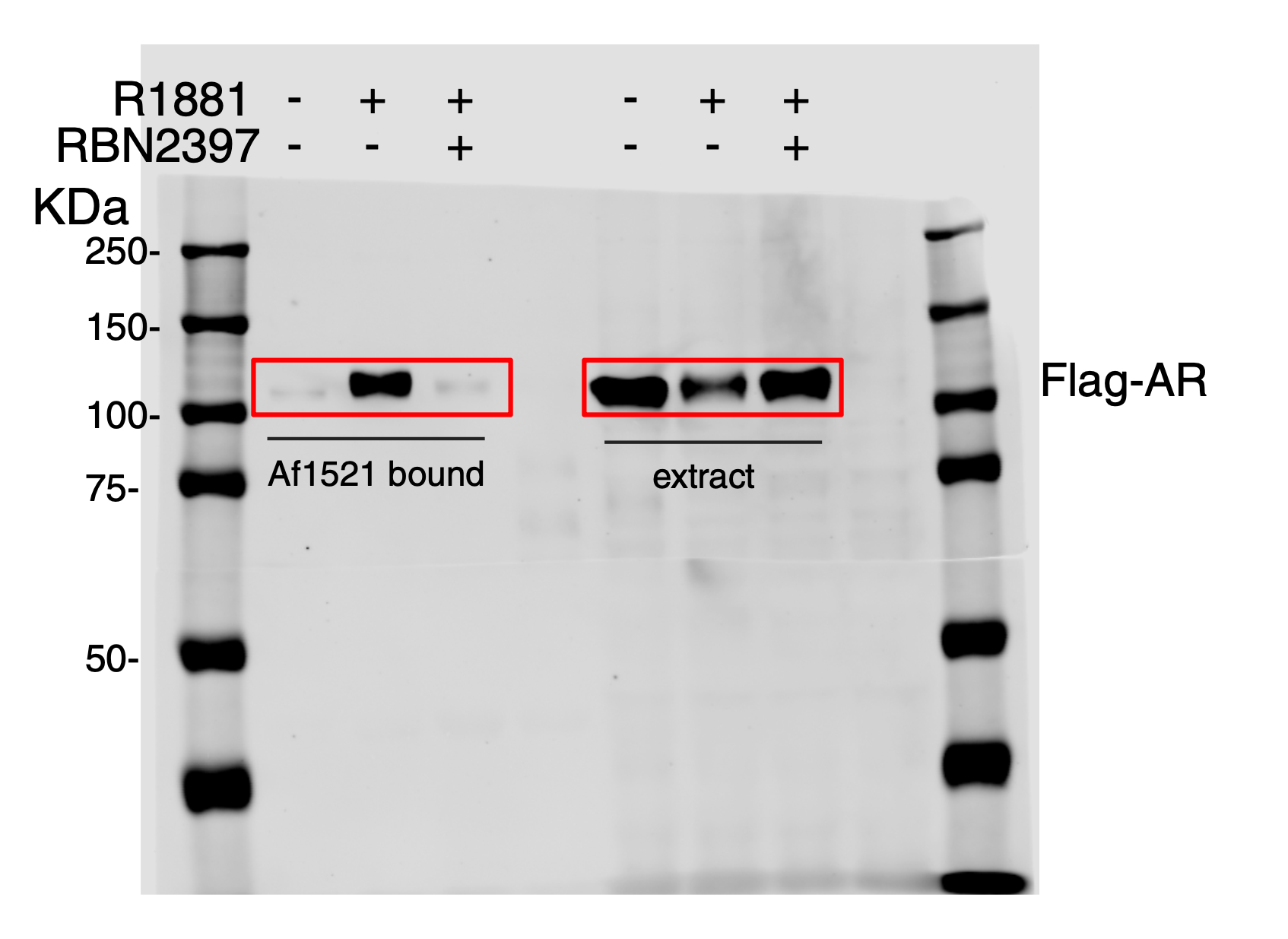

Supplement: Supplementary file 11 — Appendix Fig 1-6 Source Data [file 44318_2025_510_MOESM11_ESM.zip › Appendix Fig S5/S5H/WT Flag-AR.png]

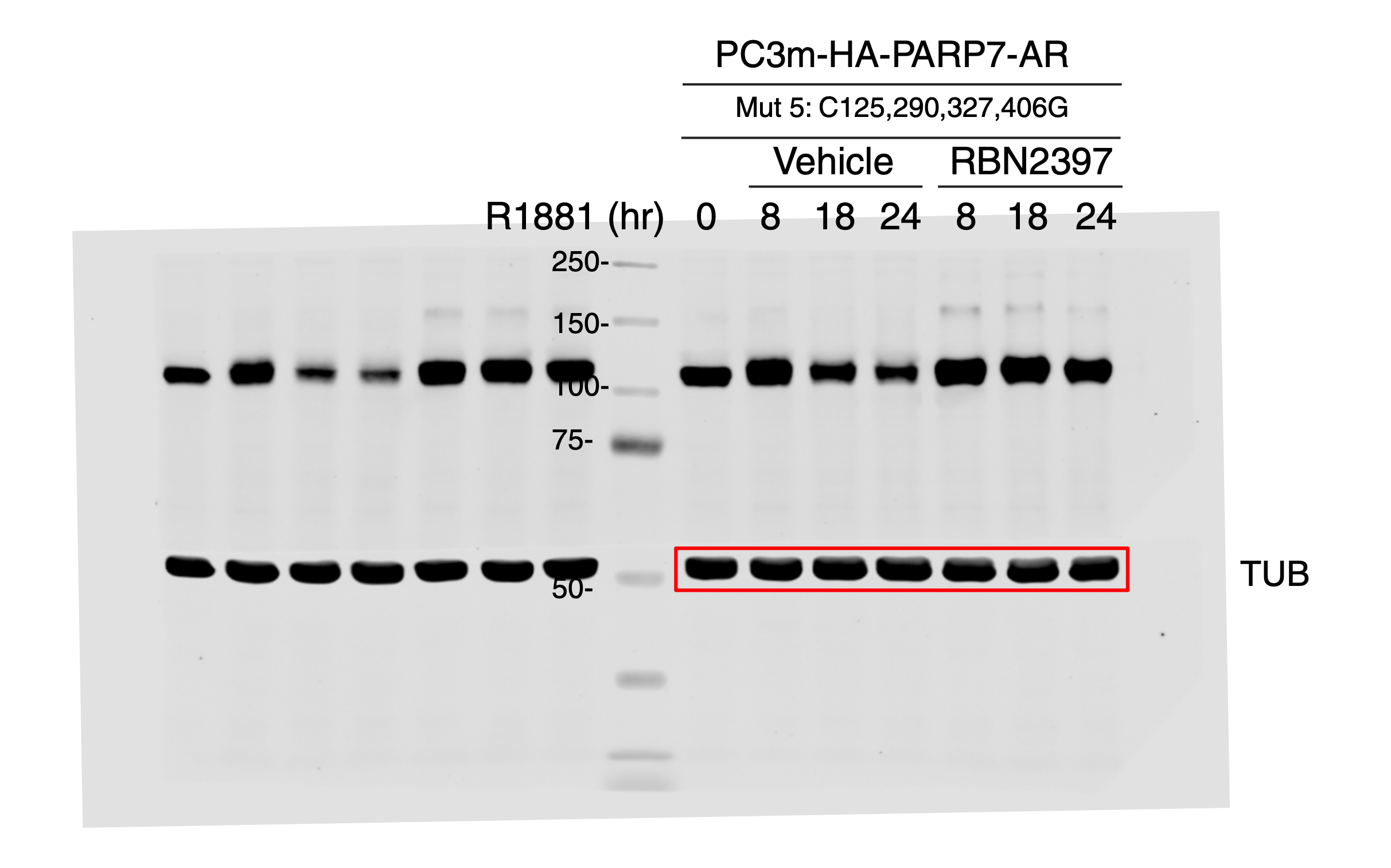

Supplement: Supplementary file 11 — Appendix Fig 1-6 Source Data [file 44318_2025_510_MOESM11_ESM.zip › Appendix Fig S5/S5A/TUB.png]

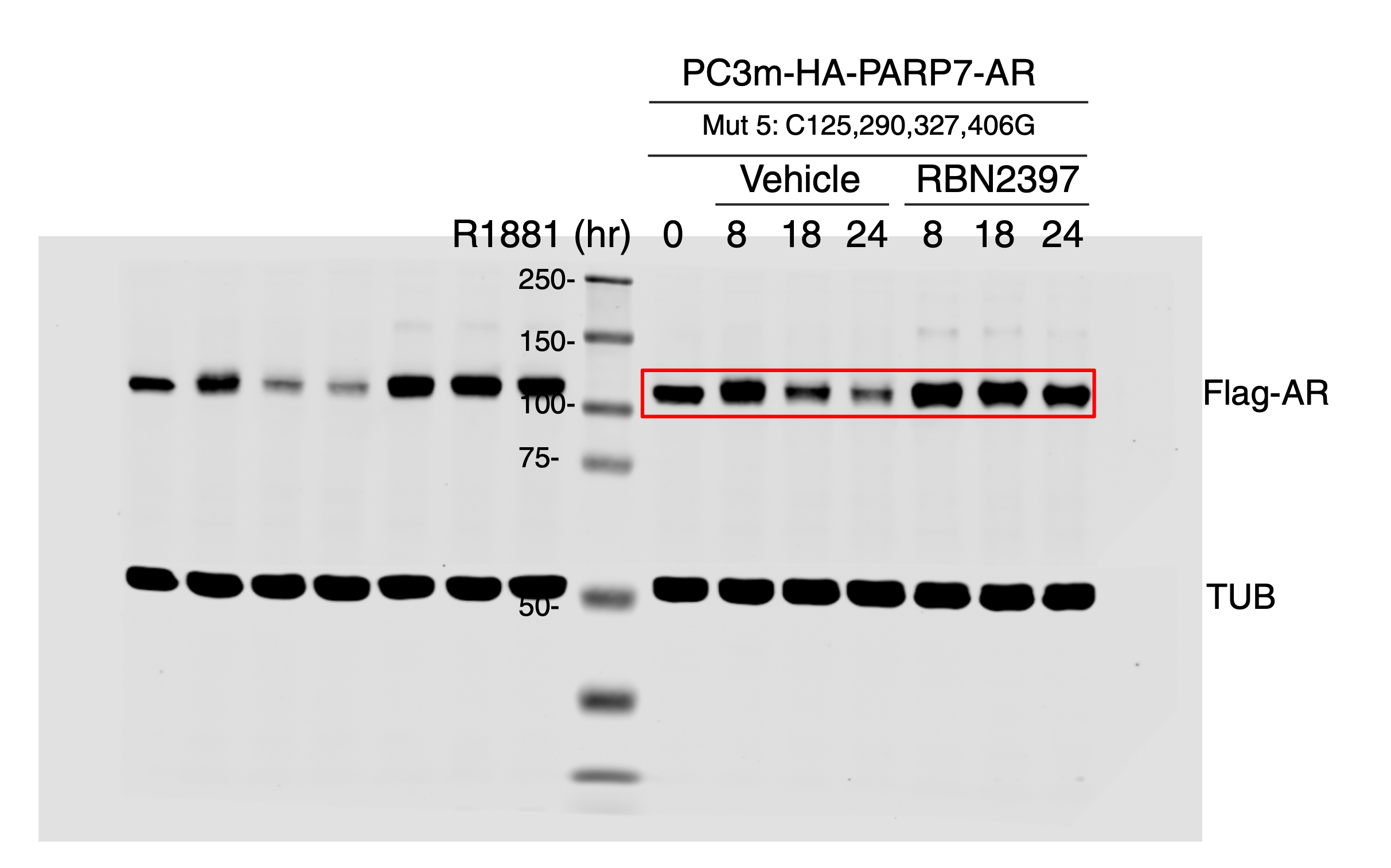

Supplement: Supplementary file 11 — Appendix Fig 1-6 Source Data [file 44318_2025_510_MOESM11_ESM.zip › Appendix Fig S5/S5A/Flag-AR.png]

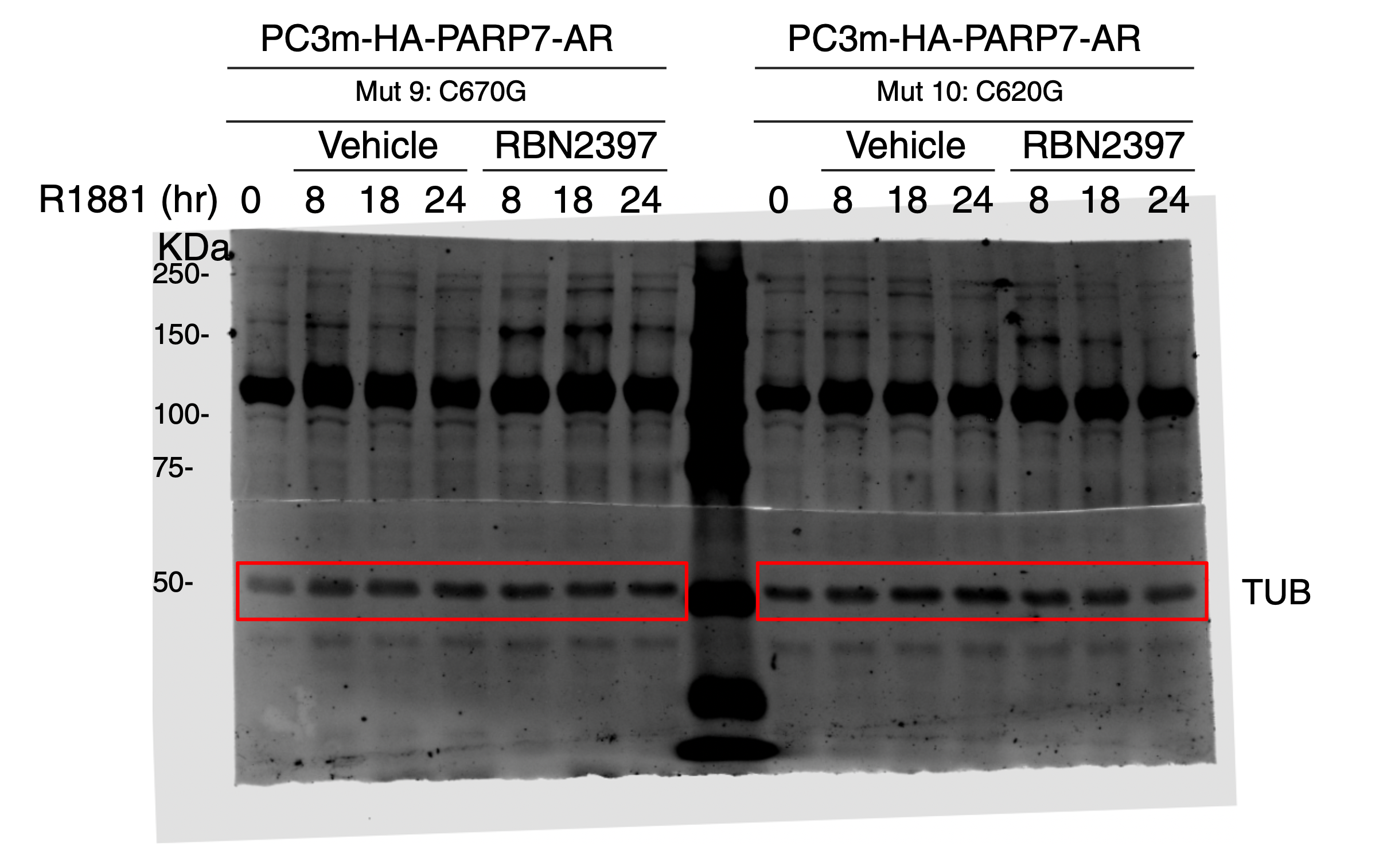

Supplement: Supplementary file 11 — Appendix Fig 1-6 Source Data [file 44318_2025_510_MOESM11_ESM.zip › Appendix Fig S5/S5E, F/TUB.png]

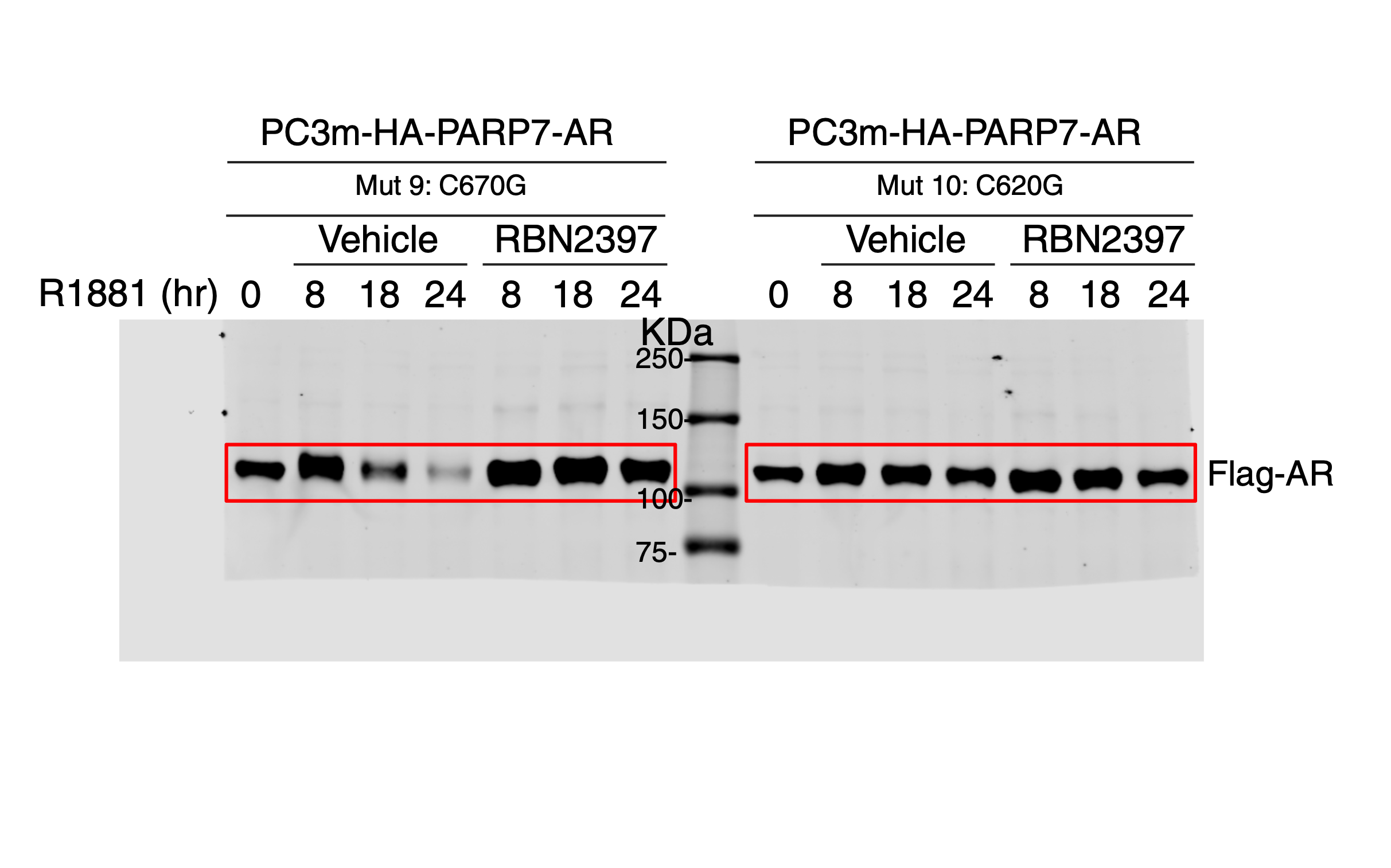

Supplement: Supplementary file 11 — Appendix Fig 1-6 Source Data [file 44318_2025_510_MOESM11_ESM.zip › Appendix Fig S5/S5E, F/Flag-AR.png]

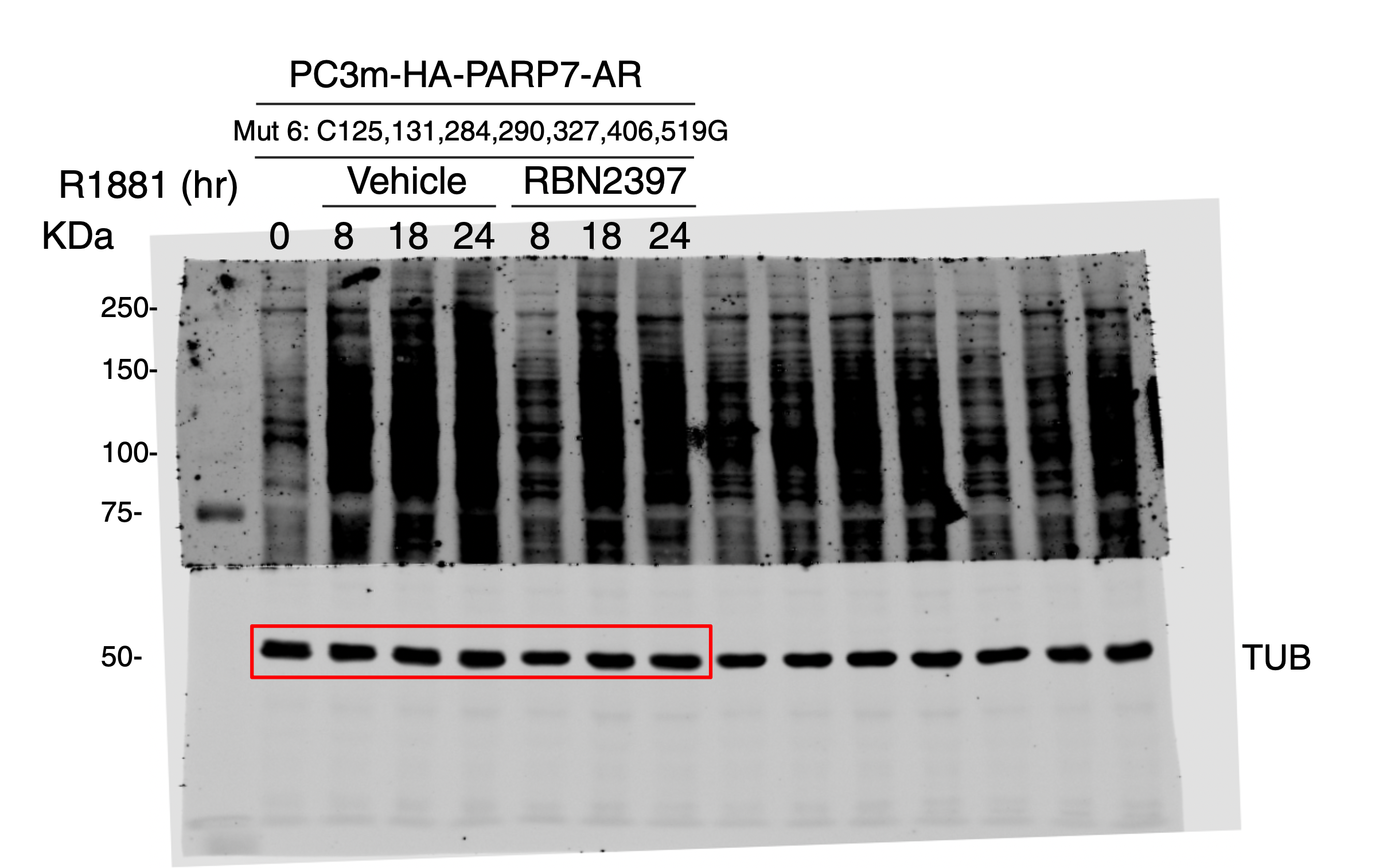

Supplement: Supplementary file 11 — Appendix Fig 1-6 Source Data [file 44318_2025_510_MOESM11_ESM.zip › Appendix Fig S5/S5B/TUB.png]

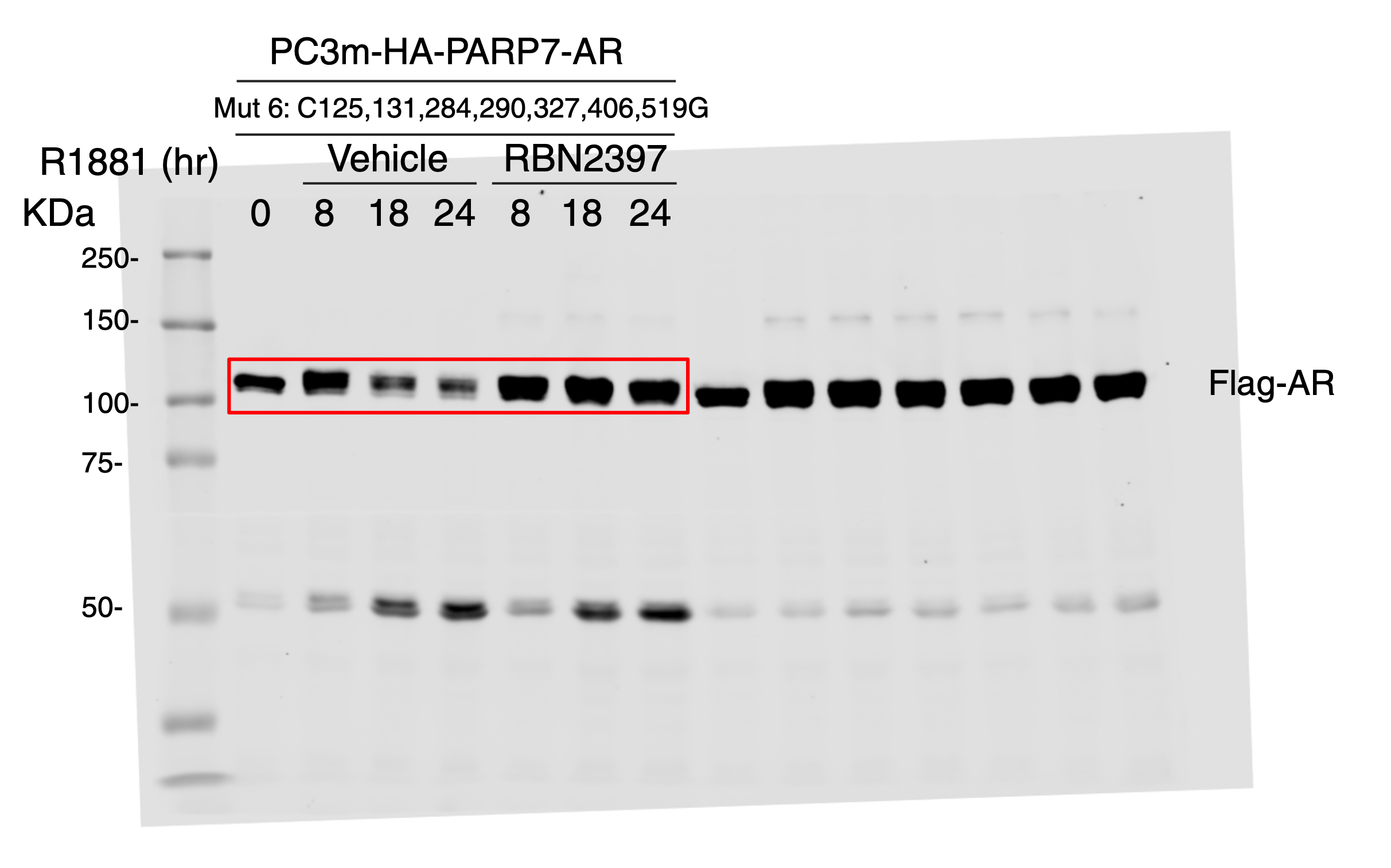

Supplement: Supplementary file 11 — Appendix Fig 1-6 Source Data [file 44318_2025_510_MOESM11_ESM.zip › Appendix Fig S5/S5B/Flag-AR.png]

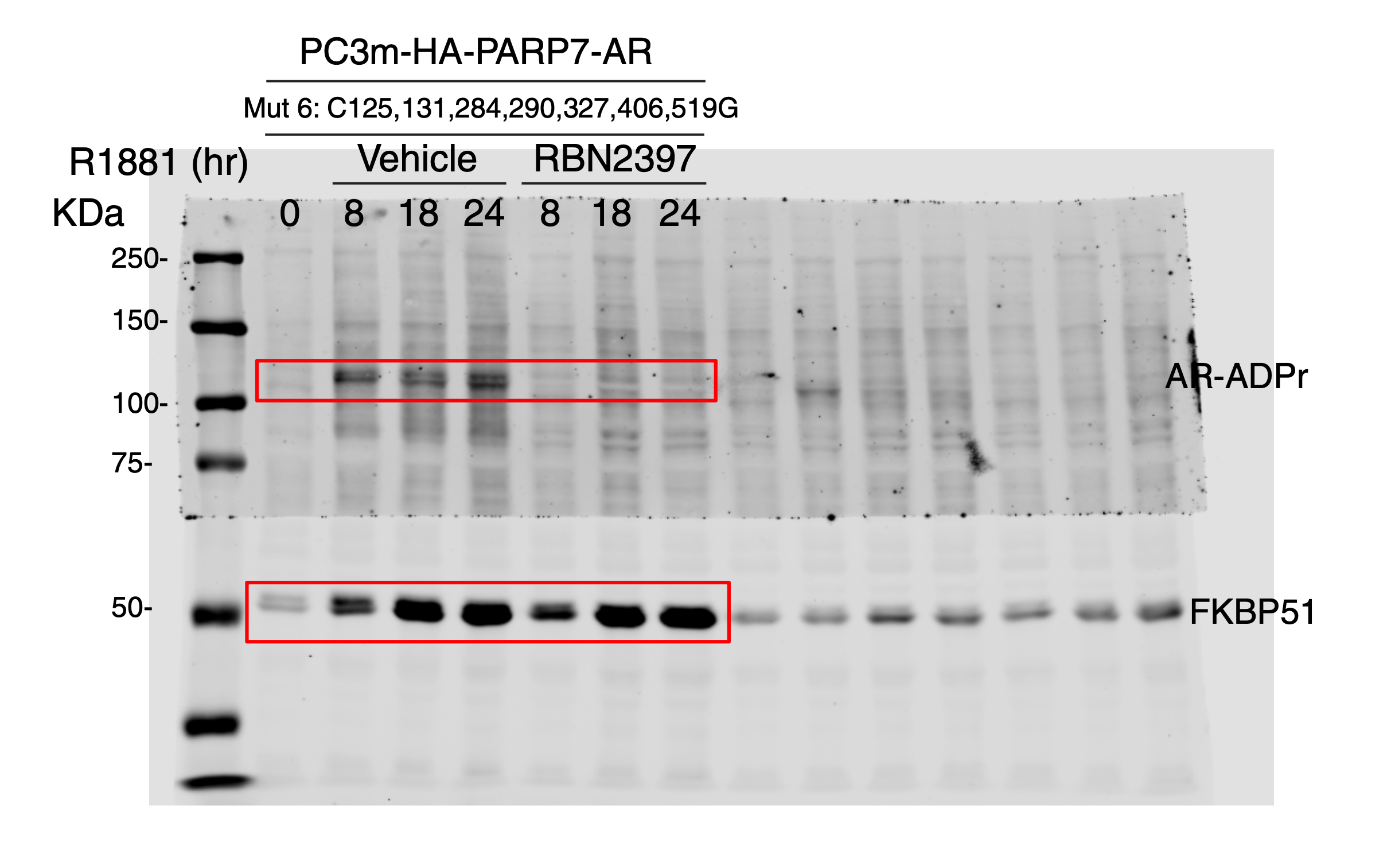

Supplement: Supplementary file 11 — Appendix Fig 1-6 Source Data [file 44318_2025_510_MOESM11_ESM.zip › Appendix Fig S5/S5B/AR-ADPr and FKBP51.png]

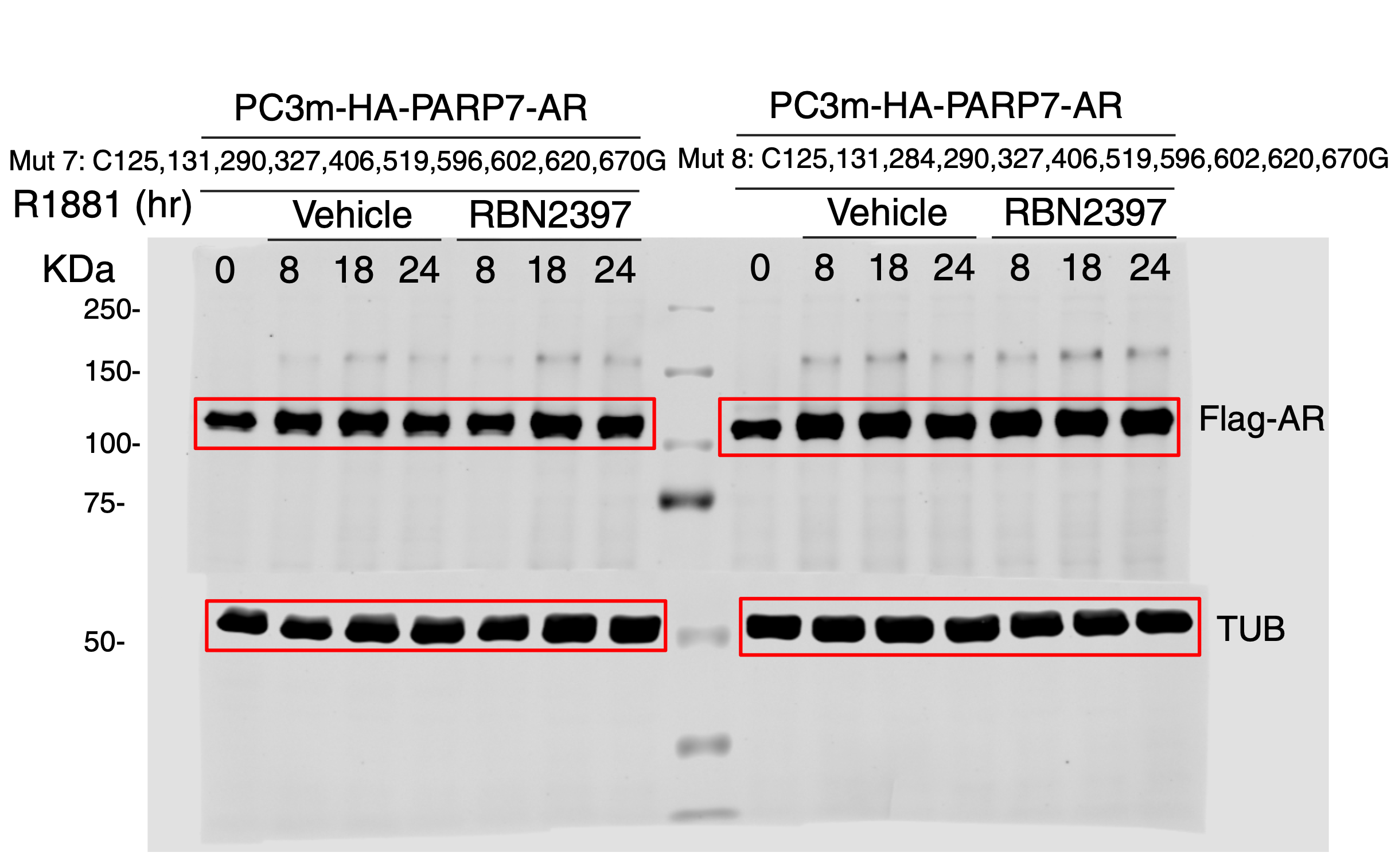

Supplement: Supplementary file 11 — Appendix Fig 1-6 Source Data [file 44318_2025_510_MOESM11_ESM.zip › Appendix Fig S5/S5C, D/Flag-AR and TUB.png]

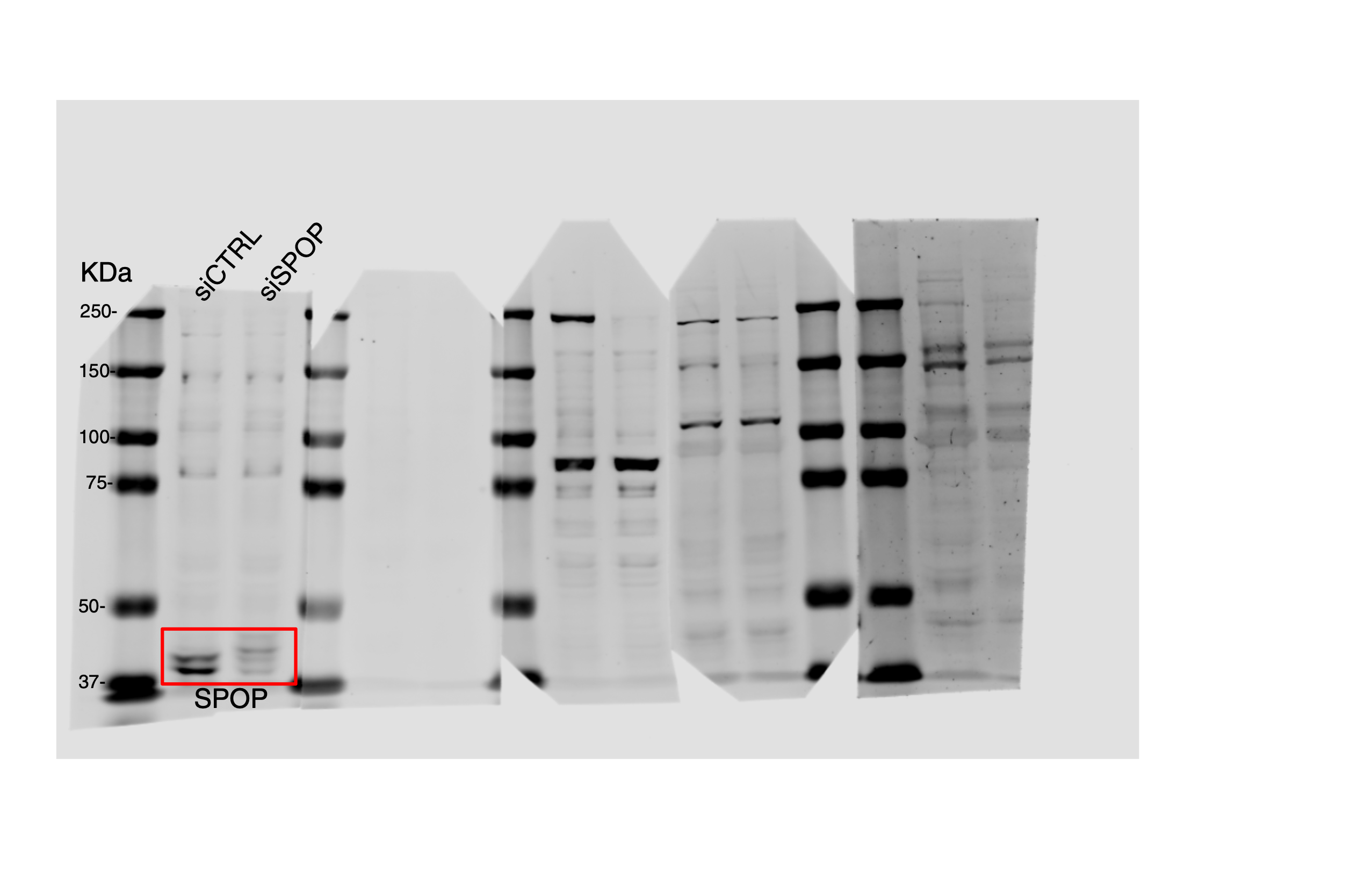

Supplement: Supplementary file 11 — Appendix Fig 1-6 Source Data [file 44318_2025_510_MOESM11_ESM.zip › Appendix Fig S6/S6A/SPOP.png]

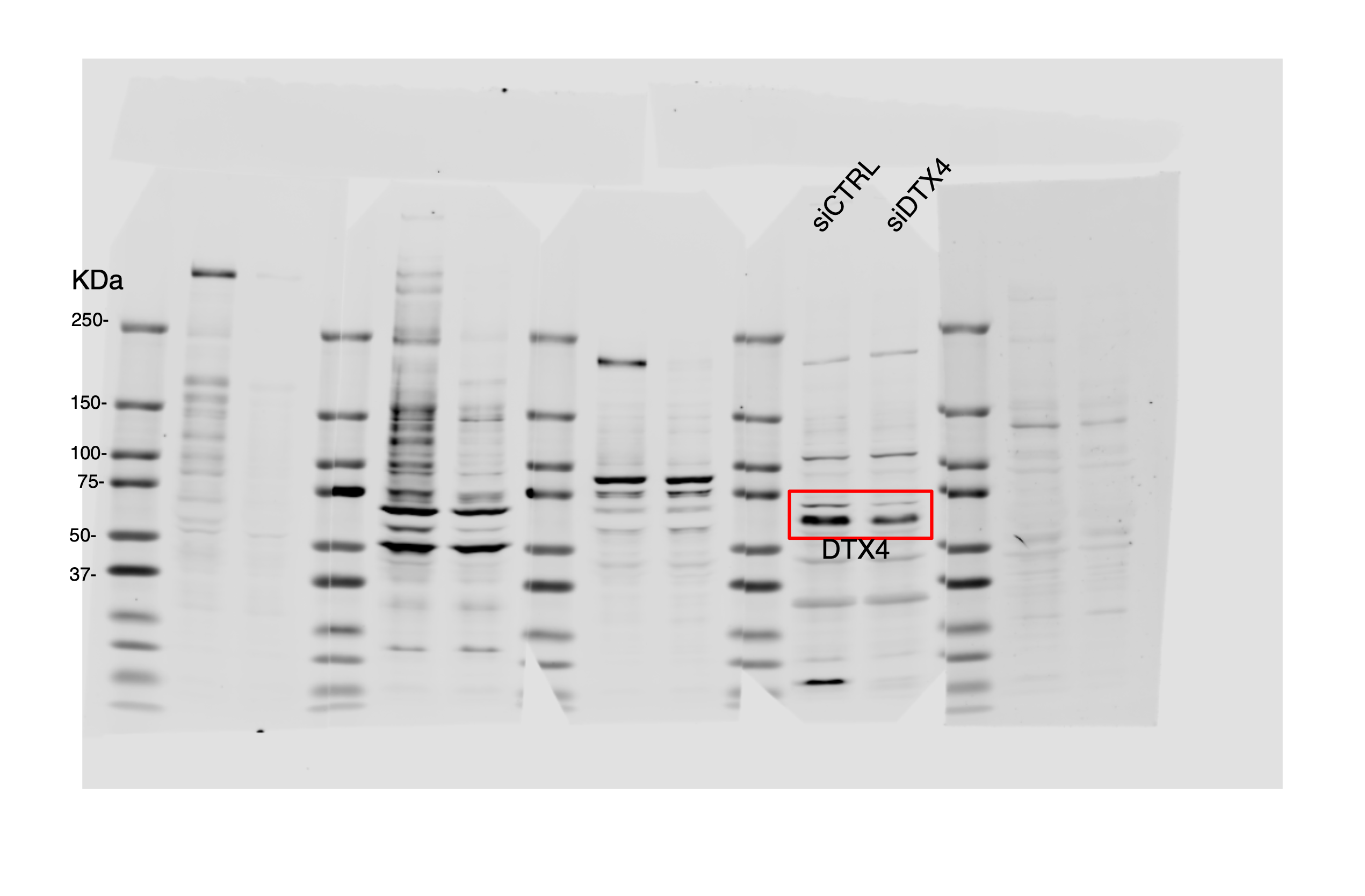

Supplement: Supplementary file 11 — Appendix Fig 1-6 Source Data [file 44318_2025_510_MOESM11_ESM.zip › Appendix Fig S6/S6A/DTX4.png]

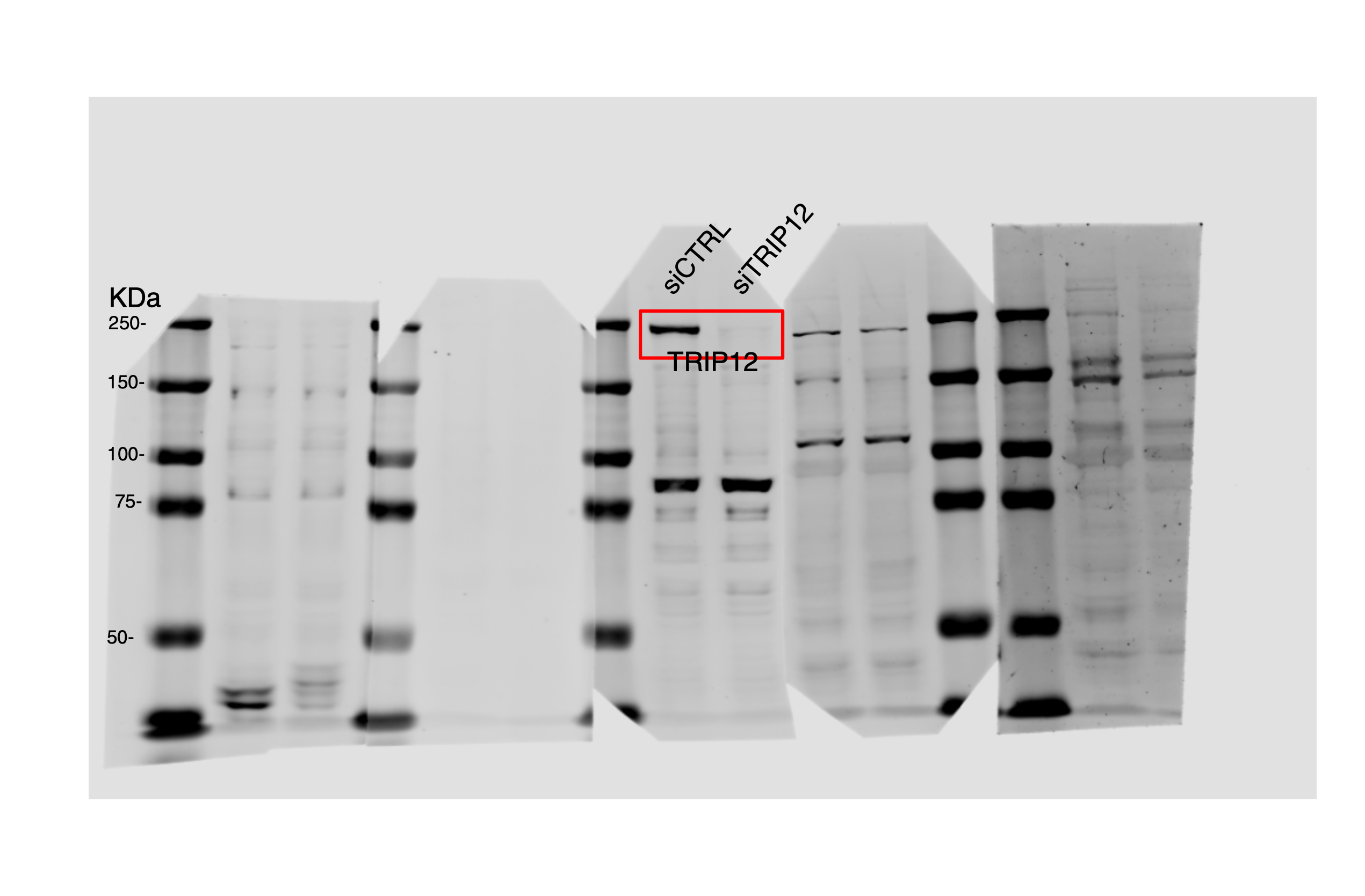

Supplement: Supplementary file 11 — Appendix Fig 1-6 Source Data [file 44318_2025_510_MOESM11_ESM.zip › Appendix Fig S6/S6A/TRIP12.png]

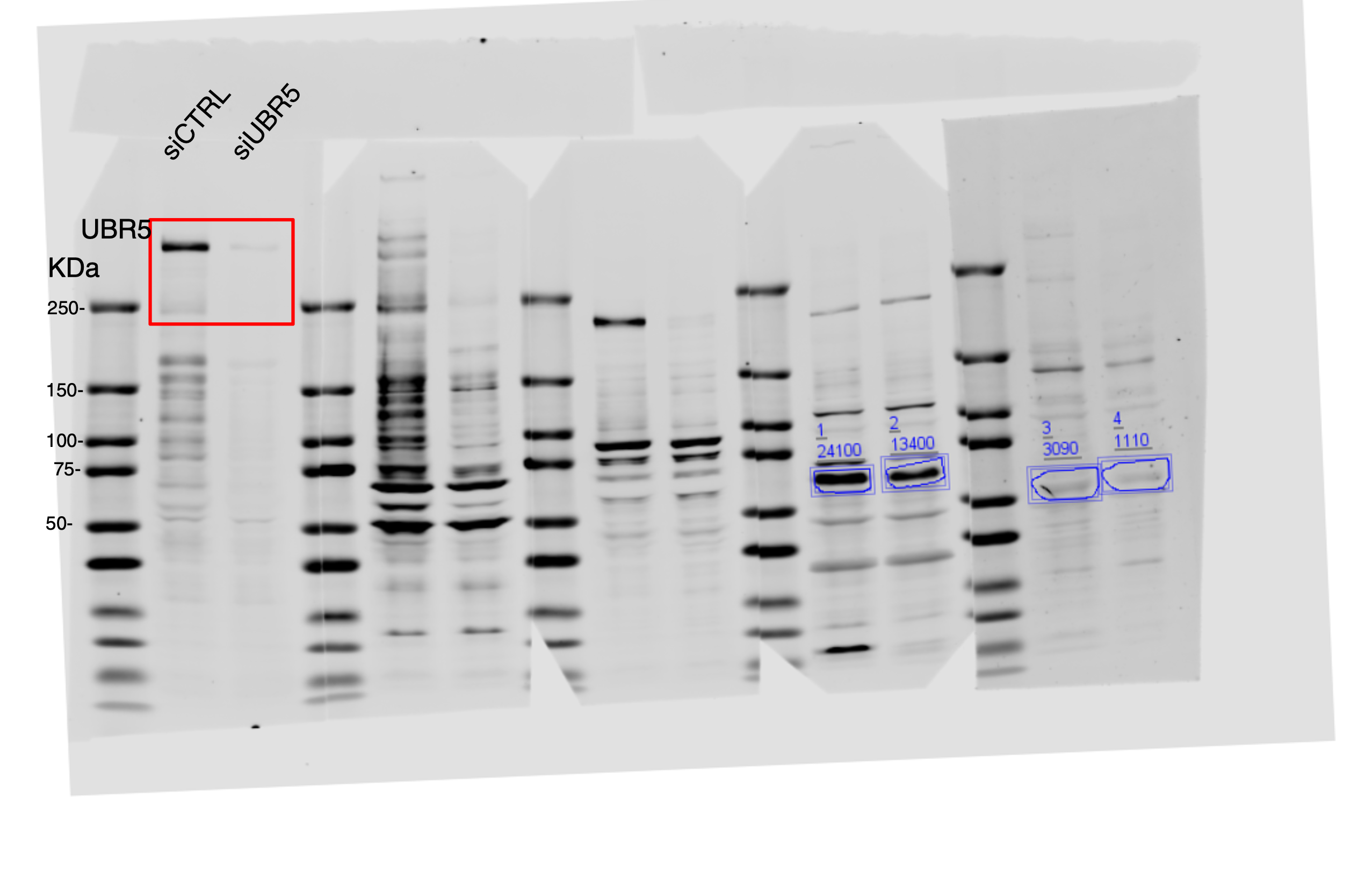

Supplement: Supplementary file 11 — Appendix Fig 1-6 Source Data [file 44318_2025_510_MOESM11_ESM.zip › Appendix Fig S6/S6A/UBR5.png]

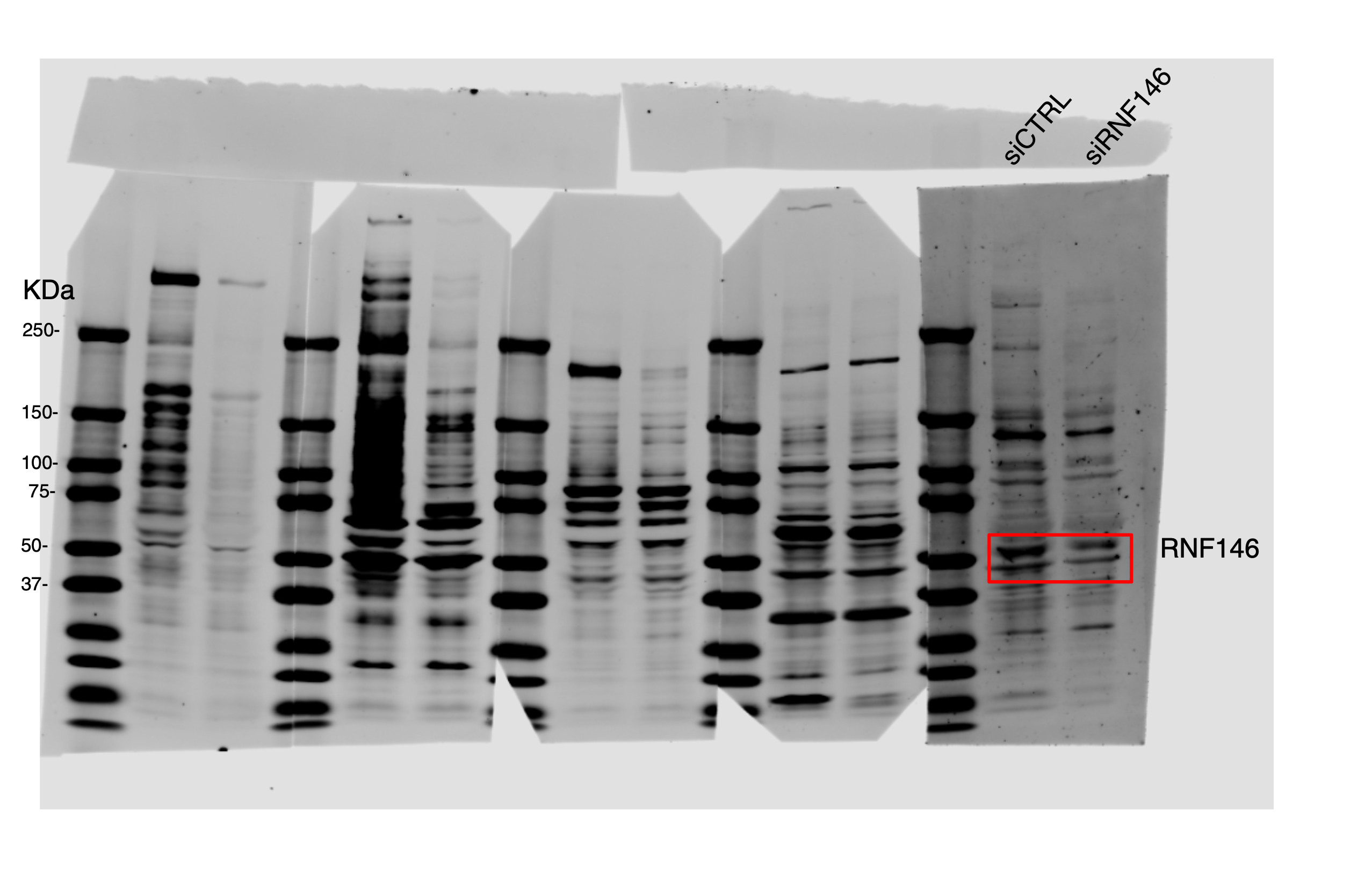

Supplement: Supplementary file 11 — Appendix Fig 1-6 Source Data [file 44318_2025_510_MOESM11_ESM.zip › Appendix Fig S6/S6A/RNF146.png]

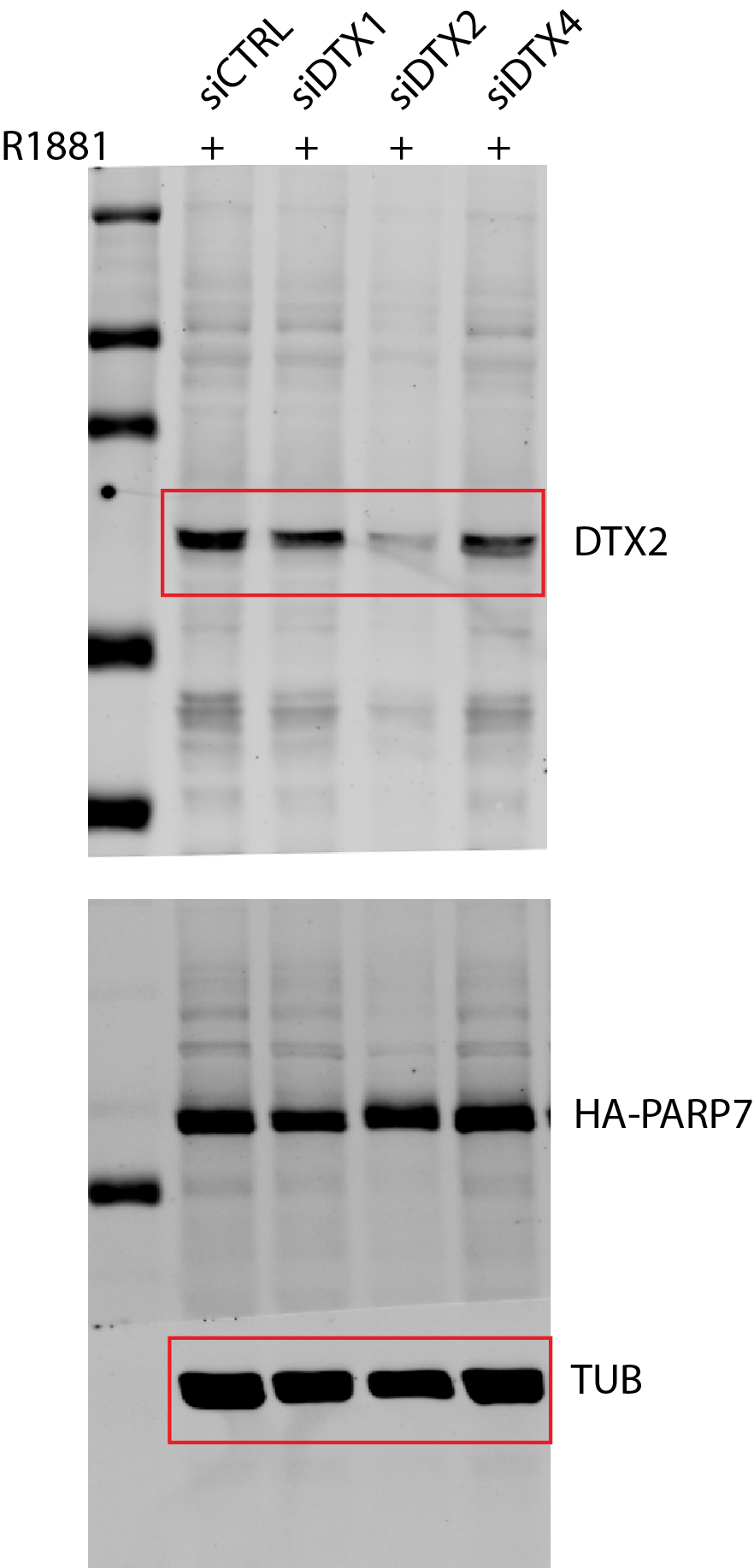

Supplement: Supplementary file 11 — Appendix Fig 1-6 Source Data [file 44318_2025_510_MOESM11_ESM.zip › Appendix Fig S6/S6A/DTX2.png]

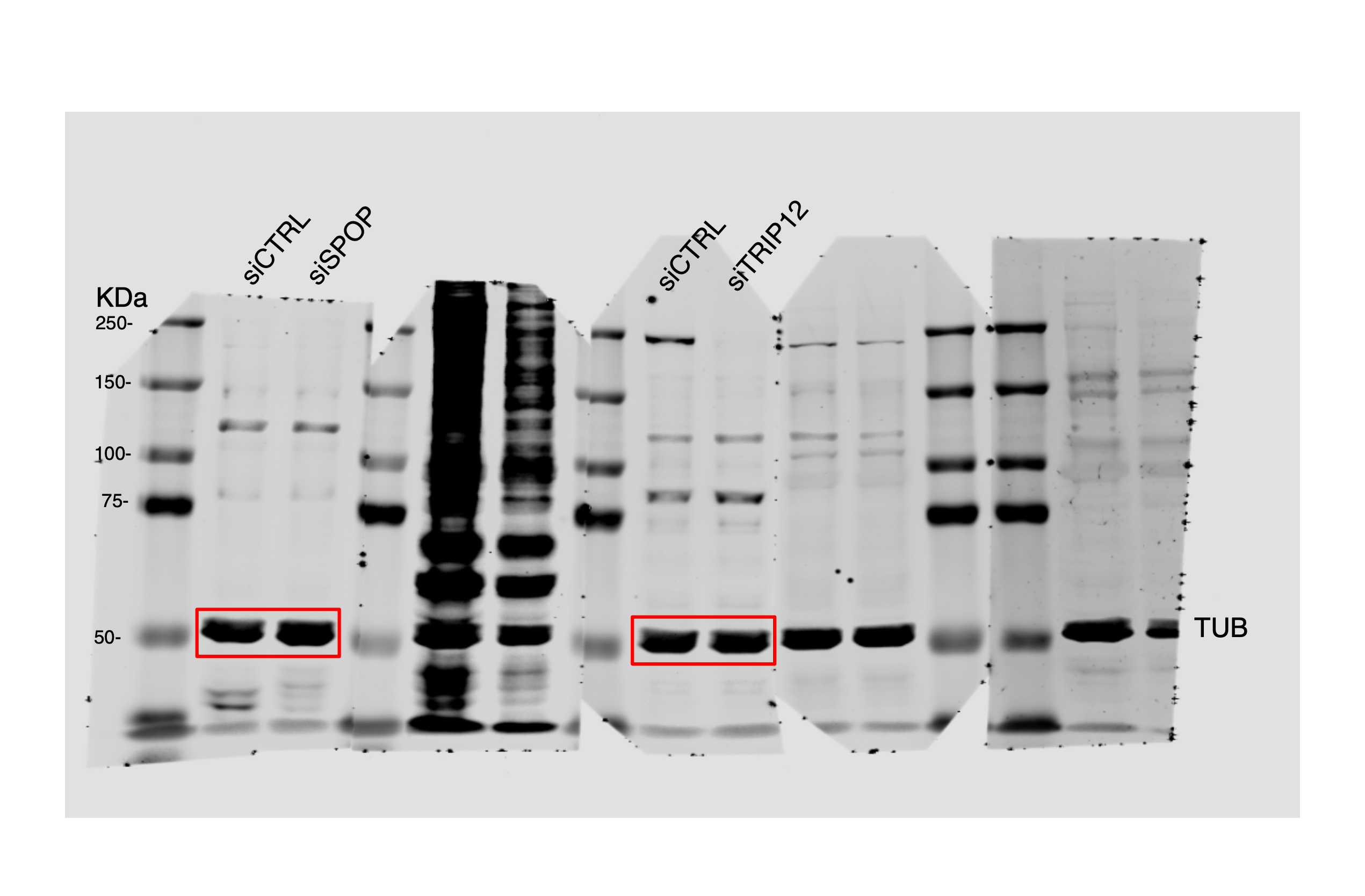

Supplement: Supplementary file 11 — Appendix Fig 1-6 Source Data [file 44318_2025_510_MOESM11_ESM.zip › Appendix Fig S6/S6A/TUB siSPOP, siTRIP12.png]

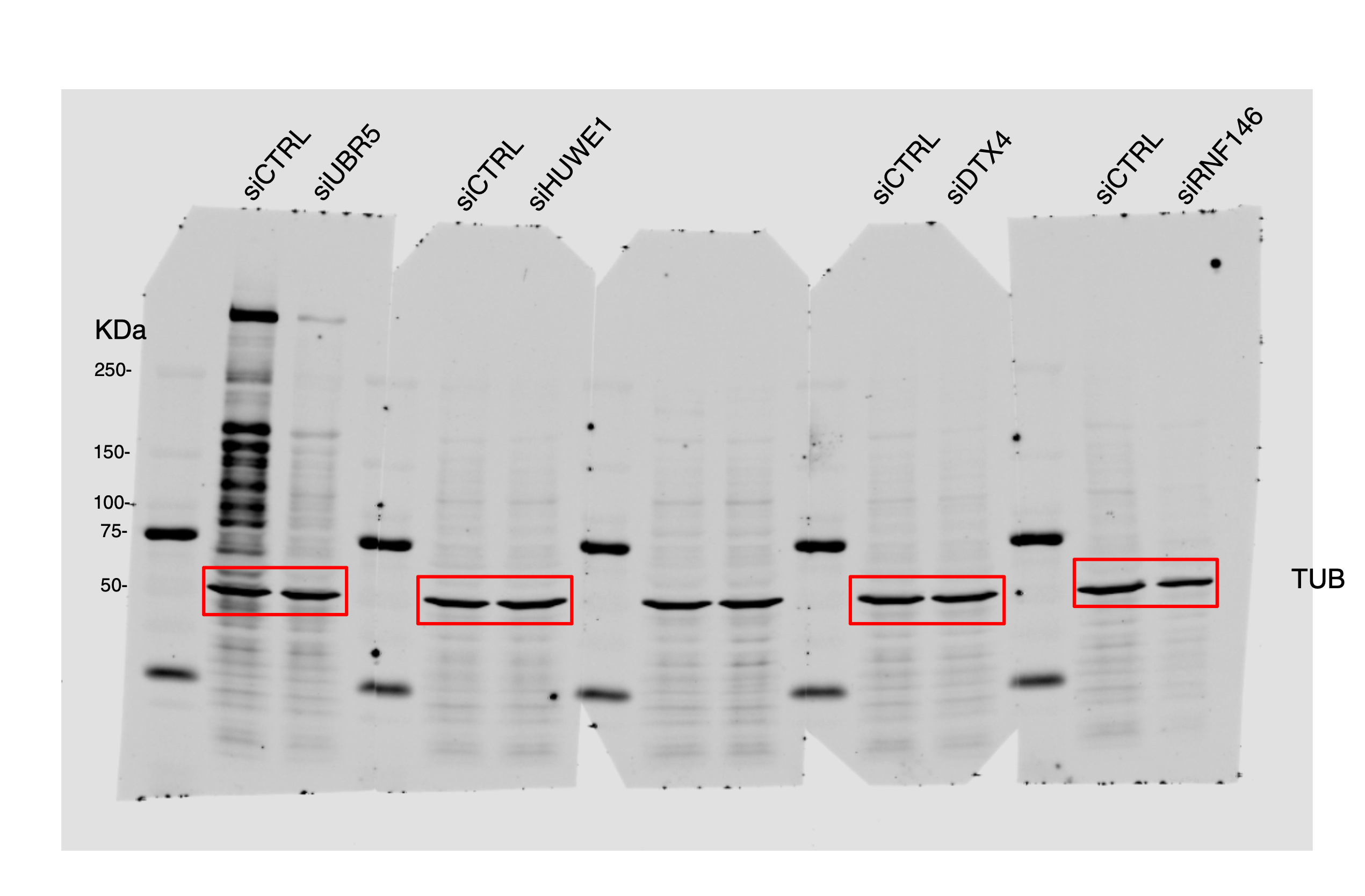

Supplement: Supplementary file 11 — Appendix Fig 1-6 Source Data [file 44318_2025_510_MOESM11_ESM.zip › Appendix Fig S6/S6A/TUB siUBR5, siHUWE1, siDTX4, siRNF146.png]

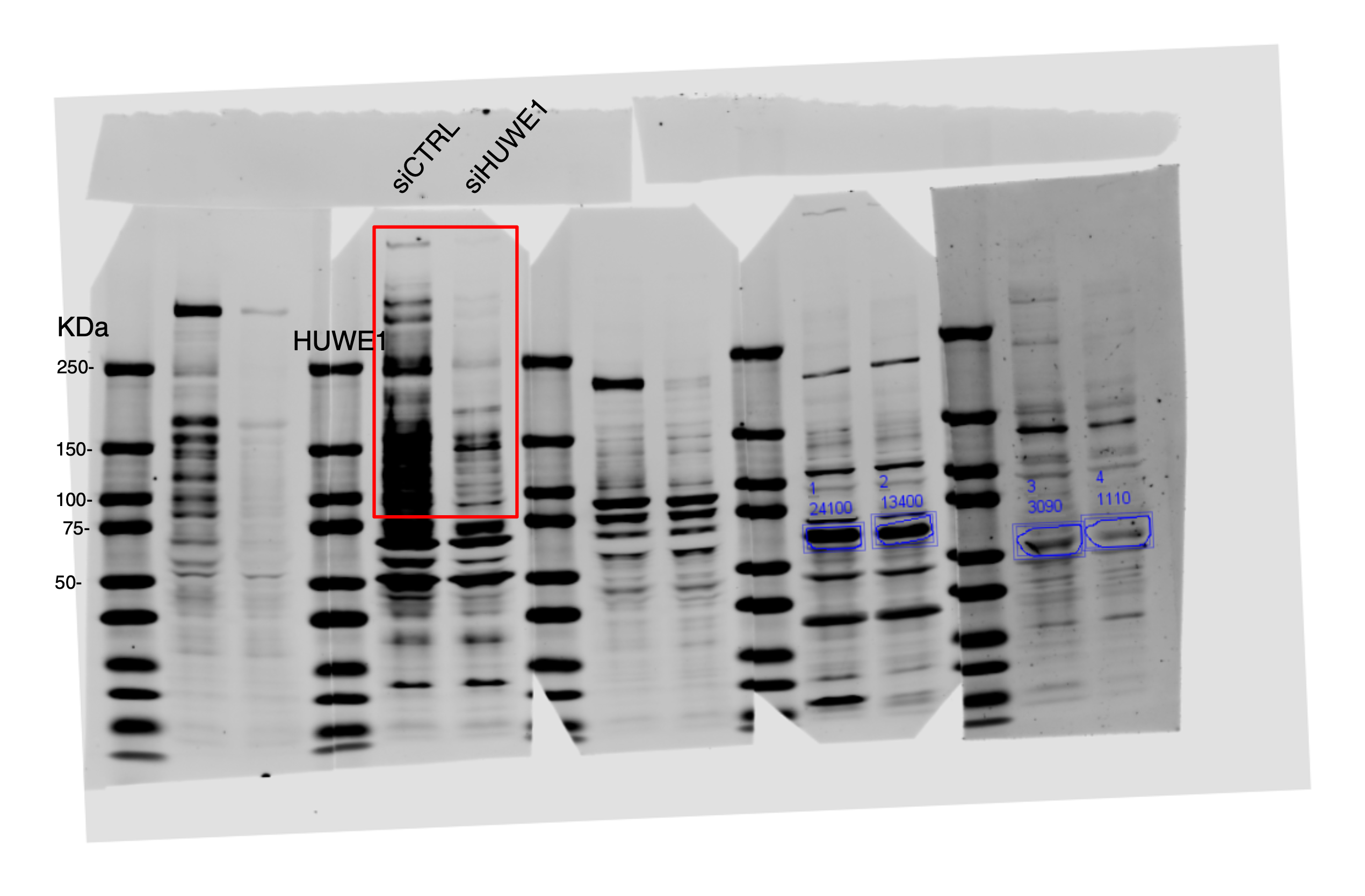

Supplement: Supplementary file 11 — Appendix Fig 1-6 Source Data [file 44318_2025_510_MOESM11_ESM.zip › Appendix Fig S6/S6A/HUWE1.png]

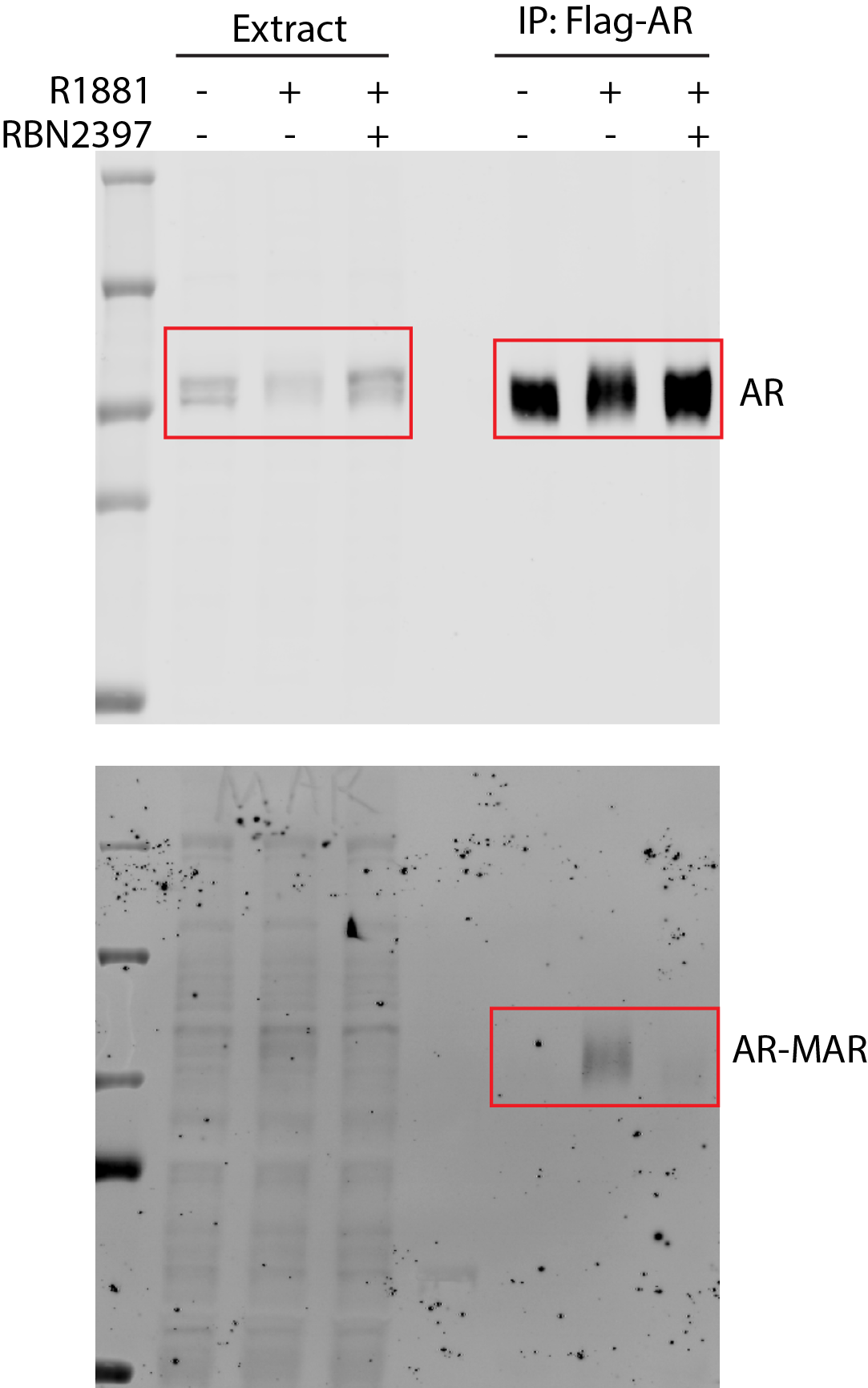

Supplement: Supplementary file 11 — Appendix Fig 1-6 Source Data [file 44318_2025_510_MOESM11_ESM.zip › Appendix Fig S6/S6D/FlagAR, FlagAR-MAR.png]
