# Supplementary material for: Parp7 generates an ADP-ribosyl degron that controls negative feedback of androgen signaling
Source: EMBO J. 2025 Jul 18;44(17):4720–44. doi: 10.1038/s44318-025-00510-4 (PMC12402299; doi:10.1038/s44318-025-00510-4)
Supplement: Supplementary file 2 — Peer Review File [file 44318_2025_510_MOESM2_ESM.pdf]

THE EMBO JOURNAL

Parp7 generates an ADP-ribosyl degron that controls negative feedback of androgen signaling

Krzysztof Wierbilowicz, Chunsong Yang, Ahmed Almaghasilah, Patryk Wesolowski, Philipp Pracht, Natalia Dworak, Jack Masur, Sven Wijngaarden, Dmitri Filippov, David Wales, Joshua Kelley, Aakrosh Ratan, and Bryce Paschal

Corresponding author(s): Bryce Paschal (paschal@virginia.edu)

Review Timeline:

Submission Date:	8th Jan 25
Editorial Decision:	19th Feb 25
Revision Received:	3rd Jun 25
Editorial Decision:	24th Jun 25
Revision Received:	30th Jun 25
Accepted:	2nd Jul 25

Editor: Hartmut Vodermaier

Transaction Report:

(Note: With the exception of the correction of typographical or spelling errors that could be a source of ambiguity, letters and reports are not edited. Depending on transfer agreements, referee reports obtained elsewhere may or may not be included in this compilation. Referee reports are anonymous unless the Referee chooses to sign their reports.)

Dr. Bryce M Paschal
University of Virginia
Center for Cell Signaling
Box 800577 Health Systems
Charlottesville, VA 22908

19th Feb 2025

Re: EMBOJ-2025-120145
Parp7 generates an ADP-ribosyl degron that controls negative feedback of androgen signaling

Dear Dr. Paschal,

Thank you for submitting your study on PAPR7-dependent generation of an ADP-ribosyl degron on androgen receptor to The EMBO Journal. Please excuse the delay in getting back to you with a decision, caused by limited referee availability in January. We have now received a full set of comments from three expert referees, copied below for your information. As you will see, all referees acknowledge the interest and potential importance of your conclusions. However, while referee 3 has only a limited number of presentational suggestions, the other two raise several well-taken major concerns. The two most salient issues appear to be the support for AR-C620 as the key modification site (ref 1 points 1, 2, ref 2 point 6), and the uncertainty of Flag-AR/AR-ADPr targeting by DTX2 and/or other ubiquitin ligases (ref 1 points 4, 5, ref 2 points 2, 4).

In my view, the suitability of this work for EMBO Journal publication would ultimately depend on whether these key concerns could be satisfactorily clarified. Thus, should you be able to adequately address them, as well as the various specific comments of all three referees, we would be open to pursuing a revised version further for publication. In this respect, I note that some other suggestions of the referees may go beyond the scope of such a revision, but should stress that they would nevertheless need to be diligently responded to at the time of resubmission, given that it is our policy to consider only a single round of major revision. In this light, I would very much encourage you to contact me with a revision plan and preliminary point-by-point response already during the early stages of your revision work, so that we could discuss if and how the main points could be resolved, or whether a less completely revised manuscript might alternatively become suitable for publication in one of our sister journals like EMBO Reports or Life Science Alliance. Of course, we would also be open to extension of the default three-months revision period if needed; our 'scooping protection' (meaning that competing work appearing elsewhere in the meantime will not affect our considerations of your study) would of course remain valid also throughout such an extension.

Detailed information on preparing, formatting and uploading a revised manuscript can be found below and in our Guide to Authors. Thank you again for the opportunity to consider this work for The EMBO Journal, and I look forward to hearing from you in due time.

With kind regards,

Hartmut

Hartmut Vodermaier, PhD
Senior Editor, The EMBO Journal
h.vodermaier@embojournal.org

*** PLEASE NOTE: All revised manuscript are subject to initial checks for completeness and adherence to our formatting guidelines. Revisions may be returned to the authors and delayed in their editorial re-evaluation if they fail to comply to the following requirements (see also our Guide to Authors for further information):

1) Every manuscript requires a Data Availability section (even if only stating that no deposited datasets are included). Primary datasets or computer code produced in the current study have to be deposited in appropriate public repositories prior to resubmission, and reviewer access details provided in case that public access is not yet allowed. Further information: embopress.org/page/journal/14602075/authorguide#dataavailability

2) Each figure legend must specify
- size of the scale bars that are mandatory for all micrograph panels
- the statistical test used to generate error bars and P-values
- the type error bars (e.g., S.E.M., S.D.)

- the number (n) and nature (biological or technical replicate) of independent experiments underlying each data point
- Figures may not include error bars for experiments with n<3; scatter plots showing individual data points should be used instead.

3) Revised manuscript text (including main tables, and figure legends for main and EV figures) has to be submitted as editable text file (e.g., .docx format). We encourage highlighting of changes (e.g., via text color) for the referees' reference.

4) Each main and each Expanded View (EV) figure should be uploaded as individual production-quality files (preferably in .eps, .tif, .jpg formats). For suggestions on figure preparation/layout, please refer to our Figure Preparation Guidelines: http://bit.ly/EMBOPressFigurePreparationGuideline

5) Point-by-point response letters should include the original referee comments in full together with your detailed responses to them (and to specific editor requests if applicable), and also be uploaded as editable (e.g., .docx) text files.

6) Please complete our Author Checklist, and make sure that information entered into the checklist is also reflected in the manuscript; the checklist will be available to readers as part of the Review Process File. A download link is found at the top of our Guide to Authors: embopress.org/page/journal/14602075/authorguide

7) All authors listed as (co-)corresponding need to deposit, in their respective author profiles in our submission system, a unique ORCiD identifier linked to their name. Please see our Guide to Authors for detailed instructions.

8) Please note that supplementary information at EMBO Press has been superseded by the 'Expanded View' for inclusion of additional figures, tables, movies or datasets; with up to five EV Figures being typeset and directly accessible in the HTML version of the article. For details and guidance, please refer to:
embopress.org/page/journal/14602075/authorguide#expandedview

9) To facilitate reproducibilty and cross-laboratory adoption of methodologies, please structure the Materials & Methods section as outlined in our guide to authors, including a completed Reagents and Tools Table that can be downloaded from our author guidelines as well (https://www.embopress.org/page/journal/14602075/authorguide#structuredmethods).

10) Digital image enhancement is acceptable practice, as long as it accurately represents the original data and conforms to community standards. If a figure has been subjected to significant electronic manipulation, this must be clearly noted in the figure legend and/or the 'Materials and Methods' section. The editors reserve the right to request original versions of figures and the original images that were used to assemble the figure. Finally, we generally encourage uploading of numerical as well as gel/blot image source data; for details see: embopress.org/page/journal/14602075/authorguide#sourcedata

At EMBO Press, we ask authors to provide source data for the main manuscript figures. Our source data coordinator will contact you to discuss which figure panels we would need source data for and will also provide you with helpful tips on how to upload and organize the files.

Further information is available in our Guide For Authors:
https://www.embopress.org/page/journal/14602075/authorguide

Revision to The EMBO Journal should be submitted online within 90 days, unless an extension has been requested and approved by the editor; please click on the link below to submit the revision online before 20th May 2025:
Link Not Available

If you choose to alternatively have this study further considered by another EMBO Press publication, please use the following hyperlink to directly transfer the manuscript, optionally with inclusion of referee reports and identities:
Link Not Available

Referee #1:

Wierbilowicz et al. reported the identification of an ADP-ribosyl degron generated by PARP7 that controls androgen receptor (AR) degradation. The study showed that the synthetic androgen R1881 induces AR-dependent gene expression and that RBN2397, a PARP7-specific inhibitor, further increases the expression pattern observed in both up- and down-regulated genes, suggesting that PARP7 acts as a negative regulator. Several AR target genes were validated, and qPCR analysis showed that RBN2397 treatment led to elevated or sustained gene expression, supporting the role of PARP7. The authors demonstrated that R1881 treatment leads to time-dependent proteasomal degradation of AR, while RBN2397 treatment inhibits this process.

Interestingly, ADP-ribosylated AR was detected when cells were treated with a proteasome inhibitor. Knockdown of PARP7 appeared to stabilize AR. A V582F AR variant, which cannot bind DNA, was stable upon R1881 treatment and showed no ADP-ribosylation.
These findings led to a model in which androgen-bound AR, when associated with DNA, is ADP-ribosylated by PARP7, subsequently resulting in proteasomal degradation. The authors then investigated the mechanism of AR degradation. Their prior work suggests that cysteine residues are ADP-ribosylated. Using mutagenesis analysis on 11 cysteines, they found that C620 is likely an ADP-ribosylation site that primes AR for degradation. They then searched for E3 ligases that recognize the ADP-ribosyl signal and found that knockdown of DTX2 greatly enhanced AR-ADPr products relative to total AR, suggesting that DTX2 might be responsible for ubiquitination and degradation of ADP-ribosylated AR. Using an in vitro system with recombinant E1, E2, DTX2, and ubiquitin (Ub) with AR isolated from cells, they showed that only ADP-ribosylated AR was ubiquitinated by DTX2. Knockdown of DTX2 also enhanced the expression of AR target genes.
Prior studies have demonstrated that DTX2 can ubiquitinate the ADP-ribosyl moiety of ADP-ribosylated substrates directly in vitro, but evidence in cells remains elusive. The authors demonstrated that DTX2 can ubiquitinate an ADP-ribosylated cysteine peptide in vitro and attempted to show that this event can occur on ADP-ribosylated AR isolated from cells using an in vitro reaction. Based on their findings, they propose that PARP7 catalyzes ADP-ribosylation of androgen-bound AR on DNA and that ADP-ribosylation of C620 is recognized by DTX2, which in turn catalyzes ubiquitination of the ADP-ribosyl moiety on ADP-ribosylated C620. This process is required for AR degradation. This model is interesting, as current knowledge of ubiquitination of the ADP-ribosyl moiety of ADP-ribosylated substrates is limited to in vitro discoveries with no known biological relevance. This study attempts to link this mechanism to AR degradation in cells. However, there are several major concerns with the data presented that need to be addressed.

Major concerns:
1. One of the major concerns is the proposed C620 site. Eleven cysteines were selected for the mutagenesis study, with eight located on disordered loops of AR, while the remaining three (C596, C602, and C620) are positioned within the AR DNA-binding domain. The crystal structure of the AR DNA-binding domain (PDB 8RM6) shows that C596 and C602 function as zinc ligands, whereas C620 is buried and forms the hydrophobic core of the DNA-binding domain. Substituting C596 and C602 would disrupt zinc coordination and thereby impair proper folding. In this reviewer's opinion, these variants are likely to be defective in DNA binding, exhibiting a similar phenotype to the V582F variant. It remains unclear how the introduction of C620S or C620G would affect the folding and DNA-binding properties of the AR DNA-binding domain. Serine introduces a hydroxyl group, which is unfavorable within a hydrophobic core, whereas glycine increases flexibility and might alter folding. The observed defect in degradation could be due to the inability of these mutants to be recruited to androgen-responsive promoters, as the authors showed that the DNA-binding-deficient V582F variant was not degraded upon R1881 treatment. It is important to demonstrate that the C620S variant retains proper folding and DNA-binding capacity. The authors might also consider substituting C620 with amino acids that have shorter hydrophobic side chains, such as alanine or valine. In this reviewer's opinion, these substitutions could preserve folding, though this should be experimentally validated.

2. It is unclear how ADP-ribosylation could occur at C620 when it is buried. The authors suggest that DNA-bound AR might exhibit an increased solvent-accessible surface area, but this does not change the fact that C620 remains buried or only partially exposed. For PARP7 to catalyze the reaction, this cysteine must be fully accessible to its active site. It is difficult to rationalize how this reaction could take place under these conditions. Could the authors detect C620 ADP-ribosylation in AR isolated from cells, or in an in vitro reaction catalyzed by PARP7 using AR purified from cells or just the AR DNA-binding domain with or without DNA?

3. The in vitro validation of DTX2-RD-catalyzed ubiquitination of the ADPr moiety on ADPr-Cys-peptides and ADPr-AR is consistent with prior studies demonstrating that DTX2-RD can catalyze Ub-ADPr linkages. However, the in vitro AR-ADPr reaction shows that some Ub products are linked via lysine residues on AR-ADPr (resistant to NUDT16). This raises the question of whether DTX2 catalyzes ubiquitination on lysine residues, the ADPr moiety, or both in cells. Is it possible to isolate ubiquitinated AR-ADPr from cells and determine whether Ub-ADPr linkages are present? While this may be challenging, such evidence would greatly strengthen the proposed model.

4. In several western blots (Figures 3H, 4D, 5A), AR-ADPr levels remain unchanged over time following R1881 treatment, while total Flag-AR levels progressively decrease. The model suggests that AR-ADPr is ubiquitinated and degraded, yet the levels of AR-ADPr do not decline in parallel with Flag-AR. This apparent discrepancy suggests that the majority of AR is degraded via alternative mechanisms.

5. Related to the previous comment, in Figure 6B, the rate of total Flag-AR reduction appears similar between siCTRL and siDTX2 over time, but AR-ADPr persists in siDTX2-treated cells. Does this imply that the majority of Flag-AR degradation is mediated by other E3 ligases and is independent of ADPr modification or DTX2?

6. Please clarify the differences between VCaP and PC3-AR cells. Why can AR-ADPr be detected in PC3-AR cells even in the absence of proteasome inhibitor treatment?

7. What was the knockdown efficiency of shPARP7 in Figure 3H? This information was not provided.

Minor comments:
1. The method used to detect AR-ADPr is not clear until Figure 5C, where the main text describes the use of fluorescently labeled tandem AF1521. It would be helpful to mention this earlier when describing Figure 3.

2. In Figure 6D (extract lane), why does the siDTX2 lane show reduced AR-ADPr levels compared to the HA-PARP7 lane? This result appears to contradict the findings in the GSH-bead-bound lanes.

3. Figure 5H is mislabeled as Figure 5G.

Referee #2:

EMBOJ-2025-120145 by Wierbiłowicz et al. (Paschal)
"Parp7 generates an ADP-ribosyl degron that controls negative feedback of androgen signaling"

Summary
In this manuscript, the authors investigate PARP7-mediated degradation of androgen receptor in prostate cancer cells. Key findings include:
1. A large fraction of genes regulated by the synthetic androgen R1881 are altered by the PARP7 inhibitor RBN2397.
2. A subset of genes upregulated by R1881 show peak expression at ~8 hrs of treatment, then a decrease at longer timepoints. RBN2397 prevents the decrease observed at >8 hrs for these genes, suggesting this effect is a negative feedback loop driven by PARP7.
3. R1881 and DHT induce ADP-ribosylation and degradation of AR. These effects are diminished by RBN2397.
4. Mathematical modeling, structural analysis, and biochemical experiments suggest PARP7 mediates these effects through ADP-ribosylation of C620 on DNA-bound AR.
5. Knockdown of DTX2 leads to the accumulation of ADP-ribosylated AR in R1881-treated prostate cancer cells. Experiments with endogenous AR immunoprecipitate and a synthetic AR peptide indicate DTX2 binds to ADP-ribosylated AR via its DTC domain and catalyzes ubiquitination through a non-canonical linkage to the ADP-ribosyl group on AR.
6. High expression of PARP7 is associated with better survival in a cohort of prostate cancer patients.

Review
This manuscript builds upon previous work from this group indicating PARP7 regulates androgen signaling through cysteine ADP-ribosylation of androgen receptor. In particular, the authors characterize the functional outcomes of PARP7-mediated ADP-ribosylation of AR C620 and provide evidence for a non-canonical ADPr-ubiquitin conjugate catalyzed by DTX2 that mediates suppression of androgen signaling.

Strengths: The authors integrate deep sequencing, mathematical modeling, and biochemistry to provide multiple lines of evidence supporting their main claims. The identification of the ADPr-ubiquitin conjugate on AR C620 provides an example of a site-specific function for this recently discovered modification that will be of interest to the field.

Weaknesses: Additional clarifications, experiments, and analyses would enhance the work. In particular, how DTX2 contributes to AR degradation versus other E3 ligases that mediate AR turnover, given the result in Figure 6A (see comment 4 below).

Comments:
(1) What is the concentration of RBN2397 was used for the RNA-seq experiments?
(2) The authors note that R1881-induced degradation of AR occurs more rapidly in VCaP cells compared to PC3 cells (~6-8 vs. 18 hr). While proteasome inhibitor (Bortezomib) was required to detect ADP-ribosylated AR by western blot in VCaP cell lysates (Figure 3C), it was not required for detection of ADP-ribosylated AR in PC3 cell lysates (Figure 3H). Can the authors comment on why these cell-type specific differences occur? Is it related to DTX2 expression? Additionally, because there are noticeable differences between these cell lines, it would be helpful to mark which data come from which cell line in all figure panels, as was done for some experiments with PC3 cells.
(3) Previous work and Figure 1 show that PARP7 mRNA abundance increases in R1881-treated cells. In the discussion, the authors also mention that androgen induction of PARP7 is part of the pathway. There are currently no Western blots of PARP7 protein in the manuscript. Can the authors comment on whether PARP7 protein is also increased by R1881 and/or RBN? This could provide additional insight into the pathway regarding which step is "rate-limiting".
(4) The authors identify DTX2 as the ligase responsible for the AR ADP-ribosyl degron. This is inferred from the blot in Figure 6A where DTX2 knockdown leads to an accumulation of ADP-ribosylated AR. However, total FLAG-AR in this sample is diminished compared to other knockdowns. If DTX2 were the main ligase responsible for degrading AR in this system, I would expect an increase in FLAG-AR protein upon DTX2 knockdown. Can the authors comment on why AR protein levels go down instead?
(5) Two other labs have recently reported ADPr-ubiquitin modifications on endogenous proteins: PARP5 (Perrard and Smith, 2023), and PARP10 (Bejan et al., 2024). Interestingly, the modification was claimed to stabilize PARP5, while the modification degrades PARP10, as was reported here for AR. In both cases, the modification was reported to consist of K11-linked diubiquitin. Can the authors provide evidence that describes the chemical nature of the ubiquitin modification on ADPr-AR? This would provide some detail about the specificity and/or heterogeneity of this modification on a new target.

(6) On a related note, can the authors detect ubiquitin on endogenous AR? A ubiquitin blot comparing WT vs. C620G AR would give an indication of whether C620-ADPr is the major acceptor site of AR ubiquitination in this system.
(7) The Kaplan-Meier plot in Figure 7G suggests this pathway may be relevant in prostate cancer patients. Can the authors comment on whether individuals with high PARP7 expression also have lower expression of AR target genes or other markers of AR activation? Databases such as DepMap could be helpful in this regard.
Minor:
(1) There is a typo in Fig. S1A - "dexreased"
(2) The Venn diagrams in Fig. 1B are a bit unclear at first glance. I believe the data are 45% of genes that are increased by R1881 are further increased by RBN, 38% of genes decreased by R1881 are further decreased by RBN, and so on. Perhaps some additional text in this figure panel or legend explicitly stating what these four groups are would increase the clarity for the reader.
(3) The panel for figure 5H is labeled 5G
(4) The blot in Figure S6D is at a lower resolution than all others in the manuscript. Can the authors provide a higher resolution image of the data?

Referee #3:

Paschal and colleagues present a study that identifies Parp7 as a negative feedback regulator of androgen receptor (AR) gene transcription. The study makes use of a Parp7 inhibitor, and monitors sets of AR-regulated genes that undergo similar patterns of expression level changes over a time course. They classify Parp7 as a negative regulator since inhibition in many cases led to further increases or decreases in gene transcript levels. The study pursues a model in which Parp7 modifies AR with ADP-ribose on a specific Cys residue, and the Cys-ADP-ribose modification is further modified with ubiquitin by DTX2, which leads to degradation of AR. The manuscript covers a great deal of work, and the story is quite compelling and consistent with other recent data highlighting ubiquitin modification of ADP-ribose. I have several comments that are largely focused on suggestions for making the presentation more clear or indicating areas where the conclusions or results could use more explanation.

As a general comment, some of the transcription analysis could use more description for researchers not overly familiar with the data presentation. Figure 1D. Not clear what the connecting lines mean of the left side of the plots. Figure 1C and text. It is not clear what the expected overlap derived from a hypergeometric distribution means.

"We found that DNA binding increased the SASA of Cys620 from 0.0754 to 0.6631 Bohr2. There is a visible rotation of Phe584, which increases the SASA of Cys620. DNA-based induction of ADP-ribosylation site accessibility to PARP7 could provide spatial regulation that limits the reaction to chromatin-bound AR."
I could not find an image where the Phe584 rotation was illustrated. It is not clear from these numbers whether the change in SASA is substantial enough to cause a difference in accessibility to PARP7. What program was used to calculate the SASA values? I did not see this in the Methods. Have the authors considered whether ADP-ribose modification in the DNA binding domain of AR might impact AR affinity for DNA?

The computation modelling is difficult to digest and it is not clear based on the presentation that it provides a clear answer. After studying the Table of parameters for the two models, it was not clear how to understand why the fit was better for Model 1 compared to Model 2, and it is also not clear that the fit differences are all that great. Perhaps some "control" models are needed to demonstrate the robustness of this result. Figure 4B. The legend does not specify the model being displayed.

"Glycine is inactive for ADP-ribosylation, while serine (similarity to cysteine) can serve as an ADP-ribose acceptor for some PARPs."
This sentence could use a reference to specify which PARPs act on serines.

Is there a reason why mutants 5, 7, 8, 9, and 10 were not analyzed for AR-ADPr and FKBP51 levels?

Figure 5. Panel H is labeled with a G.

"Because the ADP-ribose conjugated to AR by PARP7 is also recognized by the MAR antibody (Supplementary Fig. 6D), our data indicates the DTC domain of DTX2 recognizes mono-ADP-ribose conjugated to AR."
It is not clear that the MAR binding reagent alone is the best tool to indicate mono- versus- poly-ADP-ribose.

Referee #1
Wierbilowicz et al. reported the identification of an ADP-ribosyl degron generated by PARP7 that controls androgen receptor (AR) degradation. The study showed that the synthetic androgen R1881 induces AR-dependent gene expression and that RBN2397, a PARP7-specific inhibitor, further increases the expression pattern observed in both up- and down-regulated genes, suggesting that PARP7 acts as a negative regulator. Several AR target genes were validated, and qPCR analysis showed that RBN2397 treatment led to elevated or sustained gene expression, supporting the role of PARP7. The authors demonstrated that R1881 treatment leads to time-dependent proteasomal degradation of AR, while RBN2397 treatment inhibits this process. Interestingly, ADP-ribosylated AR was detected when cells were treated with a proteasome inhibitor. Knockdown of PARP7 appeared to stabilize AR. A V582F AR variant, which cannot bind DNA, was stable upon R1881 treatment and showed no ADP-ribosylation.

These findings led to a model in which androgen-bound AR, when associated with DNA, is ADP-ribosylated by PARP7, subsequently resulting in proteasomal degradation. The authors then investigated the mechanism of AR degradation. Their prior work suggests that cysteine residues are ADP-ribosylated. Using mutagenesis analysis on 11 cysteines, they found that C620 is likely an ADP-ribosylation site that primes AR for degradation. They then searched for E3 ligases that recognize the ADP-ribosyl signal and found that knockdown of DTX2 greatly enhanced AR-ADPr products relative to total AR, suggesting that DTX2 might be responsible for ubiquitination and degradation of ADP-ribosylated AR. Using an in vitro system with recombinant E1, E2, DTX2, and ubiquitin (Ub) with AR isolated from cells, they showed that only ADP-ribosylated AR was ubiquitinated by DTX2. Knockdown of DTX2 also enhanced the expression of AR target genes.

Prior studies have demonstrated that DTX2 can ubiquitinate the ADP-ribosyl moiety of ADP-ribosylated substrates directly in vitro, but evidence in cells remains elusive. The authors demonstrated that DTX2 can ubiquitinate an ADP-ribosylated cysteine peptide in vitro and attempted to show that this event can occur on ADP-ribosylated AR isolated from cells using an in vitro reaction. Based on their findings, they propose that PARP7 catalyzes ADP-ribosylation of androgen-bound AR on DNA and that ADP-ribosylation of C620 is recognized by DTX2, which in turn catalyzes ubiquitination of the ADP-ribosyl moiety on ADP-ribosylated C620. This process is required for AR degradation. This model is interesting, as current knowledge of ubiquitination of the ADP-ribosyl moiety of ADP-ribosylated substrates is limited to in vitro discoveries with no known biological relevance. This study attempts to link this mechanism to AR degradation in cells. However, there are several major concerns with the data presented that need to be addressed.

Major concerns
1. One of the major concerns is the proposed C620 site. Eleven cysteines were selected for the mutagenesis study, with eight located on disordered loops of AR, while the remaining three (C596, C602, and C620) are positioned within the AR DNA-binding domain. The crystal structure of the AR DNA-binding domain (PDB 8RM6) shows that C596 and C602 function as zinc ligands, whereas C620 is buried and forms the hydrophobic core of the DNA-binding domain. Substituting C596 and C602 would disrupt zinc coordination and thereby impair proper folding. In this reviewer's opinion, these variants are likely to be defective in DNA binding, exhibiting a similar phenotype to the V582F variant. It remains unclear how the introduction of C620S or C620G would affect the folding and DNA-binding properties of the AR DNA-binding domain. Serine introduces a hydroxyl group, which is unfavorable within a hydrophobic core, whereas glycine increases flexibility and might alter folding. The observed defect in degradation could be due to the inability of these mutants to be recruited to androgen-responsive promoters, as the authors showed that the DNA-binding-deficient V582F variant was not degraded upon R1881 treatment. It is important to demonstrate that the C620S variant retains proper folding and DNA-binding capacity. The authors might also consider substituting C620 with amino acids that have shorter hydrophobic side chains, such as alanine or valine. In this reviewer's opinion, these substitutions could preserve folding, though this should be experimentally validated.
We agree with the reviewer that an effect of Cys620 mutation on DNA binding is logical given its position in the core of the DBD. Consistent with this possibility, our cell-based transcription data in the manuscript (Fig. 6G) shows that while the AR Cys620Ser mutant is activated by androgen (R1881), the fold-change is less (VCL gene) or much less (F3 gene) than observed with WT AR. This could reflect an effect of the mutation

on DBD binding. But despite its lower activity, we feel the Cys620Ser mutant in the manuscript provides useful information because the androgen-induced activity resists PARP7 inhibition. This result links the effect of PARP7 inhibition on AR output to Cys620. In a previous study (Nat Comm 12: 2705, 2021) we identified a total of 11 ADP-ribosylation sites on AR (androgen-induced and PARP7-specific; all cysteines), three of which are in the DBD. The ADP-ribosylation sites were identified by mass spectrometry of AR that was ADP-ribosylated by endogenous PARP7 in cells. In the same study we showed that all detectable ADP-ribose on AR can be released by mercury treatment, which gives us confidence that in prostate cancer cells, ADP-ribosylation of AR occurs primarily if not exclusively on cysteines. ADP-ribosylation sites Cys596 and Cys602 are part of the second ZF in the DBD, and the Cys620 site is C-terminal to the 4th zinc-coordinating Cys614 in the second ZF. Thus, AR can be ADP-ribosylated in cells on three different sites in the DBD. As the reviewer points out, making mutations in these ADP-ribosylation sites could/would impact DBD function. This makes it very difficult to interpret data from mutagenesis experiments.

In the revised manuscript we addressed this issue in two ways. First, we modified the manuscript to explain a scenario whereby Cys620 mutation reduces DNA binding (Results p. 15-16). Second, we provide a new experiment where the WT DBD (as the source of known ADP-ribosylation acceptor sites) was shown to mediate androgen-dependent degradation in the context of an artificial transcription factor (Fig. 6H, I). We feel this result together with the text changes provides supporting evidence for the concept of ADP-ribosylation of the DBD as a key step for AR degradation (as opposed to focusing entirely on the Cys620 mutant). Experimentally, it seems challenging to mutate the ADPr acceptor sites in the DBD without some effect on DNA binding, yet we show in the manuscript that ADP-ribosylation is strictly dependent on DNA binding (proverbial chicken-and-egg). The notion of ADPr transfer to a sulfur group that is ligand bonded to a zinc atom seems a bit mysterious, but in fact, Michael Cohen's lab showed that PARP7 can ADP-ribosylate the RNA-binding ZFs in PARP13 on cysteines; they also found that PARP7 shows proteome-wide bias for modification of cysteine (Rodriguez et al. eLife 2021), consistent with our published work on PARP7 and AR. In our view the available data shows that PARP7 can impose regulation on nuclear proteins through cysteine ADP-ribosylation, and this includes DBDs and RBDs.

2. It is unclear how ADP-ribosylation could occur at C620 when it is buried. The authors suggest that DNA-bound AR might exhibit an increased solvent-accessible surface area, but this does not change the fact that C620 remains buried or only partially exposed. For PARP7 to catalyze the reaction, this cysteine must be fully accessible to its active site. It is difficult to rationalize how this reaction could take place under these conditions. Could the authors detect C620 ADP-ribosylation in AR isolated from cells, or in an in vitro reaction catalyzed by PARP7 using AR purified from cells or just the AR DNA-binding domain with or without DNA?
As mentioned above, Cys620 is one of three ADP-ribosylation sites in the DBD identified by mass spectrometry using AR modified by endogenous PARP7 in cells (AR isolated by IP; Nat Comm 12: 2705, 2021)). We share the reviewer's outlook on the limited solvent accessibility of Cys620, which motivated our energy minimization analysis in the first submission. In the revised manuscript, we have significantly expanded this analysis by calculating solvent exposure of all three DBD Cys sites that can be ADP-ribosylated in cells, using three different AR DBD-DNA complexes from rat and human. These co-crystal structures (Shaffer et al. 2004; Lee et al. 2024) were solved DNAs with different DBD affinities: DR3 DNA (K_D=520 nM), MTV DNA (KD=120 nM), and C3 DNA (KD=20 nM). In all three structures, solvent exposure of Cys620 is increased by DNA binding, particularly in chain A of the DBD homodimer. These additional data are presented as two new Appendix Tables S5 and S6. We opted to remove the panel that visualized the SASA change in Cys620 since the tabular data is much easier for the reader to understand. With regard to the experiment suggested by the reviewer (in vitro ADP-ribosylation with recombinant PARP7 and recombinant DBD - which we like), to my knowledge no lab has succeeded in purifying biochemically active full-length PARP7. The PARP7 catalytic domain sold commercially is biochemically active in our hands, but because the PARP7 zinc finger is required for AR ADP-ribosylation in cells (Kamata et al. 2021, PMCID: PMC9482820), we feel meaningful results from this type of experiment should be done with active, full-length PARP7.

3. The in vitro validation of DTX2-RD-catalyzed ubiquitination of the ADPr moiety on ADPr-Cys-peptides and ADPr-AR is consistent with prior studies demonstrating that DTX2-RD can catalyze Ub-ADPr linkages.

However, the in vitro AR-ADPr reaction shows that some Ub products are linked via lysine residues on AR-ADPr (resistant to NUDT16). This raises the question of whether DTX2 catalyzes ubiquitination on lysine residues, the ADPr moiety, or both in cells. Is it possible to isolate ubiquitinated AR-ADPr from cells and determine whether Ub-ADPr linkages are present? While this may be challenging, such evidence would greatly strengthen the proposed model.
This is a very interesting experiment, and we agree with the reviewer it would be challenging. I think our data showing that knockdown of the E3 DTX2 results in accumulation of ADP-ribosylated AR, and that DTX2 Ub conjugation to AR requires the DTC (ADPr reader domain) and ADPr-substrate, and that Ub-AR and Ub-AR peptide conjugates are cleaved by NUDT16 (ADPr cleavage enzyme) makes a strong case that Ub conjugation occurs through ADP-ribose. Lastly, pretreatment of AR-ADPr and peptide-ADPr abolishes Ub addition. A plausible explanation for the slight resistance of Ub-ADPr to NUDT16 (noted by the reviewer) is that the adenine proximal ribose ring (conjugated to Ub) sits in the NUDT16 mouth to position ADPr for cleavage; it can be posited that Ub conjugated to the ribose impedes binding. The spatial position of the Ub linkage to ADPr (proximal ribose) is very different from that of a protein substrate linked to ADPr (distal ribose), in which case NUDT16 cleaves with high efficiency (Thirawatananond et al. 2019 "Structural analyses of NudT16-ADP-ribose complexes direct rational design of mutants with improved processing of poly(ADP-ribosyl)ated proteins, Sci Reports 9: 5940). But to the reviewer's point, we acknowledge in the discussion an alternative/complementary mechanism to what we propose, which is that ADPr could be used as a binding site for an E3 reader with Ub conjugation to a proximal lysine (akin to how phosphor degrons operate). We acknowledged in the Results that "NUDT16 resistance could be indicative of Ub transfer to a proximal lysine." And "While our data shows that Ub is conjugated through an ADP-ribose linkage, it remains possible that proximal lysines in AR undergo DTX2-dependent modification which are not detected by our assay." Having said this, our peptide experiments (K-less) formally prove the DTX2-Ub mechanism can operate in an ADP-ribose-dependent and lysine-independent manner.

4. In several western blots (Figures 3H, 4D, 5A), AR-ADPr levels remain unchanged over time following R1881 treatment, while total Flag-AR levels progressively decrease. The model suggests that AR-ADPr is ubiquitinated and degraded, yet the levels of AR-ADPr do not decline in parallel with Flag-AR. This apparent discrepancy suggests that the majority of AR is degraded via alternative mechanisms.
The kinetics of AR-ADPr formation on individual sites as related to degradation rates are unknown. ADPr can be removed from AR by cellular hydrolases (Nat Comm 2021, Fig 5G) on a time scale that appears faster (tens of minutes) than the degradation of AR, so it is reasonable to think ADPr-modified AR can move "forward" to the proteasome, and ADPr-modified AR can be hydrolyzed and thus move "backward" to stable protein form that maintains its transcription function. A caveat to measuring ADPr on AR is that it is ADP-ribosylated on multiple sites and most of these are actually dispensable for PARP7- and ADPr-mediated degradation. Ideally one would employ site specific ADPr probes (akin to phosphor-site specific antibodies), but such reagents have not been developed by the field. What we can say is that depleting the DTX2 E3 that reads ADPr on AR causes an accumulation of AR-ADPr (Fig. 7A, B) and increased expression of genes that are sensitive to AR levels (Figure 7I).

5. Related to the previous comment, in Figure 6B, the rate of total Flag-AR reduction appears similar between siCTRL and siDTX2 over time, but AR-ADPr persists in siDTX2-treated cells. Does this imply that the majority of Flag-AR degradation is mediated by other E3 ligases and is independent of ADPr modification or DTX2?
As cited in the manuscript, AR can be degraded using multiple E3 enzymes. We mention five such papers on E3 enzymes that can degrade AR but all of these studies are based on AR degradation under conditions where androgen was **not** added to the cell cultures. The rationale for our focused screen was that depleting an E3 that recognizes AR-ADPr should result in the accumulation of AR-ADPr; this is conceptually similar to bortezomib treatment, which also accumulates AR-ADPr because it is an intermediate in the degradation pathway of androgen-bound AR. Conceivably, a different form of AR (ligand-free, ligand-bound, ADPr-modified, phosphorylated, sumoylated, mono-Ub) could have molecular/chemical features relevant to degradation (or stabilization) that are context-specific (cellular compartment, chromatin sites, signaling state, condensates, etc). We don't think our data (or others') allows us to state the percentage of AR degraded by a specific E3. What we can say for certain is that depletion of DTX2 increases AR-mediated gene expression, and that the striking androgen-induced degradation of AR protein in VCaP cells is

significantly reduced with a selective PARP7 inhibitor. Through these studies, we have identified a new mechanism that controls AR protein level in prostate cancer cells.

6. Please clarify the differences between VCaP and PC3-AR cells. Why can AR-ADPr be detected in PC3-AR cells even in the absence of proteasome inhibitor treatment?
PC3-AR cells are a derivative of PC3 cells where we re-introduced WT AR (stable cell line). This provides a platform for testing AR mutants without interference from endogenous AR. This is a critical consideration because AR assembles as a dimer on chromatin. Multiple labs in the AR field have used this PC3 cell line for the same reason. VCaP cells express endogenous, full-length AR and a low level of a splice variant (V7) that lacks the AR ligand-binding domain which in patients makes it resistant to androgen-based therapy. ADP-ribosylated AR is easier to detect in PC3-AR cells because AR and PARP7 expression levels are higher than VCaP cells.

7. What was the knockdown efficiency of shPARP7 in Figure 3H? This information was not provided.
We now state in the Methods that the PARP7 knockdown efficiency was 50-70%. We have reported these numbers in previous PARP7 papers (e.g. Cancer Res Commun. 2023 Apr 17;3(4):592-606).

Minor comments
1. The method used to detect AR-ADPr is not clear until Figure 5C, where the main text describes the use of fluorescently labeled tandem AF1521. It would be helpful to mention this earlier when describing Figure 3. We apologize for the omission and now mention the probe and cite the method (Yang *et al.*, 2021) when presenting Fig. 3. We also provide details on fluorescently-labeled AF1521 in the methods section.

2. In Figure 6D (extract lane), why does the siDTX2 lane show reduced AR-ADPr levels compared to the HA-PARP7 lane? This result appears to contradict the findings in the GSH-bead-bound lanes.
The experiment involves two cell lines, both expressing Flag-AR. Lane 1 shows the PC3-AR/HA-PARP7 cell line and lane 2 shows PC3-AR/siDTX2. In response to R1881 treatment, the HA-PARP7 expressing cell lines shows a higher level of total ADPr on AR, but multiple sites contribute to the total signal - not just the sites linked to degradation. When DTX2 is depleted, which our data indicates stabilizing AR-ADPr destined for degradation, the AR pulled down on the DTC reader domain contains a higher level of total ADPr. The pulldown is specific for AR-ADPr since mutation of the DTC eliminated the recovery of AR-ADPr on the beads. These data help make the point that there is an ADPr-modified pool of AR generated by PARP7, but a subset of the pool is recognized by the DTC domain which we propose including ADPr site(s) in the DBD that help specify AR degradation.

3. Figure 5H is mislabeled as Figure 5G. We corrected the label.

Referee #2
EMBOJ-2025-120145 by Wierbiłowicz et al. (Paschal) "Parp7 generates an ADP-ribosyl degron that controls negative feedback of androgen signaling"
Summary. In this manuscript, the authors investigate PARP7-mediated degradation of androgen receptor in prostate cancer cells. Key findings include:
1. A large fraction of genes regulated by the synthetic androgen R1881 are altered by the PARP7 inhibitor RBN2397.
2. A subset of genes upregulated by R1881 show peak expression at ~8 hrs of treatment, then a decrease at longer timepoints. RBN2397 prevents the decrease observed at >8 hrs for these genes, suggesting this effect is a negative feedback loop driven by PARP7.
3. R1881 and DHT induce ADP-ribosylation and degradation of AR. These effects are diminished by RBN2397.
4. Mathematical modeling, structural analysis, and biochemical experiments suggest PARP7 mediates these effects through ADP-ribosylation of C620 on DNA-bound AR.
5. Knockdown of DTX2 leads to the accumulation of ADP-ribosylated AR in R1881-treated prostate cancer cells. Experiments with endogenous AR immunoprecipitate and a synthetic AR peptide indicate DTX2 binds to ADP-ribosylated AR via its DTC domain and catalyzes ubiquitination through a non-canonical linkage to the ADP-ribosyl group on AR.

6. High expression of PARP7 is associated with better survival in a cohort of prostate cancer patients.

Review

This manuscript builds upon previous work from this group indicating PARP7 regulates androgen signaling through cysteine ADP-ribosylation of androgen receptor. In particular, the authors characterize the functional outcomes of PARP7-mediated ADP-ribosylation of AR C620 and provide evidence for a non-canonical ADPr-ubiquitin conjugate catalyzed by DTX2 that mediates suppression of androgen signaling.

Strengths: The authors integrate deep sequencing, mathematical modeling, and biochemistry to provide multiple lines of evidence supporting their main claims. The identification of the ADPr-ubiquitin conjugate on AR C620 provides an example of a site-specific function for this recently discovered modification that will be of interest to the field.

Weaknesses: Additional clarifications, experiments, and analyses would enhance the work. In particular, how DTX2 contributes to AR degradation versus other E3 ligases that mediate AR turnover, given the result in Figure 6A (see comment 4 below).

Comments:

(1) What is the concentration of RBN2397 was used for the RNA-seq experiments?

100 nM (now stated in methods)

(2) The authors note that R1881-induced degradation of AR occurs more rapidly in VCaP cells compared to PC3 cells (~6-8 vs. 18 hr). While proteasome inhibitor (Bortezomib) was required to detect ADP-ribosylated AR by western blot in VCaP cell lysates (Figure 3C), it was not required for detection of ADP-ribosylated AR in PC3 cell lysates (Figure 3H). Can the authors comment on why these cell-type specific differences occur? Is it related to DTX2 expression? Additionally, because there are noticeable differences between these cell lines, it would be helpful to mark which data come from which cell line in all figure panels, as was done for some experiments with PC3 cells.

While we don't have a complete understanding of the cell-type differences in the AR-PARP7 axis, we do know that ADP-ribosylated AR is easier to detect in PC3-AR cells because AR and PARP7 expression levels are higher than VCaP cells. Even though the mechanism is relatively straightforward, there are certainly multiple ways the two cell types could differ, including the level of ADPr hydrolyases, DUBs, E2s, and the fact that AR transcription is repressed by LSD1 in VCaP cells. We specified the cell type used in the figure legends.

(3) Previous work and Figure 1 show that PARP7 mRNA abundance increases in R1881-treated cells. In the discussion, the authors also mention that androgen induction of PARP7 is part of the pathway. There are currently no Western blots of PARP7 protein in the manuscript. Can the authors comment on whether PARP7 protein is also increased by R1881 and/or RBN? This could provide additional insight into the pathway regarding which step is "rate-limiting".

We published androgen-induction of endogenous PARP7 and RBN2397 stabilization of PARP7 protein in two previous papers (Nat Commun. 2021 May 11;12(1):2705; Cancer Res Commun. 2023 Apr 17;3(4):592-606. Without adding androgen to the culture, prostate cancer cell lines express a low level of PARP7 protein which can be nearly undetectable by blotting because of its short protein half-life (10.3390/cells10020363).

(4) The authors identify DTX2 as the ligase responsible for the AR ADP-ribosyl degron. This is inferred from the blot in Figure 6A where DTX2 knockdown leads to an accumulation of ADP-ribosylated AR. However, total FLAG-AR in this sample is diminished compared to other knockdowns. If DTX2 were the main ligase responsible for degrading AR in this system, I would expect an increase in FLAG-AR protein upon DTX2 knockdown. Can the authors comment on why AR protein levels go down instead?

In panel Fig. 6A the FLAG-AR:tubulin ratio is 0.92, a slight reduction that could be real or within the range of error for the methods. But we agree with the reviewer's expectation that FLAG-AR levels should increase if DTX2 is the primary ligase responsible for degrading bulk AR. The fact that FLAG-AR does not increase - but AR-ADPr increases about 10-fold – indicates that DTX2 is responsible for selectively degrading the ADP-ribosylated form of AR. In the same cell line/experiment, parallel knockdown of the HUWE1 E3 ligase

(published E3 for AR) increases FLAG-AR levels 2-fold with a minimal effect on AR-ADPr. Clearly, AR in prostate cancer cells is targeted by multiple E3 enzymes, but from our screen and other panels in the figure, DTX2 is limiting for AR-ADPr levels in the cell. Together with our other data, we conclude DTX2 is critical for controlling turnover of the ADP-ribosylated pool of AR generated by PARP7. We state in the manuscript that AR is handled by multiple E3s.

(5) Two other labs have recently reported ADPr-ubiquitin modifications on endogenous proteins: PARP5 (Perrard and Smith, 2023), and PARP10 (Bejan et al., 2024). Interestingly, the modification was claimed to stabilize PARP5, while the modification degrades PARP10, as was reported here for AR. In both cases, the modification was reported to consist of K11-linked diubiquitin. Can the authors provide evidence that describes the chemical nature of the ubiquitin modification on ADPr-AR? This would provide some detail about the specificity and/or heterogeneity of this modification on a new target.
Based on the existing knowledge base we infer that Ub is linked to the proximal ribose as shown in Figure 8B. This derives from the careful characterization of the Ub linkage to the 3' OH of the adenine proximal ribose ring by the Ahel group (Zhu et al. 2022, cited in our ms). We believe that experiments to study the chemical details of the Ub linkage to ADPr-AR (USP2 and NUDT16 were tested and shown) are beyond the scope of the present study. Having said this, we totally agree with the reviewer that defining the linkage(s) is the next step for detailed analysis of the degron. This question really should be attacked both in vitro and using cell-derived samples.

(6) On a related note, can the authors detect ubiquitin on endogenous AR? A ubiquitin blot comparing WT vs. C620G AR would give an indication of whether C620-ADPr is the major acceptor site of AR ubiquitination in this system.
We have had a difficult time consistently detecting ubiquitylated AR from cells, despite using the standard approaches including over-expression of tagged ubiquitin and purification in guanidinium chloride. Given the challenges of interpreting DBD mutants (e.g. C620G, that might indirectly reduce ADP-ribosylation), we provided a new experiment with a WT AR DBD and show that it mediates androgen-dependent degradation of an artificial transcription factor (VP16-DBD-LBD; Fig. 6H, I).

(7) The Kaplan-Meier plot in Figure 7G suggests this pathway may be relevant in prostate cancer patients. Can the authors comment on whether individuals with high PARP7 expression also have lower expression of AR target genes or other markers of AR activation? Databases such as DepMap could be helpful in this regard.
The response to androgen pathway analysis (now Fig. 8G) shows the relationship between "overall" androgen signaling score (calculated by PARADIGM using the TCGA-PARD cohort) and PARP7 message levels (RNA-seq) in 478 prostate cancer patients. We believe this provides the best overall measure of how PARP7 affects AR activity in prostate tumor samples. While individual genes such as PSA were once viewed as suitable biomarkers of AR activity, it is now widely appreciated that an integrated (pathway) assessment is more meaningful. There is a small negative correlation between PARP7 levels and androgen signaling (Fig. 8F; consistent with our model). The contributions of other pathway components (co-activators, co-repressors) to androgen signaling make it difficult to assess the effect of a single gene.

Minor:
(1) There is a typo in Fig. S1A - "dexreased"
Corrected – thank you.

(2) The Venn diagrams in Fig. 1B are a bit unclear at first glance. I believe the data are 45% of genes that are increased by R1881 are further increased by RBN, 38% of genes decreased by R1881 are further decreased by RBN, and so on. Perhaps some additional text in this figure panel or legend explicitly stating what these four groups are would increase the clarity for the reader.
Added to the legend: The Venn diagrams on the right show PARP7 inhibition with RBN2397 affects the expression of R1881-sensitive genes, whether they are R1881-induced or R1881-repressed. The largest group of genes affected by RBN2397 treatment are those which are positively regulated by R1881 and undergo a further increase (45%). The second largest group are negatively regulated by R1881 and

undergo a further decrease (38%). We also show genes that are upregulated by R1881 but downregulated by RBN2397, and vice versa.

(3) The panel for figure 5H is labeled 5G
Corrected – thank you.

(4) The blot in Figure S6D is at a lower resolution than all others in the manuscript. Can the authors provide a higher resolution image of the data?
This was fixed – thank you.

Referee #3
Paschal and colleagues present a study that identifies Parp7 as a negative feedback regulator of androgen receptor (AR) gene transcription. The study makes use of a Parp7 inhibitor, and monitors sets of AR-regulated genes that undergo similar patterns of expression level changes over a time course. They classify Parp7 as a negative regulator since inhibition in many cases led to further increases or decreases in gene transcript levels. The study pursues a model in which Parp7 modifies AR with ADP-ribose on a specific Cys residue, and the Cys-ADP-ribose modification is further modified with ubiquitin by DTX2, which leads to degradation of AR. The manuscript covers a great deal of work, and the story is quite compelling and consistent with other recent data highlighting ubiquitin modification of ADP-ribose. I have several comments that are largely focused on suggestions for making the presentation more clear or indicating areas where the conclusions or results could use more explanation.

As a general comment, some of the transcription analysis could use more description for researchers not overly familiar with the data presentation. Figure 1D. Not clear what the connecting lines mean of the left side of the plots. Figure 1C and text. It is not clear what the expected overlap derived from a hypergeometric distribution means.
We expanded the explanations for the gene expression analysis in Fig.1, which we think will help make it more accessible to non-specialists. We also added the following text to the figure legend to further explain the Venn diagrams. “The Venn diagrams on the right show PARP7 inhibition with RBN2397 affects the expression of R1881-sensitive genes, whether they are R1881-induced or R1881-repressed. The largest group of genes affected by RBN2397 treatment are those which are positively regulated by R1881 and undergo a further increase (45%). The second largest group are negatively regulated by R1881 and undergo a further decrease (38%). We also show genes that are upregulated by R1881 but downregulated by RBN2397, and vice versa.”

"We found that DNA binding increased the SASA of Cys620 from 0.0754 to 0.6631 Bohr2. There is a visible rotation of Phe584, which increases the SASA of Cys620. DNA-based induction of ADP-ribosylation site accessibility to PARP7 could provide spatial regulation that limits the reaction to chromatin-bound AR."
I could not find an image where the Phe584 rotation was illustrated. It is not clear from these numbers whether the change in SASA is substantial enough to cause a difference in accessibility to PARP7. What program was used to calculate the SASA values? I did not see this in the Methods. Have the authors considered whether ADP-ribose modification in the DNA binding domain of AR might impact AR affinity for DNA?
We agree the panel (molecular model) showing SASA of Cys620 was not very helpful to the reader, and the effect on Phe584 was not obvious. The panel was removed in favor of showing expanded SASA data (tables) from three different AR DBD-DNA structures. In all three structures, solvent exposure of Cys620 is increased by DNA binding, particularly in chain A of the DBD homodimer. These additional data are presented as two new Appendix Tables S5 and S6. We opted to remove the panel that visualized the SASA change in Cys620 since the tabular data is much easier for the reader.

The computation modelling is difficult to digest and it is not clear based on the presentation that it provides a clear answer. After studying the Table of parameters for the two models, it was not clear how to understand why the fit was better for Model 1 compared to Model 2, and it is also not clear that the fit differences are all

that great. Perhaps some "control" models are needed to demonstrate the robustness of this result. Figure 4B. The legend does not specify the model being displayed.
We expanded the mathematical modeling of negative feedback (which is now a separate Fig. 4) which allowed us to do a more thorough job of walking through the scenarios tested. These analyses led us to a chromatin-based model of negative feedback, which has the lowest Sum of Squares Error (SSE) for the top 100 simulations. The results of the modeling suggested that PARP7 ADP-ribosylates AR on chromatin, which we verified in cells (Fig. 5).

"Glycine is inactive for ADP-ribosylation, while serine (similarity to cysteine) can serve as an ADP-ribose acceptor for some PARPs." This sentence could use a reference to specify which PARPs act on serines.
We now cite Suskiewicz *et al*, 2021 as a review on amino acids that can accept ADP-ribose.

Is there a reason why mutants 5, 7, 8, 9, and 10 were not analyzed for AR-ADPr and FKBP51 levels?
The reviewer points out that for a subset of the AR mutants analyzed and shown in Appendix data (Mut 5, 7, 8, 9, 10), we only blotted for AR levels and tubulin, and not ADPr and FKBP51. During this part of the study, the goal was to identify sites in AR that were necessary for R1881-induced degradation indicated by FLAG-AR detection. The results from these mutants were used to make predictions and design additional mutants, for which we do show ADPr and FKBP1 blotting in the manuscript. Thus, ADPr and FKBP1 blotting are shown for WT and six mutants (V582F, Mut 1, Mut 2, Mut 3, Mut 4, Mut 6).

Figure 5. Panel H is labeled with a G. This was corrected – thank you.

"Because the ADP-ribose conjugated to AR by PARP7 is also recognized by the MAR antibody (Appendix Fig. S6D), our data indicates the DTC domain of DTX2 recognizes mono-ADP-ribose conjugated to AR." It is not clear that the MAR binding reagent alone is the best tool to indicate mono- versus- poly-ADP-ribose.
We agree with the reviewer and changed the text to read "our data suggests the DTC domain of DTX2 recognizes mono-ADP-ribose conjugated to AR. It is also formally possible that the ADP-ribose is in the form of a short chain that does not cause a gel shift."

Dr. Bryce M Paschal
University of Virginia School of Medicine
Biochemistry and Molecular Genetics
Box 800577 Health Systems
Charlottesville, VA 22908

24th Jun 2025

Re: EMBOJ-2025-120145R
Parp7 generates an ADP-ribosyl degron that controls negative feedback of androgen signaling

Dear Bryce,

Thank you for submitting your revised manuscript to The EMBO Journal. We have now received re-reviews from original referees 2 and 3 (copied below), in light of which we shall be happy to accept the study for publication, as soon as a few remaining editorial issues have been addressed:

- Please make sure to explicitly mention, in each respective figure legend, that (and why) control micrographs are re-displayed in Figure 5D and Appendix Figure S4.

- Please move the Funding information given in the manuscript text into the 'Acknowledgements' section. Please rename the 'Material & Methods' section simply as 'Methods'.

- Please carefully go through the reference list and make sure that each reference is complete with citation year, volume, and page/elocator numbers; or in case of databases, access information.
Please also adjust the format for citation of preprints as specified in our author guidelines:
The citation in the text should be: "(preprint: NAME1 et al, YEAR)"
The citation in the reference list: "Author NAME1, Author NAME2, ... (YEAR) article title. PLATFORM NAME, doi: XXX"
Please, refer to our detailed online guidelines for further information:
https://www.embopress.org/page/journal/14602075/authorguide#referencesformat

- Please note that the Data Availability section should only list datasets generated as part of the present study (the reviewer's token should be removed now), while all previously generated datasets should only appear in the Data Citation section, and be cited with the prefix "Data ref: XXX" when being described in text (results and/or methods). Again, please refer to our detailed online guidelines for further information: https://www.embopress.org/page/journal/14602075/authorguide#referencesformat

- On the first page of the Appendix PDF, please include manuscript title and author names.

- Finally, please provide suggestions for a short 'blurb' text prefacing and summing up the conceptual aspect of the study in two sentences (max. 250 characters), followed by 3-5 one-sentence 'bullet points' with brief factual statements of key results of the paper; they will form the basis of an editor-written 'Synopsis' accompanying the online version of the article. Please also upload a synopsis image, which can be used as a "visual title" for the synopsis section of your paper. The image should be in PNG or JPG format, and please make sure that it remains in the modest dimensions of (exactly) 550 pixels wide and 300-600 pixels high.

I am returning the manuscript to you for a final round of minor revision, solely to allow you to make these modifications and upload the revised files. Once we will have received them, we should hopefully be ready to swiftly proceed with formal acceptance and production of the manuscript.

With kind regards,

Hartmut

Hartmut Vodermaier, PhD
Senior Editor, The EMBO Journal
h.vodermaier@embojournal.org

*** PLEASE NOTE: All revised manuscript are subject to initial checks for completeness and adherence to our formatting guidelines. Revisions may be returned to the authors and delayed in their editorial re-evaluation if they fail to comply to the

following requirements (see also our Guide to Authors for further information):

1) Every manuscript requires a Data Availability section (even if only stating that no deposited datasets are included). Primary datasets or computer code produced in the current study have to be deposited in appropriate public repositories prior to resubmission, and reviewer access details provided in case that public access is not yet allowed. Further information: embopress.org/page/journal/14602075/authorguide#dataavailability

2) Each figure legend must specify
- size of the scale bars that are mandatory for all micrograph panels
- the statistical test used to generate error bars and P-values
- the type error bars (e.g., S.E.M., S.D.)
- the number (n) and nature (biological or technical replicate) of independent experiments underlying each data point
- Figures may not include error bars for experiments with n<3; scatter plots showing individual data points should be used instead.

3) Revised manuscript text (including main tables, and figure legends for main and EV figures) has to be submitted as editable text file (e.g., .docx format). We encourage highlighting of changes (e.g., via text color) for the referees' reference.

4) Each main and each Expanded View (EV) figure should be uploaded as individual production-quality files (preferably in .eps, .tif, .jpg formats). For suggestions on figure preparation/layout, please refer to our Figure Preparation Guidelines: http://bit.ly/EMBOPressFigurePreparationGuideline

5) Point-by-point response letters should include the original referee comments in full together with your detailed responses to them (and to specific editor requests if applicable), and also be uploaded as editable (e.g., .docx) text files.

6) Please complete our Author Checklist, and make sure that information entered into the checklist is also reflected in the manuscript; the checklist will be available to readers as part of the Review Process File. A download link is found at the top of our Guide to Authors: embopress.org/page/journal/14602075/authorguide

7) All authors listed as (co-)corresponding need to deposit, in their respective author profiles in our submission system, a unique ORCiD identifier linked to their name. Please see our Guide to Authors for detailed instructions.

8) Please note that supplementary information at EMBO Press has been superseded by the 'Expanded View' for inclusion of additional figures, tables, movies or datasets; with up to five EV Figures being typeset and directly accessible in the HTML version of the article. For details and guidance, please refer to: embopress.org/page/journal/14602075/authorguide#expandedview

9) To facilitate reproducibilty and cross-laboratory adoption of methodologies, please structure the Materials & Methods section as outlined in our guide to authors, including a completed Reagents and Tools Table that can be downloaded from our author guidelines as well (https://www.embopress.org/page/journal/14602075/authorguide#structuredmethods).

10) Digital image enhancement is acceptable practice, as long as it accurately represents the original data and conforms to community standards. If a figure has been subjected to significant electronic manipulation, this must be clearly noted in the figure legend and/or the 'Materials and Methods' section. The editors reserve the right to request original versions of figures and the original images that were used to assemble the figure. Finally, we generally encourage uploading of numerical as well as gel/blot image source data; for details see: embopress.org/page/journal/14602075/authorguide#sourcedata

At EMBO Press we ask authors to provide source data for the main manuscript figures. You will receive a separate email with instructions for providing source data with your revised manuscript, including how to upload and organize the files.

Further information is available in our Guide For Authors: https://www.embopress.org/page/journal/14602075/authorguide

In the interest of ensuring the conceptual advance provided by the work, we recommend submitting a revision within 3 months (22nd Sep 2025). Please discuss the revision progress ahead of this time with the editor if you require more time to complete the revisions. Use the link below to submit your revision:

Link Not Available

Referee #2:

In their revised manuscript, the authors have addressed a number of my previous concerns, primarily through explanation rather than the addition of new data. Based on their descriptions, it appears that technical limitations have prevented them from demonstrating and testing obvious endpoints (e.g., detecting ubiquitylated AR; chemical nature of the ubiquitin modification on ADPR-AR). This is a limitation of the paper. Overall, I think that the paper has been improved, but perhaps not as much as it could have been. Nonetheless, this is a timely paper that makes interesting conclusions that are supported reasonably well by the data. ADPR-Ub is an exploding area of PARP research, so I think the field would generally be open to and interested in this work.

Referee #3:

The authors have addressed the concerns that I raised during the initial review of the study.

All editorial and formatting issues were resolved by the authors.

EMBO Press Author Checklist

Corresponding Author Name: Bryce Paschal	
Journal Submitted to: EMBO Journal	
Manuscript Number: EMBOJ-2025-120145	

USEFUL LINKS FOR COMPLETING THIS FORM
The EMBO Journal - Author Guidelines
EMBO Reports - Author Guidelines
Molecular Systems Biology - Author Guidelines
EMBO Molecular Medicine - Author Guidelines

Reporting Checklist for Life Science Articles (updated January 2022)
This checklist is adapted from Materials Design Analysis Reporting (MDAR) Checklist for Authors. MDAR establishes a minimum set of requirements in transparent reporting in the life sciences (see Statement of Task: 10.31222/osf.io/9sm4x). Please follow the journal's guidelines in preparing your manuscript.
Please note that a copy of this checklist will be published alongside your article.

Abridged guidelines for figures
1. Data
The data shown in figures should satisfy the following conditions:
- the data were obtained and processed according to the field's best practice and are presented to reflect the results of the experiments in an accurate and unbiased manner.
- ideally, figure panels should include only measurements that are directly comparable to each other and obtained with the same assay.
- plots include clearly labeled error bars for independent experiments and sample sizes. Unless justified, error bars should not be shown for technical
- if n<5, the individual data points from each experiment should be plotted. Any statistical test employed should be justified.
- Source Data should be included to report the data underlying figures according to the guidelines set out in the authorship guidelines on Data Presentation.

2. Captions
Each figure caption should contain the following information, for each panel where they are relevant:
- a specification of the experimental system investigated (eg cell line, species name).
- the assay(s) and method(s) used to carry out the reported observations and measurements.
- an explicit mention of the biological and chemical entity(ies) that are being measured.
- an explicit mention of the biological and chemical entity(ies) that are altered/varied/perturbed in a controlled manner.
- the exact sample size (n) for each experimental group/condition, given as a number, not a range;
- a description of the sample collection allowing the reader to understand whether the samples represent technical or biological replicates (including how many animals, litters, cultures, etc.).
- a statement of how many times the experiment shown was independently replicated in the laboratory.
- definitions of statistical methods and measures:

  - common tests, such as t-test (please specify whether paired vs. unpaired), simple χ2 tests, Wilcoxon and Mann-Whitney tests, can be unambiguously identified by name only, but more complex techniques should be described in the methods section;
  - are tests one-sided or two-sided?
  - are there adjustments for multiple comparisons?
  - exact statistical test results, e.g., P values = x but not P values < x;
  - definition of 'center values' as median or average;
  - definition of error bars as s.d. or s.e.m.

Please complete ALL of the questions below.
Select "Not Applicable" only when the requested information is not relevant for your study.

Materials

Newly Created Materials	Information included in the manuscript?	In which section is the information available? (Reagents and Tools Table, Materials and Methods, Figures, Data Availability Section)
New materials and reagents need to be available; do any restrictions apply?	Yes	Plasmids and cell lines described in Reagents and Tool Table, Materials and Methods

Antibodies	Information included in the manuscript?	In which section is the information available? (Reagents and Tools Table, Materials and Methods, Figures, Data Availability Section)
For **antibodies** provide the following information:<br>- Commercial antibodies: RRID (if possible) or supplier name, catalogue number and or/clone number<br>- Non-commercial: RRID or citation	Yes	Reagents and Tool Table, Materials and Methods

DNA and RNA sequences	Information included in the manuscript?	In which section is the information available? (Reagents and Tools Table, Materials and Methods, Figures, Data Availability Section)
**Short novel DNA or RNA including primers, probes**: provide the sequences.	Yes	Reagents and Tool Table, Materials and Methods

Cell materials	Information included in the manuscript?	In which section is the information available? (Reagents and Tools Table, Materials and Methods, Figures, Data Availability Section)
**Cell lines:** Provide species information, strain. Provide accession number in repository **OR** supplier name, catalog number, clone number, and/**OR** RRID.	Yes	Reagents and Tool Table, Materials and Methods
**Primary cultures:** Provide species, strain, sex of origin, genetic modification status.	Not Applicable	
Report if the cell lines were recently **authenticated** (e.g., by STR profiling) and tested for mycoplasma contamination.	Not Applicable	

Experimental animals	Information included in the manuscript?	In which section is the information available? (Reagents and Tools Table, Materials and Methods, Figures, Data Availability Section)
**Laboratory animals or Model organisms:** Provide species, strain, sex, age, genetic modification status. Provide accession number in repository **OR** supplier name, catalog number, clone number, **OR** RRID.	Not Applicable	
**Animal observed in or captured from the field:** Provide species, sex, and age where possible.	Not Applicable	
Please detail **housing and husbandry conditions**.	Not Applicable	

Plants and microbes	Information included in the manuscript?	In which section is the information available? (Reagents and Tools Table, Materials and Methods, Figures, Data Availability Section)
**Plants:** provide species and strain, ecotype and cultivar where relevant, unique accession number if available, and source (including location for collected wild specimens).	Not Applicable	
**Microbes:** provide species and strain, unique accession number if available, and source.	Not Applicable	

Human research participants	Information included in the manuscript?	In which section is the information available? (Reagents and Tools Table, Materials and Methods, Figures, Data Availability Section)
If collected and within the bounds of privacy constraints report on age, sex and gender or ethnicity for all study participants.	Not Applicable	

Core facilities	Information included in the manuscript?	In which section is the information available? (Reagents and Tools Table, Materials and Methods, Figures, Data Availability Section)
If your work benefited from core facilities, was their service mentioned in the acknowledgments section?	Not Applicable	

Design

Study protocol	Information included in the manuscript?	In which section is the information available? (Reagents and Tools Table, Materials and Methods, Figures, Data Availability Section)
If study protocol has been **pre-registered, provide DOI in the manuscript**. For clinical trials, provide the trial registration number **OR** cite DOI.	Not Applicable	
Report the **clinical trial registration number** (at ClinicalTrials.gov or equivalent), where applicable.	Not Applicable	

Laboratory protocol	Information included in the manuscript?	In which section is the information available? (Reagents and Tools Table, Materials and Methods, Figures, Data Availability Section)
Provide DOI OR other citation details if **external detailed step-by-step protocols** are available.	Not Applicable	

Experimental study design and statistics	Information included in the manuscript?	In which section is the information available? (Reagents and Tools Table, Materials and Methods, Figures, Data Availability Section)
Include a statement about **sample size** estimate even if no statistical methods were used.	Yes	Materials and Methods
Were any steps taken to minimize the effects of subjective bias when allocating animals/samples to treatment (e.g. **randomization procedure**)? If yes, have they been described?	Not Applicable	
Include a statement about **blinding** even if no blinding was done.	Not Applicable	
Describe **inclusion/exclusion criteria** if samples or animals were excluded from the analysis. Were the criteria pre-established?<br><br>If sample or data points were omitted from analysis, report if this was due to attrition or intentional exclusion and provide justification.	Not Applicable	
For every figure, are **statistical tests** justified as appropriate? Do the data meet the assumptions of the tests (e.g., normal distribution)? Describe any methods used to assess it. Is there an estimate of variation within each group of data? Is the variance similar between the groups that are being statistically compared?	Not Applicable	

Sample definition and in-laboratory replication	Information included in the manuscript?	In which section is the information available? (Reagents and Tools Table, Materials and Methods, Figures, Data Availability Section)
In the figure legends: state number of times the experiment was **replicated** in laboratory.	Not Applicable	
In the figure legends: define whether data describe **technical or biological replicates**.	Not Applicable	

Ethics

Ethics	Information included in the manuscript?	In which section is the information available? (Reagents and Tools Table, Materials and Methods, Figures, Data Availability Section)
Studies involving **human participants**: State details of **authority granting ethics approval** (IRB or equivalent committee(s), provide reference number for approval.	Not Applicable	
Studies involving **human participants**: Include a statement confirming that **informed consent** was obtained from all subjects and that the experiments conformed to the principles set out in the WMA Declaration of Helsinki and the Department of Health and Human Services Belmont Report.	Not Applicable	
Studies involving **human participants:** For publication of **patient photos**, include a statement confirming that consent to publish was obtained.	Not Applicable	
Studies involving experimental **animals**: State details of **authority granting ethics approval** (IRB or equivalent committee(s), provide reference number for approval. Include a statement of compliance with ethical regulations.	Not Applicable	
Studies involving **specimen and field samples:** State if relevant **permits** obtained, provide details of authority approving study; if none were required, explain why.	Not Applicable	

Dual Use Research of Concern (DURC)	Information included in the manuscript?	In which section is the information available? (Reagents and Tools Table, Materials and Methods, Figures, Data Availability Section)
Could your study fall under dual use research restrictions? Please check biosecurity documents and list of **select agents and toxins** (CDC): https://www.selectagents.gov/sat/list.htm	Not Applicable	
If you used a select agent, is the security level of the lab appropriate and reported in the manuscript?	Not Applicable	
If a study is subject to dual use research of concern regulations, is the name of the **authority granting approval and reference number** for the regulatory approval provided in the manuscript?	Not Applicable	

Reporting

The MDAR framework recommends adoption of discipline-specific guidelines, established and endorsed through community initiatives. Journals have their own policy about requiring specific guidelines and recommendations to complement MDAR.

Adherence to community standards	Information included in the manuscript?	In which section is the information available? (Reagents and Tools Table, Materials and Methods, Figures, Data Availability Section)
State if relevant guidelines or checklists (e.g., **ICMJE, MIBBI, ARRIVE, PRISMA**) have been followed or provided.	Not Applicable	
For **tumor marker prognostic studies,** we recommend that you follow the **REMARK** reporting guidelines (see link list at top right). See author guidelines, under 'Reporting Guidelines'. Please confirm you have followed these guidelines.	Not Applicable	
For **phase II and III randomized controlled trials**, please refer to the **CONSORT** flow diagram (see link list at top right) and submit the CONSORT checklist (see link list at top right) with your submission. See author guidelines, under 'Reporting Guidelines'. Please confirm you have submitted this list.	Not Applicable	

Data Availability

Data availability	Information included in the manuscript?	In which section is the information available? (Reagents and Tools Table, Materials and Methods, Figures, Data Availability Section)
Have **primary datasets** been deposited according to the journal's guidelines (see 'Data Deposition' section) and the respective accession numbers provided in the Data Availability Section?	Yes	Reagents and Tool Table, Materials and Methods
Were **human clinical and genomic datasets** deposited in a public access-controlled repository in accordance to ethical obligations to the patients and to the applicable consent agreement?	Not Applicable	
Are **computational models** that are central and integral to a study available without restrictions in a machine-readable form? Were the relevant accession numbers or links provided?	Not Applicable	
If publicly available data were reused, provide the respective **data citations in the reference list**.	Yes	
